# Fluorogenic Platform for Real-Time Imaging of Subcellular Payload Release in Antibody–Drug Conjugates

**DOI:** 10.1021/jacs.4c16842

**Published:** 2025-02-18

**Authors:** Ferran Nadal-Bufi, Paulin L. Salomon, Fabio de Moliner, Kathy A. Sarris, Zhi Wang, Rachel D. Wills, Violeta L. Marin, Xiaona Shi, Kuo Zhou, Zhongyuan Wang, Zhou Xu, Michael J. McPherson, Christopher C. Marvin, Adrian D. Hobson, Marc Vendrell

**Affiliations:** † Centre for Inflammation Research, The University of Edinburgh, Edinburgh EH16 4UU, U.K.; ‡ IRR Chemistry Hub, Institute for Regeneration and Repair, The University of Edinburgh, Edinburgh EH16 4UU, U.K.; § AbbVie Bioresearch Center, 381 Plantation Street, Worcester, Massachusetts 01605, United States; ∥ AbbVie Inc., 1 North Waukegan Road, North Chicago, Illinois 60064, United States; ⊥ WuXi AppTec, 168 Nanhai Road, Tianjin Economic-Technological Development Area TEDA, Tianjin 300457, China

## Abstract

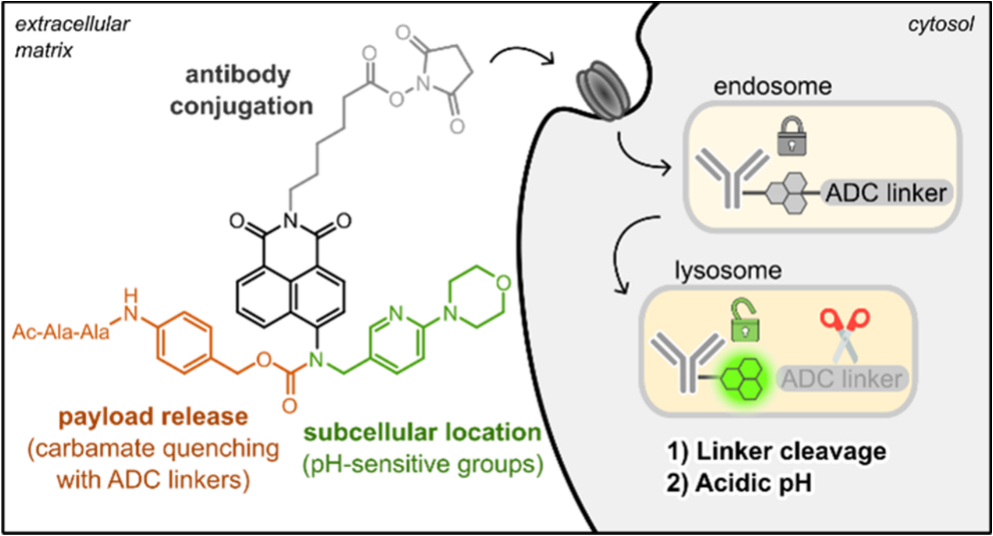

Antibody–drug conjugates (ADCs) represent promising therapeutic constructs to enhance the selective delivery of drugs to target cells; however, attaining precise control over the timing and location of payload release remains challenging due to the complex intracellular processes that define ADC internalization, trafficking, and linker cleavage. In this study, we present novel real-time fluorogenic probes to monitor both subcellular dynamics of ADC trafficking and payload release. We optimized a tandem molecular design of sequential pH- and enzyme-activatable naphthalimide fluorophores to (1) track their subcellular localization along the endolysosomal pathway and (2) monitor linker cleavage with OFF-to-ON fluorescence switches. Live-cell imaging microscopy revealed that fluorogenic ADCs can traffic to the lysosomes and yet require residence time in these subcellular compartments for efficient linker cleavage. Notably, the compact size of fluorogenic naphthalimides did not impair the recognition of target cell surface reporters or the kinetics of payload release. This modular platform is applicable to many ADCs and holds promise to inform their rational design for optimal release profiles and therapeutic efficacy.

## Introduction

Antibody–drug conjugates (ADCs) combine drugs and antibodies binding surface receptors to enhance the cell-specific delivery of therapeutic payloads.^1,2^ Many FDA-approved ADCs contain cleavable linkers that respond to intracellular triggers (e.g., lysosomal acidic pH,^3,4^ enzymatic targets like cathepsins)^5,6^ to release drugs only inside target cells; however, monitoring where and when these events take place in live cells remains challenging. Recent studies have reported evidence that linker cleavage in ADCs can occur early in the endolysosomal pathway before reaching lysosomal compartments.^7,8^ Other studies report significant variability in release rates, largely due to variations in the expression levels of cathepsins and other proteases.^9^ The design of general imaging platforms for real-time tracking of payload release from ADCs in live cells and at the subcellular level has the potential to accelerate the rational design of ADCs with enhanced therapeutic efficacy.

Several approaches have been described to monitor trafficking and payload release in ADCs. Isotope labeling enables dual visualization of antibody (e.g., ^89^Zr and ^124^I) and payload (e.g., ^3^H and ^14^C) at the macroscopic level (e.g., in vivo via PET or SPECT imaging), but with limited cellular resolution.^10,11^ Similarly, molecular approaches involving the genetic engineering of cell lines (e.g., luciferase-expressing cells) can report on payload release using in-bulk functional assays.^12^ Additionally, mass spectrometry provides further molecular information (e.g., conjugation sites, drug-to-antibody ratios) as well as quantification of the rates of released versus intact ADCs in cells and biosamples.^13^ To date, mass spectrometry is restricted in spatiotemporal resolution and cannot directly report in situ kinetics and subcellular localization of ADCs.

Fluorescence imaging enables real-time, noninvasive visualization of discrete events in live cells with exceptional spatial and temporal resolution.^14^ Recent advances in the chemical design of fluorophores have rendered activatable probes with labile groups that temporarily mask fluorescence readouts and release them only under defined microenvironments (e.g., variable pH, enzymatic activity).^15−18^ For example, our group and others have described pH-sensitive groups^19^ and electron-withdrawing carbamate cages^20^ to fine-tune fluorescence emission on the basis of biological activity. Fewer examples of activatable fluorophores have been described as mechanistic probes for ADCs.^21^ For instance, fluorescently labeled antibodies with pH-sensitive dyes or endocytosis markers can track the location of ADCs,^22−24^ but are unable to monitor linker cleavage and payload release. In this work, we present a new class of tandem pH- and enzyme-activatable probes for simultaneous imaging of the subcellular localization and payload release of ADCs in live cells and in real time.

**Figure 1:**
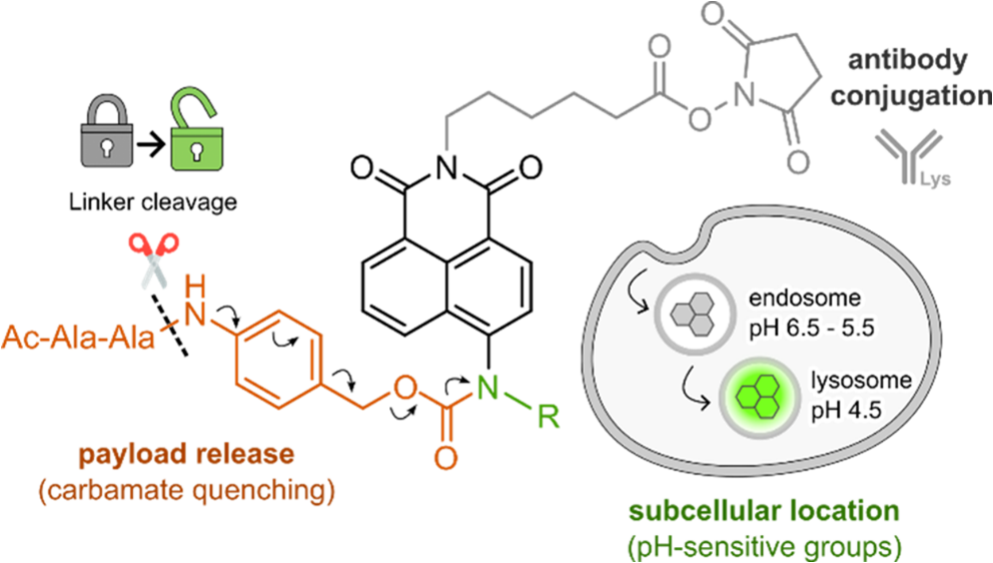
Chemical design of fluorogenic probes for ADC imaging. The naphthalimide scaffold was modified with three orthogonal moieties: pH-sensitive amine groups to modulate fluorescence emission (green), cleavable linkers acting as switches for payload release (orange), and a succinimidyl ester group for direct conjugation to Lys residues in antibodies (gray).

To achieve this goal, we designed naphthalimide-based fluorogens that would allow us to study two key steps linked to the processing and efficacy of ADCs, namely lysosomal localization and proteolytic cleavage of linkers for drug release (Figure 1). Although several naphthalimide probes have been reported for live-cell imaging,^25−27^ to date they have not been utilized as mechanistic tools for the visualization of ADCs. In our chemical design, we modified the naphthalimide scaffold to accommodate (1) pH-dependent emission (to turn-on or turn-off in acidic lysosomes), (2) intramolecular quenching of carbamate groups to report linker cleavage and payload release, and (3) direct conjugation to Lys residues in antibodies through amide bond formation (Figure 1). The combination of all three structural elements in a single fluorescent scaffold has rendered some of the first fluorogenic probes for real-time subcellular imaging of ADCs in live cells.

## Results and Discussion

### Synthesis and Characterization of Naphthalimide pH-Dependent Fluorophores

Naphthalimides have been reported as fluorescent probes for numerous applications, including the detection of metal ions, reactive oxygen species, and enzymatic activity, among others.^28^ The intracellular trafficking of ADCs encounters progressively acidified microenvironments, from early endosomes (pH ∼ 6.5) to late endosomes (pH ∼ 5.5) and lysosomes (pH ∼ 4.5); therefore, we decided to synthesize new naphthalimides to detect pH variations across the entire endolysosomal pathway (pH 4.0–7.4). For this purpose, we designed a combinatorial library of 26 naphthalimide compounds with amine moieties selected to cover chemical diversity in electron-donating and withdrawing groups, aiming to modulate p*K*_a_ values and fluorescence emission at different pHs.

**Figure 2:**
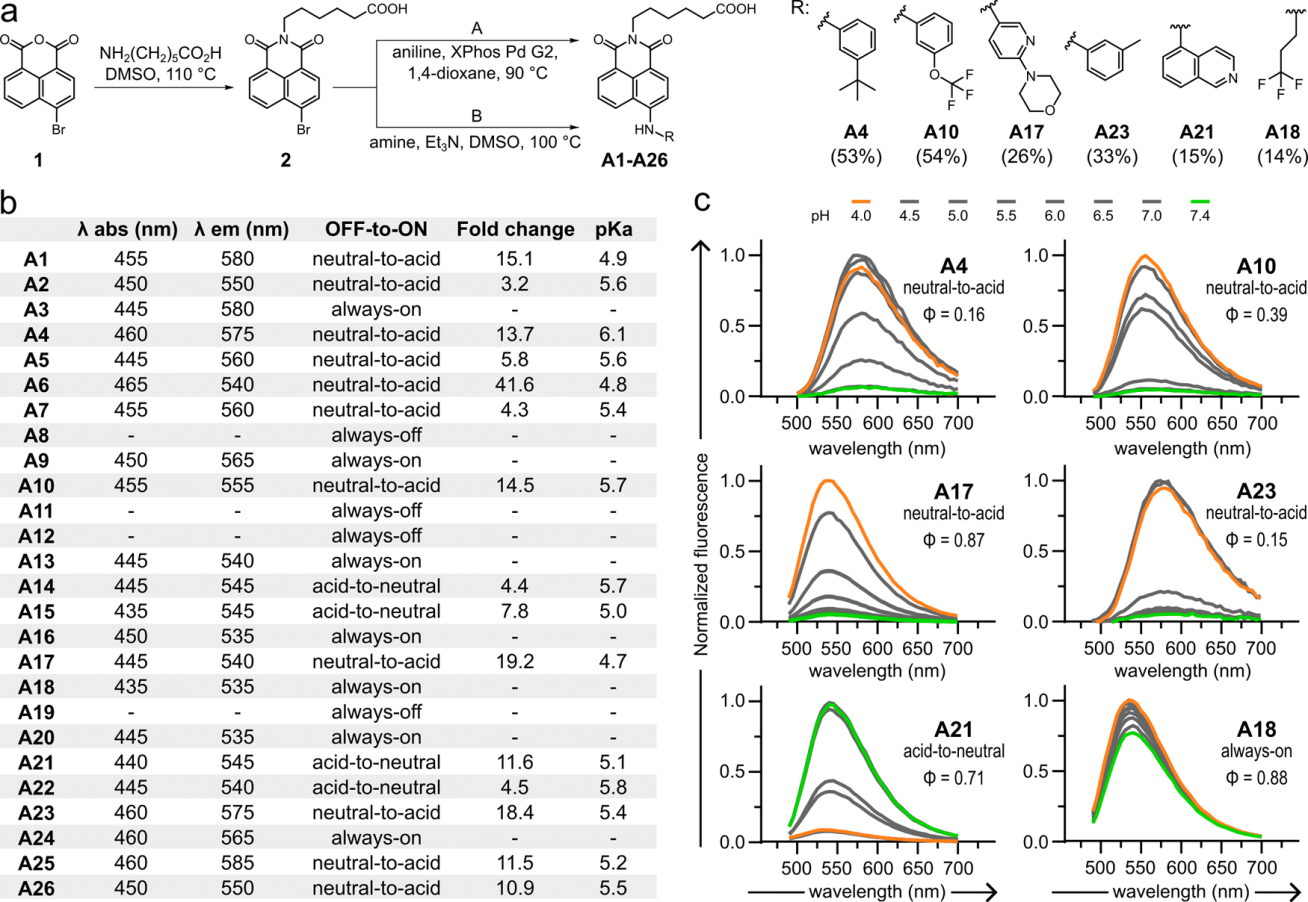
Synthesis and characterization of a library of pH-sensitive naphthalimides. (a) General procedure for the synthesis of compounds **A1-A26**, including structures and synthetic yields of selected compounds **A4, A10, A17, A18, A21**, and **A23**. (b) Optical properties of compounds **A1-A26**, including excitation/emission maxima wavelengths, fluorescence fold increase ratios between pH 7.4 and pH 4.0, and p*K*_a_ values. (c) Normalized fluorescence emission spectra of the 6 selected compounds (10 μM) in buffers ranging from pH 4.0 to pH 7.4 (λ_exc_: 450 nm). Relative fluorescence quantum yields were calculated at pH 4.0 for neutral-to-acid compounds (**A4, A10, A17**, and **A23**) and at pH 7.4 for acid-to-neutral (**A21**) and always-on (**A18**) compounds.

Starting from the commercially available 4-bromo-1,8-naphthalic anhydride (**1**, Figure 2a), we first prepared the conjugatable naphthalimide precursor (**2**, Figure 2a) in gram scale by reacting compound **1** with 6-aminohexanoic acid. Next, we used compound **2** to perform parallel synthesis of amine-substituted naphthalimides. For aniline moieties, we performed Pd-catalyzed Buchwald couplings. For other amine substituents, we reacted compound **2** with the different amines under heating in basic conditions. The final 26 naphthalimide fluorophores (**A1**-**A26**, Figure S1) were purified by reverse-phase preparative HPLC and isolated with purities ≥90% with yields ranging from 12% to 53% (chemical structures, synthetic details and full characterization data of the entire library are included in the Supporting Information).

Next, we acquired the absorbance and emission spectra of the entire library at different pHs within the 4.0–7.4 range (Figures S2 and S3). As shown in Figure 2b, we observed a relatively broad range of fluorescence signatures within the library, confirming that amine diversification represents an efficient strategy to fine-tune the pH dependence of the naphthalimide core.^29^ Interestingly, the characterization of the library revealed 4 types of pH sensitivity: (1) always-on fluorophores with pH-independent emission (compounds **A3**, **A9**, **A13**, **A16**, **A18**, **A20** and **A24**), (2) always-off fluorophores with quenched emission (compounds **A8**, **A11**, **A12** and **A19**), (3) neutral-to-acid fluorophores with low emission at neutral pH and activation in acidic conditions (compounds **A1**, **A2**, **A4**, **A5**, **A6**, **A7**, **A10**, **A17**, **A23**, **A25** and **A26**), and (4) acid-to-neutral fluorophores with activation at neutral pH and quenched emission in acidic environments (compounds **A14**, **A15**, **A21** and **A22**).

The analysis of structure-spectroscopy relationships highlighted that neutral-to-acid activation was predominantly observed for aniline-substituted fluorophores (9 out of 11 naphthalimides). However, some aniline groups with electron-donating moieties in para position prevented fluorescence activation even at acidic pHs, resulting in always-off responses (e.g., compounds **A8** and **A12**). In contrast, most aliphatic amines, with the exception of compounds **A14**, **A21** and **A22**, exhibited lower pH-dependence, accounting for 4 out of 7 always-on fluorophores. From this spectroscopic analysis, we selected and scaled-up 6 naphthalimide fluorophores (compounds **A4**, **A10**, **A17**, **A18**, **A21**, and **A23**), including 4 neutral-to-acid fluorophores (compounds **A4**, **A10**, **A17**, and **A23**) with large fluorescence fold increases (13.7, 14.5, 19.2, and 18.4, respectively) and different p*K*_a_ values (6.1, 5.7, 4.7, and 5.4, respectively). Our selection also included compound **A21** as an acid-to-neutral fluorophore (fold increase: 11.6, p*K*_a_: 5.1) and compound **A18** as an always-on fluorophore, as complementary molecules for imaging studies. Prior to moving on to cellular studies, we confirmed that the fluorescence emission spectra of the 6 selected naphthalimides were not affected by other physiologically relevant metabolites, such as glutathione or reactive oxygen species (Figure S4).

### Antibody Conjugation and Imaging of Activatable Fluorescent Conjugates in Real Time

Having selected a subset of 6 naphthalimide fluorophores, we conjugated them to the murine antibody 8C11, which targets the murine tumor necrosis factor α (mTNFα).^30^ TNFα is a pro-inflammatory cytokine and a relevant therapeutic target in both oncology and inflammatory diseases.^31^ Furthermore, the binding of 8C11 to mTNFα triggers endocytosis-based internalization of the antibody,^32^ thus being an optimal model to study the intracellular trafficking of ADCs.^30^ We prepared the 8C11-naphthalimide conjugates by direct conjugation of the fluorophores to Lys residues of the antibody. Activation of the carboxylic acid groups into the corresponding succinimidyl esters followed by amide formation in slightly basic buffer (0.5 M borate buffer, pH 8.0) and ultracentrifugation purification rendered the 8C11 fluorescent antibodies (**8C11_A4, 8C11_A10, 8C11_A17, 8C11_A18, 8C11_A21** and **8C11_A23**) with fluorophore-antibody ratios (FAR) between 0.6 and 3.0. Importantly, we performed spectral characterization of all 6 conjugates and confirmed that the pH responses of most naphthalimide fluorophores (i.e., **A10, A17, A18** and **A21**) were retained after antibody conjugation (Figure S5).

Next, we evaluated the cellular internalization of the 8C11 fluorescent antibodies by confocal microscopy in live HEK293 cells transfected with mTNFα and compared the results to wild-type, nonantigen expressing cells. With the exception of **8C11_A4** (FAR: 1.0) and **8C11_A18** (FAR: 0.6), which could not distinguish between transfected and nontransfected cells, the incubation of HEK293 cells with fluorescent 8C11 conjugates rendered substantially brighter intracellular signals in mTNFα-transfected cells than in nontransfected cells (Figure S6), further confirming the TNFα receptor-mediated internalization of 8C11 conjugates. Among all 8C11 fluorescent antibodies, **8C11_A17** (neutral-to-acid, p*K*_a_: 4.4) and **8C11_A21** (acid-to-neutral, p*K*_a_: 5.1) displayed the best imaging profiles, and were selected for further studies.

**Figure 3:**
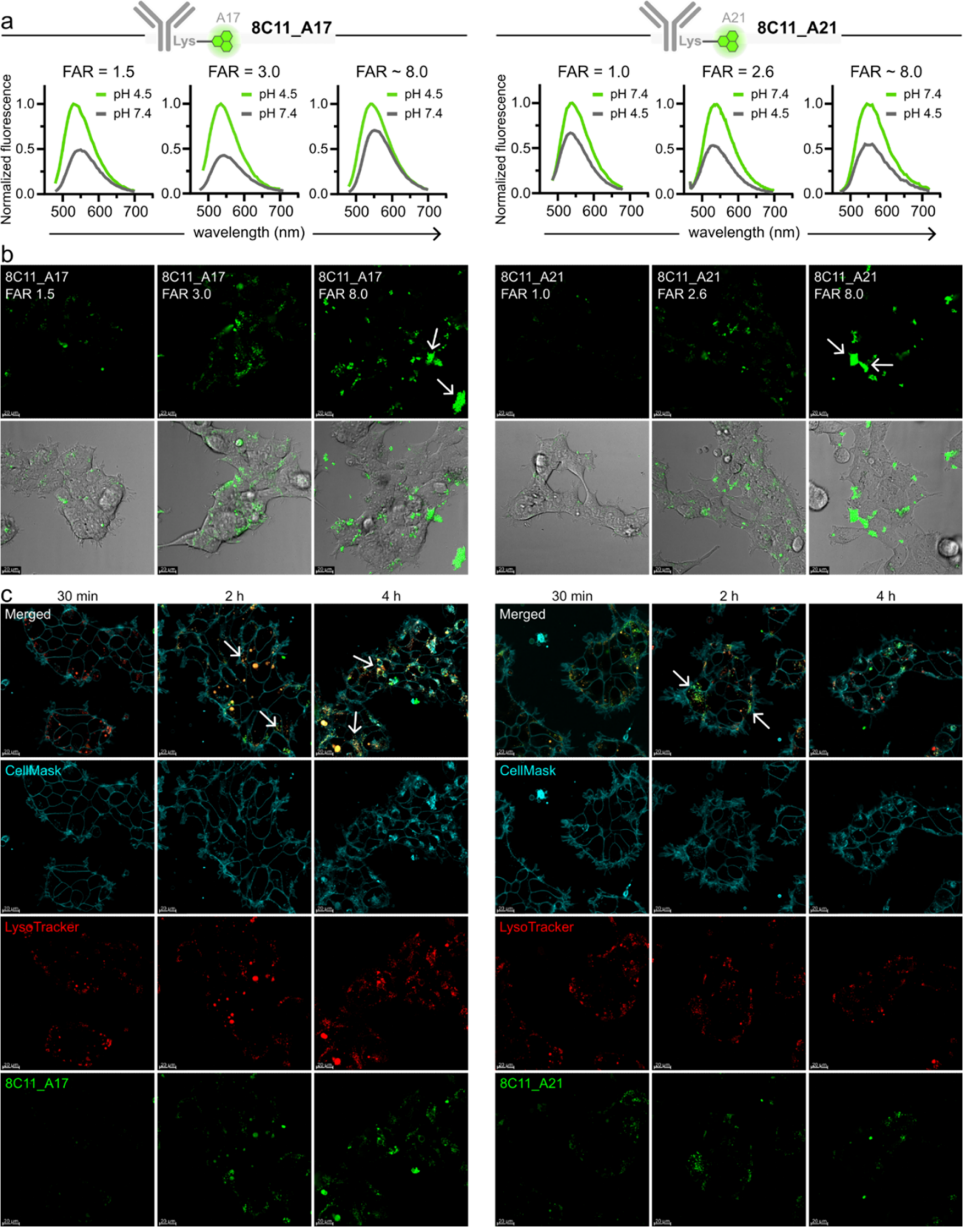
Fluorescent conjugates enable real-time subcellular imaging of antibody localization. (a) Normalized fluorescence emission spectra of the conjugates **8C11_A17** and **8C11_A21** (both at 200 nM) at pH 4.5 and pH 7.4 (λ_exc_: 450 nm), and with varying degrees of labeling (FARs: ∼1.5, ∼3.0, and ∼8.0). For FAR calculations, the extinction coefficient of 8C11 was determined as 210,000 M^–1^ cm^–1^. (b) Representative fluorescence microscopy images of HEK293 cells transfected with mTNFα and incubated with **8C11_A17** (200 nM, left) and **8C11_A21** (200 nM, right) with different FARs after incubation for 2 h (exc/em: 450/550 nm). The presence of aggregates is highlighted by white arrows. The bottom row includes overlay images of fluorescence and brightfield. (c) Representative fluorescence microscopy images of live HEK293 cells transfected with mTNFα and treated with **8C11_A17** (200 nM, left) or **8C11_A21** (200 nM, right) at different time points (t: 30 min, 2 h, and 4 h). Subcellular activation of the fluorophores (**A17** and **A21**) is highlighted by white arrows. Cells were costained with LysoTracker Red (50 nM, exc/em: 573/593 nm, red) and CellMask Deep Red (500 nM, exc/em: 660/675 nm, cyan). The bottom rows of the merged images include the single-channel fluorescence images of 8C11-dye conjugates, LysoTracker Red, and CellMask Deep Red. Scale bars: 20 μm.

Next, we decided to optimize the conjugation of the **8C11_A17** and **8C11_A21** by systematic derivatization at different FAR values, including low (∼1.5), intermediate (∼3.0) and high (∼8.0) ratios. After preparing 6 derivatives (2 conjugates at 3 different FARs), we examined them under confocal microscopy in mTNFα-transfected HEK293 cells (Figure 3a). The two conjugates with FAR around 1.0–1.5 resulted in weak fluorescence readouts, while conjugates with FAR ∼ 8.0 led to visible extracellular antibody aggregates (Figures 3b and S7). Based on these observations, we chose conjugates with intermediate FARs to run time-course imaging experiments for both **8C11_A17** (FAR: 3.0) and **8C11_A21** (FAR: 2.6).

We performed real-time imaging experiments in mTNFα-transfected HEK293 cells upon incubation at 37 °C with the fluorescent antibodies (200 nM), alongside the lysosomal marker Lysotracker Red and the cell membrane marker CellMask Deep Red. For the fluorescent antibody **8C11_A17**, the fluorescence signals were only detectable after 2 h. At 4-h incubation time points, the fluorescence signals of **8C11_A17** were brightest and colocalized with LysoTracker Red (Figures S8 and S9). Given that the naphthalimide **A17** is a neutral-to-acid pH-sensitive fluorophore, these findings indicate that **8C11_A17** reached the late compartments of the endolysosomal pathway at 2 h, and that the increase in emission at 4 h was the result of cumulative trafficking to the lysosomes. In sharp contrast, the **8C11_A21** conjugate exhibited good fluorescent signals after 30 min, reaching maximal intensity at 2 h but with poor colocalization with LysoTracker Red (Figure S8). Because the acid-to-neutral naphthalimide **A21** is brighter at neutral pH, this observation is in agreement with fluorescence emission being observed at prelysosomal stages of the endolysosomal pathway (Figure 3c). Furthermore, the lower emission intensity of **A21** in acidic microenvironments explains the decrease in fluorescence intensity as the antibody traffics to the lysosomes. Altogether, these results demonstrate that the conjugation of the pH-sensitive naphthalimides **A17** and **A21** enabled real-time subcellular monitoring of the localization of 8C11 in live cells. Their complementary pH sensitivity profiles (**A17**: bright at low pH, **A21**: bright at neutral pH) provides detailed information on the endolysosomal trafficking of antibodies, underscoring their utility as functional fluorophores for imaging the intracellular dynamics of ADCs.

### Incorporation of Intramolecular Quenchers Enables In Situ Monitoring of Linker Cleavage

Having identified pH-sensitive naphthalimides as efficient indicators of ADC subcellular localization, next we chemically modified them to incorporate additional reporters of linker cleavage to monitor payload release. For this purpose, we selected carbamate cages^20^ because they are (1) essential structural elements of cleavable linkers in ADCs,^33^ and (2) utilized to introduce enzyme-activatable quenching groups via self-immolative linkers.^34^ We envisaged that the incorporation of carbamates at the position 4 of the naphthalimide core would alter its push–pull dipole and function as a molecular switch to turn on the emission of the fluorophores only after cleavage of the ADC linker.

**Figure 4:**
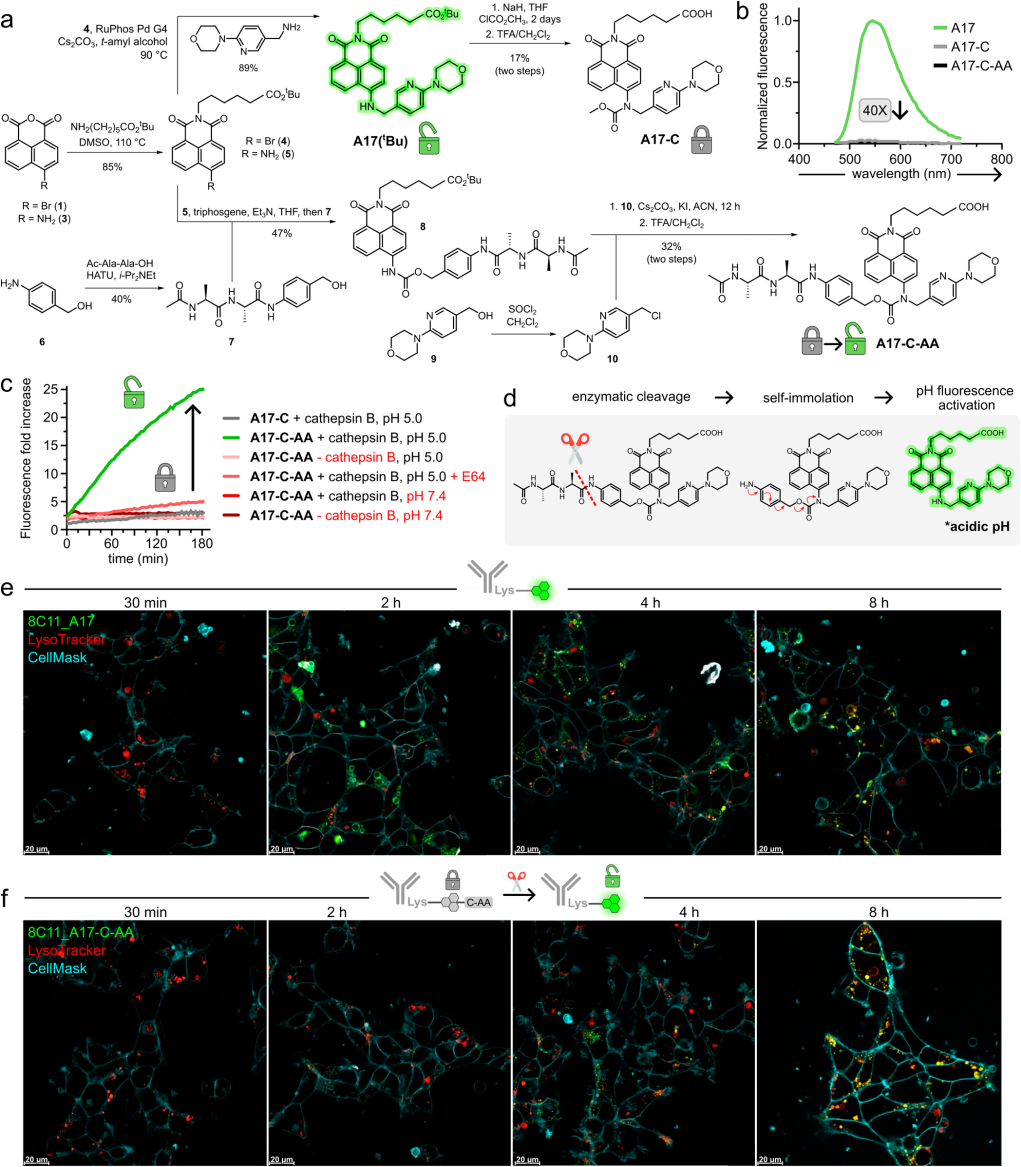
Design and synthesis of intramolecular quenchers to enable in situ monitoring of ADC linker cleavage. (a) Synthetic scheme for compounds **A17-C** and **A17-C-AA**. (b) Normalized fluorescence emission spectra of compounds **A17**, **A17-C**, and **A17-C-AA** at pH 4.0 (λ_exc_: 450 nm). The fold decrease in maximal emission intensity between **A17** and **A17-C** is highlighted in gray. (c) In vitro lysosomal payload release assays. **A17-C** or **A17-C-AA** (40 μM) were incubated with or without cathepsin B (40 nM) at pH 5.0 or 7.4, and with or without the cathepsin B inhibitor E64 (2 μM). Fluorescence fold increases were determined by referring to the caged compound **A17-C**. (d) Proposed mechanism for the tandem fluorescence activation of the dual-activated fluorophore **8C11_A17-C-AA**. (e, f) Representative fluorescence microscopy images of live HEK293 cells transfected with mTNFα and treated with 200 nM **8C11_ A17** (e) or **8C11_A17-C-AA** (f) (exc/em: 450/550) at different time points (t: 30 min, 2, 4, and 8 h). Cells were costained with LysoTracker Red (exc/em: 573/593 nm, red) and CellMask Deep Red (exc/em: 660/675 nm, cyan). Figure S13 contains the single-channel fluorescence images of 8C11-dye conjugates, LysoTracker Red and CellMask Deep Red. Scale bars: 20 μm.

First, we synthesized compound **A17-C** (Figure 4a) as a model derivative of compound **A17** including a methyl carbamate group. The *tert*-butyl ester derivative of **A17** (**A17(**^**t**^**Bu)**) was reacted with methyl chloroformate and then subjected to acid hydrolysis of the ester moiety to render compound **A17-C** (full synthetic and characterization details in the Supporting Information). As expected, the introduction of the carbamate group at the position 4 of the naphthalimide fluorophore drastically reduced its fluorescence emission (i.e., ∼ 40-fold reduction, Figures 4b and S10), confirming its suitability to incorporate enzyme-activatable quenchers. Notably, we demonstrated that the same strategy was applicable to the fluorophore **A21** by preparing the carbamate-quenched compound **A21-C** (Figure S11). These results prove that this quenching strategy is independent of the amine moiety appended to the naphthalimide fluorophore.

Encouraged by the quenching efficacy of compound **A17-C**, we synthesized the compound **A17-C-AA** (Figure 4a), whereby the carbamate quenching group included the cleavable dipeptide Ala-Ala-PABC (*p*-aminobenzyloxy-carbonyl) linker. Dipeptide linkers are widely used in ADCs and react with lysosomal proteases to release drug payloads at late endolysosomal stages.^8^ In particular, Ala-Ala linkers demonstrated compatibility with 8C11 by enabling stable attachment to both the antibody and payload while affording ADCs with low levels of aggregation.^35,36^

The synthesis of the caged compound **A17-C-AA** proved challenging. Unlike compound **A17-C**, the introduction of a peptidic self-immolative carbamate linker onto the **A17** structure failed to render the expected compound, likely due to steric hindrance and poor nucleophilicity of the aniline moiety. Therefore, we first prepared compound **8** by coupling the unsubstituted naphthalimide **5** to the Ala-Ala-PABC linker **7**, followed by alkylation of the carbamate with **10**, i.e., as the chlorinated derivative of the pH-sensitive amine **9** (Figure 4a). This synthetic approach not only afforded compound **A17-C-AA** in good purity and reasonable scale (i.e., >200 mg) but also represents a versatile strategy to prepare caged carbamate prodrugs and profluorophores for scaffolds featuring bulky or unreactive groups (e.g., anilines).

Having synthesized compound **A17-C-AA**, we analyzed its optical properties and confirmed that it retained the strong fluorescence quenching effect initially observed in **A17-C** (Figure 4b). Next, we investigated the fluorescence activation of compound **A17-C-AA** in vitro using the protease cathepsin B, which is found in lysosomal environments during ADC linker cleavage and payload release. Under these conditions, the compound **A17-C-AA** exhibited significant fluorescence intensity only after dual activation (i.e., cathepsin activity and acidic pH). Control experiments without cathepsin B or in the presence of the cathepsin B inhibitor E64 confirmed that enzyme-mediated linker cleavage was necessary for fluorescence detection (Figure 4c). Similarly, control experiments at pH 7.4 confirmed that acidic conditions were also required for fluorescence emission. Collectively, these findings indicate that **A17-C-AA** is a suitable probe to study the trafficking and payload release of ADCs, with a tandem-activated fluorophore that emits exclusively in acidic environments with high proteolytic activity (Figure 4d).

Finally, we conjugated the activatable fluorophore **A17-C-AA** to the antibody 8C11 (FAR: 3.0). The optical properties of **8C11_A17-C-AA** were similar to those of compound **A17-C-AA** (Figure S12). We performed live-cell imaging in HEK293 cells transfected with mTNFα to monitor the fluorescence activation of **8C11_A17-C-AA**, which would be indicative of dipeptide cleavage and lysosomal localization (Figures 4e,f, and S13). In these experiments, the labeled antibody **8C11_A17-C-AA** displayed fluorescence emission only after 4 h with bright intracellular signals in the lysosomes (by colocalization with LysoTracker Red) after 8 h. Furthermore, cotreatment with the cathepsin inhibitor E64 prevented the fluorescence activation of **8C11_A17-C-AA** (Figure S14). In contrast, the antibody **8C11_A17**, which did not contain the cleavable linker, was emissive in lysosomes after 2 h. These findings indicate that lysosomal localization on its own does not entirely correspond to payload release, and that **8C11_A17-C-AA** required some residence time in acidic compartments to observe effective linker cleavage. Furthermore, we found that a fraction of the antibody **8C11_A17-C-AA** emitted fluorescence signals at 4 h in subcellular regions not colocalizing with lysosomes, suggesting that payload release could partially occur outside lysosomes. Overall, these data reveal important insights into the spatiotemporal dynamics of payload release, underscoring the potential application of **A17-C-AA** as a dual-activatable fluorophore to monitor release mechanisms of ADCs in different subcellular environments.

### Dual Monitoring of Fluorescence Activation and Payload Release in ADCs

**Figure 5:**
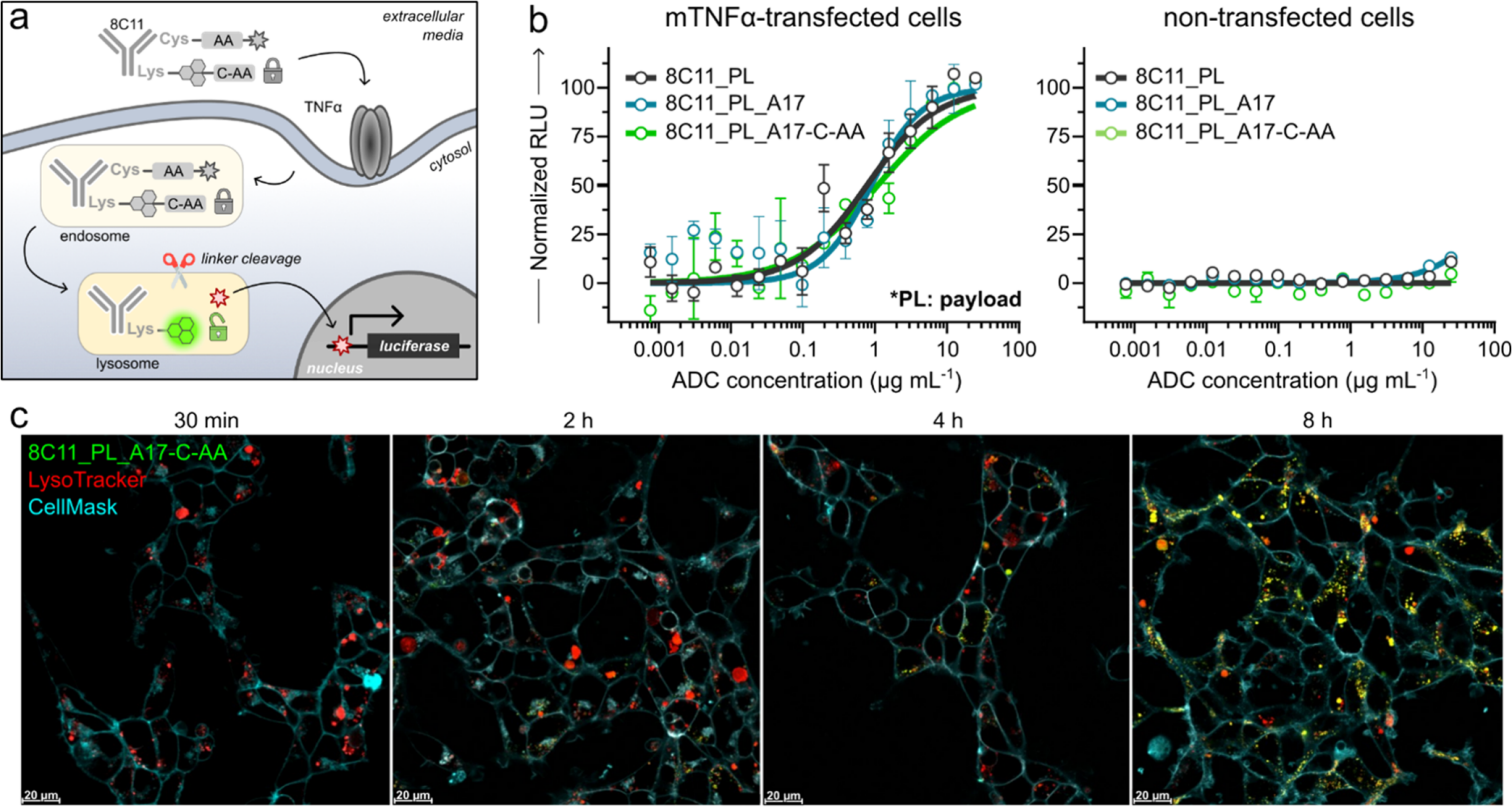
Tracking linker cleavage and payload release in dual-conjugated ADCs. (a) Schematic representation of the mechanism of action of dual-conjugated anti-TNFα antibody (8C11) bearing both the payload GRM-103 (DAR: 4.0, represented by a gray star (inactive payload) or red star (active payload)) and the fluorophore (**A17-C-AA**, DAR: 3.0) attached via Ala-Ala linkers. (b) Luminescence-based cell reporter assay for measuring payload release. K562 cells, either transfected with a GRE reporter gene alone or cotransfected with mTNFα, were treated with serially diluted concentrations of ADCs (starting from 25 μg mL^–1^) for 72 h. Payload release was quantified by measuring firefly luciferase activity (RLU) after cell lysis. PBS and dexamethasone (100 nM) were used as negative and positive controls, respectively. Values presented as means and error bars as SEM (*n* = 3). (c) Representative fluorescence microscopy images of live HEK293 cells transfected with mTNFα treated with 200 nM **8C11_PL_A17-C-AA** (exc/em: 450/550) at different time points (t: 30 min, 2, 4, and 8 h). Cells were costained with LysoTracker Red (exc/em: 573/593 nm, red) and CellMask Deep Red (exc/em: 660/675 nm, cyan). Scale bars: 20 μm.

Next, we examined the application of incorporating our fluorogenic platform in an ADC that had a glucocorticoid receptor modulator (GRM) payload conjugated to Cys residues through a protease-activatable linker. By using separate linkers containing the short Ala-Ala sequence in both the fluorogen **A17-C-AA** and the GRM payload, we envisioned that the fluorescence activation of compound **A17** could be a suitable indicator of protease activity and payload release (Figure 5a). We performed these experiments with the ADC **8C11_PL**, which contained the mTNFα-targeting 8C11 antibody and GRM-103 as a linker-payload (Figure S15)^36^ with a drug-antibody ratio (DAR) of 4.0. We labeled the Lys residues of **8C11_PL** with the uncaged fluorophore **A17** and the caged **A17-C-AA** using the previously optimized protocols. Both fluorogenic ADCs (i.e., **8C11_PL_A17** and **8C11_PL_A17-C-AA**, respectively) were prepared with a FAR of 3.0.

In order to determine if fluorophore conjugation had altered the kinetics of payload release, we compared the efficacy of all ADCs in K562 GRE reporter cells that had been transfected with mTNFα and in wild-type K562 GRE reporter cells that did not express mTNFα. The K562 GRE reporter cell line was genetically engineered to trigger luciferase expression upon GRM payload release, which could be readily quantified by in vitro luminescence assays (Figure 5a). Notably, we observed that the nonfluorescent **8C11_PL** showed an EC_50_ of 0.8 ± 0.2 μg mL^–1^, similar to those of the fluorescent **8C11_PL_A17** (EC_50_ = 0.9 ± 0.4 μg mL^–1^) and **8C11_PL_A17-C-AA** (EC_50_ = 1.1 ± 0.5 μg mL^–1^) (Figure 5b). Furthermore, none of the ADCs showed drug release in wild-type K562 cells lacking mTNFα expression, confirming that payload release was dependent on mTNFα receptor-mediated internalization of the ADC. These results identify naphthalimides as compact fluorescent labels for ADC modification that do not impair the capacity of the antibody for receptor-mediated endocytosis or the targeted release of payloads in live cells.

Finally, we performed live-cell imaging studies to examine the dynamics of cellular localization and linker cleavage of **8C11_PL_A17** and **8C11_PL_A17-C-AA**. Fluorescence microscopy revealed localization patterns in agreement with those observed for single-conjugated probes (**8C11_A17** and **8C11_A17-C-AA**; Figures 5c and S16), which confirmed that the addition of the GRM-103 payload did not affect the mechanisms of fluorescence activation. The pH-activatable ADC **8C11_PL_A17** showed localization in lysosomes after 2 h (Figure S9), while the pH- and enzyme-activatable ADC **8C11_PL_A17-C-AA** required an extended lysosomal residence time, with fluorescence activation only becoming evident after 4 h (Figure 5c). After 8 h, bright fluorescence intensity and strong colocalization with LysoTracker Red highlighted the lysosomal accumulation and effective linker cleavage (Figure 5c).

These findings confirm that the tandem activation mechanism of the pH- and enzyme-activatable fluorophore **A17-C-AA** is a reliable approach for simultaneous live-cell tracking of subcellular location and linker cleavage in fully functional ADCs. The respective nonfluorescent and fluorogenic **8C11_PL** and **8C11_PL_A17-C-AA** showed comparable drug release rates in functional reporter assays (Figure 5b), while the drug-free **8C11_A17-C-AA** and drug-conjugated **8C11_PL_A17-C-AA** antibodies demonstrated similar subcellular localization and cleavage kinetics (Figures 4f and 5c). Given that the GRM-103 payload and the **A17** fluorophore were conjugated to the 8C11 antibody through Ala-Ala cleavable units, the fluorescence signals from **8C11_PL_A17-C-AA** can be used as indicators of proteolytic linker cleavage from ADC constructs.

## Conclusions

In summary, we have designed a tandem activatable imaging platform to simultaneously monitor both subcellular trafficking and payload release in ADCs. First, we synthesized a library of 26 naphthalimide fluorophores with diverse pH sensitivity profiles. Several aniline-functionalized compounds showed substantial fluorescence enhancements (i.e., >10-fold increase) at pHs across the entire endolysosomal pathway and proved useful to image the real-time subcellular localization of a mTNFα-targeting antibody upon receptor-mediated endocytosis. Furthermore, we adapted pH-dependent naphthalimides to monitor enzymatic activation by introducing OFF-to-ON carbamate switches that respond to linker cleavage. For this purpose, we optimized a synthetic route where the unsubstituted naphthalimide core was first coupled to the Ala-Ala-PABC linker, followed by alkylation of the carbamate with a pH-sensitive moiety. This approach may enable the synthesis of less accessible carbamate-containing prodrugs and profluorophores featuring bulky or unreactive groups. Optimal FARs were around 3, at which the labeling of ADCs with fluorogenic naphthalimides did not impair targeted recognition of cell surface reporters or the kinetics of payload release. Finally, we performed live-cell imaging microscopy in TNFα-expressing cells, which provided insights into the residence time of labeled ADCs in lysosomal compartments for efficient payload release. The versatility of this platform for imaging ADCs with variable molecular components (e.g., antibodies, drugs, linkers) will accelerate their rational design for optimal therapeutic efficacy.

## Supporting Information Available

Full synthetic and chemical characterization details; NMR spectra; HRMS; HPLC traces; experimental procedures; chemical structures of all compounds of the naphthalimide library (Figure S1); representative absorbance spectra of naphthalimides at different pH (Figure S2); representative fluorescence spectra of naphthalimides at different pH (Figure S3); representative fluorescence spectra of naphthalimides with different metabolites (Figure S4); representative fluorescence spectra of 8C11-fluorophore conjugates (Figure S5); representative confocal microscopy images of 8C11-fluorophore conjugates in transfected and nontransfected HEK293 cells (Figure S6); *Z*-stack imaging of **8C11_A17** (FAR: 8.0) and **8C11_A21** (FAR: 8.0) (Figure S7); quantification of mean cell fluorescence of **8C11_A17** and **8C11_A21** over time (Figure S8); correlation coefficients of colocalization with LysoTracker Red (Figure S9); representative absorbance and fluorescence spectra of compound **A17-C** (Figure S10); representative absorbance and fluorescence spectra of compound **A21-C** (Figure S11); representative fluorescence spectra of compound **8C11_A17-C-AA** (Figure S12); single-channel fluorescence images of **8C11_A17** and **8C11_A17-C-AA** over time (Figure S13)**;** enzymatic inhibition of cathepsin B in cells treated with **8C11_A17-C-AA** (Figure S14); chemical structure of GRM-103 (Figure S15); representative confocal microscopy images of **8C11_PL_A17** and **8C11_PL** in transfected HEK293 cells (Figure S16) (PDF)
