# Supplementary material for: Fluorogenic Platform for Real-Time Imaging of Subcellular Payload Release in Antibody–Drug Conjugates
Source: J Am Chem Soc. 2025 Feb 18;147(9):7578–87. doi: 10.1021/jacs.4c16842 (PMC11887046; doi:10.1021/jacs.4c16842)
Supplement: Supplementary file 1 — ja4c16842_si_001.pdf [file ja4c16842_si_001.pdf]

Electronic Supporting Information

A fluorogenic platform for real-time imaging of subcellular payload release in antibody-drug conjugates

Ferran Nadal-Bufi,[1,2] Paulin L. Salomon,[3] Fabio de Moliner,[1,2] Kathy A. Sarris,[4] Zhi Wang,[4] Rachel D. Wills,[4] Violeta L. Marin,[4] Xiaona Shi,[5] Kuo Zhou,[5] Zhongyuan Wang,[5] Zhou Xu,[5] Michael J. McPherson,[3] Christopher C. Marvin,[4] Adrian D. Hobson,[3] Marc Vendrell[1,2,*]

[1] Centre for Inflammation Research, The University of Edinburgh, Edinburgh EH16 4UU, UK.

[2] IRR Chemistry Hub, Institute for Regeneration and Repair, The University of Edinburgh, Edinburgh EH16 4UU, UK.

[3] AbbVie Bioresearch Center, 381 Plantation Street, Worcester, Massachusetts 01605, USA.

[4] AbbVie Inc., 1 North Waukegan Road, North Chicago, Illinois 60064, USA.

[5] WuXi AppTec, 168 Nanhai Road, Tianjin Economic-Technological Development Area TEDA, Tianjin 300457, China.

* Corresponding author's e-mail: marc.vendrell@ed.ac.uk.

Table of Contents

1. Experimental Details
2. Chemical Synthesis
3. Supplementary Figures
4. NMR Spectra
5. High-Resolution Mass Spectra
6. HPLC Traces
7. Supplementary References

1. Experimental Details

Materials and methods. Final compounds were purified to ≥90% purity by analytical high performance liquid chromatography (HPLC). Reagents and solvents were purchased from Combi-Blocks, Millipore Sigma, or other vendors as noted, and were used without further purification. Reactions were carried out under an inert atmosphere of nitrogen. Yields refer to isolated yields of analytically pure (>90%) material unless noted otherwise. Compound names were generated with Perkin Elmer ChemDraw 20.1. Whenever possible, reactions were monitored by liquid chromatography-mass spectrometry (LCMS). Magnetic resonance spectra (^{1}H,^{13}C NMR and ^{19}F NMR) were measured with 400, 500, or 600 MHz spectrometers. NMR data were processed with MestreNova software. Chemical shifts are reported in parts per million (δ, ppm). First order splitting patterns were interpreted and coupling constants rounded to the nearest 0.1 Hz. Splitting patterns are designated as: s, singlet; d, doublet; t, triplet; q, quartet; and m, multiplet. ^{1}H NMR spectra are referenced to residual chloroform (7.27 ppm) or residual dimethyl sulfoxide (2.50 ppm); ^{13}C NMR spectra are referenced to the central line of the 1:1:1 triplet of $CDCl_3$ (77.23 ppm) or DMSO-d6 (39.52 ppm). ^{19}F NMR spectra are referenced to trichlorofluoromethane (0 ppm). HRMS (ESI positive) were obtained with a Bruker ESI Micro-TOF mass spectrometer. Spectroscopic data were measured on a Synergy HT spectrophotometer (Biotek) and data analysis was performed using GraphPad Prism 5.0. Cell imaging was performed with a Leica SP8 fluorescence confocal microscope equipped with a live-cell imaging stage. Images were acquired and processed with the corresponding microscope software, Leica Application Suite X (LAS X) V1.4.6.

Photophysical characterization. Absorbance and fluorescence emission spectra were measured using a Synergy HT spectrophotometer (Biotek) in 96-well black/clear bottom polystyrene microplates. Samples (100 µL per well) were prepared in 0.1 M Na_2HPO_4–citric acid buffers, with the pH adjusted between 4.0 and 7.4. Free dyes were measured at a concentration of 10 µM, and 8C11-dye conjugates at 200 nM. Absorbance spectra were recorded from 300–600 nm, and fluorescence emission spectra from 490–700 nm (λ_{ex}: 450 nm), using 5 nm increments. All measurements were performed in triplicate, and the spectra presented in the manuscript are representative of the data. Data analysis was conducted using GraphPad Prism 5.0. Relative fluorescence quantum yields were calculated using fluorescein as a reference (ΦF = 0.91 in 0.1 M NaOH, λ_{ex}: 480 nm).

Dye-antibody conjugations. Initially, the carboxylic acid groups of the fluorophores were activated into the corresponding succinimidyl esters, by reacting them with 1 molar equivalent of *N,N'*-dicyclohexylcarbodiimide and 2 molar equivalents of *N*-hydroxysuccinimide in THF for 24 h at r.t.. Reaction conversions over 90% were confirmed by HPLC. The murine antibody 8C11 (anti-TNFα)[1] or the ADC **8C11_PL** (anti-TNFα with GRM-103 as a payload)[2] (both at 10 mg mL^{-1} in PBS) were spiked in 10% (v/v) in 0.5 M borate buffer (pH 8.0). Afterward, the succinimidyl ester fluorophores were added at 2.0, 4.5 or 10.0 molar equivalents targeting fluorophore-antibody ratios (FARs) of 1.5, 3.0 and 8.0, respectively. The reaction was incubated for 90 min at r.t. with gentle rocking. Following incubation, the conjugates were purified with PBS using Zeba™ Spin Desalting Columns, 7K MWCO, 2 mL (ThermoFisher Cat# 89890), according to manufacturer's instructions. The purified conjugates were filtered through a 0.22 µm filter. To calculate the FARs, the concentration of

fluorophore was obtained by measuring the absorbance at 450 nm and the concentration of protein was obtained by measuring absorbance at 280 nm (MW = 150 kDa, ε = 210,000 M^{-1} cm^{-1}) and subtracting the contribution of the fluorophore at 280 nm. The conjugation reactions for the main constructs (**8C11_A17** and **8C11_A21**) were performed in triplicate yielding standard deviations in FAR within the 10% range.

Cell culture and imaging. HEK293 cells and mTNFα-transfected HEK293 cells were grown in Dulbecco's modified Eagle's medium (DMEM) supplemented with 10% (v/v) fetal bovine serum (FBS), 20 mM L-glutamine, and 1% (v/v) penicillin–streptomycin. HEK293 mTNFα cells were additionally cultured with 0.5 mg mL^{-1} geneticin/neomycin to maintain selection pressure. Cells were resuspended in complete medium, counted using a Countess II FL, plated (10,000 cells $well^{-1}$) in a μ-slide 18-well glass bottom chamber (IBIDI®), and incubated at 37°C with 5% CO_2 for 24 h. Afterward, 10 μL fluorophore-antibody conjugates were added for a final concentration of 200 nM and incubated over time for 30 min, 2 h, 4 h or 8 h. Before imaging, the medium was removed by suction and replaced with 100 μL phenol red-free medium containing LysoTracker™ Red (50 nM) or not, and CellMask™ Deep Red (500 nM) or not. Cells were imaged at 37°C in a Leica SP8 fluorescence confocal microscope (fluorophore-antibody conjugates, exc/em: 450/460-563 nm; LysoTracker™ Red, exc/em: 573/593-645 nm; CellMask™ Deep Red, exc/em: 660/675-715 nm) equipped with a live-cell imaging stage using a HC PL APO CS2 20x/0.75 dry lens. For Z-stack imaging microscopy, 40 images were acquired across a physical depth of ~25 μm. To inhibit cathepsins, cells were co-treated with E64 (10 μM). All images were acquired and processed with the corresponding microscope software, Leica Application Suite X

(LAS X) V1.4.6. All imaging experiments were performed at least in triplicate, with a minimum of three images taken for each condition in each experiment. All images included in the manuscript and Supplementary Information are representative.

In vitro enzymatic assays of lysosomal linker cleavage. The probes **A17-C** or **A17-C-AA** (40 µM) were prepared in 96-well flat bottom polystyrene microplates (Corning® Cat# 3340) in buffer composed of 100 mM sodium acetate, 100 mM sodium chloride, 10 mM DTT, and 1 mM EDTA, and adjusted to pH 5.0 or 7.4. The cathepsin B inhibitor E64 was added or not into the sample, and pre-incubated for 10 min. The enzymatic reactions were then initiated by spiking human cathepsin B (40 nM) or not. The fluorescence intensity was monitored every 3 min over 180 min. Fold increases in fluorescence emission over time were calculated by normalizing each time point to the initial fluorescence value of the caged compound **A17-C**. Data were plotted using GraphPad.

Cell culture and luminescence-based payload release reporter assay. K562 GRE cells[3] were cultured in RPMI medium (phenol red-free) supplemented with L-glutamine, 10% FBS, 1% sodium pyruvate, and 1% MEM non-essential amino acids. Hygromycin B (50 mg mL^{-1}) was added at a final concentration of 125 µg mL^{-1} to maintain selection pressure. K562 GRE mTNFα cells were maintained under the same conditions, with the addition of 0.5 mg mL^{-1} geneticin/neomycin to ensure selection for the mTNFα-expressing population. Cells were seeded in 96-well white flat-bottom plates at a density of 50,000 cells per well in 75 µL of assay media (phenol red-free RPMI supplemented with 1% L-glutamine, 1% sodium pyruvate, 1% essential amino acids, 1% charcoal-stripped fetal bovine serum, 125 µg mL^{-1} hygromycin B, and 0.5

mg mL^{-1} geneticin/neomycin or not). The cells were incubated at 37°C with 5% CO_2 for 5 h to allow stabilization. Following the initial incubation, the assay plates were treated with 25 µL of serially diluted ADCs (maximum final concentration: 25 µg mL^{-1}) and incubated for 60 h. After incubation, the plates were removed from the incubator and allowed to cool to r.t. for approximately 10 min. Next, 100 µL of Promega Dual-Glo substrate was added to each well, following the manufacturer's instructions. The plates were allowed to incubate at r.t. for 10 min to ensure complete cell lysis. Luminescence was then measured using a Synergy HT spectrophotometer. Data were analyzed and plotted using GraphPad.

2. Chemical Synthesis

General Procedure A. The aniline building blocks (1.5 equiv., 0.15 mmol), Cs_2CO_3 (3.0 equiv., 0.3 mmol) and XPHOS Pd G2 (0.1 equiv., 0.01 mmol) were weighed into a 4 mL vial equipped with a stir bar. The vial was moved to a nitrogen-atmosphere dry box. There, a solution of 6-(6-bromo-1,3-dioxo-1H-benzo[de]isoquinolin-2(3H)-yl)hexanoic acid (1 equiv., 0.1 mmol) in 1.0 mL 1,4-dioxane was added to the vial. The vial was capped and placed to heat/stir at 90°C for 4 h. Upon completion, the crude material was then passed through a filter plate containing Celite, washed with MeOH, and then concentrated to dryness. Then, it was re-dissolved in 1.8 mL H_2O:DMSO (1:9), analyzed by LCMS and purified by reverse-phase HPLC to provide the final compounds.

General Procedure B. The amine building block (1.5 equiv., 0.15 mmol), was weighed into a 4 mL vial equipped with a stir bar. Then, a solution of 6-(6-bromo-1,3-dioxo-1H-benzo[de]isoquinolin-2(3H)-yl)hexanoic acid (1 equiv., 0.09 mmol) in 1.0 mL DMSO was added to the vial followed by triethylamine neat (27 equiv., 355 µL). The vial was capped and placed to heat/stir at 100°C overnight. Upon completion, the crude material was then passed through a filter plate containing Celite. Then, an additional 800 µL H_2O:DMSO (1:9) were added, analyzed by LCMS and purified by reverse-phase HPLC to provide the final compounds.

HPLC purification and analysis. Samples were purified by preparative HPLC on two coupled C_8 5 μm 100 Å columns (30 mm × 75 mm). The two methods were used interchangeably:

Method 1: A gradient of ACN (A) and 0.1% TFA in H_2O (B) was used, at a flow rate of 50 mL min^{-1} (0 - 0.5 min 5% A, 0.5-8.5 min linear gradient 5 -100% A, 8.7-10.7 min 100% A, 10.7 - 11 min linear gradient 100 – 5 % A).

Method 2: A gradient of ACN (A) and 10 mM ammonium acetate in H_2O (B) was used, at a flow rate of 50 mL min^{-1} (0 - 0.5 min 5% A, 0.5 - 8.5 min linear gradient 5 - 100% A, 8.7 - 10.7 min 100% A, 10.7 - 11 min linear gradient 100 - 5% A).

Analytical method for HPLC traces: A gradient of 0.1 % formic acid in ACN (A) and 0.1 % formic acid in H_2O (B) was used, at a flow rate of 1.8 mL min^{-1} (0 - 0.5 min 5% A, 0.5 - 23 min linear gradient 5 - 95% A, 23 - 26 min 95% A, 26.2 - 29 min 5% A).

6-(6-Bromo-1,3-dioxo-1H-benzo[de]isoquinolin-2(3H)-yl)hexanoic acid (compound 2)

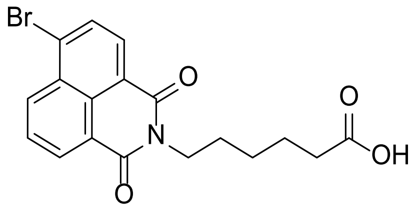

6-Aminocaproic acid (0.67 g, 5.1 mmol, 1 equiv.) and 4-bromo-1,8-naphthalic anhydride (1.39 g, 5.0 mmol, 10 equiv.) were suspended in DMSO (10 mL). The brown suspension was heated to 110 °C under nitrogen for 16 h. The resulting dark solution was cooled to r.t., poured into water (75 mL) and stirred for 30 min to precipitate the product. The product was filtered and washed with water (3 x 15 mL) to give a yellow solid that was dried in the vacuum oven to afford 6-(6-bromo-1,3-dioxo-1H-benzo[de]isoquinolin-2(3H)-yl)hexanoic acid (1.73 g, 84% yield), which was used without further purification.

^{1}H NMR (400 MHz, DMSO-d6) δ 12.00 (s, 1H), 8.43 (d, J = 7.2 Hz, 1H), 8.38 (d, J = 8.4 Hz, 1H), 8.18 (d, J = 7.8 Hz, 1H), 8.08 (d, J = 7.8 Hz, 1H), 7.88 (t, J = 7.9 Hz, 1H), 3.95 (t, J = 7.5 Hz, 2H), 2.21 (t, J = 7.4 Hz, 2H), 1.73 – 1.64 (m, 2H), 1.58 – 1.53 (m, 2H), 1.38 – 1.29 (m, 2H).

^{13}C NMR (101 MHz, DMSO-d6) δ 174.4, 162.7, 162.6, 132.4, 131.4, 131.2, 130.8, 129.6, 129.0, 128.6, 128.1, 122.5, 121.8, 39.6, 33.5, 27.1, 26.0, 24.2.

6-(6-((4-Fluorophenyl)amino)-1,3-dioxo-1H-benzo[de]isoquinolin-2(3H)-yl)hexanoic acid (A1)

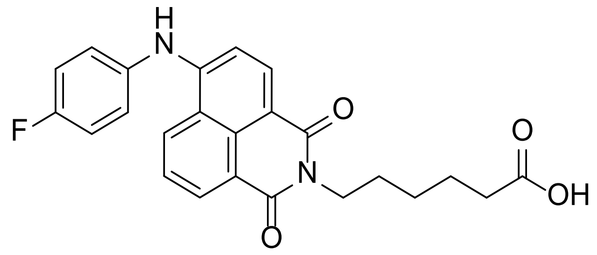

The title compound was prepared following **General Procedure A.** 6-(6-bromo-1,3-dioxo-1H-benzo[de]isoquinolin-2(3H)-yl)hexanoic acid (0.05 g, 0.137 mmol) was used as the aryl halide core and 4-fluoroaniline (0.05 mL, 0.495 mmol) was used as the aniline building block. The crude residue was purified by normal-phase silica-gel chromatography eluting with a gradient of 0-10% MeOH/DCM to give 6-(6-((4-fluorophenyl)amino)-1,3-dioxo-1H-benzo[de]isoquinolin-2(3H)-yl)hexanoic acid (45 mg, 79% yield) .

^{1}H NMR (400 MHz, DMSO-d6) δ 11.95 (s, 1H), 9.32 (s, 1H), 8.77 (d, J = 8.4 Hz, 1H), 8.46 (d, J = 7.3 Hz, 1H), 8.22 (d, J = 8.5 Hz, 1H), 7.80 – 7.71 (m, 1H), 7.44 – 7.34 (m, 2H), 7.32 – 7.21 (m, 2H), 7.06 (d, J = 8.5 Hz, 1H), 3.97 (t, J = 7.4 Hz, 2H), 2.17 (t, J = 7.3 Hz, 2H), 1.67 – 1.49 (m, , 4H), 1.38 – 1.29 (m, 2H).

^{13}C NMR (101 MHz, DMSO-d6) δ 174.0, 164.1, 163.2, 160.7, 158.3, 148.8, 137.0, 133.9, 131.4, 129.9, 129.3, 125.8, 125.5, 122.5, 121.7, 116.9, 116.6, 111.3, 107.6, 39.6, 34.0, 27.8, 26.5, 24.7.

HRMS (ESI) for $C_{24}H_{22}FN_2O_4$ m/z $[M+H]^+$ calcd.: 421.1558, found: 421.1556.

6-(6-((2-(bis(pyridin-2-ylmethyl)amino)ethyl)amino)-1,3-dioxo-1H-benzo[de]isoquinolin-2(3H)-yl)hexanoic acid (A2)

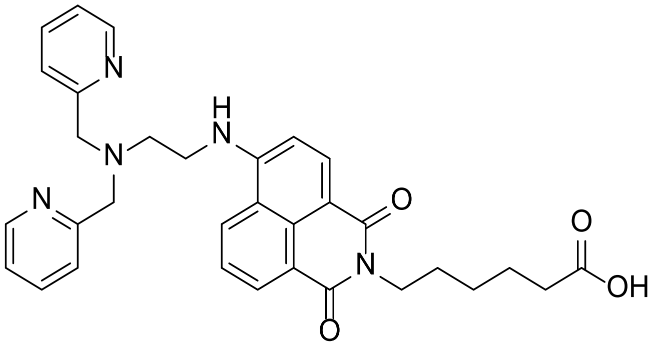

The title compound was prepared following **General Procedure B.** N^1,N^1-bis(pyridin-2-ylmethyl)ethane-1,2-diamine (0.10 g, 0.4 mmol) was dissolved in 2-methoxyethanol (1 mL) and then transferred to a vial containing 6-(6-bromo-1,3-dioxo-1H-benzo[de]isoquinolin-2(3H)-yl)hexanoic acid (0.05 g, 0.13 mmol) and triethylamine (0.5 ml, 3.6 mmol). The reaction was heated to 88°C for 2 days followed by heating at 115°C for 7 h. The reaction mixture was cooled down to r.t., and the solvent removed under reduced pressure. The residue was purified by normal-phase silica gel chromatography eluting with a gradient of 0-50% MeOH in DCM. The compound was further purified by reverse-phase preparative HPLC to afford the title compound 6-(6-((2-(bis(pyridin-2-ylmethyl)amino)ethyl)amino)-1,3-dioxo-1H-benzo[de]isoquinolin-2(3H)-yl)hexanoic acid (13 mg, 18% yield).

^{1}H NMR (400 MHz, DMSO-d6) δ 8.61 – 8.54 (m, 3H), 8.47 (d, J = 7.2 Hz, 1H), 8.23 (d, J = 8.5 Hz, 1H), 7.90 (td, J = 7.7, 1.8 Hz, 2H), 7.75 (q, J = 7.7 Hz, 2H), 7.58 (d, J = 7.8 Hz, 2H), 7.43 (dd, J = 7.5, 4.9 Hz, 2H), 6.80 (d, J = 8.6 Hz, 1H), 4.55 (s, 4H), 4.01 (t, J = 7.4 Hz, 2H), 3.84 (t, J = 6.8 Hz, 2H), 3.40 (t, J = 6.7 Hz, 2H), 2.22 (t, J = 7.3 Hz, 2H), 1.65 – 1.50 (m, 4H), 1.37 – 1.29 (m, 2H).

^{13}C NMR (101 MHz, DMSO-d6) δ 174.9, 164.1, 163.4, 152.6, 150.3, 148.6, 139.2, 134.3, 131.2, 129.7, 128.9, 125.3, 125.0, 124.5, 122.4, 120.8, 109.0, 104.5, 57.6, 51.8, 39.6, 38.9, 34.0, 27.8, 26.5, 24.7.

HRMS (ESI) for $C_{32}H_{34}N_5O_4$ m/z $[M+H]^+$ calcd.: 552.2605, found: 552.2603.

6-(6-((4-(ethylsulfonyl)phenyl)amino)-1,3-dioxo-1H-benzo[de]isoquinolin-2(3H)-yl)hexanoic acid (A3)

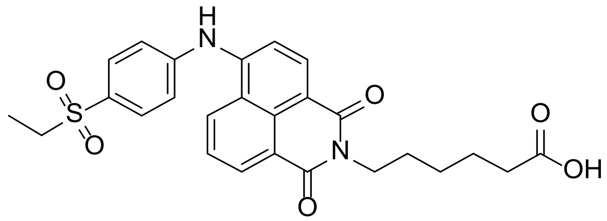

The title compound was prepared following **General Procedure A.** 6-(6-bromo-1,3-dioxo-1H-benzo[de]isoquinolin-2(3H)-yl)hexanoic acid (39 mg 0.1 mmol) was used as the aryl halide core and 4-(ethylsulfonyl)aniline (28 mg, 0.15 mmol) was used as the aniline building block. The crude residue was purified by preparative reverse-phase HPLC (mass triggered collection) to afford the title compound 6-(6-((4-(ethylsulfonyl)phenyl)amino)-1,3-dioxo-1H-benzo[de]isoquinolin-2(3H)-yl)hexanoic acid (26 mg, 52% yield).

^{1}H NMR (400 MHz, DMSO-d6) δ 11.97 (s, 1H), 9.71 (s, 1H), 8.73 (dd, J = 8.6, 1.1 Hz, 1H), 8.57 – 8.51 (m, 1H), 8.40 (d, J = 8.3 Hz, 1H), 7.91 – 7.79 (m, 3H), 7.68 (d, J = 8.3 Hz, 1H), 7.56 – 7.48 (m, 2H), 4.03 (t, J = 7.3 Hz, 2H), 3.26 (q, J = 7.3 Hz, 2H), 2.22 (t, J = 7.3 Hz, 2H), 1.68 – 1.51 (m, 4H), 1.41 – 1.29 (m, 2H), 1.14 (t, J = 7.4 Hz, 3H).

^{13}C NMR (101 MHz, DMSO-d6) δ 174.9, 163.9, 163.1, 147.2, 145.2, 132.9, 131.5, 131.0, 130.1, 129.5, 126.2, 123.83, 123.81, 122.6, 119.2, 114.7, 112.8, 50.0, 39.3, 34.0, 27.7, 26.5, 24.7, 7.8.

HRMS (ESI) for $C_{26}H_{27}N_2O_6S$, m/z $[M+H]^+$ calcd.: 495.1584, found: 495.1582.

6-(6-((3-(*tert*-butyl)phenyl)amino)-1,3-dioxo-1H-benzo[de]isoquinolin-2(3H)-yl)hexanoic acid (A4)

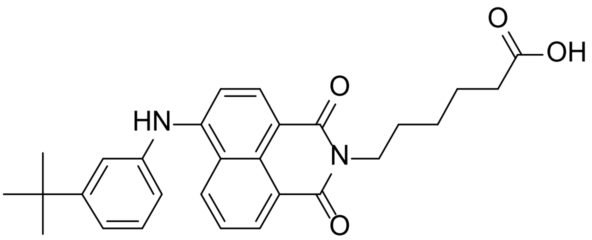

The title compound was prepared following **General Procedure A.** 6-(6-bromo-1,3-dioxo-1H-benzo[de]isoquinolin-2(3H)-yl)hexanoic acid (39 mg, 0.1 mmol) was used as the aryl halide core and 3-*tert*-butylaniline (22 mg, 0.15 mmol) was used as the aniline building block. The crude residue was purified by preparative reverse-phase HPLC (mass triggered collection) to afford the title compound 6-(6-((3-(tert-butyl)phenyl)amino)-1,3-dioxo-1H-benzo[de]isoquinolin-2(3H)-yl)hexanoic acid (24 mg, 53% yield).

^{1}H NMR (400 MHz, DMSO-d6) δ 11.97 (s, 1H), 9.38 (s, 1H), 8.83 (dd, J = 8.6, 1.1 Hz, 1H), 8.50 (dd, J = 7.3, 1.0 Hz, 1H), 8.27 (d, J = 8.5 Hz, 1H), 7.83 – 7.75 (m, 1H), 7.43 – 7.34 (m, 2H), 7.27 – 7.20 (m, 3H), 4.02 (t, J = 7.4 Hz, 2H), 2.21 (t, J = 7.3 Hz, 2H), 1.68 – 1.49 (m, 4H), 1.40 – 1.33 (m, 2H), 1.32 (s, 9H).

^{13}C NMR (101 MHz, DMSO-d6) δ 174.4 (C), 163.6 (C), 162.8 (C), 152.3 (C), 148.0 (C), 140.0 (C), 133.4 (CH), 131.0 (CH), 129.5 (C), 129.1 (CH), 128.9 (CH), 124.9 (CH), 122.0 (C), 121.5 (C), 121.3 (CH), 120.0 (CH), 119.7 (CH), 110.7 (C), 107.5 (CH), 39.2 (CH_2), 34.5 (C), 33.5 (CH_2), 31.1 (CH_3 x 3), 27.4 (CH_2), 26.1 (CH_2), 24.2 (CH_2).

HRMS (ESI) for $C_{28}H_{30}N_2O_4$ m/z $[M+H]^+$ calcd.: 459.2278, found: 459.2282.

6-(1,3-dioxo-6-((4-(trifluoromethyl)phenyl)amino)-1H-benzo[de]isoquinolin-2(3H)-yl)hexanoic acid (A5)

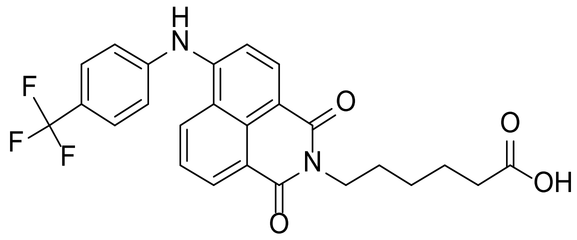

The title compound was prepared following **General Procedure A.** 6-(6-bromo-1,3-dioxo-1H-benzo[de]isoquinolin-2(3H)-yl)hexanoic acid (39 mg, 0.1mmol) was used as the aryl halide core and 4-(trifluoromethyl)aniline (24 mg, 0.15 mmol) was used as the aniline building block. The crude residue was purified by preparative reverse-phase HPLC (mass triggered collection) to afford the title compound 6-(1,3-dioxo-6-((4-(trifluoromethyl)phenyl)amino)-1H-benzo[de]isoquinolin-2(3H)-yl)hexanoic acid**.** (23 mg, 50% yield).

^{1}H NMR (400 MHz, DMSO-d6) δ 11.97 (s, 1H), 9.60 (s, 1H), 8.75 (dd, J = 8.7, 1.2 Hz, 1H), 8.53 (dd, J = 7.3, 1.0 Hz, 1H), 8.36 (d, J = 8.3 Hz, 1H), 7.89 – 7.81 (m, 1H), 7.73 (d, J = 8.5 Hz, 2H), 7.58 (d, J = 8.4 Hz, 1H), 7.52 (d, J = 8.4 Hz, 2H), 4.03 (t, J = 7.4 Hz, 2H), 2.22 (t, J = 7.3 Hz, 2H), 1.69 – 1.49 (m, 4H), 1.40 – 1.28 (m, 2H).

^{13}C NMR (101 MHz, DMSO-d6) δ 174.9, 163.8, 163.1, 145.8, 145.6, 133.1, 131.4, 129.5, 129.4, 127.1, 127.0, 126.9, 126.3, 125.9, 123.6, 123.2, 123.0, 122.7, 122.4, 120.3, 113.8, 111.1, 33.9, 27.7, 26.5, 24.7.

HRMS (ESI) for $C_{25}H_{22}F_3N_2O_4$ m/z $[M+H]^+$ calcd.: 471.1526, found: 471.1524.

6-(1,3-dioxo-6-(phenylamino)-1H-benzo[de]isoquinolin-2(3H)-yl)hexanoic acid (A6)

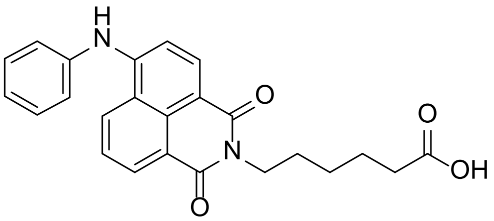

The title compound was prepared following **General Procedure A.** 6-(6-bromo-1,3-dioxo-1H-benzo[de]isoquinolin-2(3H)-yl)hexanoic acid (39 mg, 0.1 mmol) was used as the aryl halide core and aniline (14 mg, 0.15 mmol) was used as the aniline building block. The crude residue was purified by preparative reverse-phase HPLC (mass triggered collection) to afford the title compound 6-(1,3-dioxo-6-(phenylamino)-1H-benzo[de]isoquinolin-2(3H)-yl)hexanoic acid (19 mg, 48% yield).

^{1}H NMR (400 MHz, DMSO-d6) δ 11.97 (s, 1H), 9.39 (s, 1H), 8.83 (d, J = 8.4 Hz, 1H), 8.51 (d, J = 7.2 Hz, 1H), 8.28 (d, J = 8.5 Hz, 1H), 7.80 (t, 7.3 Hz, 1H), 7.50 – 7.36 (m, 4H), 7.28 – 7.15 (m, 2H), 4.02 (t, J = 7.4 Hz, 2H), 2.21 (t, J = 7.3 Hz, 2H), 1.68 – 1.49 (m, 4H), 1.40 – 1.27 (m, 2H).

^{13}C NMR (101 MHz, DMSO-d6) δ 174.9, 164.0, 163.1, 148.2, 140.8, 133.7, 131.31, 131.29, 130.0, 129.8, 129.3, 125.4, 124.6, 123.1, 122.3, 121.9, 111.4, 108.0, 39.6, 34.0, 27.8, 26.5, 24.7.

HRMS (ESI) for $C_{24}H_{22}N_2O_4$, m/z $[M+H]^+$ calcd.: 403.1652, found: 403.1651.

6-(6-((3-(oxazol-5-yl)phenyl)amino)-1,3-dioxo-1H-benzo[de]isoquinolin-2(3H)-yl)hexanoic acid (A7)

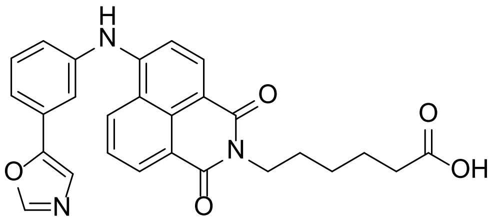

The title compound was prepared following **General Procedure A.** 6-(6-bromo-1,3-dioxo-1H-benzo[de]isoquinolin-2(3H)-yl)hexanoic acid (39 mg, 0.1mmol) was used as the aryl halide core and 3-oxazol-5-ylaniline (24 mg, 0.15 mmol) was used as the aniline building block. The crude residue was purified by preparative reverse-phase HPLC (mass triggered collection) to afford the title compound 6-(6-((3-(oxazol-5-yl)phenyl)amino)-1,3-dioxo-1H-benzo[de]isoquinolin-2(3H)-yl)hexanoic acid (12 mg, 26% yield).

^{1}H NMR (400 MHz, DMSO-d6) δ 11.97 (s, 1H), 9.49 (s, 1H), 8.83 (dd, J = 8.5, 1.2 Hz, 1H), 8.52 (dd, J = 7.4, 1.0 Hz, 1H), 8.47 (s, 1H), 8.32 (d, J = 8.4 Hz, 1H), 7.83 (dd, J = 8.5, 7.3 Hz, 1H), 7.76 – 7.71 (m, 2H), 7.60 – 7.50 (m, 2H), 7.45 – 7.38 (m, 1H), 7.35 (d, J = 8.4 Hz, 1H), 4.03 (t, J = 7.4 Hz, 2H), 2.22 (t, J = 7.3 Hz, 2H), 1.69 – 1.49 (m, 4H), 1.40 – 1.28 (m, 2H).

^{13}C NMR (101 MHz, DMSO-d6) δ 174.9, 164.0, 163.2, 152.4, 150.6, 147.7, 141.9, 133.7, 131.4, 130.8, 129.8, 129.3, 129.1, 125.7, 122.9, 122.6, 122.5, 122.3, 120.0, 118.0, 112.1, 108.9, 39.7, 34.0, 27.8, 26.5, 24.7.

HRMS (ESI) for $C_{27}H_{24}N_3O_5$, m/z $[M+H]^+$ calcd.: 470.1710, found: 470.1709.

6-(6-((3,4-dimethoxyphenyl)amino)-1,3-dioxo-1H-benzo[de]isoquinolin-2(3H)-yl)hexanoic acid (A8)

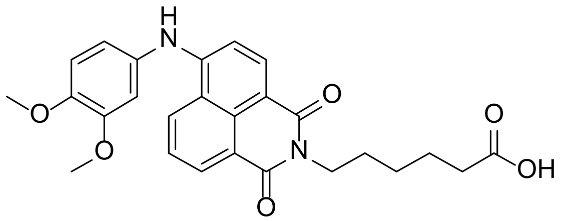

The title compound was prepared following **General Procedure A.** 6-(6-bromo-1,3-dioxo-1H-benzo[de]isoquinolin-2(3H)-yl)hexanoic acid (39 mg, 0.1 mmol) was used as the aryl halide core and 3,4-dimethoxyaniline (23 mg, 0.15 mmol) was used as the aniline building block. The crude residue was purified by preparative reverse-phase HPLC (mass triggered collection) to afford the title compound 6-(6-((3,4-dimethoxyphenyl)amino)-1,3-dioxo-1H-benzo[de]isoquinolin-2(3H)-yl)hexanoic acid (24 mg, 53% yield).

^{1}H NMR (400 MHz, DMSO-d6) δ 9.31 (s, 1H), 8.82 (dd, J = 8.7, 1.2 Hz, 1H), 8.48 (dd, J = 7.3, 1.0 Hz, 1H), 8.23 (d, J = 8.5 Hz, 1H), 7.81 – 7.72 (m, 1H), 7.08 – 6.90 (m, 4H), 4.05 – 3.97 (m, 2H), 3.79 (d, J = 10.7 Hz, 6H), 2.21 (t, J = 7.3 Hz, 2H), 1.67 – 1.49 (m, 4H), 1.39 – 1.27 (m, 2H).

^{13}C NMR (101 MHz, DMSO-d6) δ 174.9, 164.1, 163.2, 149.9, 149.5, 146.8, 134.0, 133.4, 131.3, 129.8, 129.1, 125.1, 122.3, 121.3, 116.5, 112.9, 110.3, 109.3, 107.1, 56.2, 56.0, 39.6, 34.0, 27.8, 26.5, 24.7.

HRMS (ESI) for $C_{26}H_{27}N_2O_6$, m/z $[M+H]^+$ calcd.: 463.1864, found: 463.1862.

6-(1,3-dioxo-6-((4-(trifluoromethoxy)phenyl)amino)-1H-benzo[de]isoquinolin-2(3H)-yl)hexanoic acid (A9)

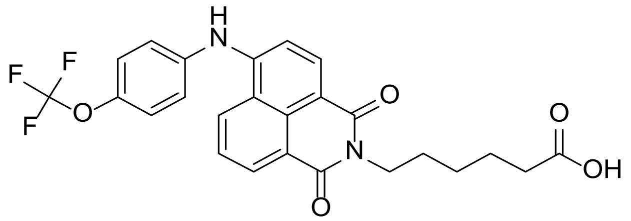

The title compound was prepared following **General Procedure A.** 6-(6-bromo-1,3-dioxo-1H-benzo[de]isoquinolin-2(3H)-yl)hexanoic acid (39 mg, 0.1 mmol) was used as the aryl halide core and 4-(trifluoromethoxy)aniline (27 mg, 0.15 mmol) was used as the aniline building block. The crude residue was purified by preparative reverse-phase HPLC (mass triggered collection) to afford the title compound 6-(1,3-dioxo-6-((4-(trifluoromethoxy)phenyl)amino)-1H-benzo[de]isoquinolin-2(3H)-yl)hexanoic acid (17 mg, 35% yield).

^{1}H NMR (400 MHz, DMSO-d6) δ 11.97 (s, 1H), 9.45 (s, 1H), 8.78 (d, 1H), 8.52 (d, J = 7.2 Hz, 1H), 8.31 (d, J = 8.4 Hz, 1H), 7.82 (t, J = 7.3 Hz, 1H), 7.54 – 7.47 (m, 2H), 7.46 – 7.40 (m, 2H), 7.34 (d, J = 8.4 Hz, 1H), 4.02 (t, J = 7.4 Hz, 2H), 2.21 (t, J = 7.3 Hz, 2H), 1.68 – 1.49 (m, 4H), 1.40 – 1.28 (m, 2H).

^{13}C NMR (101 MHz, DMSO-d6) δ 174.9, 163.9, 163.1, 147.4, 144.4 (q, J = 1.9 Hz), 140.4, 133.5, 131.4, 129.7, 129.3, 125.6, 124.5, 123.7, 122.7, 122.4, 122.3, 121.9, 119.4, 116.8, 112.3, 108.8, 39.6, 34.0, 27.8, 26.5, 24.7.

HRMS (ESI) for $C_{25}H_{22}F_3N_2O_5$, m/z $[M+H]^+$ calcd.: 487.1475, found: 487.1473.

6-(1,3-dioxo-6-((3-(trifluoromethoxy)phenyl)amino)-1H-benzo[de]isoquinolin-2(3H)-yl)hexanoic acid (A10)

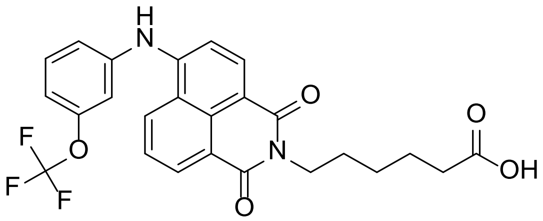

The title compound was prepared following **General Procedure A.** 6-(6-bromo-1,3-dioxo-1H-benzo[de]isoquinolin-2(3H)-yl)hexanoic acid (39 mg, 0.1 mmol) was used as the aryl halide core and 3-(trifluoromethoxy)aniline (27 mg, 0.15 mmol) was used as the aniline building block. The crude residue was purified by preparative reverse-phase HPLC (mass triggered collection) to afford the title compound 6-(1,3-dioxo-6-((3-(trifluoromethoxy)phenyl)amino)-1H-benzo[de]isoquinolin-2(3H)-yl)hexanoic acid (26 mg, 54% yield).

^{1}H NMR (400 MHz, DMSO-d6) δ 11.97 (s, 1H), 9.49 (s, 1H), 8.76 (dd, J = 8.5, 1.1 Hz, 1H), 8.52 (d, J = 7.2 Hz, 1H), 8.33 (d, J = 8.4 Hz, 1H), 7.87 – 7.80 (m, 1H), 7.54 (t, J = 8.2 Hz, 1H), 7.46 – 7.41 (m, 2H), 7.34 – 7.30 (m, 1H), 7.09 (dd, J = 8.3, 2.3 Hz, 1H), 4.02 (t, J = 7.4 Hz, 2H), 2.22 (t, J = 7.3 Hz, 2H), 1.69 – 1.49 (m, 4H), 1.40 – 1.28 (m, 2H).

^{13}C NMR (101 MHz, DMSO-d6) δ 174.4 (C), 163.5 (C), 162.8 (C), 149.2 (C), 146.3 (C), 142.9 (C), 133.0 (CH), 131.1 (CH), 131.1 (CH), 129.3 (C), 128.9 (CH), 125.5 (CH), 122.3 (C), 122.1 (C), 120.1 (C, q, J_{CF} = 256.7 Hz), 119.7 (CH), 115.1 (CH), 113.4 (CH), 112.7 (C), 109.5 (CH), 39.2 (CH_2), 33.5 (CH_2), 27.3 (CH_2), 26.1 (CH_2), 24.2 (CH_2).

^{19}F NMR (376 MHz, DMSO-d6) δ -56.63.

HRMS (ESI) for $C_{25}H_{21}F_3N_2O_5$ m/z $[M+H]^+$ calcd.: 487.1475, found: 487.1479.

6-(6-((4-(dimethylamino)phenyl)amino)-1,3-dioxo-1H-benzo[de]isoquinolin-2(3H)-yl)hexanoic acid (A11)

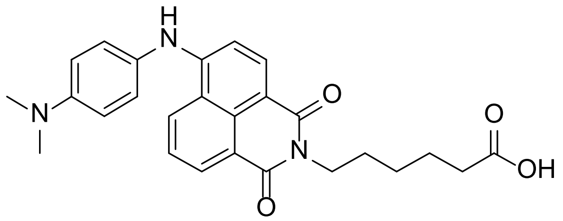

The title compound was prepared following **General Procedure A.** 6-(6-bromo-1,3-dioxo-1H-benzo[de]isoquinolin-2(3H)-yl)hexanoic acid (39 mg, 0.1 mmol) was used as the aryl halide core and N1,N1-dimethylbenzene-1,4-diamine (20 mg, 0.15 mmol) was used as the aniline building block. The crude residue was purified by preparative reverse-phase HPLC (mass triggered collection) to afford the title compound 6-(6-((4-(dimethylamino)phenyl)amino)-1,3-dioxo-1H-benzo[de]isoquinolin-2(3H)-yl)hexanoic acid (7 mg, 15% yield).

^{1}H NMR (400 MHz, DMSO-d6) δ 9.31 (s, 1H), 8.82 (d, J = 8.5 Hz, 1H), 8.49 (d, J = 7.3 Hz, 1H), 8.22 (d, J = 8.5 Hz, 1H), 7.76 (t, J = 7.9 Hz, 1H), 7.27 (d, J = 8.3 Hz, 2H), 7.05 – 6.88 (m, 3H), 4.01 (t, J = 7.4 Hz, 2H), 2.99 (s, 6H), 2.21 (t, J = 7.3 Hz, 2H), 1.67 – 1.48 (m, 4H), 1.39 – 1.27 (m, 2H).

^{13}C NMR (101 MHz, DMSO-d6) δ 174.9, 164.1, 163.2, 149.9, 147.4, 134.1, 131.5, 130.5, 129.9, 129.2, 125.8, 125.2, 122.3, 121.2, 114.8, 109.9, 106.9, 41.5, 39.5, 33.9, 27.7, 26.4, 24.6.

HRMS (ESI) for $C_{26}H_{28}N_3O_4$, m/z $[M+H]^+$ calcd.: 446.2074, found: 446.2074.

6-(6-((4-methoxyphenyl)amino)-1,3-dioxo-1H-benzo[de]isoquinolin-2(3H)-yl)hexanoic acid (A12)

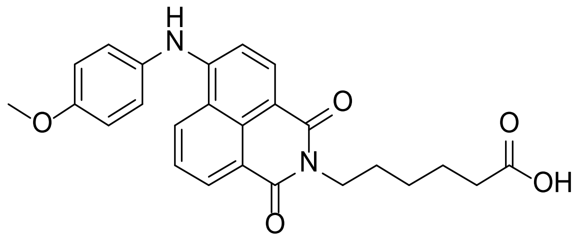

The title compound was prepared following **General Procedure A.** 6-(6-bromo-1,3-dioxo-1H-benzo[de]isoquinolin-2(3H)-yl)hexanoic acid (39 mg, 0.1 mmol) was used as the aryl halide core and 4-methoxyaniline (19 mg, 0.15 mmol) was used as the aniline building block. The crude residue was purified by preparative reverse-phase HPLC (mass triggered collection) to afford the title compound 6-(6-((4-methoxyphenyl)amino)-1,3-dioxo-1H-benzo[de]isoquinolin-2(3H)-yl)hexanoic acid (20 mg, 47% yield).

^{1}H NMR (400 MHz, DMSO-d6) δ 11.97 (s, 1H), 9.31 (s, 1H), 8.83 (d, J = 8.4 Hz, 1H), 8.49 (d, J = 7.2 Hz, 1H), 8.22 (d, J = 8.5 Hz, 1H), 7.77 (t, J = 7.9 Hz, 1H), 7.37 – 7.28 (m, 2H), 7.10 – 7.01 (m, 2H), 6.94 (d, J = 8.5 Hz, 1H), 4.01 (t, J = 7.4 Hz, 2H), 3.80 (s, 3H), 2.21 (t, J = 7.3 Hz, 2H), 1.67 – 1.48 (m, 4H), 1.39 – 1.27 (m, 2H).

^{13}C NMR (101 MHz, DMSO-d6) δ 174.9, 164.0, 163.2, 157.2, 149.6, 134.0, 133.0, 131.3, 129.9, 129.1, 126.3, 126.2, 125.1, 122.3, 121.2, 115.3, 110.2, 106.7, 55.8, 39.6, 34.0, 27.8, 26.5, 24.7.

HRMS (ESI) for $C_{25}H_{25}N_2O_5$, m/z $[M+H]^+$ calcd.: 433.1758, found: 433.1757.

6-(1,3-dioxo-6-((pyridin-2-ylmethyl)amino)-1H-benzo[de]isoquinolin-2(3H)-yl)hexanoic acid (A13)

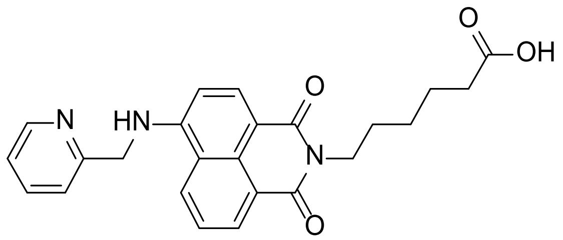

The title compound was prepared following **General Procedure B.** 6-(6-bromo-1,3-dioxo-1H-benzo[de]isoquinolin-2(3H)-yl)hexanoic acid (35 mg, 0.09 mmol) was used as the aryl halide core and 2-(aminomethyl)pyridine (16 mg, 0.15 mmol) was used as the amine building block. The crude residue was purified by preparative reverse-phase HPLC (mass triggered collection) to afford the title compound 6-(1,3-dioxo-6-((pyridin-2-ylmethyl)amino)-1H-benzo[de]isoquinolin-2(3H)-yl)hexanoic acid (6 mg, 12% yield). **^{1}H NMR** (400 MHz, DMSO-d6) δ 8.76 (d, J = 8.4 Hz, 1H), 8.62 (d, J = 5.0 Hz, 1H), 8.54 – 8.40 (m, 2H), 8.19 (d, J = 8.5 Hz, 1H), 7.88 (t, J = 7.8 Hz, 1H), 7.76 (t, J = 7.9 Hz, 1H), 7.49 (d, J = 7.9 Hz, 1H), 7.44 – 7.37 (m, 1H), 6.67 (d, J = 8.5 Hz, 1H), 4.79 (s, 2H), 4.03 – 3.94 (m, 2H), 2.20 (t, J = 7.3 Hz, 2H), 1.65 – 1.47 (m, 4H), 1.37 – 1.25 (m, 2H).

HRMS (ESI) for $C_{24}H_{24}N_3O_4$, m/z $[M+H]^+$ calcd.: 418.1761, found: 418.1760.

6-(6-(benzylamino)-1,3-dioxo-1H-benzo[de]isoquinolin-2(3H)-yl)hexanoic acid (A14)

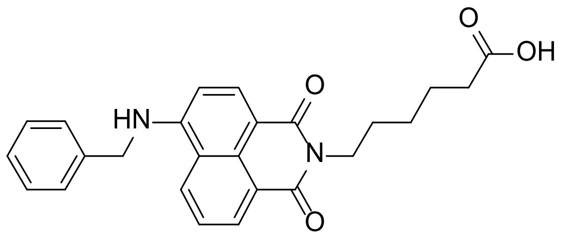

The title compound was prepared following **General Procedure B.** 6-(6-bromo-1,3-dioxo-1H-benzo[de]isoquinolin-2(3H)-yl)hexanoic acid (35 mg, 0.09 mmol) was used as the aryl halide core and benzylamine (16 mg, 0.15 mmol) was used as the amine building block. The crude residue was purified by preparative reverse-phase HPLC (mass triggered collection) to afford the title compound 6-(6-(benzylamino)-1,3-dioxo-1H-benzo[de]isoquinolin-2(3H)-yl)hexanoic acid (8 mg, 21% yield).

^{1}H NMR (400 MHz, DMSO-d6) δ 11.96 (s, 1H), 8.78 (dd, J = 8.6, 1.2 Hz, 1H), 8.51 – 8.44 (m, 2H), 8.18 (d, J = 8.5 Hz, 1H), 7.77 – 7.71 (m, 1H), 7.44 – 7.38 (m, 2H), 7.37 – 7.29 (m, 2H), 7.28 – 7.21 (m, 1H), 6.68 (d, J = 8.6 Hz, 1H), 4.67 (d, J = 5.9 Hz, 2H), 3.99 (t, J = 7.4 Hz, 2H), 2.20 (t, J = 7.3 Hz, 2H), 1.65 – 1.47 (m, 4H), 1.37 – 1.21 (m, 2H).

^{13}C NMR (101 MHz, DMSO-d6) δ 164.1, 163.3, 150.8, 138.8, 134.4, 131.1, 129.7, 128.9, 127.4, 127.3, 125.0, 122.4, 120.7, 108.5, 105.0, 46.3, 39.6, 27.9, 26.8, 25.4.

HRMS (ESI) for $C_{25}H_{25}N_2O_4$, m/z $[M+H]^+$ calcd.: 417.1809, found: 417.1808.

6-(6-((2-(4-methylpiperazin-1-yl)ethyl)amino)-1,3-dioxo-1H-benzo[de]isoquinolin-2(3H)-yl)hexanoic acid (A15)

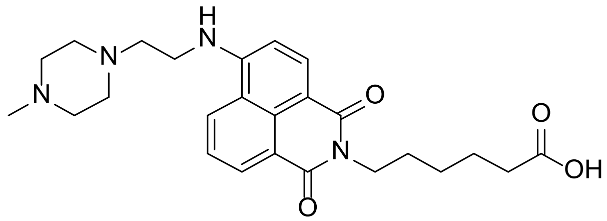

The title compound was prepared following **General Procedure B.** 6-(6-bromo-1,3-dioxo-1H-benzo[de]isoquinolin-2(3H)-yl)hexanoic acid (35 mg, 0.09 mmol) was used as the aryl halide core and 2-(4-methylpiperazin-1-yl)ethanamine (22 mg, 0.15 mmol) was used as the amine building block. The crude residue was purified by preparative reverse-phase HPLC (mass triggered collection) to afford the title compound 6-(6-((2-(4-methylpiperazin-1-yl)ethyl)amino)-1,3-dioxo-1H-benzo[de]isoquinolin-2(3H)-yl)hexanoic acid (12 mg, 22% yield).

^{1}H NMR (400 MHz, DMSO-d6) δ 8.65 (d, J = 8.4 Hz, 1H), 8.46 (d, J = 7.2 Hz, 1H), 8.30 (d, J = 8.5 Hz, 1H), 7.76 – 7.69 (m, 1H), 7.66 (s, 1H), 6.86 (d, J = 8.6 Hz, 1H), 4.00 (t, J = 7.4 Hz, 2H), 3.03 – 2.95 (m, 2H), 2.80 (s, 3H), 2.21 (t, J = 7.3 Hz, 2H), 1.66 – 1.48 (m, 4H), 1.38 – 1.29 (m, 2H).

[Note: piperazine signal not visible under the water peak]

^{13}C NMR (101 MHz, DMSO-d6) δ 174.9, 164.2, 163.4, 158.8, 150.7, 134.6, 131.2, 129.8, 128.9, 124.9, 122.4, 120.7, 108.7, 104.5, 54.7, 52.1, 50.1, 49.6, 42.6, 40.8, 34.0, 27.9, 26.5, 24.7.

HRMS (ESI) for $C_{25}H_{33}N_4O_4$, m/z $[M+H]^+$ calcd.: 453.2496, found: 453.2497.

6-(6-((2-(dimethylamino)ethyl)amino)-1,3-dioxo-1H-benzo[de]isoquinolin-2(3H)-yl)hexanoic acid (A16)

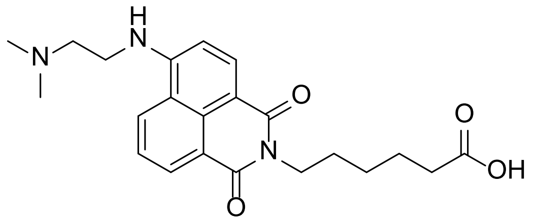

The title compound was prepared following **General Procedure B.** 6-(6-bromo-1,3-dioxo-1H-benzo[de]isoquinolin-2(3H)-yl)hexanoic acid (35 mg, 0.09 mmol) was used as the aryl halide core and N',N'-dimethylethane-1,2-diamine (13 mg, 0.15 mmol) was used as the amine building block. The crude residue was purified by preparative reverse-phase HPLC (mass triggered collection) to afford the title compound 6-(6-((2-(dimethylamino)ethyl)amino)-1,3-dioxo-1H-benzo[de]isoquinolin-2(3H)-yl)hexanoic acid (7 mg, 15% yield).

^{1}H NMR (400 MHz, DMSO-d6) δ 9.49 (s, 1H), 8.63 (d, J = 8.5 Hz, 1H), 8.48 (d, J = 7.3 Hz, 1H), 8.32 (d, J = 8.5 Hz, 1H), 7.80 – 7.65 (m, 2H), 6.92 (d, J = 8.6 Hz, 1H), 4.01 (t, J = 7.4 Hz, 2H), 3.82 – 3.73 (m, 2H), 2.89 (d, J = 4.1 Hz, 6H), 2.21 (t, J = 7.3 Hz, 2H), 1.67 – 1.49 (m, 4H), 1.39 – 1.27 (m, 2H).

[Note: 2 alkyl protons not visible under the water peak]

^{13}C NMR (101 MHz, DMSO-d6) δ 174.9, 164.2, 163.4, 150.3, 134.4, 131.3, 129.7, 129.1, 125.1, 122.4, 120.9, 109.4, 104.8, 54.9, 43.0, 40.5, 39.6, 38.2, 34.8, 34.0, 27.8, 26.5, 24.7.

HRMS (ESI) for, $C_{22}H_{28}N_3O_4$ m/z [M+H]$^+$ calcd.: 398.2074, found: 398.2075.

6-(6-(((6-morpholinopyridin-3-yl)methyl)amino)-1,3-dioxo-1H-benzo[de]isoquinolin-2(3H)-yl)hexanoic acid (A17)

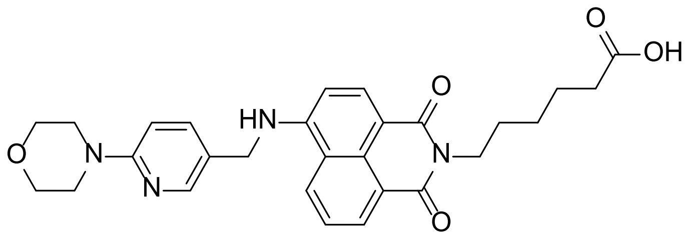

The title compound was prepared following **General Procedure B.** 6-(6-bromo-1,3-dioxo-1H-benzo[de]isoquinolin-2(3H)-yl)hexanoic acid (35 mg, 0.09 mmol) was used as the aryl halide core and (6-morpholino-3-pyridyl)methanamine (29 mg, 0.15 mmol) was used as the amine building block. The crude residue was purified by preparative reverse-phase HPLC (mass triggered collection) to afford the title compound 6-(6-(((6-morpholinopyridin-3-yl)methyl)amino)-1,3-dioxo-1H-benzo[de]isoquinolin-2(3H)-yl)hexanoic acid (15 mg, 26% yield).

^{1}H NMR (400 MHz, DMSO-d6) δ 8.72 (d, J = 8.6, 1.1 Hz, 1H), 8.45 (d, J = 7.2 Hz, 1H), 8.36 – 8.28 (m, 1H), 8.21 (d, J = 8.5 Hz, 1H), 8.19 – 8.14 (m, 1H), 7.82 (dd, J = 9.1, 2.3 Hz, 1H), 7.77 – 7.68 (m, 1H), 7.07 (d, J = 9.1 Hz, 1H), 6.80 (d, J = 8.6 Hz, 1H), 4.62 – 4.54 (m, 2H), 3.99 (t, J = 7.6 Hz, 2H), 3.69 (t, J = 5.1 Hz, 4H), 3.48 (t, J = 4.9 Hz, 4H), 2.20 (t, J = 7.4 Hz, 2H), 1.65 – 1.47 (m, 4H), 1.37 – 1.25 (m, 2H).

^{13}C NMR (101 MHz, DMSO-d6) δ 174.4 (C), 163.7 (C), 162.9 (C), 158.5 (C), 158.2 (C), 155.2 (C), 149.9 (C), 140.8 (CH), 140.4 (CH), 133.9 (CH), 130.8 (CH), 129.3 (C), 128.5 (CH), 124.6 (CH), 123.3 (C), 122.0 (C), 120.4 (C), 110.0 (CH), 108.5 (C), 104.6 (CH), 65.5 (CH_2 x 2), 45.5 (CH_2 x 2), 42.6 (CH_2), 39.1 (CH_2), 33.5 (CH_2), 27.4 (CH_2), 26.1 (CH_2), 24.3 (CH_2).

HRMS (ESI) for $C_{28}H_{30}N_4O_5$ m/z $[M+H]^+$ calcd.: 503.2289, found: 503.2292.

6-(1,3-dioxo-6-((3,3,3-trifluoropropyl)amino)-1H-benzo[de]isoquinolin-2(3H)-yl)hexanoic acid (A18)

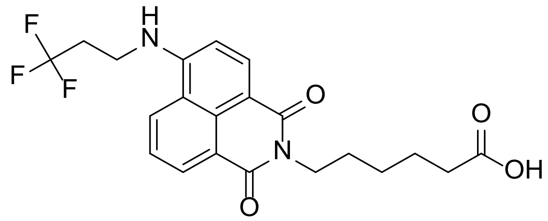

The title compound was prepared following **General Procedure B.** 6-(6-bromo-1,3-dioxo-1H-benzo[de]isoquinolin-2(3H)-yl)hexanoic acid (35 mg, 0.09 mmol) was used as the aryl halide core and 3,3,3-trifluoropropan-1-amine (17 mg, 0.15 mmol) was used as the amine building block. The crude residue was purified by preparative reverse-phase HPLC (mass triggered collection) to afford the title compound 6-(1,3-dioxo-6-((3,3,3-trifluoropropyl)amino)-1H-benzo[de]isoquinolin-2(3H)-yl)hexanoic acid (6 mg, 14% yield).

^{1}H NMR (400 MHz, DMSO-d6) δ 11.96 (s, 1H), 8.65 (d, J = 8.6, 1.2 Hz, 1H), 8.45 (d, J = 7.3, 1.0 Hz, 1H), 8.30 (d, J = 8.5 Hz, 1H), 7.80 (t, J = 5.6 Hz, 1H), 7.76 – 7.67 (m, 1H), 6.85 (d, J = 8.6 Hz, 1H), 4.00 (t, J = 7.4 Hz, 2H), 3.71 – 3.62 (m, 2H), 2.83 – 2.66 (m, 2H), 2.21 (t, J = 7.3 Hz, 2H), 1.67 – 1.48 (m, 4H), 1.39 – 1.27 (m, 2H).

^{13}C NMR (101 MHz, DMSO-d6) δ 174.9, 164.1, 163.3, 150.2, 134.5, 131.1, 130.0, 129.64, 128.7, 128.2, 126.3, 125.0, 124.5, 122.3, 120.6, 108.9, 104.3, 40.4, 39.5, 36.5 (q, J_{CF} = 3.8), 33.9, 31.9 (q, J_{CF} = 26.8 Hz), 27.8, 26.4, 24.6.

^{19}F NMR (376 MHz, DMSO-d6) δ -63.60.

HRMS (ESI) for $C_{21}H_{21}F_3N_2O_4$ m/z $[M+H]^+$ calcd.: 423.1526, found: 423.1528.

6-(1,3-dioxo-6-((pyrimidin-5-ylmethyl)amino)-1H-benzo[de]isoquinolin-2(3H)-yl)hexanoic acid (A19)

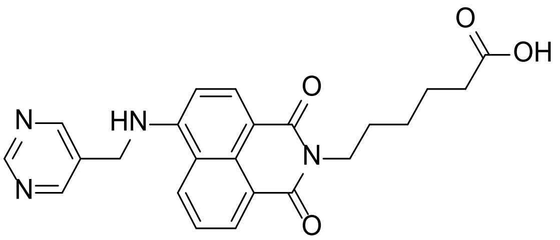

The title compound was prepared following **General Procedure B.** 6-(6-bromo-1,3-dioxo-1H-benzo[de]isoquinolin-2(3H)-yl)hexanoic acid (35 mg, 0.09 mmol) was used as the aryl halide core and pyrimidin-5-ylmethanamine (16 mg, 0.15 mmol) was used as the amine building block. The crude residue was purified by preparative reverse-phase HPLC (mass triggered collection) to afford the title compound 6-(1,3-dioxo-6-((pyrimidin-5-ylmethyl)amino)-1H-benzo[de]isoquinolin-2(3H)-yl)hexanoic acid (5 mg, 12% yield).

^{1}H NMR (400 MHz, DMSO-d6) δ 9.36 (s, 1H), 9.20 (s, 1H), 8.91 (s, 2H), 8.69 (d, J = 7.7 Hz, 1H), 8.61 – 8.51 (m, 2H), 8.13 (d, J = 7.7 Hz, 1H), 7.96 – 7.87 (m, 1H), 6.79 (s, 1H), 4.05 (t, J = 7.4 Hz, 2H), 2.22 (t, J = 7.3 Hz, 2H), 1.71 – 1.49 (m, 4H), 1.41 – 1.30 (m, 2H).

[Note: 2 alkyl protons not visible under the water peak]

^{13}C NMR (101 MHz, DMSO-d6) δ 174.8, 163.6, 163.4, 159.0, 157.4, 138.8, 131.4, 131.3, 130.6, 130.2, 128.5, 128.42, 125.5, 123.4, 123.3, 51.6, 50.1, 40.4, 33.8, 27.6, 26.3, 24.6.

HRMS (ESI) for, $C_{23}H_{23}N_4O_4$ m/z $[M+H]^+$ calcd.: 419.1714, found: 419.1713.

6-(6-(((1-isopropyl-1H-pyrazol-5-yl)methyl)amino)-1,3-dioxo-1H-benzo[de]isoquinolin-2(3H)-yl)hexanoic acid (A20)

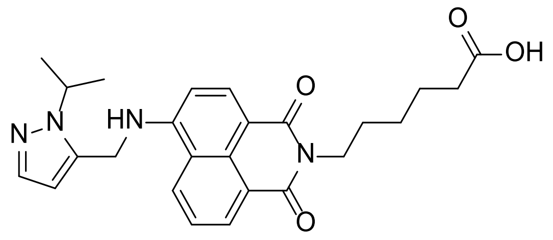

The title compound was prepared following **General Procedure B.** 6-(6-bromo-1,3-dioxo-1H-benzo[de]isoquinolin-2(3H)-yl)hexanoic acid (35 mg, 0.09 mmol) was used as the aryl halide core and 2-isopropylpyrazol-3-yl)methanamine (21 mg, 0.15 mmol) was used as the amine building block. The crude residue was purified by preparative reverse-phase HPLC (mass triggered collection) to afford the title compound 6-(6-(((1-isopropyl-1H-pyrazol-5-yl)methyl)amino)-1,3-dioxo-1H-benzo[de]isoquinolin-2(3H)-yl)hexanoic acid (10 mg, 24% yield).

^{1}H NMR (400 MHz, DMSO-d6) δ 8.73 (d, J = 8.5, 1.1 Hz, 1H), 8.45 (d, J = 7.3, 1.0 Hz, 1H), 8.28 – 8.19 (m, 2H), 7.76 – 7.68 (m, 1H), 7.37 (d, J = 1.8 Hz, 1H), 6.88 (d, J = 8.6 Hz, 1H), 6.23 (d, J = 1.8 Hz, 1H), 4.81 – 4.67 (m, 3H), 4.02 – 3.97 (m, 2H), 2.21 (t, J = 7.3 Hz, 2H), 1.66 – 1.48 (m, 4H), 1.37 (d, J = 6.5 Hz, 6H), 1.35 – 1.27 (m, 2H).

^{13}C NMR (101 MHz, DMSO-d6) δ 174.0, 164.2, 163.4, 150.4, 137.9, 137.7, 134.3, 131.2, 129.7, 128.9, 125.1, 122.4, 120.8, 109.1, 105.9, 105.1, 38.1, 33.9, 27.8, 26.5, 24.7, 23.1.

HRMS (ESI) for $C_{25}H_{28}N_4O_4$, m/z $[M+H]^+$ calcd.: 449.2183, found: 449.2187.

6-(6-((isoquinolin-5-ylmethyl)amino)-1,3-dioxo-1H-benzo[de]isoquinolin-2(3H)-yl)hexanoic acid (A21)

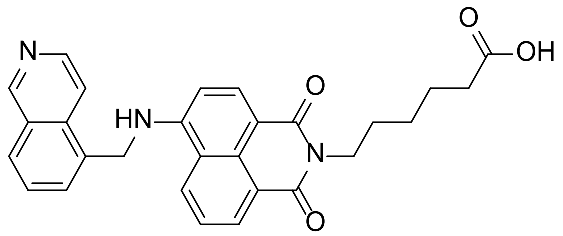

The title compound was prepared following **General Procedure B.** 6-(6-bromo-1,3-dioxo-1H-benzo[de]isoquinolin-2(3H)-yl)hexanoic acid (35 mg, 0.09 mmol) was used as the aryl halide core and 5-isoquinolylmethanamine (24 mg, 0.15 mmol) was used as the amine building block. The crude residue was purified by preparative reverse-phase HPLC (mass triggered collection) to afford the title compound -(6-((isoquinolin-5-ylmethyl)amino)-1,3-dioxo-1H-benzo[de]isoquinolin-2(3H)-yl)hexanoic acid (8 mg, 15% yield).

^{1}H NMR (400 MHz, DMSO-d6) δ 9.54 (s, 1H), 8.81 (d, J = 8.4 Hz, 1H), 8.66 (d, J = 6.2 Hz, 1H), 8.51 – 8.43 (m, 2H), 8.35 (d, J = 6.2 Hz, 1H), 8.19 (d, J = 8.4 Hz, 2H), 7.87 (d, J = 7.1 Hz, 1H), 7.79 – 7.69 (m, 2H), 6.73 (d, J = 8.6 Hz, 1H), 5.17 (d, J = 5.4 Hz, 2H), 3.99 (t, J = 7.4 Hz, 2H), 2.20 (t, J = 7.3 Hz, 2H), 1.65 – 1.47 (m, 4H), 1.38 – 1.27 (m, 2H).

^{13}C NMR (101 MHz, DMSO-d6) δ 174.5 (C), 163.7 (C), 162.9 (C), 152.9 (CH), 150.4 (C), 143.2 (CH), 134.0 (CH), 133.5 (C), 132.7 (C), 130.7 (CH), 129.3 (C), 128.7 (CH), 128.72 (C), 128.6 (CH), 127.1 (CH), 127.0 (CH), 124.6 (CH), 121.9 (C), 120.3 (C), 116.6 (CH), 108.4 (C), 104.6 (CH), 43.6 (CH_2), 39.0 (CH_2), 33.6 (CH_2), 27.4 (CH_2), 26.1 (CH_2), 24.3 (CH_2).

HRMS (ESI) for $C_{28}H_{26}N_3O_4$ m/z $[M+H]^+$ calcd.: 468.1918, found: 468.1918.

6-(6-((3-(difluoromethoxy)phenyl)amino)-1,3-dioxo-1H-benzo[de]isoquinolin-2(3H)-yl)hexanoic acid (A22)

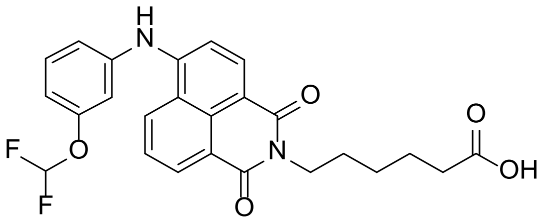

The title compound was prepared following **General Procedure A.** 6-(6-bromo-1,3-dioxo-1H-benzo[de]isoquinolin-2(3H)-yl)hexanoic acid (39 mg, 0.1 mmol) was used as the aryl halide core and 3-(difluoromethoxy)aniline (24 mg, 0.15 mmol) was used as the aniline building block. The crude residue was purified by preparative reverse-phase HPLC (mass triggered collection) to afford the title compound 6-(6-((3-(difluoromethoxy)phenyl)amino)-1,3-dioxo-1H-benzo[de]isoquinolin-2(3H)-yl)hexanoic acid (13 mg, 28% yield).

^{1}H NMR (400 MHz, DMSO-d6) δ 8.78 (d, J = 8.5 Hz, 1H), 8.52 (d, J = 7.2 Hz, 1H), 8.32 (d, J = 8.4 Hz, 1H), 7.82 (t, 1H), 7.53 – 7.05 (m, 5H), 6.94 (dd, J = 8.1, 2.4 Hz, 1H), 4.02 (t, J = 7.4 Hz, 2H), 2.19 (t, J = 7.3 Hz, 2H), 1.68 – 1.48 (m, 4H), 1.39 – 1.27 (m, 2H).

^{13}C NMR (101 MHz, DMSO-d6) δ 175.1, 164.0, 163.2, 152.3 (J_{CF} = 3.2 Hz), 147.1, 142.9, 133.5, 131.4, 129.7, 129.4, 125.7, 122.5, 119.4, 118.4, 116.8, 114.3, 113.8, 112.6, 112.1, 109.5, 49.1, 39.7, 34.2, 27.8, 26.6, 24.8.

HRMS (ESI) for $C_{25}H_{23}F_2N_2O_5$, m/z $[M+H]^+$ calcd.: 469.1569, found: 469.1569.

6-(1,3-dioxo-6-(m-tolylamino)-1H-benzo[de]isoquinolin-2(3H)-yl)hexanoic acid (A23)

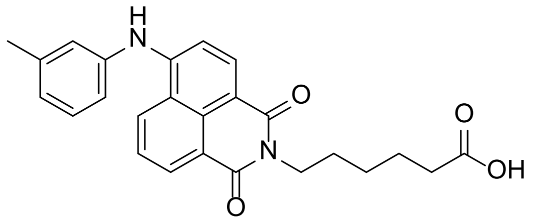

The title compound was prepared following **General Procedure A.** 6-(6-bromo-1,3-dioxo-1H-benzo[de]isoquinolin-2(3H)-yl)hexanoic acid (39 mg, 0.1 mmol) was used as the aryl halide core and 3-methylaniline (16.1 mg, 0.15 mmol) was used as the aniline building block. The crude residue was purified by preparative reverse-phase HPLC (mass triggered collection) to afford the title compound 6-(1,3-dioxo-6-(m-tolylamino)-1H-benzo[de]isoquinolin-2(3H)-yl)hexanoic acid (14 mg, 33% yield).

^{1}H NMR (400 MHz, DMSO-d6) δ 8.82 (d, J = 8.4 Hz, 1H), 8.50 (d, J = 7.3 Hz, 1H), 8.27 (d, J = 8.5 Hz, 1H), 7.83 – 7.75 (m, 1H), 7.34 (t, J = 7.7 Hz, 1H), 7.27 – 7.16 (m, 3H), 7.01 (d, J = 7.5 Hz, 1H), 4.01 (t, J = 7.4 Hz, 2H), 2.35 (s, 3H), 2.16 (t, J = 7.3 Hz, 2H), 1.67 – 1.47 (m, 4H), 1.39 – 1.26 (m, 2H).

^{13}C NMR (101 MHz, DMSO-d6) δ 174.4 (C), 163.6 (C), 162.8 (C), 148.0 (C), 140.2 (C), 138.9 (C), 133.4 (CH), 130.9 (CH), 129.4 (C), 129.3 (CH), 128.9 (CH), 125.0 (CH), 124.9 (C), 123.3 (CH), 122.0 (C), 121.5 (C), 119.8 (CH), 110.9 (C), 107.7 (CH), 39.1 (CH_2), 33.5 (CH_2), 27.4 (CH_2), 26.1 (CH_2), 24.2 (CH_2), 21.1 (CH_3).

HRMS (ESI) for $C_{25}H_{24}N_2O_4$ m/z $[M+H]^+$ calcd.: 417.1809, found: 417.1810.

6-(6-((3,4-dichlorophenyl)amino)-1,3-dioxo-1H-benzo[de]isoquinolin-2(3H)-yl)hexanoic acid (A24)

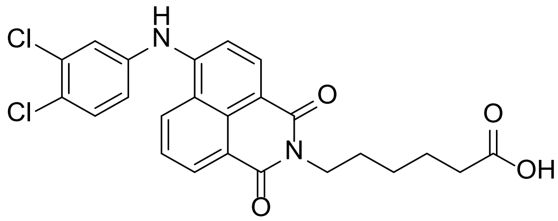

The title compound was prepared following **General Procedure A.** 6-(6-bromo-1,3-dioxo-1H-benzo[de]isoquinolin-2(3H)-yl)hexanoic acid (39 mg, 0.1 mmol) was used as the aryl halide core and 3,4-dichloroaniline (24 mg, 0.15 mmol) was used as the aniline building block. The crude residue was purified by preparative reverse-phase HPLC (mass triggered collection) to afford the title compound 6-(6-((3,4-dichlorophenyl)amino)-1,3-dioxo-1H-benzo[de]isoquinolin-2(3H)-yl)hexanoic acid (8 mg, 17% yield).

^{1}H NMR (400 MHz, DMSO-d6) δ 9.47 (s, 1H), 8.74 (d, J = 8.4 Hz, 1H), 8.53 (d, J = 7.2 Hz, 1H), 8.34 (d, J = 8.4 Hz, 1H), 7.84 (t, J = 7.9 Hz, 1H), 7.64 (d, J = 8.7 Hz, 1H), 7.58 (d, J = 2.6 Hz, 1H), 7.44 (d, J = 8.4 Hz, 1H), 7.38 (dd, J = 8.7, 2.6 Hz, 1H), 4.02 (t, J = 7.4 Hz, 2H), 2.20 (t, J = 7.3 Hz, 2H), 1.68 – 1.48 (m, 4H), 1.40 – 1.29 (m, 2H).

^{13}C NMR (101 MHz, DMSO-d6) δ 174.9, 164.0, 163.2, 146.5, 141.8, 133.4, 132.1, 131.6, 131.5, 129.7, 129.3, 126.0, 124.9, 122.8, 122.5, 121.5, 113.3, 110.3, 39.6, 34.0, 27.7, 26.4, 24.6.

HRMS (ESI) for, m/z $[M+H]^+$ calcd.: 471.0873, found: 471.0872.

6-(1,3-dioxo-6-(p-tolylamino)-1H-benzo[de]isoquinolin-2(3H)-yl)hexanoic acid (A25)

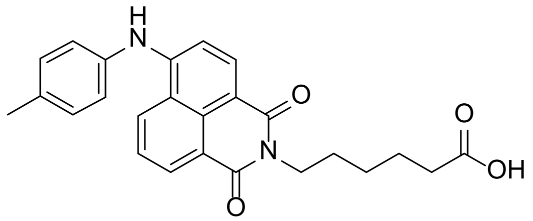

The title compound was prepared following **General Procedure A.** 6-(6-bromo-1,3-dioxo-1H-benzo[de]isoquinolin-2(3H)-yl)hexanoic acid (39 mg, 0.1 mmol) was used as the aryl halide core and 4-methylaniline (16 mg, 0.15 mmol) was used as the aniline building block. The crude residue was purified by preparative reverse-phase HPLC (mass triggered collection) to afford the title compound 6-(1,3-dioxo-6-(p-tolylamino)-1H-benzo[de]isoquinolin-2(3H)-yl)hexanoic acid (13 mg, 32% yield).

^{1}H NMR (400 MHz, DMSO-d6) δ 9.34 (s, 1H), 8.83 (d, J = 8.5 Hz, 1H), 8.50 (d, J = 7.1 Hz, 1H), 8.25 (d, J = 8.5 Hz, 1H), 7.82 – 7.74 (m, 1H), 7.34 – 7.23 (m, 4H), 7.12 (d, J = 8.5 Hz, 1H), 4.01 (t, J = 7.4 Hz, 2H), 2.34 (s, 3H), 2.20 (t, J = 7.3 Hz, 2H), 1.67 – 1.48 (m, 4H), 1.39 – 1.27 (m, 2H).

^{13}C NMR (101 MHz, DMSO-d6) δ 174.9, 164.1, 163.2, 148.9, 137.9, 134.2, 133.9, 131.4, 130.5, 129.9, 129.3, 125.3, 123.8, 122.4, 121.6, 110.8, 107.4, 39.6, 34.1, 27.8, 26.6, 24.7, 21.0.

HRMS (ESI) for $C_{25}H_{25}N_2O_4$, m/z $[M+H]^+$ calcd.: 417.1809, found: 417.1808.

6-(6-((3-chlorophenyl)amino)-1,3-dioxo-1H-benzo[de]isoquinolin-2(3H)-yl)hexanoic acid (A26)

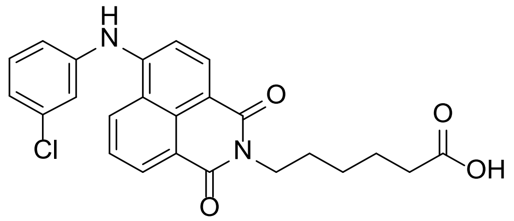

The title compound was prepared following **General Procedure A.** 6-(6-bromo-1,3-dioxo-1H-benzo[de]isoquinolin-2(3H)-yl)hexanoic acid (39 mg, 0.1 mmol) was used as the aryl halide core and 3-chloroaniline (19 mg, 0.15 mmol) was used as the aniline building block. The crude residue was purified by preparative reverse-phase HPLC (mass triggered collection) to afford the title compound 6-(6-((3-chlorophenyl)amino)-1,3-dioxo-1H-benzo[de]isoquinolin-2(3H)-yl)hexanoic acid (14 mg, 32% yield).

^{1}H NMR (400 MHz, DMSO-d6) δ 8.76 (d, J = 8.4 Hz, 1H), 8.51 (d, J = 7.2 Hz, 1H), 8.33 (d, J = 8.4 Hz, 1H), 7.86 – 7.78 (m, 1H), 7.48 – 7.33 (m, 4H), 7.22 – 7.14 (m, 1H), 4.02 (t, J = 7.4 Hz, 2H), 2.19 (t, J = 7.3 Hz, 2H), 1.68 – 1.48 (m, 4H), 1.39 – 1.27 (m, 2H).

^{13}C NMR (101 MHz, DMSO-d6) δ 175.0, 163.9, 163.1, 146.9, 143.0, 134.2, 133.5, 131.4, 129.7, 129.3, 125.7, 123.5, 122.6, 122.5, 121.5, 120.2, 112.8, 109.5, 39.7, 34.1, 27.8, 26.6, 24.7.

HRMS (ESI) for $C_{24}H_{22}ClN_2O_4$, m/z $[M+H]^+$ calcd.: 437.1263, found: 437.1262.

tert-Butyl 6-(6-bromo-1,3-dioxo-benzo[de]isoquinolin-2-yl)hexanoate (compound 4)

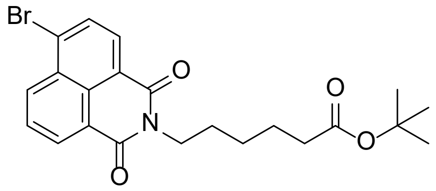

4-Bromo-1,8-naphthalic anhydride (1.50 g, 5.16 mmol, 1 equiv.) and tert-butyl 6-aminohexanoate (0.99 g, 5.15 mmol, 1 equiv.) were suspended in DMSO (10 mL). The brown suspension was heated to 110 °C under nitrogen for 16 h. The resulting dark solution was cooled to r.t., poured into water (75 mL), stirred for 30 min and extracted with MTBE (3 x 50 mL). The combined organics were washed with sat. aq. NaCl (25 mL), dried over Na_2SO_4, and the solvents were removed under reduced pressure. The title compound tert-butyl 6-(6-bromo-1,3-dioxo-benzo[de]isoquinolin-2-yl)hexanoate (2.41 g, 5.4 mmol) was isolated. NMR analysis indicated that the product was ~90% pure with a major naphthalene-related impurity. This material was used without further purification.

^{1}H NMR (600 MHz, DMSO-d6) δ 8.48 – 8.43 (m, 1H), 8.43 – 8.37 (m, 1H), 8.20 (d, J = 7.6 Hz, 1H), 8.10 (dd, J = 8.3, 4.1 Hz, 1H), 7.89 (dd, J = 9.5, 6.2 Hz, 1H), 3.97 (t, J = 7.4 Hz, 2H), 2.17 (t, J = 7.3, 2H), 1.66 – 1.60 (m, 2H), 1.57 – 1.51 (m, 2H), 1.41 – 1.26 (m, 11H).

tert-Butyl 6-(6-amino-1,3-dioxo-1H-benzo[de]isoquinolin-2(3H)-yl)hexanoate (compound 5)

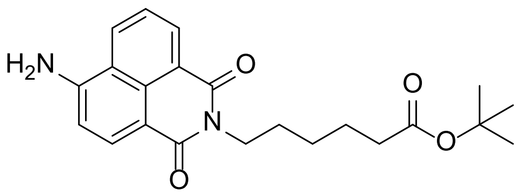

tert-Butyl 6-aminohexanoate (0.97 g, 5.16 mmol, 1 equiv.) was added to a solution of 6-aminobenzo[de]isochromene-1,3-dione (1.0 g, 4.69 mmol, 10 equiv.) in DMSO (10 mL). The resulting mixture was stirred at 110 °C for 12 h. The reaction mixture was diluted with water (50 mL) and extracted with EtOAc (50 mL x 3). The combined organic layers were washed with brine (50 mL x 3), dried over Na_2SO_4, filtered, and concentrated under reduced pressure. The residue was triturated with EtOAc (50 mL) at 25 °C for 30 min to give the title compound tert-butyl 6-(6-amino-1,3-dioxo-1H-benzo[de]isoquinolin-2(3H)-yl)hexanoate (1.6 g, 85% yield).

^{1}H NMR (400 MHz, DMSO-d6) δ 8.60 (dd, J = 8.4, 1.2 Hz, 1H), 8.41 (dd, J = 7.3, 1.1 Hz, 1H), 8.18 (d, J = 8.4 Hz, 1H), 7.64 (dd, J = 8.4, 7.3 Hz, 1H), 7.41 (s, 2H), 6.84 (d, J = 8.4 Hz, 1H), 3.99 (t, J = 7.3 Hz, 2H), 2.17 (t, J = 7.2 Hz, 2H), 1.64 – 1.60 (m, 2H), 1.55 – 1.48 (m, 2H), 1.41 – 1.35 (m, 2H), 1.34 (s, 9H).

***tert*-Butyl 6-[6-[(6-morpholino-3-pyridyl)methylamino]-1,3-dioxo-benzo[de]isoquinolin-2-yl]hexanoate (A17(tBu))**

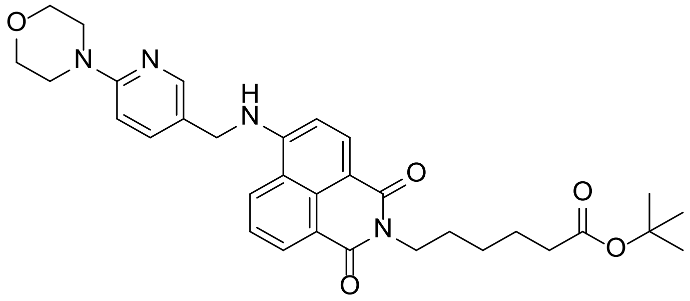

A 20 mL vial was charged with *tert*-butyl 6-(6-bromo-1,3-dioxo-benzo[de]isoquinolin-2-yl)hexanoate (0.30 g, 0.68 mmol, 1 equiv.), (6-morpholino-3-pyridyl)methanamine (0.16 g, 0.82 mmol, 1.2 equiv.), Cs_2CO_3 (0.44 g, 1.36 mmol, 2.0 equiv.), and *tert*-amyl alcohol (6 mL). The mixture was degassed by sparging with argon, then [dicyclohexyl(2′,6′-diisopropoxy-2-biphenylyl)phosphine-κP](methanesulfonatato-κO)[2′-(methylamino-κN)-2-biphenylyl-κC2]palladium (Ruphos Pd G4) (0.06 g, 0.07 mmol, 0.1 equiv.) was added and the resulting mixture was heated at 90 °C overnight. The reaction was cooled to r.t., diluted with water (10 mL), 1 N aq. HCl (2 mL) was added, and the mixture was extracted with ethyl acetate (3 x 20 mL). The organic layers were combined, and solvent was removed under reduced pressure. Purification by reverse-phase preparative HPLC gave the product tert-butyl 6-[6-[(6-morpholino-3-pyridyl)methylamino]-1,3-dioxo-benzo[de]isoquinolin-2-yl]hexanoate (0.34 g, 89% yield).

^{1}H NMR (400 MHz, $CDCl_3$) δ 8.32 (dd, J = 7.2, 3.9 Hz, 1H), 8.23 – 8.07 (m, 3H), 7.74 (dd, J = 9.2, 2.2 Hz, 1H), 7.41 – 7.32 (m, 1H), 6.92 (br s, 1H), 6.77 (d, J = 9.3 Hz, 1H), 6.39 (dd, J = 8.2, 3.8 Hz, 1H), 4.35 (s, 2H), 3.96 (t, J = 7.5 Hz, 2H), 3.67 (t, J = 4.8 Hz, 4H), 3.46 (t, J = 4.9 Hz, 4H), 2.06 (t, J = 7.4 Hz, 2H), 1.67 – 1.48 (m, 4H), 1.33 – 1.21 (m, 2H), 1.27 (s, 9H).

^{13}C NMR (101 MHz, $CDCl_3$) δ 173.4 (C), 164.6 (C), 164.0 (C), 153.2 (C), 148.9 (C), 143.2 (CH), 138.4 (CH), 134.0 (CH), 131.3 (CH), 129.7 (C), 127.0 (CH), 125.1 (CH), 123.8 (C), 122.9 (C), 120.7 (C), 111.4 (C), 111.2 (CH), 104.7 (CH), 80.2 (C), 65.9 (CH_2 x 2), 46.4 (CH_2 x 2), 43.9 (CH_2), 40.1 (CH_2), 35.6 (CH_2), 28.2 (CH_3 x 3), 28.0 (CH_2), 26.8 (CH_2), 25.0 (CH_2).

***tert*-Butyl 6-[6-[methoxycarbonyl-[(6-morpholino-3-pyridyl)methyl]amino]-1,3-dioxo-benzo[de]isoquinolin-2-yl]hexanoate (A17-C(tBu))**

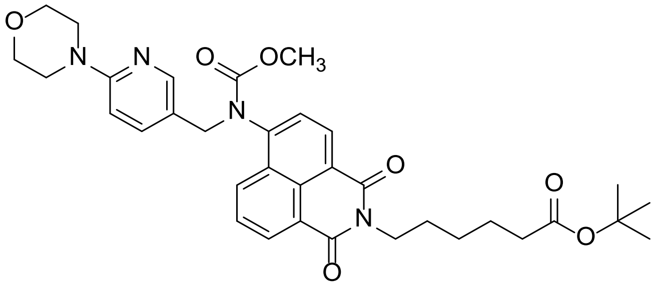

A 30 mL vial was charged with *tert*-butyl 6-(6-(((6-morpholinopyridin-3-yl)methyl)amino)-1,3-dioxo-1H-benzo[de]isoquinolin-2(3H)-yl)hexanoate (0.31 g, 0.55 mmol, 1.0 equiv.), THF (3 mL) and sodium hydride (0.18 g, 4.4 mmol, 8.0 equiv.). Within 10 min, the reaction mixture became red with stirring at r.t.. Methyl chloroformate (0.21 mL, 2.73 mmol, 5.0 equiv.) was added and the resulting yellow mixture was stirred for 2 days. The reaction was quenched with ice water (50 mL) and 2 N aq. citric acid (5 mL), extracted with EtOAc (2 x 50 mL), and the combined organic layers were concentrated under reduced pressure. Purification by reverse-phase preparative HPLC gave the product tert-butyl 6-[6-[methoxycarbonyl-[(6-morpholino-3-pyridyl)methyl]amino]-1,3-dioxo-benzo[de]isoquinolin-2-yl]hexanoate (60 mg, 17% yield).

^{1}H NMR (500 MHz, DMSO-d6) δ 8.50 (dd, J = 7.2, 1.1 Hz, 1H), 8.46 (d, J = 7.8 Hz, 1H), 8.20 – 8.08 (m, 1H), 7.92 – 7.77 (m, 2H), 7.62 (dd, J = 8.5, 5.5 Hz, 2H), 6.89 (d, J = 9.0 Hz, 1H), 5.02 (d, J = 15.0 Hz, 1H), 4.75 (d, J = 14.9 Hz, 1H), 4.03 (t, J = 7.3 Hz, 2H), 3.69 – 3.61 (m, 4H), 3.55 – 3.48 (m, 3H), 3.41 (t, J = 4.9 Hz, 4H), 2.17 (t, J = 7.2 Hz, 2H), 1.66 – 1.59 (m, 2H), 1.56 – 1.49 (m, 2H), 1.33 (s, 9H), 1.41 – 1.23 (m, 2H).

6-[6-[Methoxycarbonyl-[(6-morpholino-3-pyridyl)methyl]amino]-1,3-dioxo-benzo[de]isoquinolin-2-yl]hexanoic acid (A17-C)

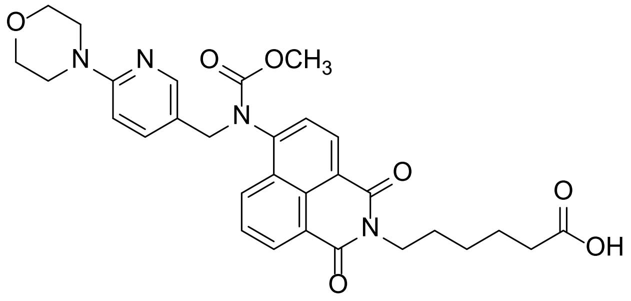

Trifluoroacetic acid (0.14 mL, 1.88 mmol, 20.0 eq) was added to a r.t. solution of *tert*-butyl 6-[6-[methoxycarbonyl-[(6-morpholino-3-pyridyl)methyl]amino]-1,3-dioxo-benzo[de]isoquinolin-2-yl]hexanoate (0.06 g, 0.09 mmol, 1.0 equiv.) in DCM (5 mL). After 4 h, solvents were removed under reduced pressure. Purification by reverse-phase preparative HPLC gave the product 6-[6-[methoxycarbonyl-[(6-morpholino-3-pyridyl)methyl]amino]-1,3-dioxo-benzo[de]isoquinolin-2-yl]hexanoic acid (30 mg, 59% yield).

^{1}H NMR (400 MHz, DMSO-d6, 90°C) δ 11.45 (br s, 1H), 8.49 (d, J = 7.2 Hz, 1H), 8.46 (d, J = 7.7 Hz, 1H), 8.10 (d, J = 8.4 Hz, 1H), 7.88 (d, J = 2.4 Hz, 1H), 7.83 (t, J = 7.9 Hz, 1H), 7.60 (d, J = 7.8 Hz, 1H), 7.44 (dd, J = 8.8, 2.4 Hz, 1H), 6.69 (d, J = 8.8 Hz, 1H), 4.99 – 4.95 (m, 1H), 4.78 – 4.73 (m, 1H), 4.04 (t, J = 7.4 Hz, 2H), 3.64 (t, J = 4.8 Hz, 4H), 3.59 (s, 3H), 3.36 (t, J = 4.9 Hz, 4H), 2.21 (t, J = 7.3 Hz, 2H), 1.68 – 1.60 (m, 2H), 1.59 – 1.53 (m, 2H), 1.43 – 1.31 (m, 2H).

^{13}C NMR (101 MHz, DMSO-d6, 90°C) δ 173.5 (C), 162.8 (C), 162.4 (C), 158.2 (C), 155.0 (C), 147.2 (CH), 143.3 (C), 137.7 (CH), 130.3 (CH), 130.2 (CH), 128.8 (CH), 128.6 (C), 128.1 (C), 127.0 (CH), 126.6 (CH), 122.4 (C), 121.2 (C), 121.1 (C), 106.1 (CH), 65.4 (CH_2 x 2), 52.4 (CH_3), 51.0 (CH_2), 44.8 (CH_2 x 2), 39.1 (CH_2), 33.1 (CH_2), 26.8 (CH_2), 25.6 (CH_2), 23.8 (CH_2).

HRMS (ESI) for $C_{30}H_{32}N_4O_7$ m/z $[M+H]^+$ calcd.: 561.2344, found: 561.2348.

tert-Butyl 6-[6-(5-isoquinolylmethylamino)-1,3-dioxo-benzo[de]isoquinolin-2-yl]hexanoate (A21(tBu))

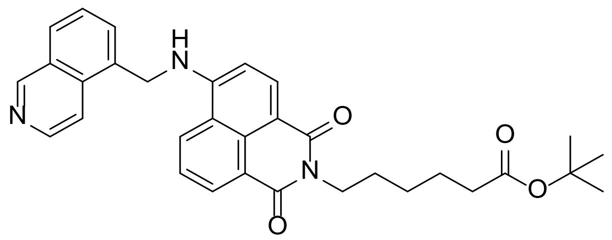

A 20 mL vial was charged with tert-butyl 6-(6-bromo-1,3-dioxo-benzo[de]isoquinolin-2-yl)hexanoate (0.28 g, 0.62 mmol, 1 equiv.), 5-isoquinolylmethanamine (0.15 g, 0.93 mmol, 1.5 equiv.), Cs_2CO_3 (0.40 mg, 1.24 mmol, 2 equiv.), and tert-amyl alcohol (8 mL). The reaction mixture was degassed by sparging with argon, then [dicyclohexyl(2′,6′-diisopropoxy-2-biphenylyl)phosphine-κP](methanesulfonatato-κO)[2′-(methylamino-κN)-2-biphenylyl-κC2]palladium (Ruphos Pd G4) (0.05 mg, 0.06 mmol, 0.1 equiv.) was added and the resulting mixture was sealed and heated at 90°C overnight. The reaction was cooled to r.t., diluted with water (10 mL), 1 N aq. HCl (2 mL) was added, and the mixture was extracted with EtOAc (3 x 20 mL). The organic layers were combined, and solvent was removed under reduced pressure. Purification by reverse-phase preparative HPLC gave the product tert-butyl 6-[6-(5-isoquinolylmethylamino)-1,3-dioxo-benzo[de]isoquinolin-2-yl]hexanoate (0.24 g, 73% yield).

^{1}H NMR (600 MHz, DMSO-d6) δ 9.66 (s, 1H), 8.80 (dd, J = 8.5, 1.1 Hz, 1H), 8.70 (d, J = 6.4 Hz, 1H), 8.50 – 8.44 (m, 3H), 8.27 (d, J = 8.2 Hz, 1H), 8.18 (d, J = 8.5 Hz, 1H), 7.95 (dd, J = 7.2, 1.2 Hz, 1H), 7.79 (dd, J = 8.2, 7.2 Hz, 1H), 7.74 (dd, J = 8.4, 7.3 Hz, 1H), 6.72 (d, J = 8.6 Hz, 1H), 5.18 (d, J = 5.4 Hz, 2H), 3.99 (d, J = 14.6 Hz, 1H), 2.16 (t, J = 7.2 Hz, 2H), 1.63 – 1.55 (m, 2H), 1.57 – 1.46 (m, 2H), 1.32 (s, 9H), 1.31 – 1.25 (m, 2H).

tert-Butyl 6-[6-[5-isoquinolylmethyl(methoxycarbonyl)amino]-1,3-dioxo-benzo[de]isoquinolin-2-yl]hexanoate (A21-C(tBu))

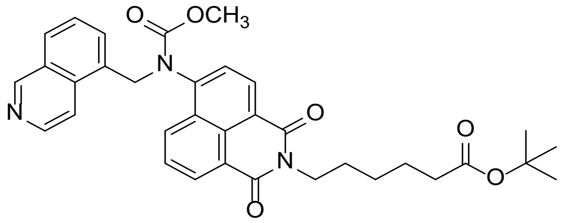

Sodium hydride (0.046 g, 1.15 mmol, 6 equiv.) was added to a solution of tert-butyl 6-[6-(5-isoquinolylmethylamino)-1,3-dioxo-benzo[de]isoquinolin-2-yl]hexanoate (0.10 g, 0.19 mmol, 1 equiv.) in THF (6 mL) at 0°C. After 10 min, the cooling bath was removed and the reaction was stirred at r.t. for 2 h. The reaction was then cooled to 0°C and treated with methyl chloroformate (0.15 mL, 1.91 mmol, 10 equiv.) to give a yellow mixture that was stirred at r.t. for 2 days. The reaction mixture was quenched with ice water (50 mL) and 2 N citric acid (5 mL), extracted with EtOAc (2 x 50 mL), and the combined organic layers were concentrated under reduced pressure. Purification by reverse-phase preparative HPLC gave an enriched mixture of product and unreacted starting material (25 mg, 23% yield). The material was used without further purification.

6-[6-[5-Isoquinolylmethyl(methoxycarbonyl)amino]-1,3-dioxo-benzo[de]isoquinolin-2-yl]hexanoic acid (A21-C)

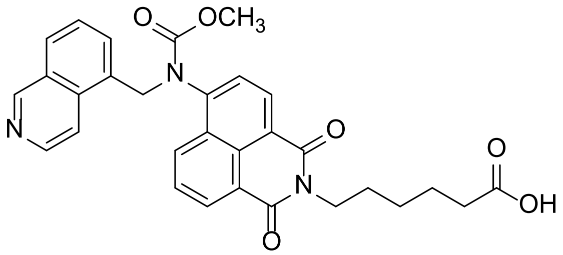

Trifluoroacetic acid (0.19 mL, 2.41 mmol, 50 equiv.) was added to a solution of tert-butyl 6-[6-[5-isoquinolylmethyl(methoxycarbonyl)amino]-1,3-dioxo-benzo[de]isoquinolin-2-yl]hexanoate (0.03 g, 0.05 mmol, 1 equiv.) in DCM (5 mL). After 4 h, volatiles were removed under reduced pressure. Purification by reverse-phase preparative HPLC gave the product 6-[6-[5-Isoquinolylmethyl(methoxycarbonyl)amino]-1,3-dioxo-benzo[de]isoquinolin-2-yl]hexanoic acid as (15 mg, 59% yield).

^{1}H NMR (400 MHz, DMSO-d6, 90°C) δ 11.77 (s, 1H), 9.24 (s, 1H), 8.50 (d, J = 6.0 Hz, 1H), 8.38 (t, J = 7.7 Hz, 2H), 7.99 (d, J = 6.0 Hz, 1H), 7.93 (t, J = 7.6 Hz, 2H), 7.64 (t, J = 8.0 Hz, 2H), 7.47 (d, J = 7.0 Hz, 1H), 7.42 (t, J = 7.5 Hz, 1H), 5.55 – 5.49 (m, 2H), 4.00 (t, J = 7.4 Hz, 2H), 3.63 (s, 3H), 2.21 (t, J = 7.3 Hz, 2H), 1.65 – 1.57 (m, 2H), 1.56 – 1.48 (m, 2H), 1.41 – 1.29 (m, 2H).

^{13}C NMR (101 MHz, DMSO-d6, 90°C) δ 173.5 (C), 162.7 (C), 162.3 (C), 155.0 (C), 152.4 (CH), 142.8 (CH), 142.8 (C), 133.4 (C), 131.4 (CH), 131.3 (C), 130.2 (CH), 130.1 (CH), 128.6 (C), 128.5 (CH), 128.0 (C), 127.9 (C), 127.4 (CH), 126.8 (CH), 126.5 (CH), 126.1 (CH), 122.3 (C), 121.2 (C), 115.8 (CH), 52.7 (CH_3), 50.3 (CH_2), 39.1 (CH_2), 33.1 (CH_2), 26.7 (CH_2), 25.6 (CH_2), 23.8 (CH_2).

HRMS (ESI) for $C_{30}H_{27}N_3O_6$ m/z $[M+H]^+$ calcd.: 526.1973, found: 526.1976.

(S)-2-Acetamido-*N*-((S)-1-((4-(hydroxymethyl)phenyl)amino)-1-oxopropan-2-yl)propenamide (compound 7)

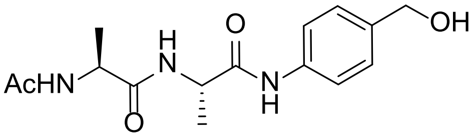

HATU (4.51 g, 11.9 mmol, 1.1 equiv.) and diisopropylethylamine (5.2 mL, 29.7 mmol, 3 equiv.) were added to a stirred solution of 4-aminobenzyl alcohol (1.5 g, 11.9 mmol, 1.1 equiv.) and Ac-Ala-Ala-OH (2 g, 9.9 mmol, 1.1 equiv.) in DMF (20 mL). The reaction mixture was stirred at 25°C for 3 h. Purification by reverse-phase preparative HPLC gave the title compound (S)-2-Acetamido-*N*-((S)-1-((4-(hydroxymethyl)phenyl)amino)-1-oxopropan-2-yl)propenamide (1.7 g, 40% yield).

^{1}H NMR (400 MHz, DMSO-d6) δ 9.78 (s, 1H), 8.09 (t, J = 7.2 Hz, 1H), 7.59 – 7.52 (m, 2H), 7.23 (d, J = 8.3 Hz, 2H), 5.10 (t, J = 5.7 Hz, 1H), 4.43 (s, 1H), 4.44 – 4.32 (m, 2H), 4.28 – 4.23 (m, 1H), 1.85 (s, 3H), 1.30 (d, J = 7.1 Hz, 3H), 1.20 (d, J = 7.2 Hz, 3H).

tert-Butyl 6-(6-((((4-((S)-2-((S)-2-acetamidopropanamido)propanamido)benzyl)oxy)carbonyl)amino)-1,3-dioxo-1H-benzo[de]isoquinolin-2(3H)-yl)hexanoate (compound 8)

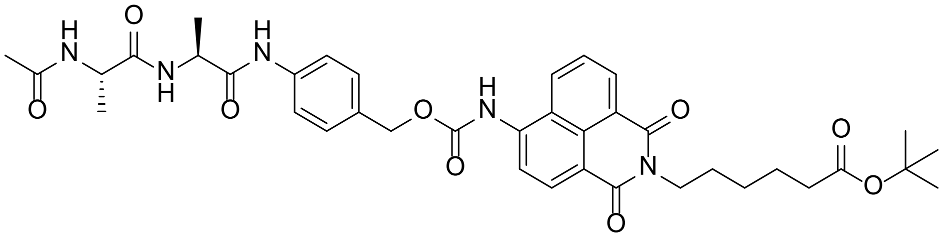

For this compound, three batches were run in parallel. Triethylamine (0.51 mL, 3.7 mmol, 2.2 equiv.) was added to a solution of tert-butyl 6-(6-amino-1,3-dioxo-1H-benzo[de]isoquinolin-2(3H)-yl)hexanoate (0.67 g, 1.7 mmol, 1 equiv.) and triphosgene (0.25 g, 0.8 mmol, 0.5 equiv.) in THF (7 mL) at 0 °C. The reaction mixture was stirred at 0 °C for 2 h. Then a solution of (S)-2-acetamido-*N*-((S)-1-((4-(hydroxymethyl)phenyl)amino)-1-oxopropan-2-yl)propanamide (0.56 g, 1.8 mmol, 1.05 equiv.) and triethylamine (0.35 mL, 2.5 mmol, 1.5 equiv.) in THF (7 mL) was added dropwise and the mixture was stirred at 25°C for 4.5 h. The reaction mixture was filtered. The filter cake was washed with EtOAc and triturated with water (50 mL x 3) at 25°C for 10 min to give the title compound tert-butyl 6-(6-((((4-((S)-2-((S)-2-acetamidopropanamido)propanamido)benzyl)oxy) carbonyl)amino)-1,3-dioxo-1H-benzo[de]isoquinolin-2(3H)-yl)hexanoate (1.5 g, 47% yield). Sample purity could be enhanced by triturating 400 mg in DMF (10 mL) at 25 °C for 30 min to give the product (0.25 g, 64% yield).

^{1}H NMR (400 MHz, DMSO-d6) δ 10.35 (s, 1H), 9.91 (s, 1H), 8.70 (d, J = 8.6 Hz, 1H), 8.49 (dd, J = 10.9, 7.8 Hz, 2H), 8.20 (d, J = 8.3 Hz, 1H), 8.11 (dd, J = 18.2, 7.1 Hz, 2H), 7.83 (t, J = 7.9 Hz, 1H), 7.67 (d, J = 8.2 Hz, 2H), 7.44 (d, J = 8.2 Hz, 2H), 5.21 (s, 2H), 4.43 – 4.35 (m, 1H), 4.30 – 4.23 (m, 1H), 4.02 (t, J = 7.3 Hz, 2H), 2.89 (s, 1H),

2.73 (s, 1H), 2.18 (t, J = 7.2 Hz, 2H), 1.85 (s, 3H), 1.64 (q, J = 7.5 Hz, 2H), 1.57 – 1.49 (m, 2H), 1.37 (s, 1H), 1.34 (s, 10H), 1.31 (d, J = 6.8 Hz, 4H), 1.20 (d, J = 7.1 Hz, 3H). **^{13}C NMR** (101 MHz, DMSO-d6) δ 172.3 (C), 172.2 (C), 171.2 (C), 169.4 (C), 163.5 (C), 162.9 (C), 162.3 (C), 154.0 (C), 140.8 (C), 139.0 (C), 131.7 (CH), 130.9 (CH), 129.3 (CH), 129.1 (CH x 2), 128.3 (C), 126.3 (CH), 123.8 (C), 122.2 (C), 119.1 (CH x 2), 118.2 (CH), 117.0 (C), 79.3 (C), 66.4 (CH_2), 49.0 (CH), 48.4 (CH), 39.3 (CH_2), 34.6 (CH_2), 27.7 (CH_3 x 3), 27.2 (CH_2), 25.8 (CH_2), 24.4 (CH_2), 22.6 (CH_3), 18.0 (CH_3), 17.9 (CH_3).

4-(5-(Chloromethyl)pyridin-2-yl)morpholine (compound 10)

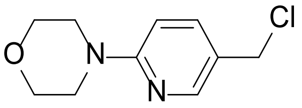

$SOCl_2$ (0.94 mL, 12.9 mmol, 5 equiv.) was added to a solution of (6-morpholinopyridin-3-yl)methanol (0.5 g, 2.6 mmol, 1 equiv.) in CH_2Cl_2 (5 mL) at 0 °C. The reaction mixture was stirred at 25 °C for 2 h. The reaction mixture was concentrated under reduced pressure to give the title compound 4-(5-(chloromethyl)pyridin-2-yl)morpholine (0.58 g), which was used in the next step without purification.

^{1}H NMR (400 MHz, DMSO-d6) δ 8.18 (s, 1 H), 8.00 (br d, J = 9.4 Hz, 1 H), 7.35 (br d, J = 9.4 Hz, 1 H), 4.79 (s, 2 H), 3.74 (br d, J = 4.3 Hz, 4 H), 3.71 (br s, 4 H).

tert-Butyl 6-(6-((((4-((S)-2-((S)-2-acetamidopropanamido)propanamido)benzyl)oxy)carbonyl)((6-morpholinopyridin-3-yl)methyl)amino)-1,3-dioxo-1H-benzo[de]isoquinolin-2(3H)-yl)hexanoate (A17-C-AA(tBu))

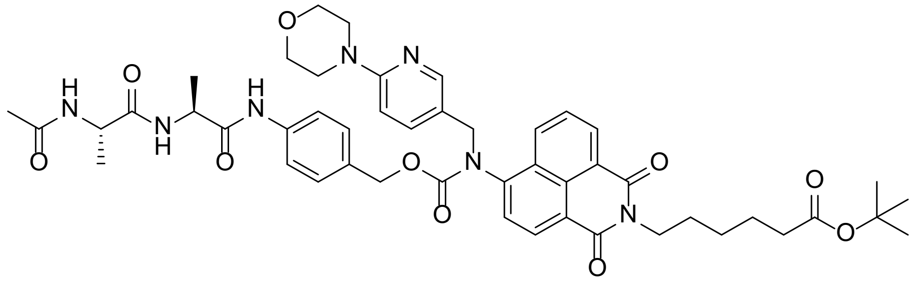

For this compound, three batches were run in parallel. Cs_2CO_3 (246 mg, 0.75 mmol, 3 equiv.) and KI (8.4 mg, 0.05 mmol, 0.2 equiv.) were added to a r.t. solution of tert-butyl 6-(6-((((4-((S)-2-((S)-2-acetamidopropanamido)propanamido)benzyl)oxy)carbonyl) amino)-1,3-dioxo-1H-benzo[de]isoquinolin-2(3H)-yl)hexanoate (200 mg, 0.25 mmol, 1 equiv.) and 4-(5-(chloromethyl)pyridin-2-yl)morpholine (81 mg, 0.38 mmol, 1.5 equiv.) in ACN (6 mL). After 12 h, the mixture was diluted with water (10 mL) and extracted with EtOAc (10 mL x 3). The combined organic layers were washed with brine (10 mL x 3), dried over Na_2SO_4, filtered, and concentrated under reduced pressure to give the title compound tert-butyl 6-(6-((((4-((S)-2-((S)-2-acetamidopropanamido)propanamido)benzyl)oxy) carbonyl)((6-morpholinopyridin-3-yl)methyl)amino)-1,3-dioxo-1H-benzo[de]isoquinolin-2(3H)-yl)hexanoate (710 mg, 74% yield), which was used in the next step without purification.

6-[6-[[4-[[(2S)-2-[[(2S)-2-Acetamidopropanoyl]amino]propanoyl]amino]phenyl]methoxycarbonyl-[(6-morpholino-3-pyridyl)methyl]amino]-1,3-dioxo-benzo[de]isoquinolin-2-yl]hexanoic acid (A17-C-AA)

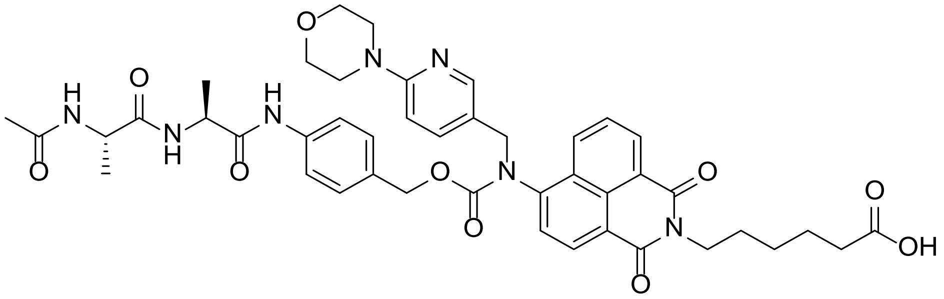

Trifluoroacetic acid (2 mL) was added to a r.t. mixture of tert-butyl 6-(6-((((4-((S)-2-((S)-2-acetamidopropanamido)propanamido)benzyl)oxy)carbonyl)((6-morpholinopyridin-3-yl)methyl)amino)-1,3-dioxo-1H-benzo[de]isoquinolin-2(3H)-yl)hexanoate (710 mg, 0.6 mmol) in CH_2Cl_2 (10 mL). After 30 min, solvent was removed under reduced pressure. Purification by reverse-phase preparative HPLC gave the title compound 6-[6-[[4-[[(2S)-2-[[(2S)-2-acetamidopropanoyl]amino]propanoyl]amino]phenyl]methoxy-carbonyl-[(6-morpholino-3-pyridyl)methyl]amino]-1,3-dioxo-benzo[de]isoquinolin-2-yl]hexanoic acid (209 mg, 43% yield).

^{1}H NMR (400 MHz, DMSO-d6, 90°C) δ 9.50 (s, 1H), 8.52 – 8.43 (m, 2H), 8.04 (dd, J = 8.5, 1.1 Hz, 1H), 7.88 (d, J = 2.4 Hz, 1H), 7.80 – 7.69 (m, 3H), 7.59 (d, J = 7.8 Hz, 1H), 7.48 (d, J = 8.3 Hz, 2H), 7.38 (dd, J = 8.7, 2.5 Hz, 1H), 7.07 (d, J = 8.2 Hz, 2H), 6.63 (d, J = 8.7 Hz, 1H), 5.06 (s, 2H), 4.88 (s, 2H), 4.45 – 4.39 (m, 1H), 4.31 – 4.25 (m, 1H), 4.10 – 4.02 (m, 2H), 3.68 – 3.61 (m, 4H), 3.41 – 3.34 (m, 4H), 2.22 (t, J = 7.3 Hz, 2H), 1.89 (s, 3H), 1.73 – 1.63 (m, 2H), 1.65 – 1.55 (m, 2H), 1.50 – 1.38 (m, 2H), 1.33 (d, J = 7.0 Hz, 3H), 1.25 (d, J = 7.1 Hz, 3H).

^{13}C NMR (101 MHz, DMSO-d6, 90°C) δ 173.5 (C), 171.7 (C), 170.5 (C), 169.0 (C), 162.8 (C), 162.4 (C), 158.3 (C), 154.4 (C), 147.4 (CH), 143.2 (C), 138.2 (C), 137.6 (CH), 130.7 (C), 130.3 (CH), 130.25 (CH),128.9 (CH), 128.5 (C), 128.1 (C), 127.7 (CH x 2), 126.9 (CH), 126.6 (CH), 122.4 (C), 121.2 (C), 121.1 (C), 119.0 (CH x 2), 106.0 (CH), 66.5 (CH_2), 65.4 (CH_2 x 2), 51.1 (CH_2), 48.7 (CH), 48.3 (CH), 44.8 (CH_2 x 2), 39.1 (CH_2), 33.1 (CH_2), 26.8 (CH_2), 25.6 (CH_2), 23.8 (CH_2), 22.0 (CH_3), 17.4 (CH_3), 17.2 (CH_3).

HRMS (ESI) for $C_{44}H_{49}N_7O_{10}$ m/z $[M+H]^+$ calcd.: 836.3614, found: 836.3624.

3. Supplementary Figures

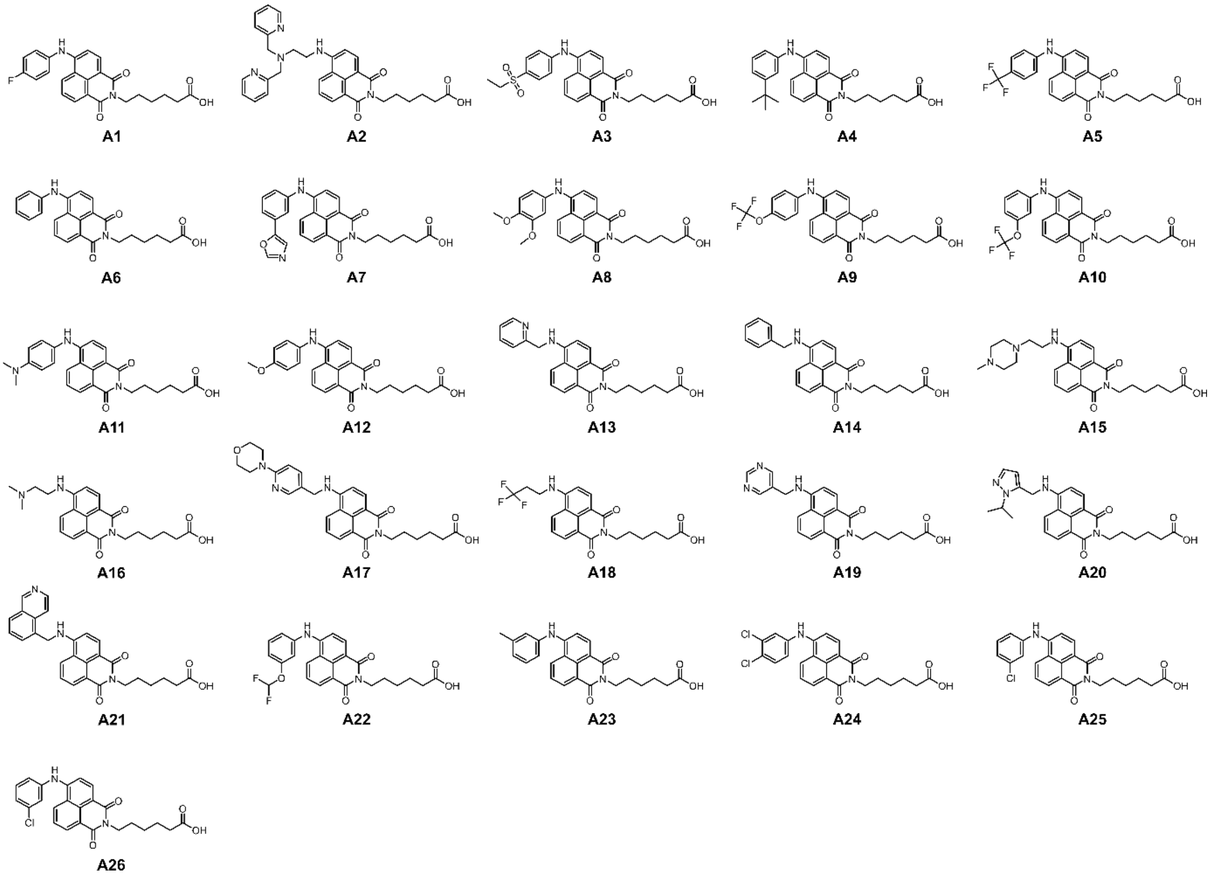

Figure S1. Chemical structures of the combinatorial library of naphthalimide fluorophores (**A1**-**A26**).

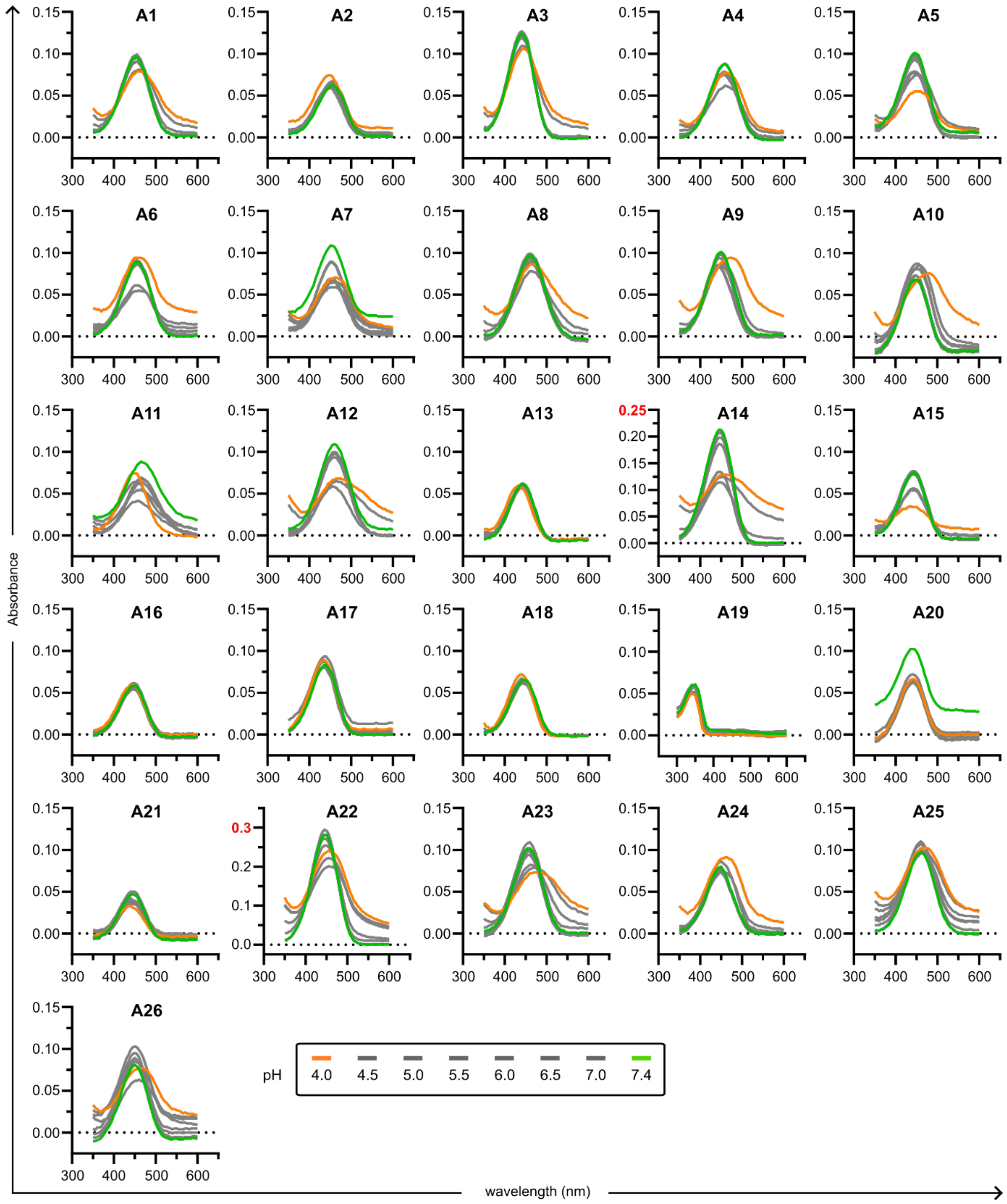

Figure S2. Representative absorbance spectra of naphthalimides. Absorbance spectra of the 26 compounds (10 μM) were measured in Na_2HPO_4–citric acid buffers ranging from pH 4.0 to pH 7.4. Absorbance scales are uniform except for compounds **A14** and **A22**, with values highlighted in red.

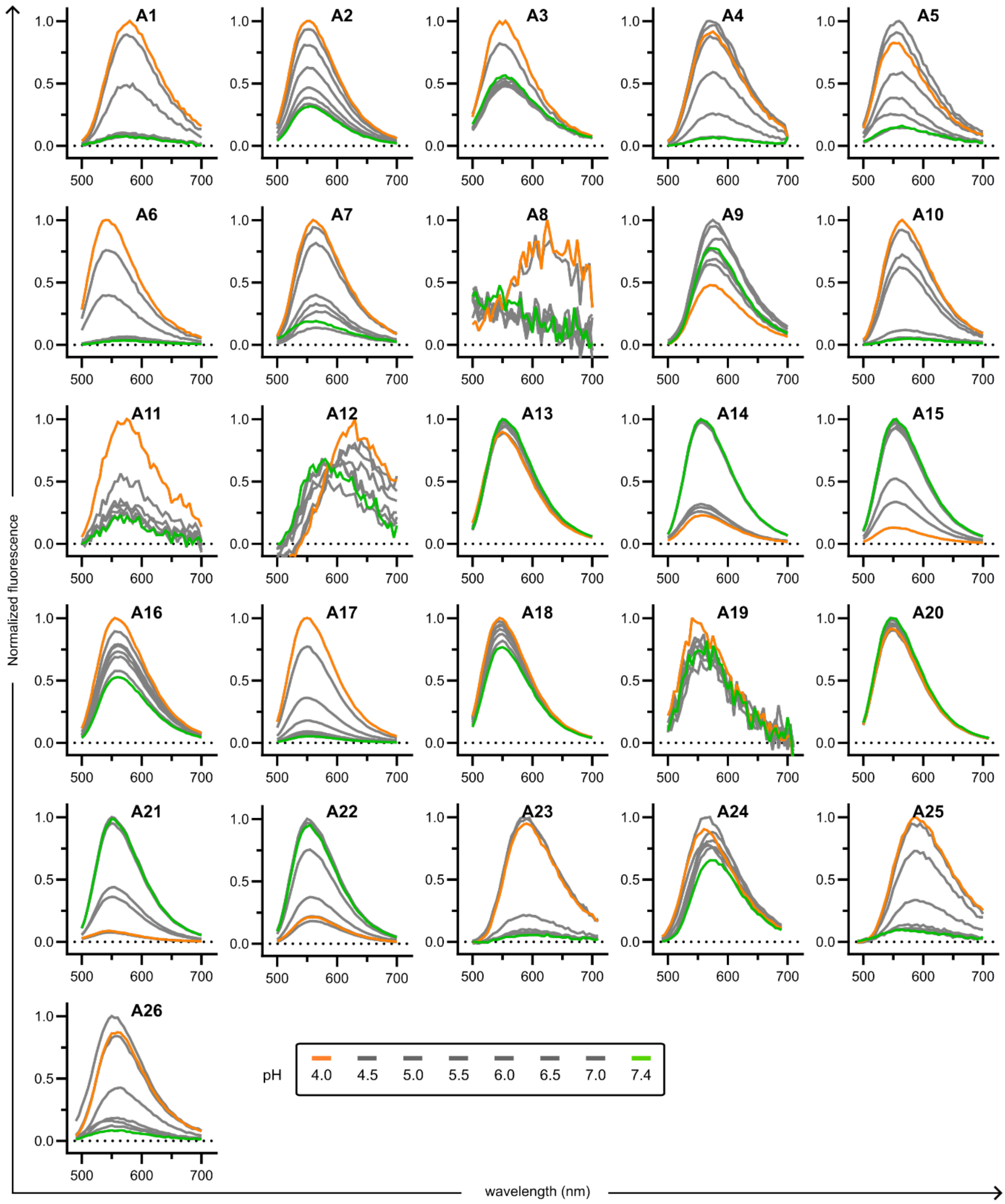

Figure S3. Representative fluorescence spectra of naphthalimides. Normalized fluorescence emission spectra of the 26 compounds (10 µM) in Na_2HPO_4–citric acid buffers ranging from pH 4.0 to pH 7.4 (λ_{exc}: 450 nm).

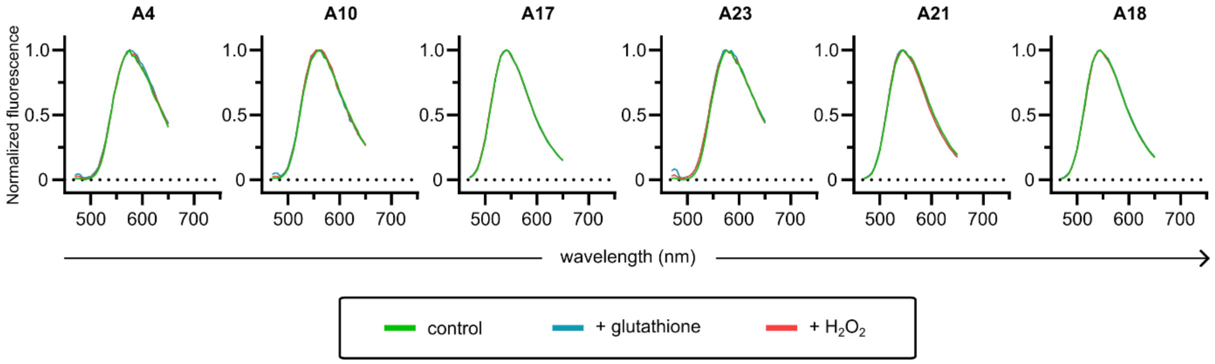

Figure S4. Representative fluorescence spectra of selected naphthalimides in reducing or oxidative conditions. Selected naphthalimides were diluted in Na_2HPO_4–citric acid buffers at pH 4.0 (**A4**, **A10**, **A17** and **A23**) or pH 7.4 (**A21** and **A18**) and incubated or not with glutathione (300 µM) or H_2O_2 (1 mM) before recording their fluorescence spectra (λ_{exc}: 450 nm).

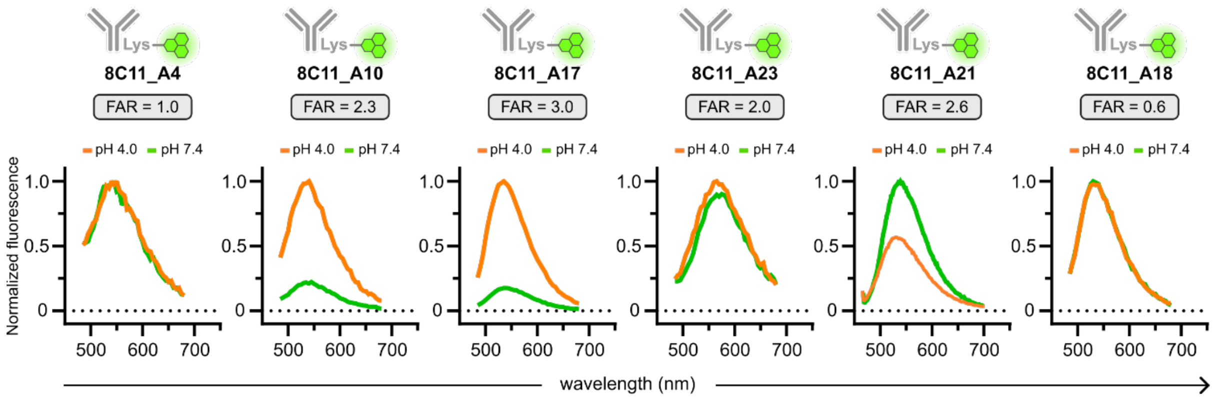

Figure S5. Representative fluorescence spectra of 8C11-fluorophore conjugates. Normalized fluorescence emission spectra of 8C11-fluorophore conjugates in Na_2HPO_4–citric acid buffers ranging from pH 4.0 to pH 7.4 (λ_{exc}: 450 nm). Fluorophore-antibody ratios (FAR) are indicated in the grey boxes.

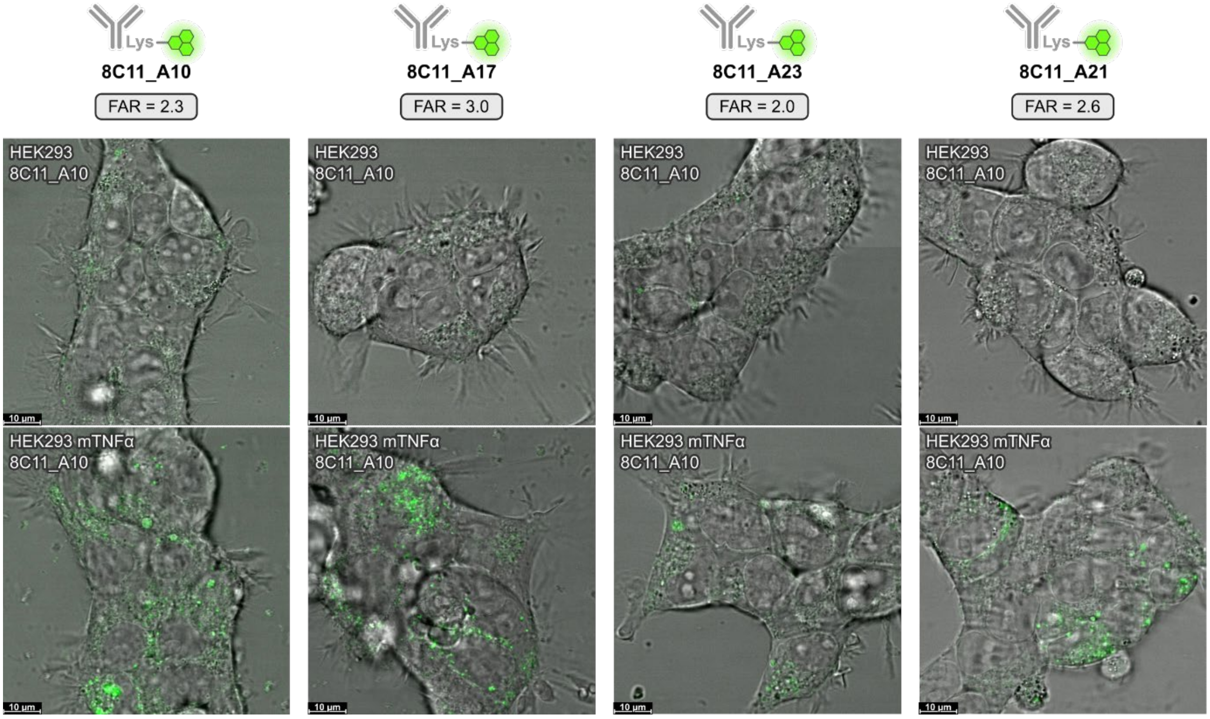

Figure S6. Representative confocal microscopy images of selected 8C11-fluorophore conjugates in transfected and non-transfected HEK293 cells. Representative fluorescence microscopy images of live HEK293 cells transfected with mTNFα and treated with **8C11_A10** (200 nM), **8C11_A17** (200 nM), **8C11_A23** (200 nM), **8C11_A21** (200 nM) for 2 hours. Images are overlays of fluorescence (exc/em: 450/525 nm) and brightfield. Scale bars: 10 µm.

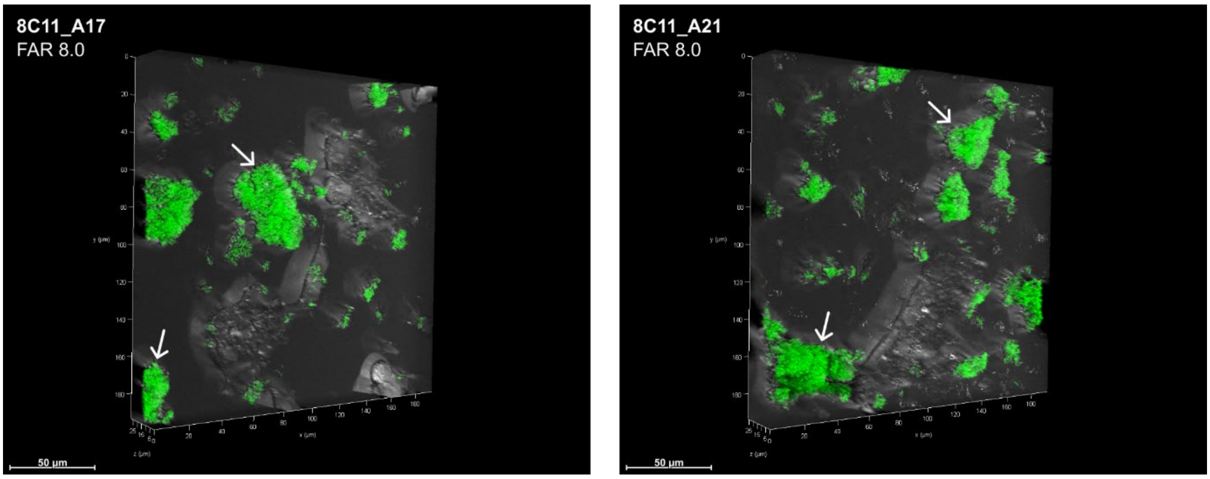

Figure S7. Z-stack imaging experiments of 8C11_A17 (FAR: 8.0) and 8C11_A21 (FAR: 8.0). Representative fluorescence microscopy 3D reconstructions of HEK293 cells transfected with mTNFα and incubated with **8C11_A17** (FAR: 8.0) (200 nM, left) and **8C11_A21** (FAR: 8.0) (200 nM, right) for 2 hours (exc/em: 450/550 nm). The presence of aggregates is highlighted by white arrows. Stack images were acquired across 25 µm with 0.6 µm increments. Scale bars: 50 µm.

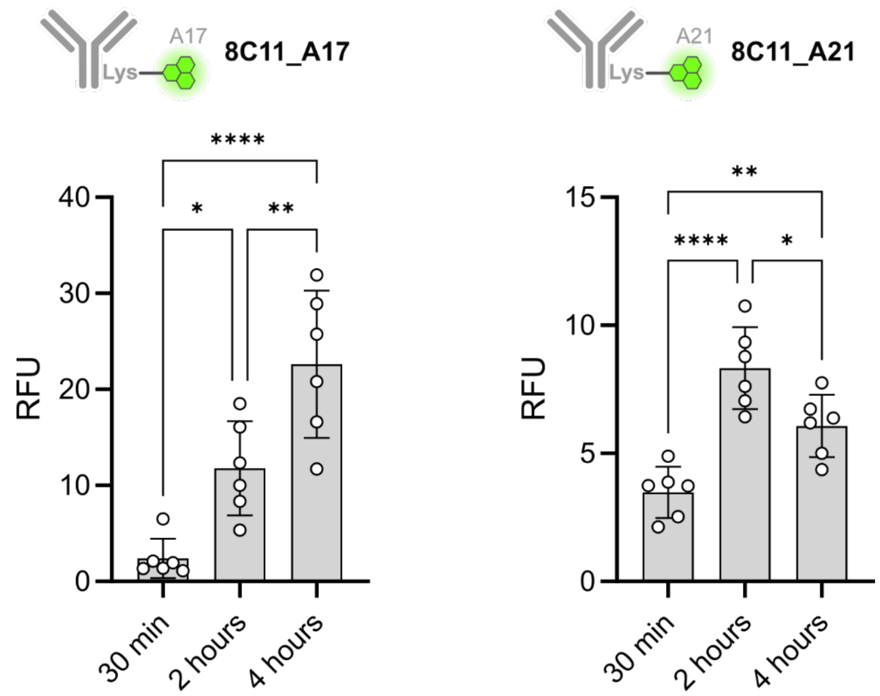

Figure S8. Quantification of mean cell fluorescence signals from Figure 4. Values of mean cell fluorescence signals (exc/em: 450/550 nm) from representative images of live HEK293 cells transfected with mTNFα and treated with **8C11_A17** (200 nM, left) or **8C11_A21** (200 nM, right) at different time points (t: 30 minutes, 2 hours, and 4 hours) were calculated with FIJI and presented as individual replicates and bars as means ± SEM (n=6). *P*-values were calculated using a one-way ANOVA; $p < 0.05$ (*), $p < 0.01$ (**), $p < 0.001$ (***), $p < 0.0001$ (****).

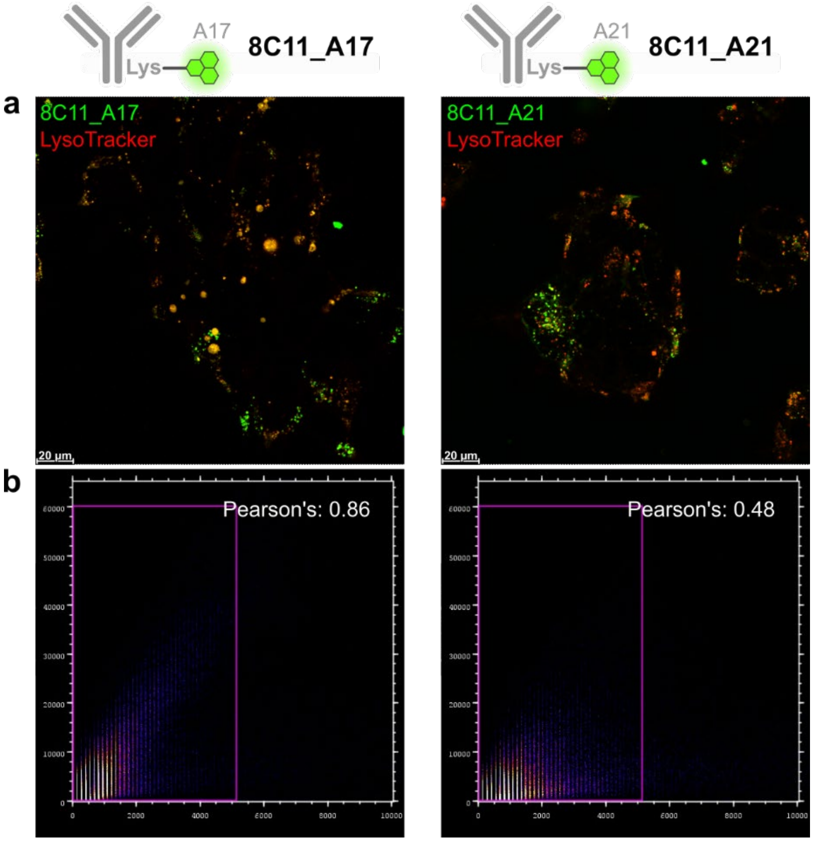

Figure S9. Co-localization analysis of 8C11_A17 and 8C11_A21 with LysoTracker™ Red. a) Representative fluorescence microscopy images of HEK293 cells transfected with mTNFα and co-incubated with **8C11_A17** (left) or **8C11_A21** (right) (both at 200 nM, exc/em: 450/550 nm, green) and LysoTracker™ Red (50 nM, exc/em: 573/593 nm, red) for 2 hours. Scale bars: 20 µm. b) Pearson correlation coefficients were calculated with FIJI, and co-localization graphs generated using the same software.

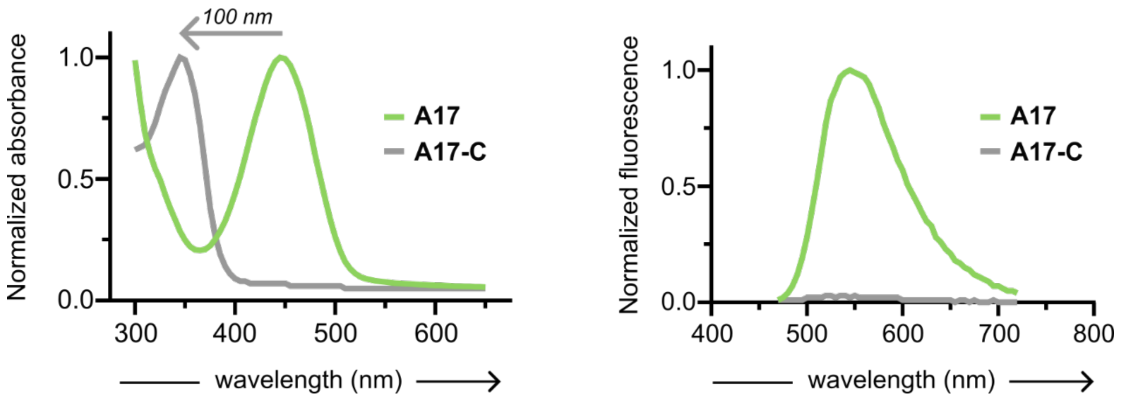

Figure S10. Representative absorbance and emission spectra of compounds A17 and A17-C. Normalized absorbance (left) and emission spectra (right, λ_{exc}: 450 nm) of compounds **A17** (10 µM) and **A17-C** (10 µM) in Na_2HPO_4–citric acid buffer (pH 4.0). The shift in absorbance between compounds is indicated with an arrow.

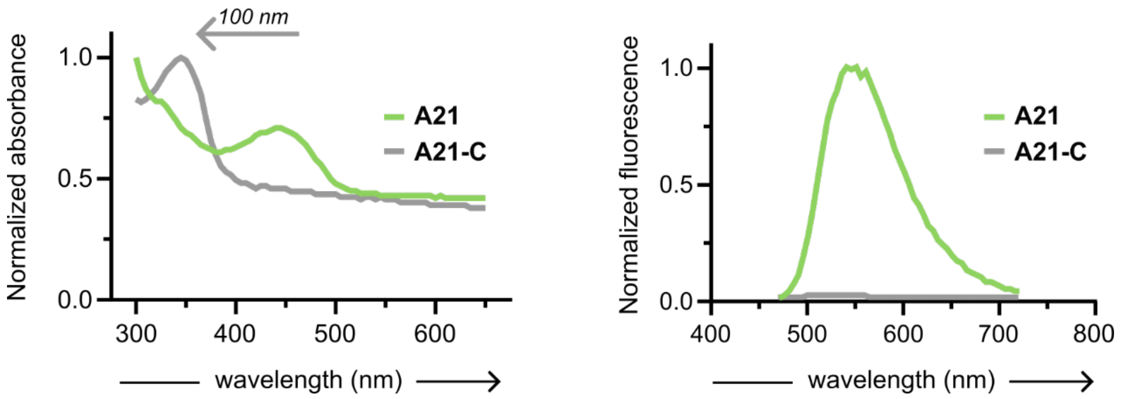

Figure S11. Representative absorbance and emission spectra of A21-C. Normalized absorbance (left) and emission spectra (right, λ_{exc}: 450 nm) of compounds **A21** (10 μM) and **A21-C-AA** (10 μM) in Na_2HPO_4–citric acid buffer pH 7.4. The shift in absorbance between compounds is indicated with an arrow.

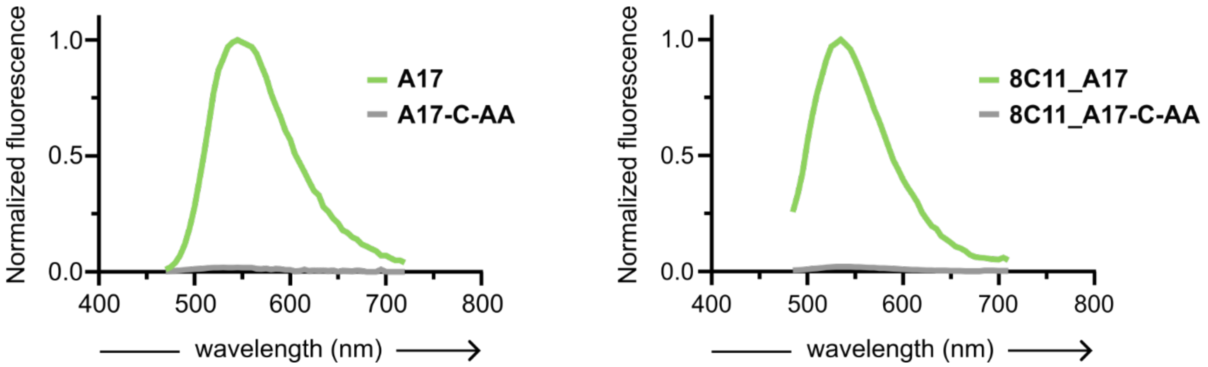

Figure S12. Representative emission spectra of A17-C-AA and 8C11_A17-C-AA. Normalized emission spectra of the free dye **A17-C-AA** (10 µM, left) and its antibody-conjugated form, **8C11_A17-C-AA** (200 nM, right), in Na_2HPO_4–citric acid buffer (pH 4.0). Spectra were normalized to their uncaged analogues, **A17** (10 µM) and **8C11_A17** (200 nM), respectively. (λ_{exc}: 450 nm).

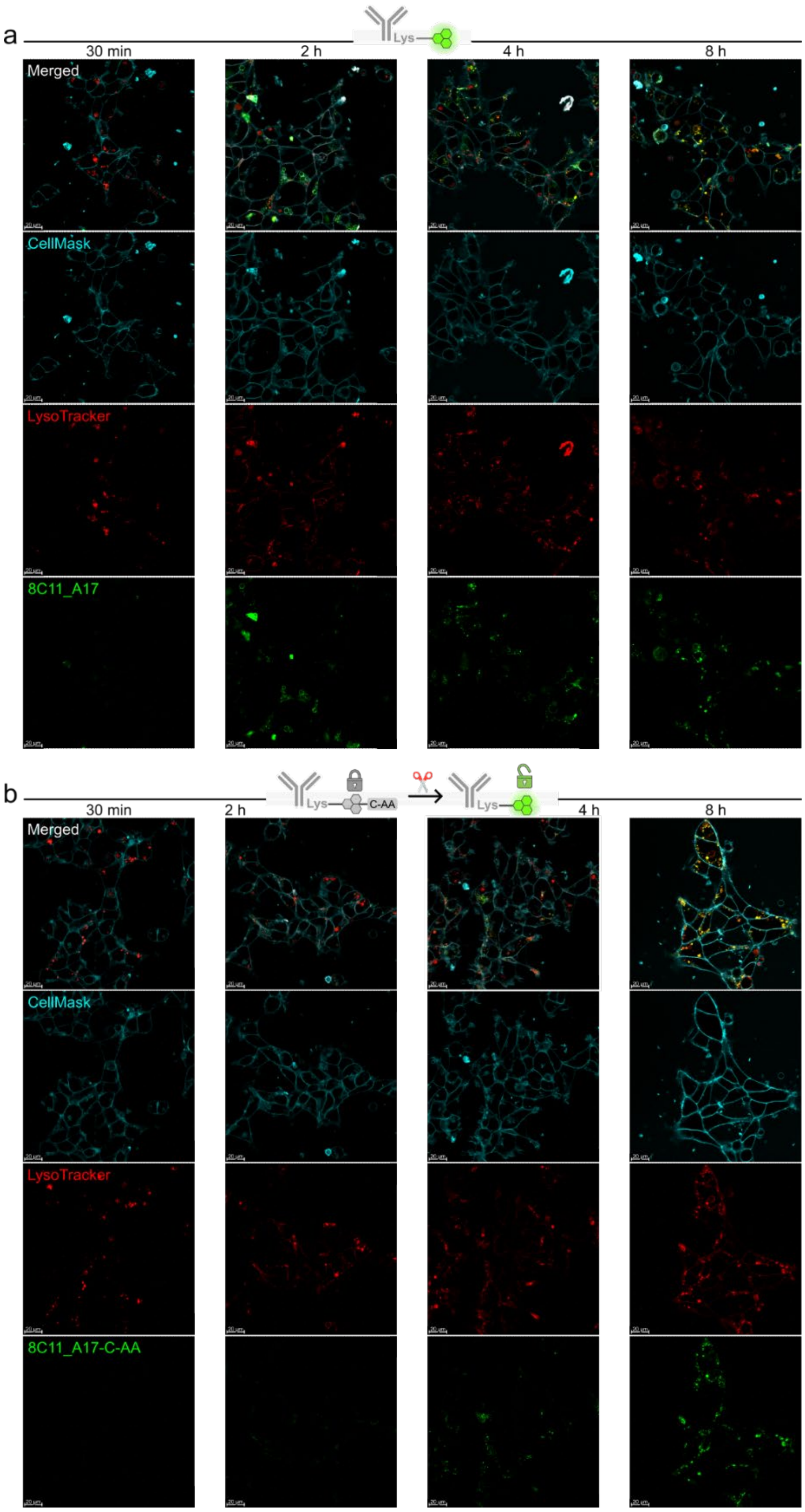

Figure S13. Single-channel fluorescence images from Figure 4. Representative fluorescence microscopy images of live HEK293 cells transfected with mTNFα and treated with 200 nM **8C11_ A17** (top) or **8C11_A17-C-AA** (bottom) (exc/em: 450/550) at different time points (t: 30 minutes, 2 hours, 4 hours and 8 hours). Cells were co-stained with LysoTracker^{TM} Red (exc/em: 573/593 nm, red) and CellMask^{TM} Deep Red

(exc/em: 660/675 nm, cyan). The bottom rows of the merged images include the single-channel fluorescence of 8C11-dye conjugates, LysoTracker™ Red and CellMask™ Deep Red. Scale bars: 20 μm.

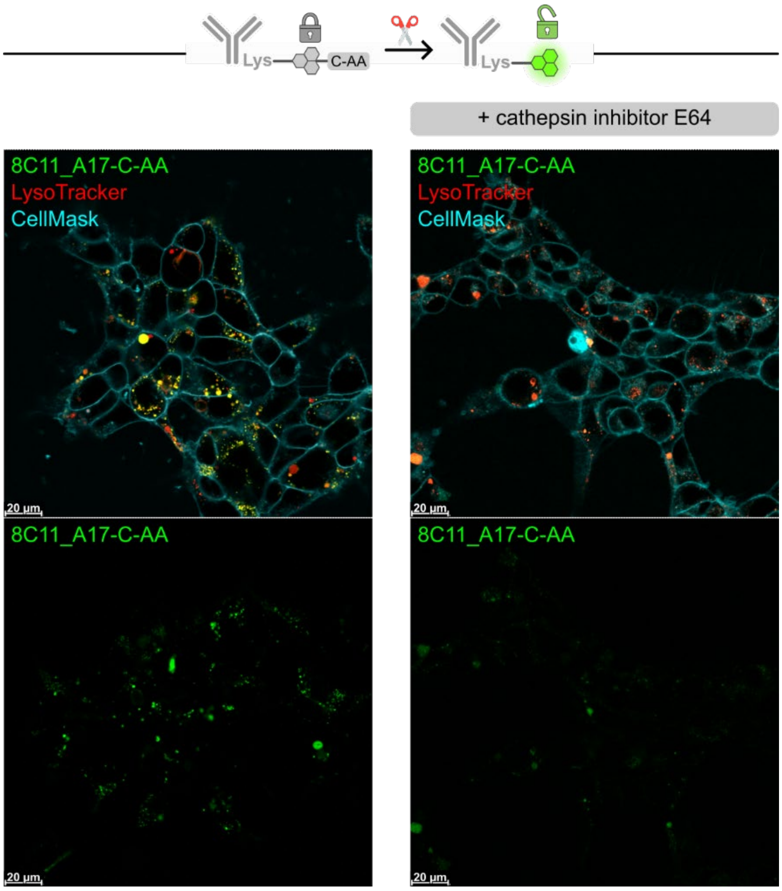

Figure S14. Enzymatic inhibition of cathepsin B in cells treated with A17-C-AA. Representative fluorescence microscopy images of live HEK293 cells transfected with mTNFα and treated with 200 nM **8C11_A17-C-AA** (exc/em: 450/550) for 8 hours, with (right) or without (left) the cathepsin inhibitor E64 (10 µM). Cells were co-stained with LysoTracker™ Red (exc/em: 573/593 nm, red) and CellMask™ Deep Red (exc/em: 660/675 nm, cyan). The bottom row shows single-channel fluorescence images of the 8C11-dye conjugates. Scale bars: 20 µm.

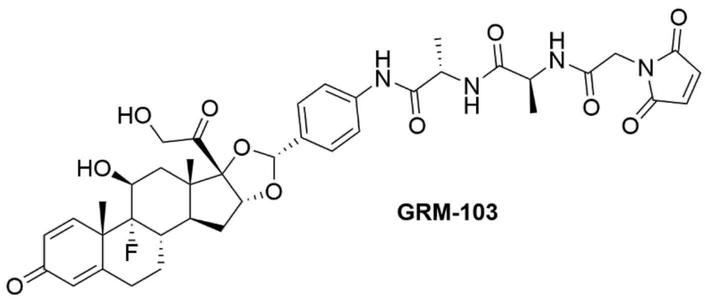

GRM-103

Figure S15. Chemical structure of GRM-103.[2] This payload-linker bearing a cleavable Ala-Ala dipeptide was used for the preparation of **8C11_PL** (DAR: 4.0) and **8C11_PL_A17-C-AA** (DAR: 4.0, FAR: 3.0).

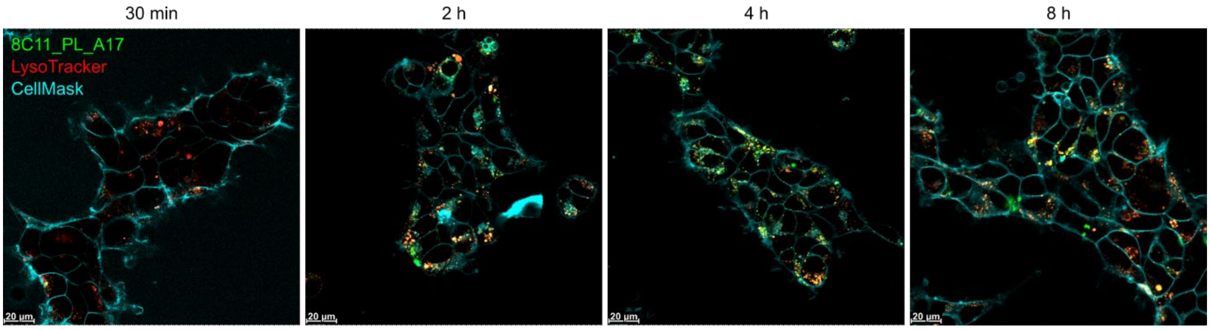

Figure S16. Representative confocal microscopy images of 8C11_PL_A17 in transfected HEK293 cells. Representative fluorescence microscopy images of live HEK293 cells transfected with mTNFα and treated with 200 nM **8C11_ PL_A17.** Images were taken at different time points (30 min, 2 hours, 4 hours and 8 hours). Cells were co-stained with Lysotracker^{TM} Red (exc/em: 573/593 nm, red) and CellMask^{TM} Deep Red (exc/em: 660/675 nm, cyan). Scale bars: 20 µm.

4. NMR Spectra

compound 2 (DMSO-d6)

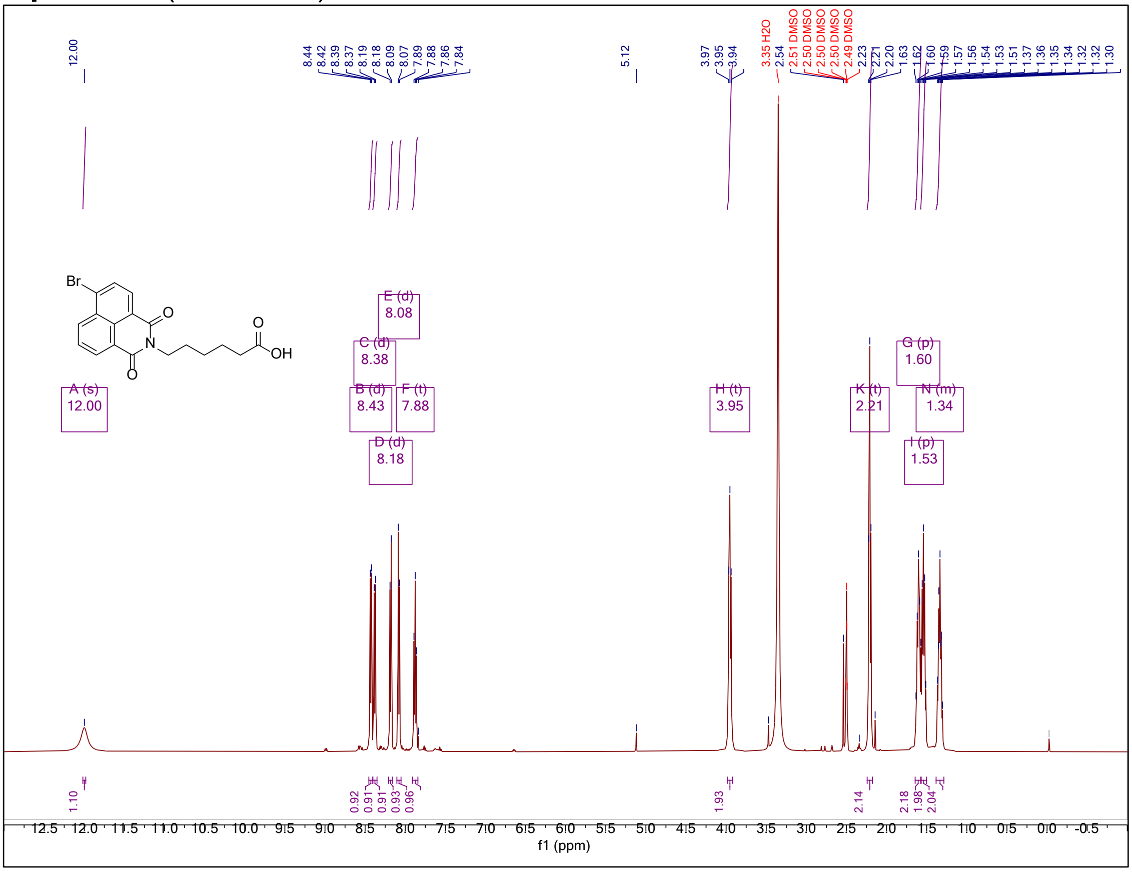

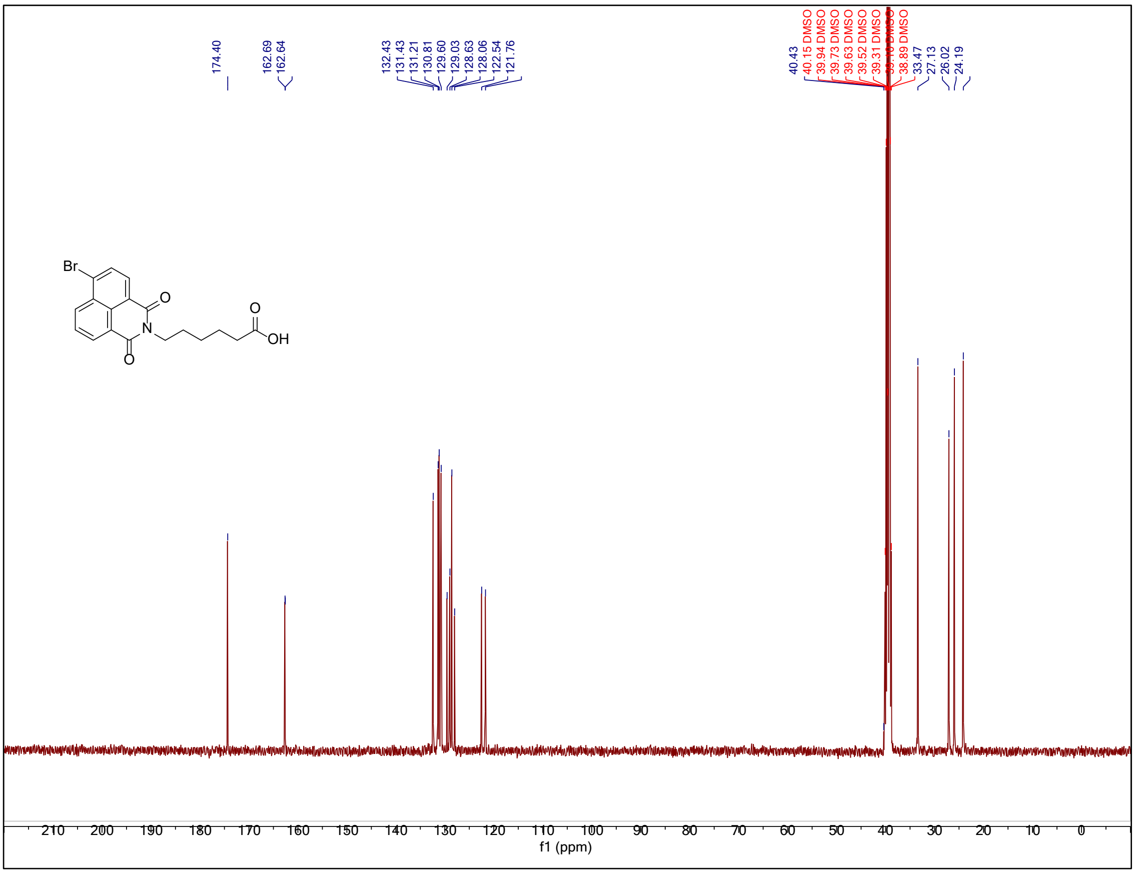

compound A1 (DMSO-d6)

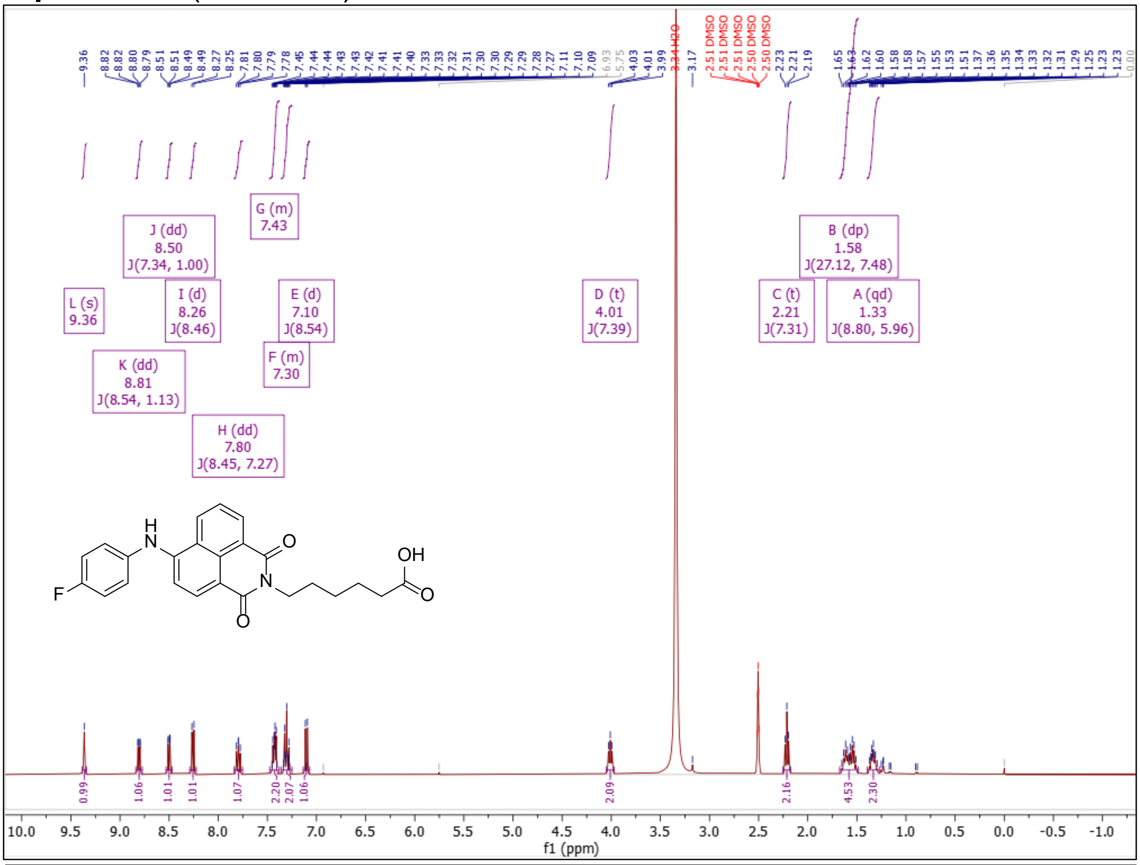

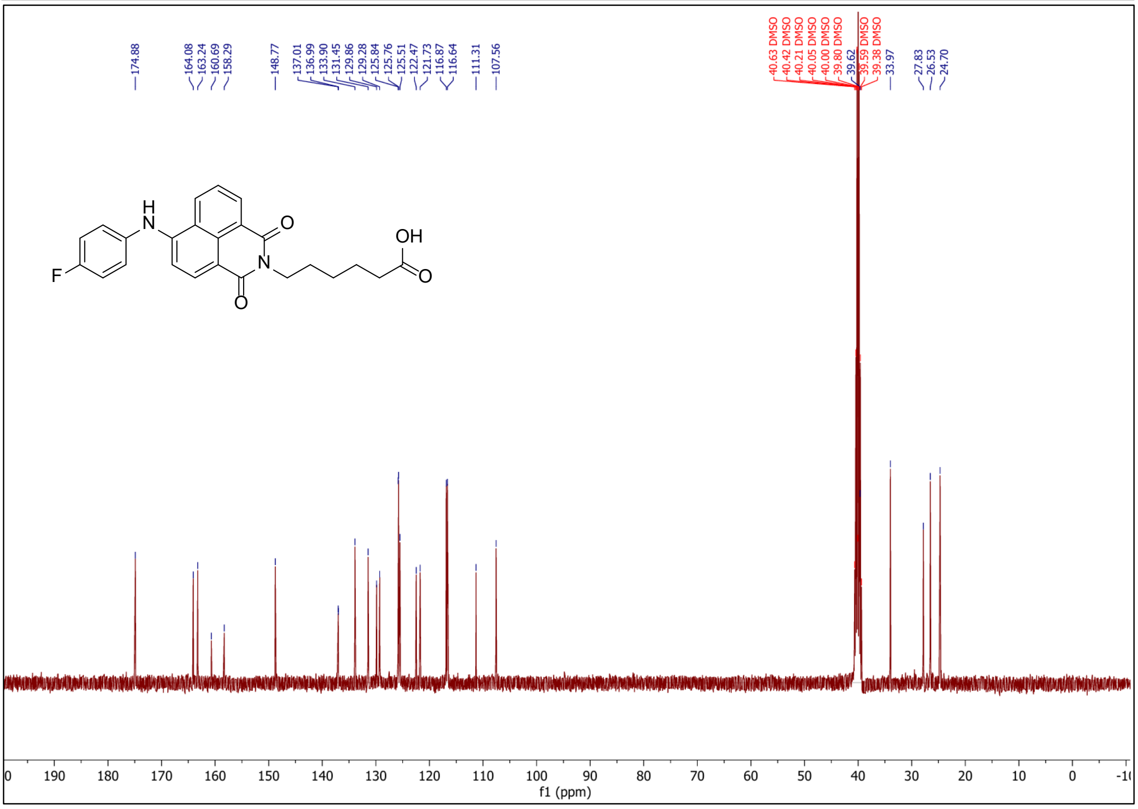

compound A2 (DMSO-d6)

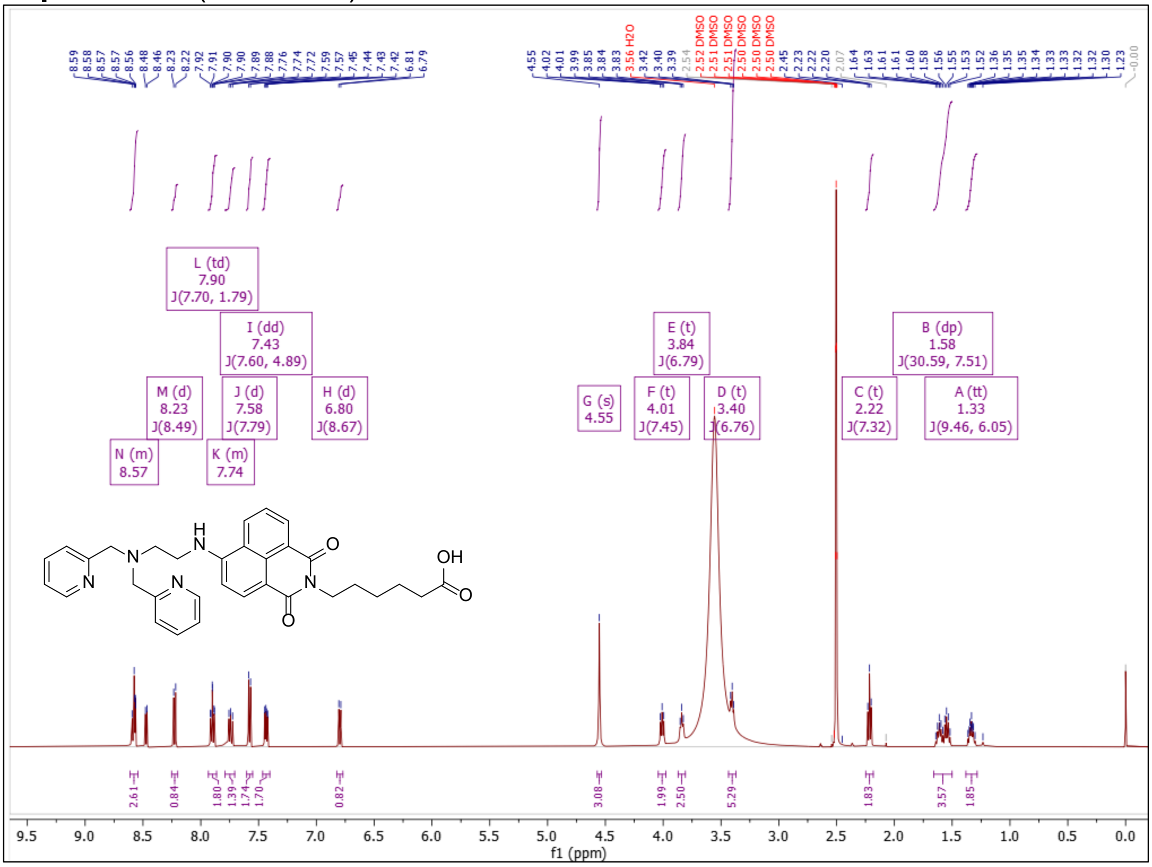

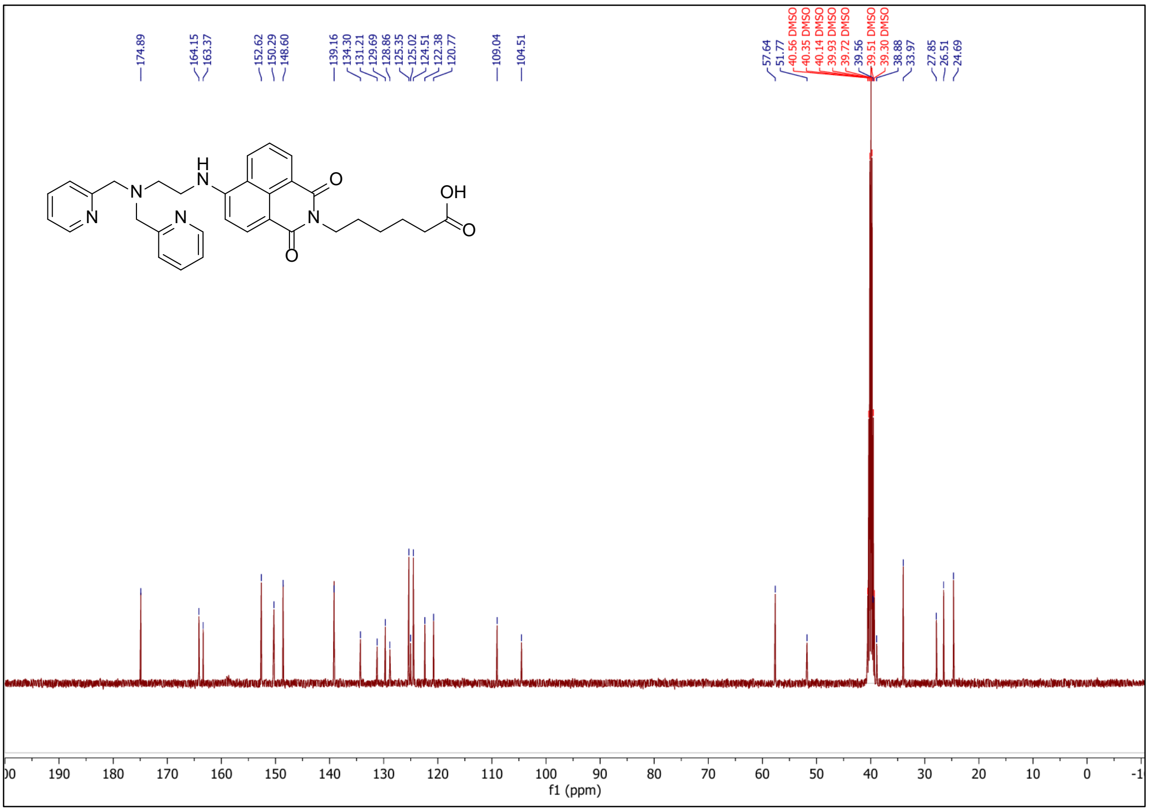

compound A3 (DMSO-d6)

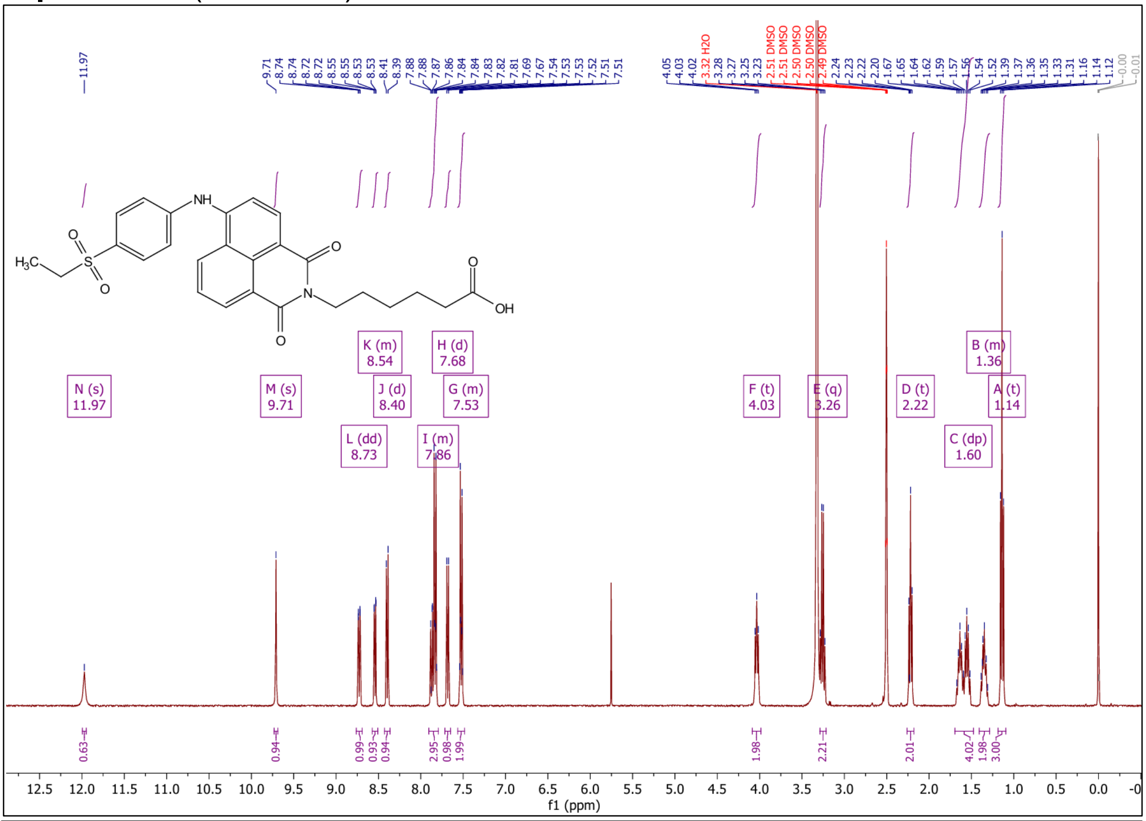

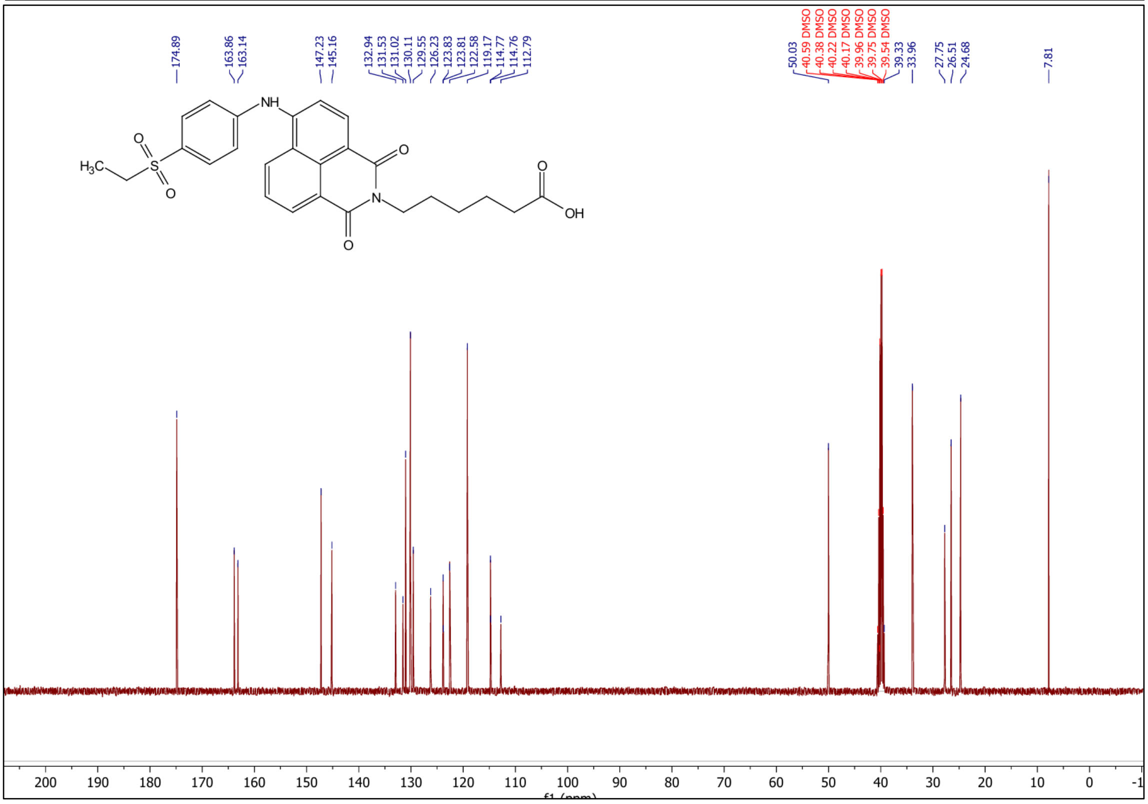

compound A4 (DMSO-d6)

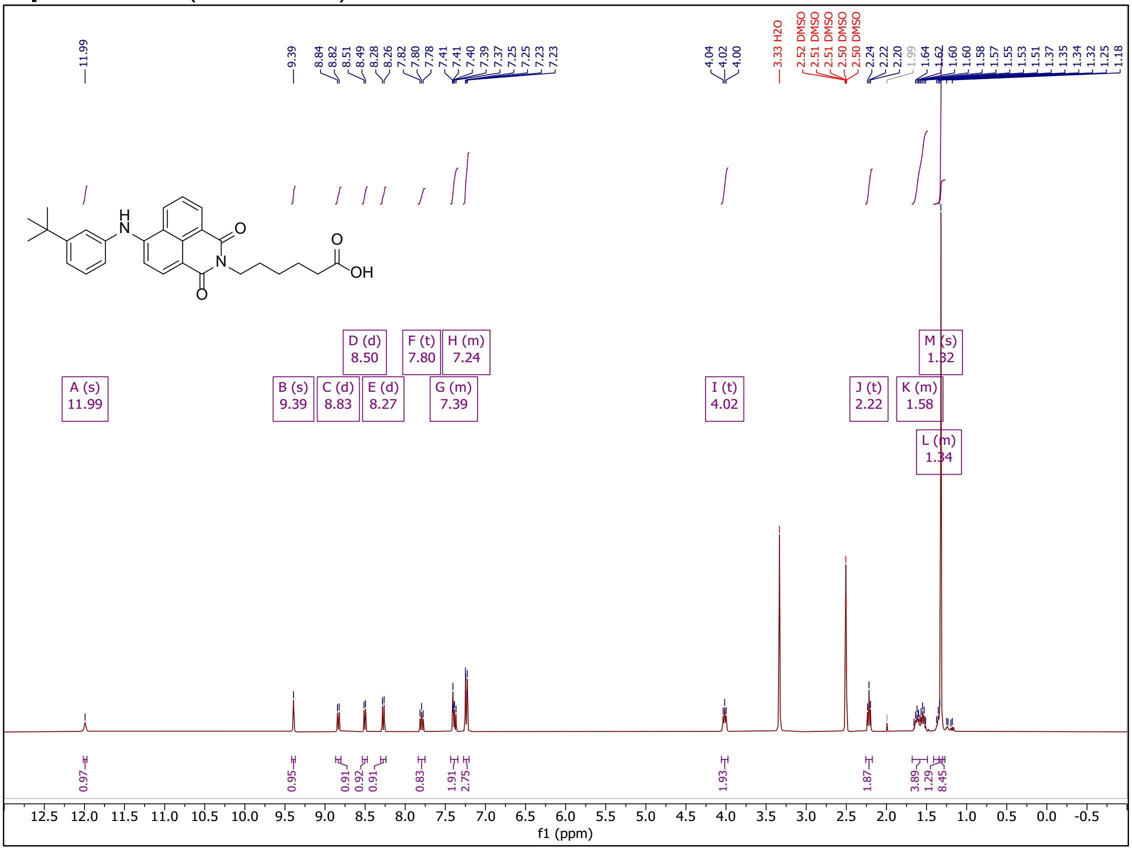

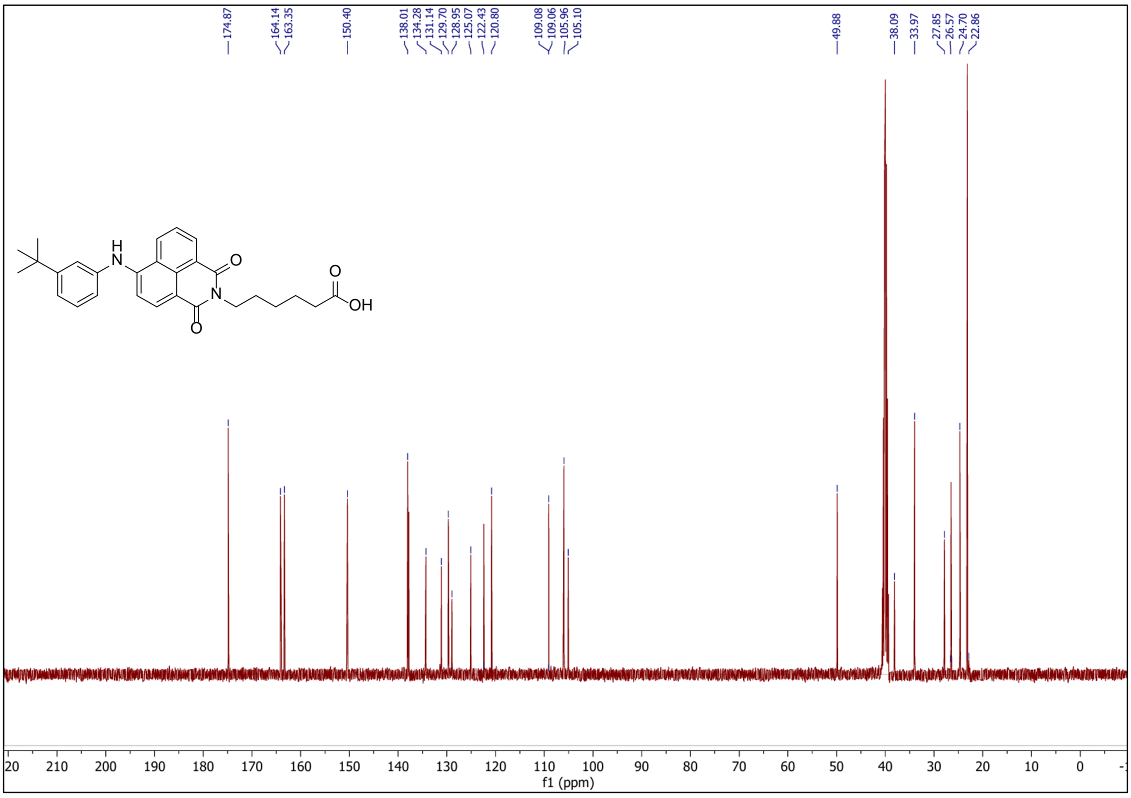

compound A5 (DMSO-d6)

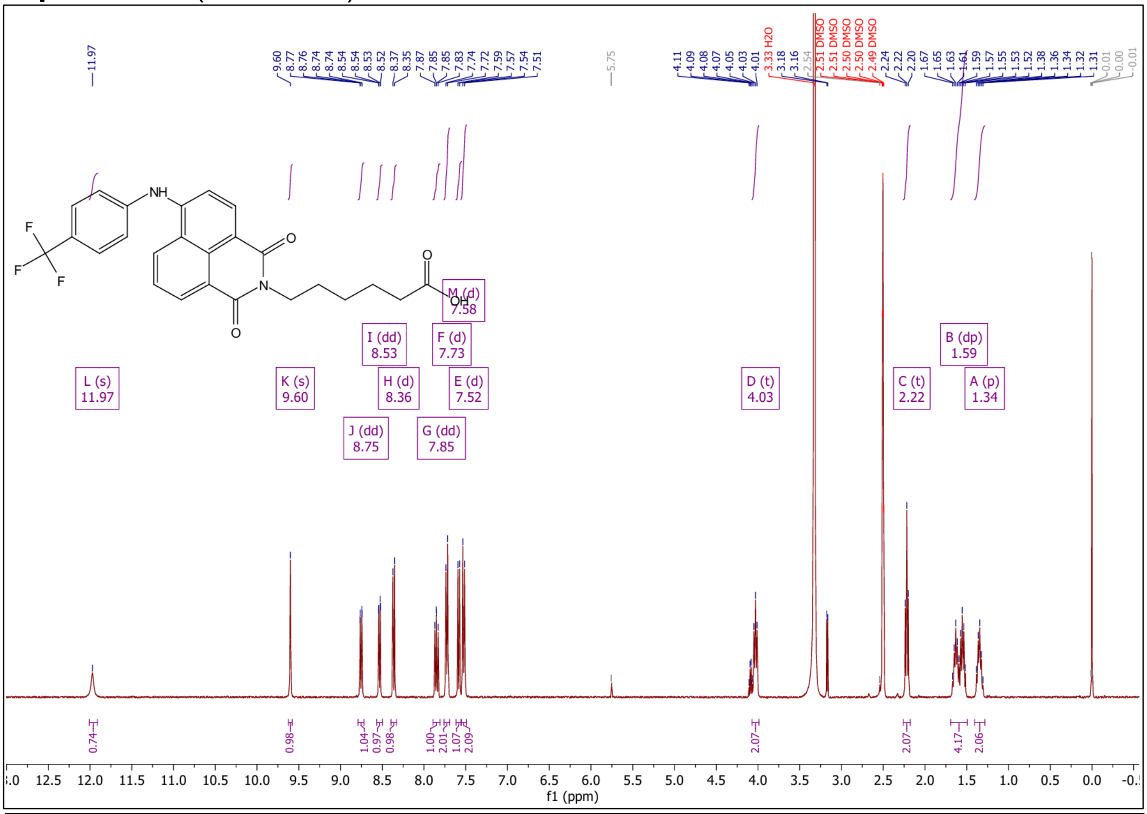

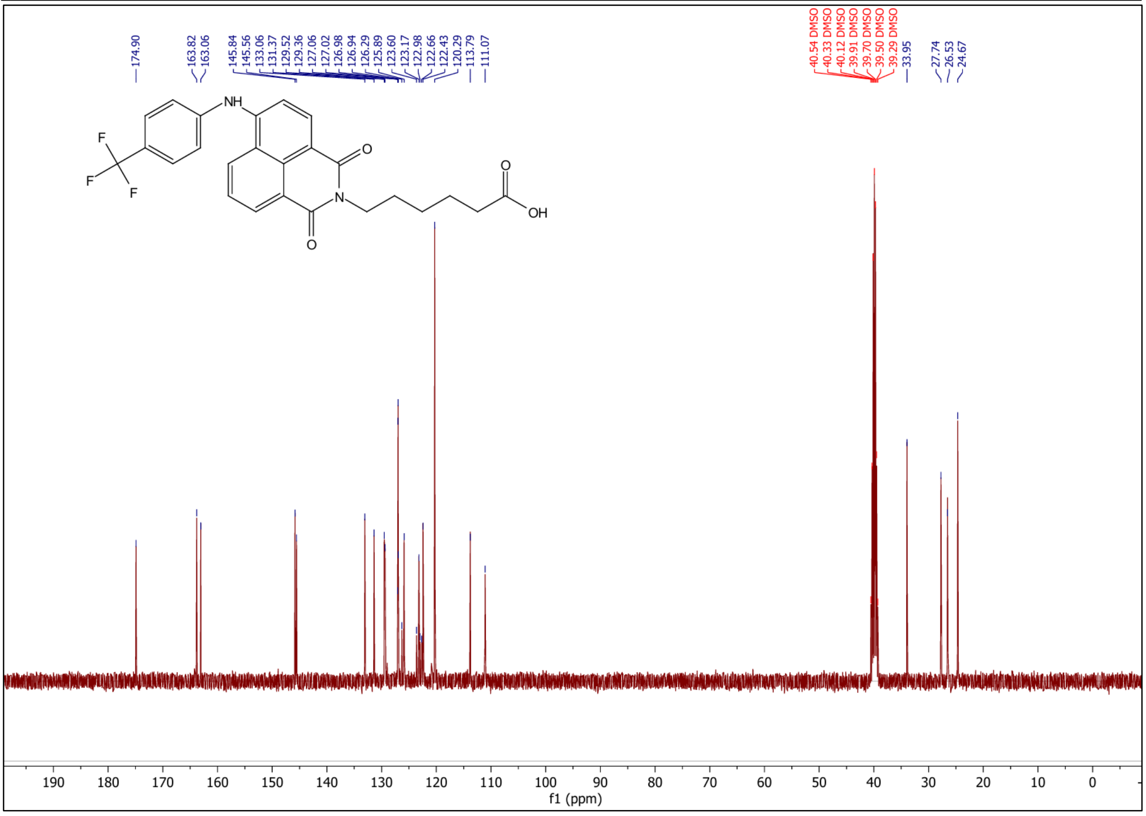

compound A6 (DMSO-d6)

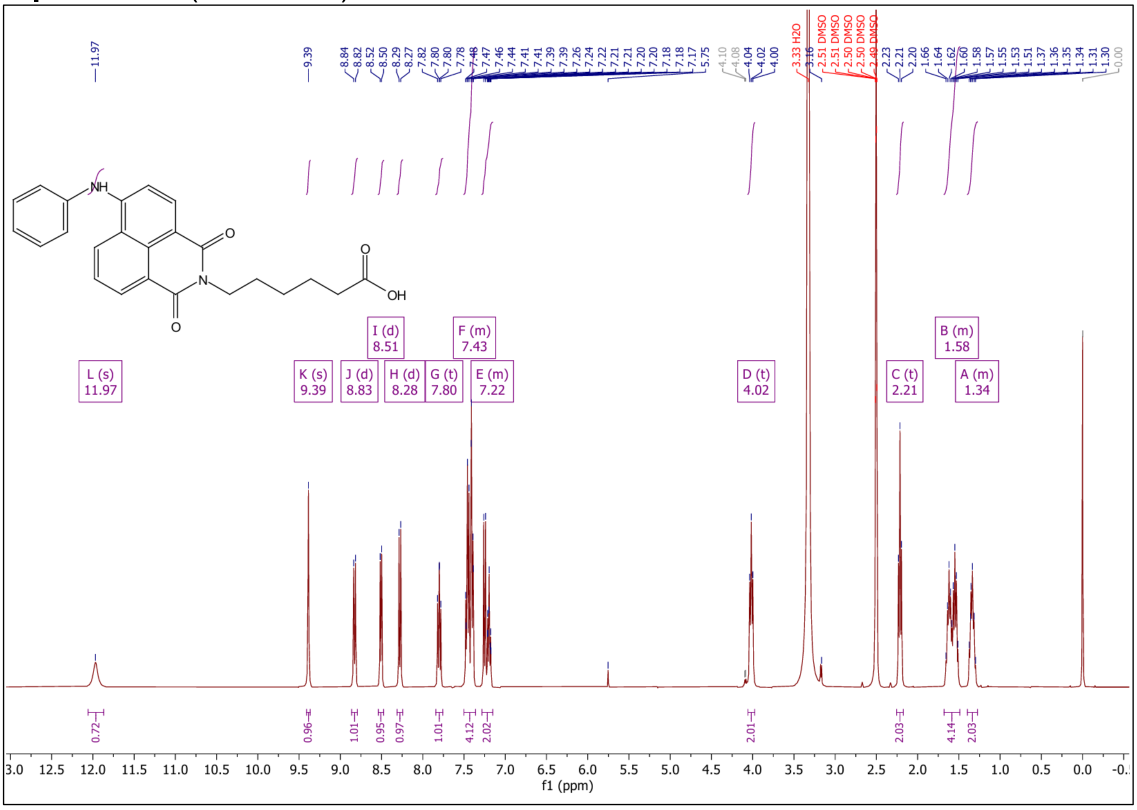

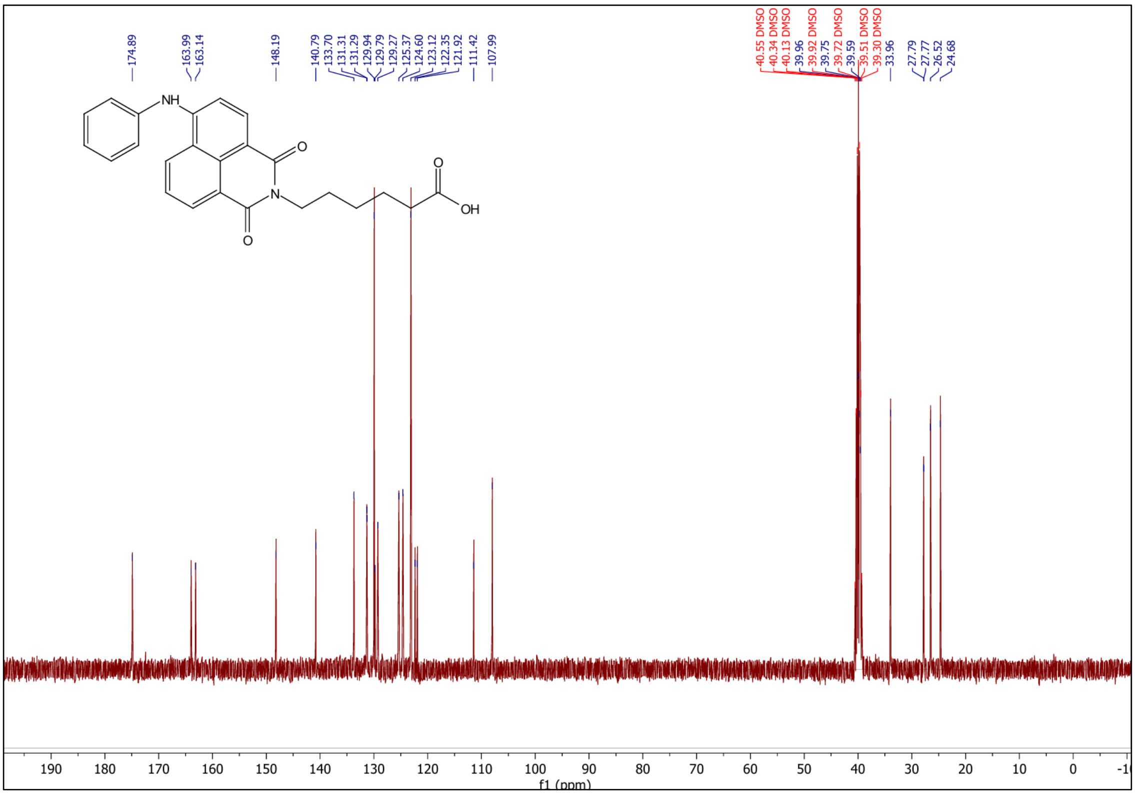

compound A7 (DMSO-d6)

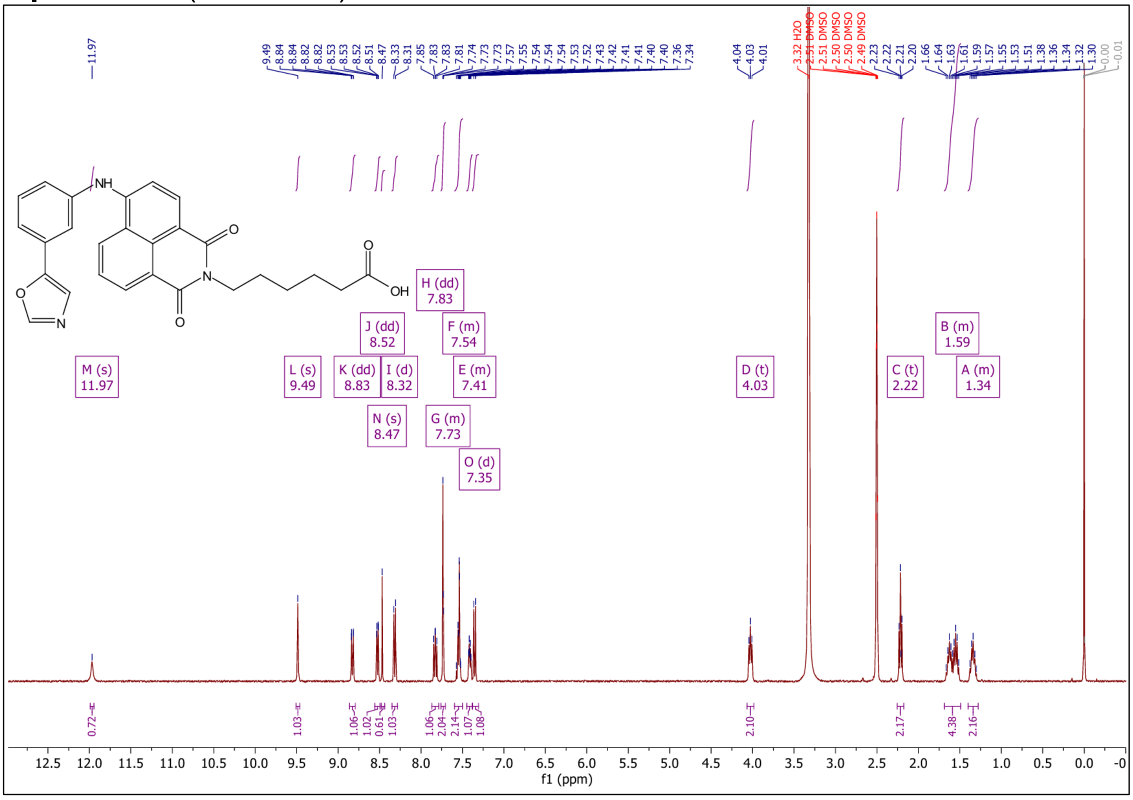

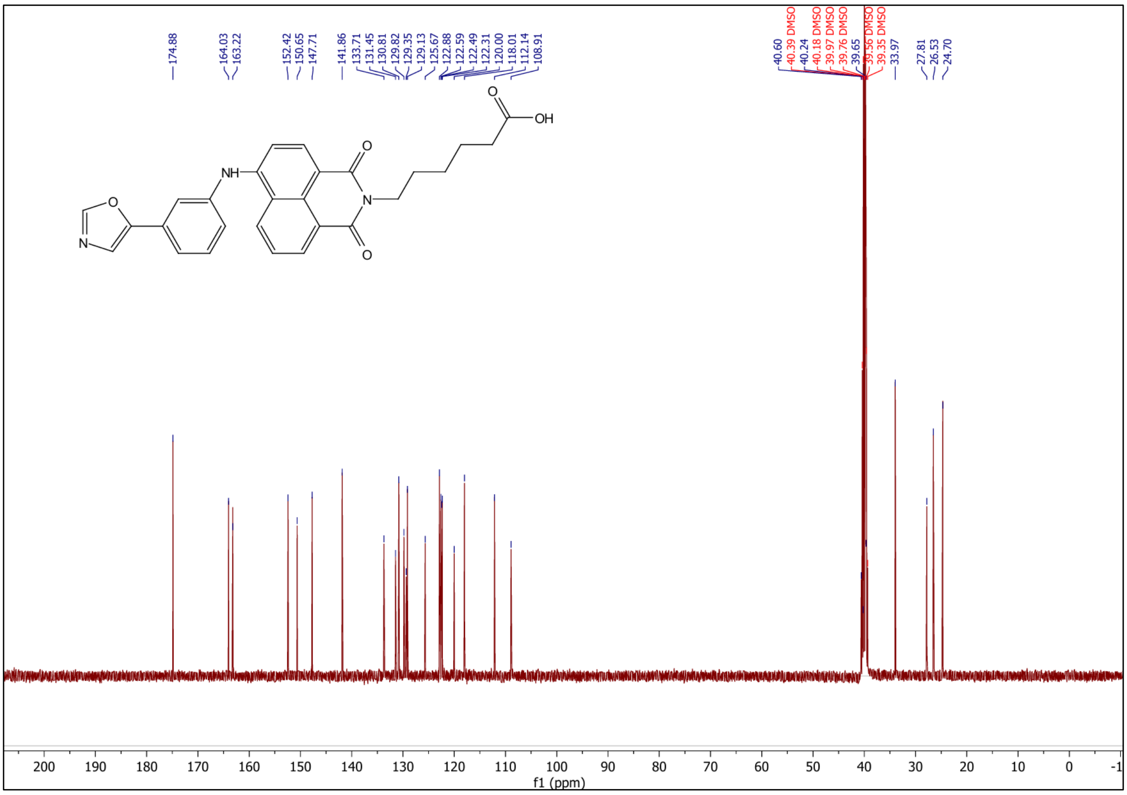

compound A8 (DMSO-d6)

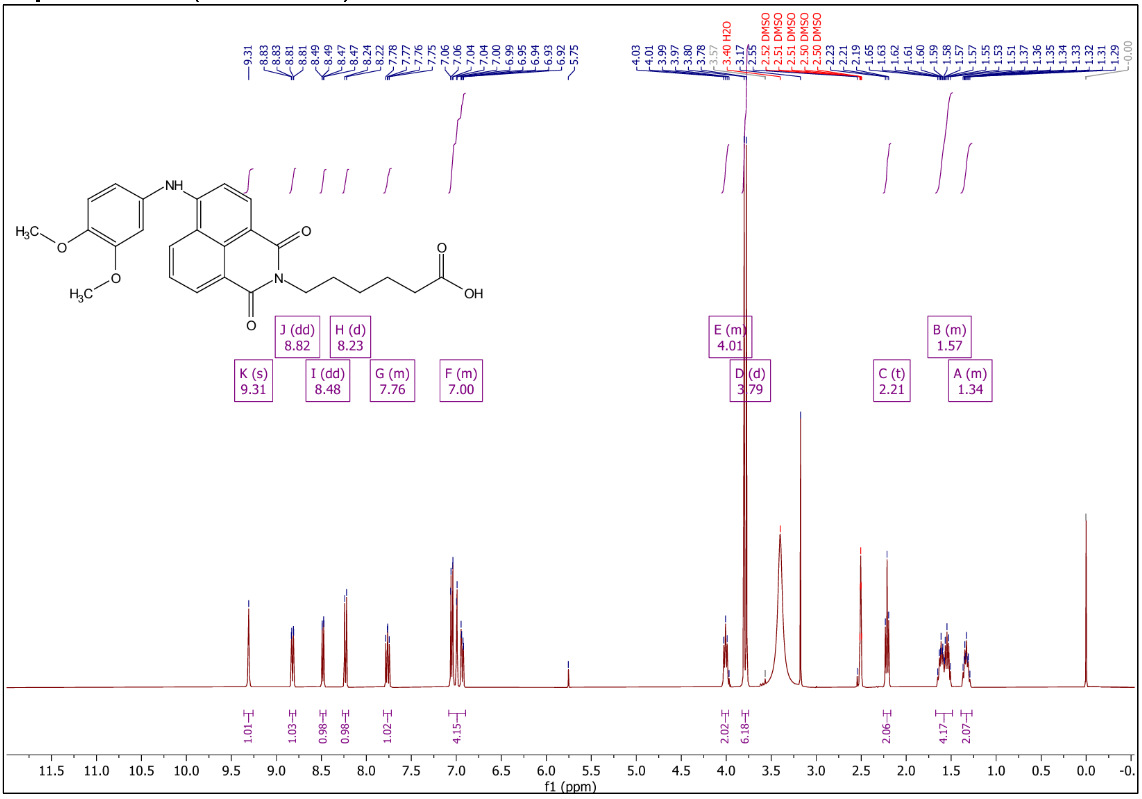

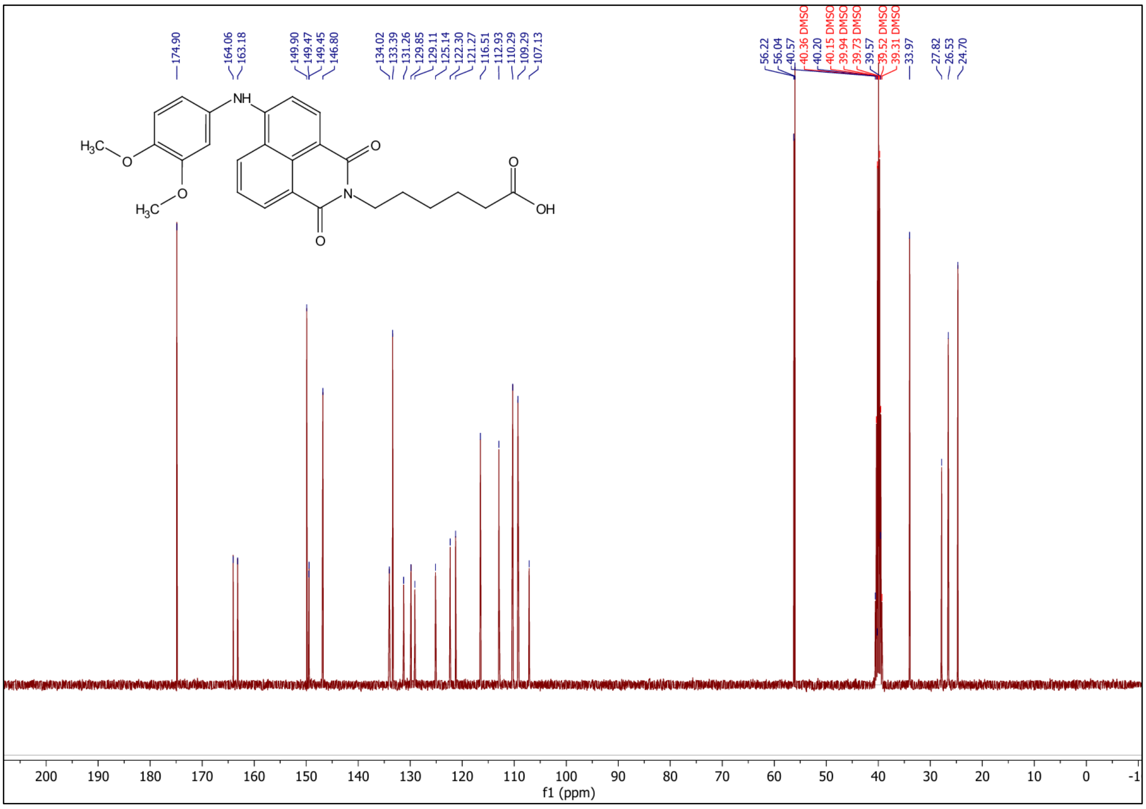

compound A9 (DMSO-d6)

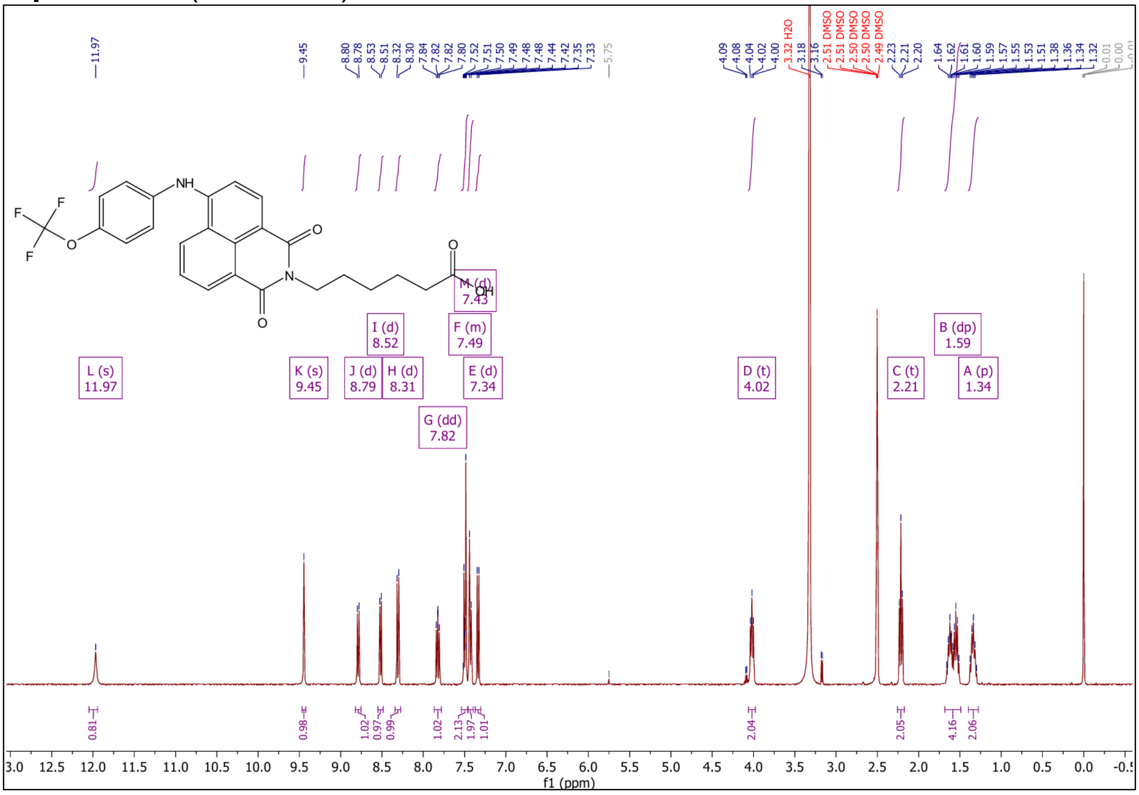

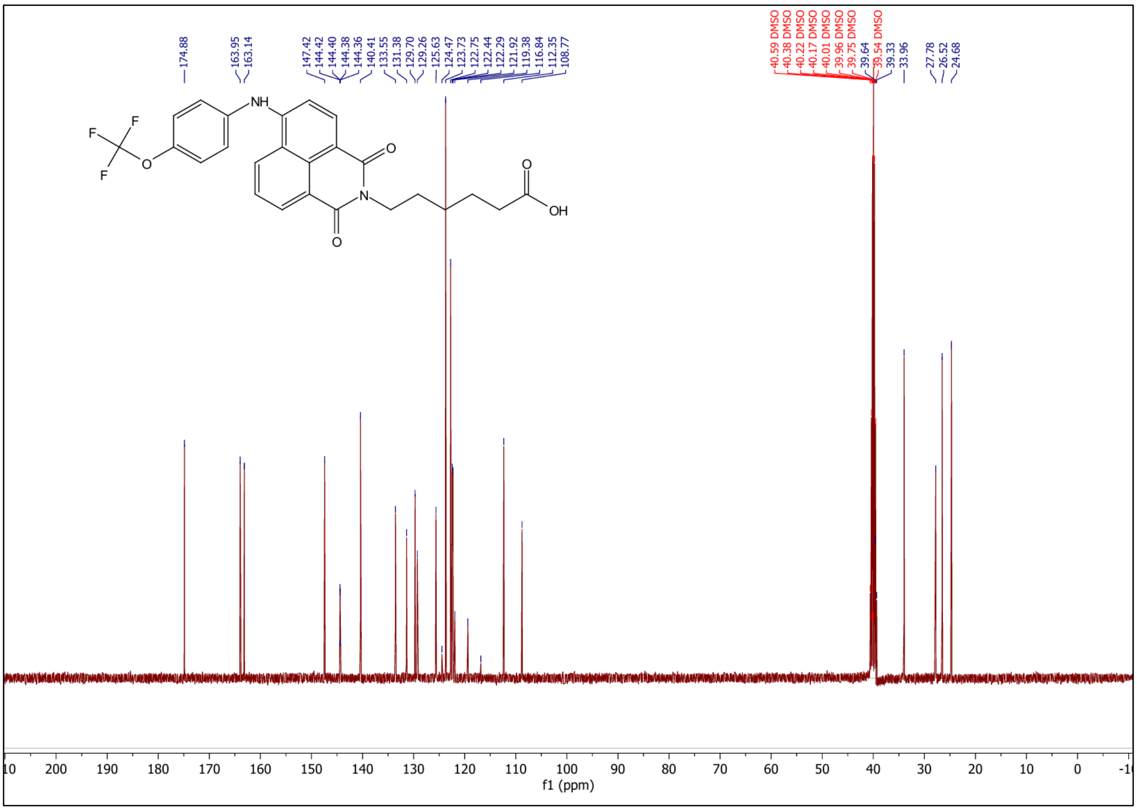

compound A10 (DMSO-d6)

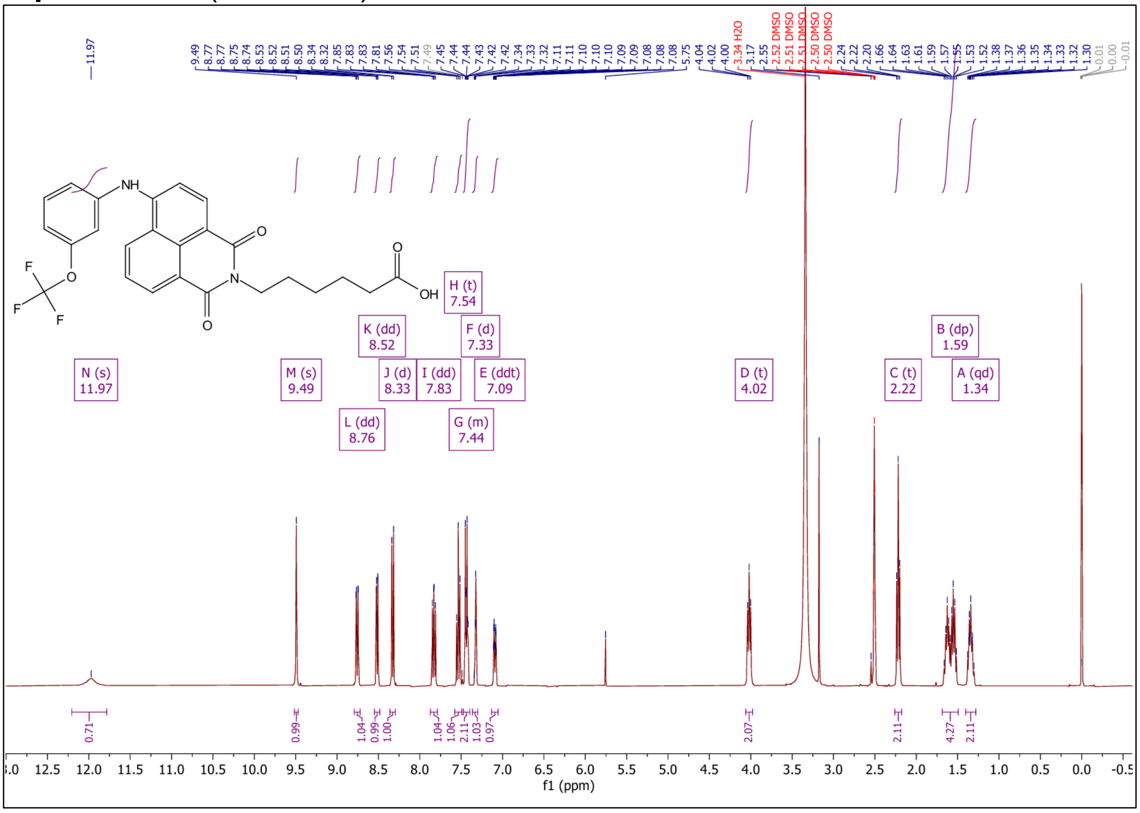

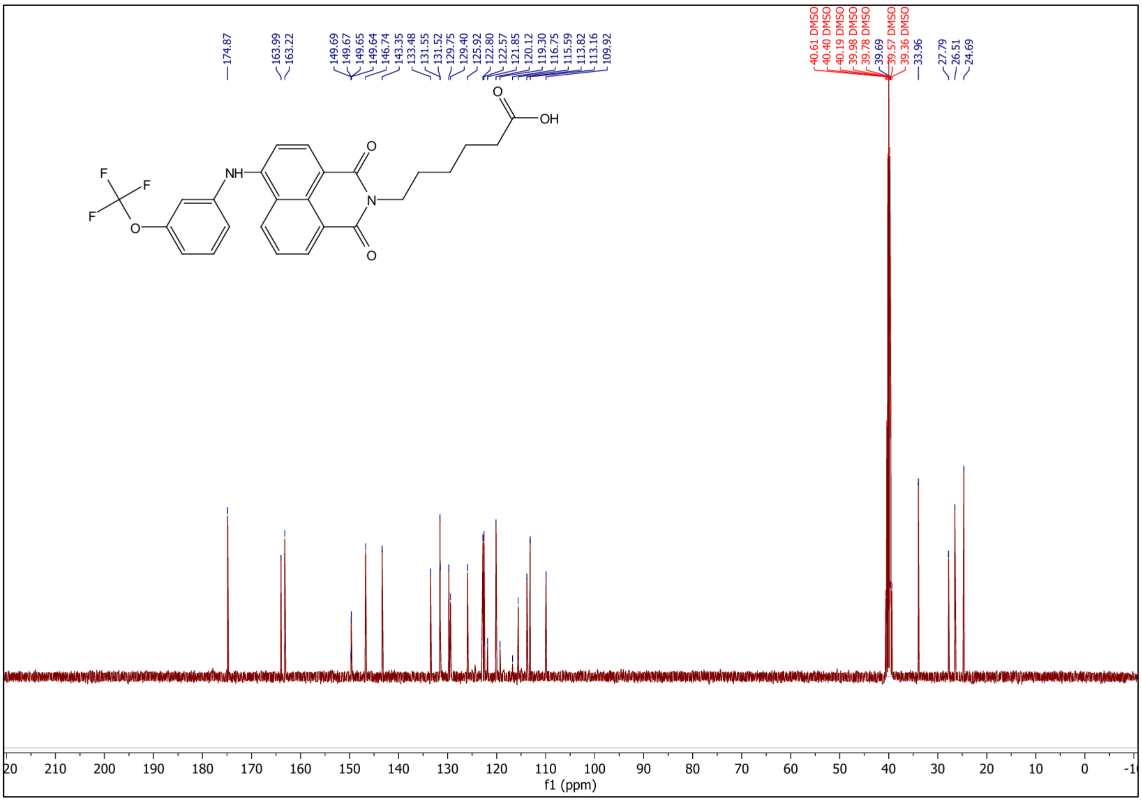

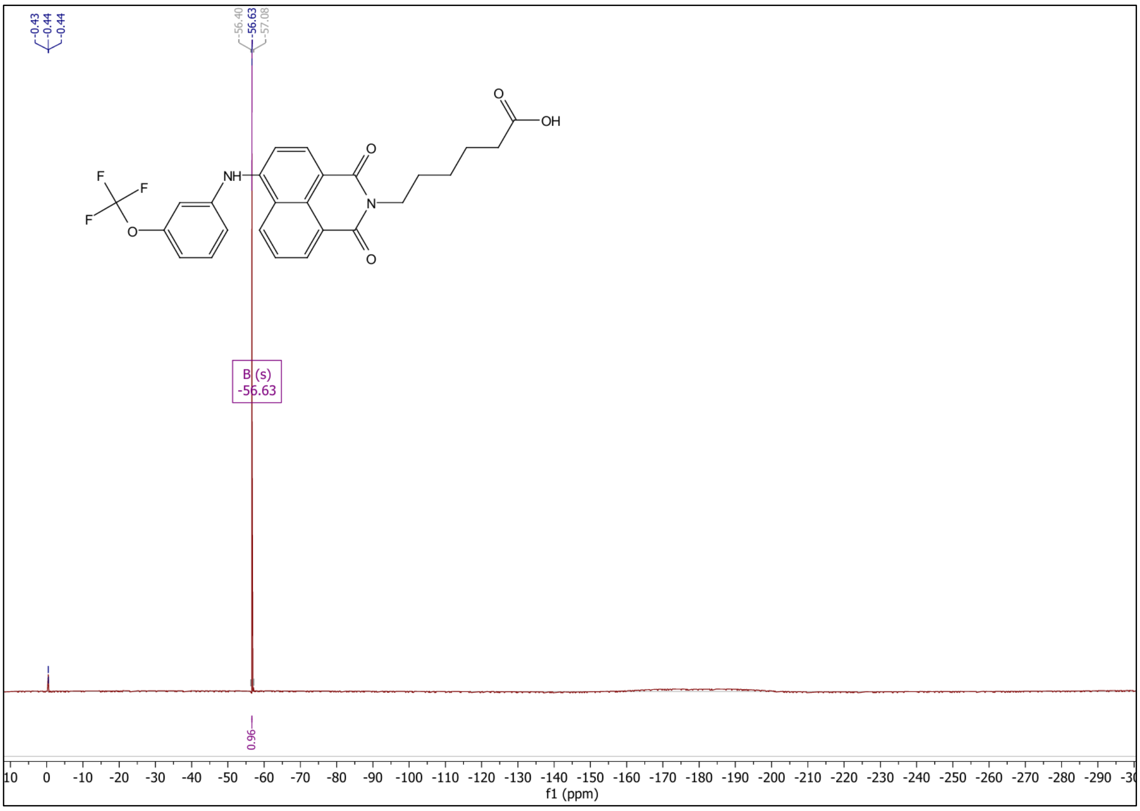

-0.43
-0.44
-0.44
-56.40
-56.63
-57.08
B (s)
-56.63
0.96
10 0 -10 -20 -30 -40 -50 -60 -70 -80 -90 -100 -110 -120 -130 -140 -150 -160 -170 -180 -190 -200 -210 -220 -230 -240 -250 -260 -270 -280 -290
f1 (ppm)

compound A11 (DMSO-d6)

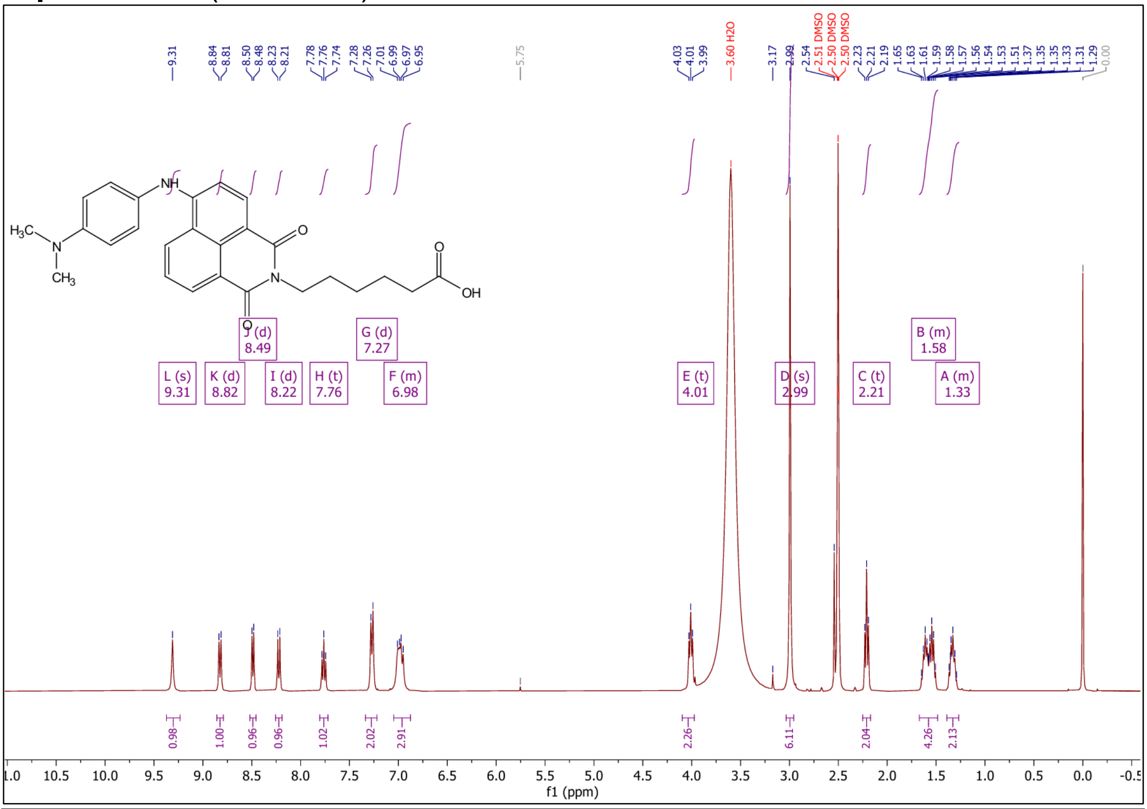

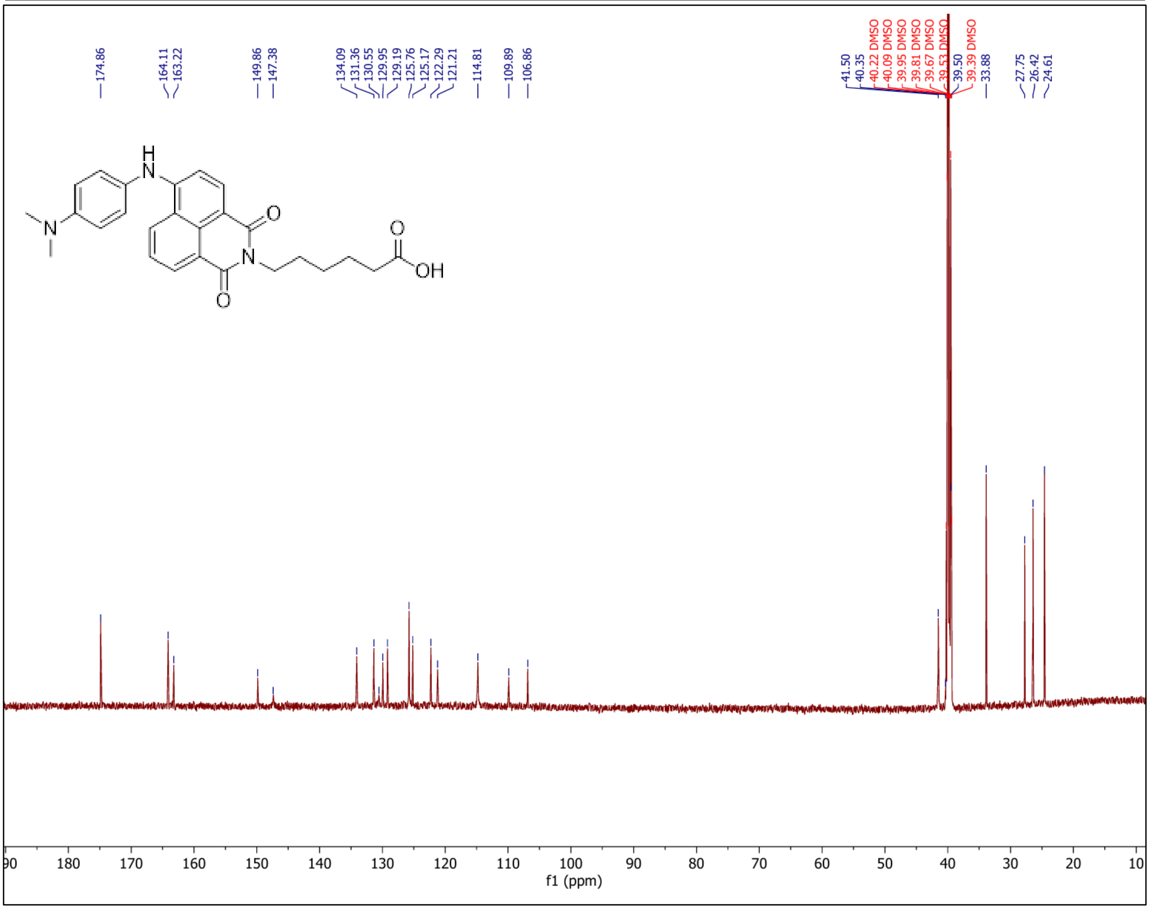

compound A12 (DMSO-d6)

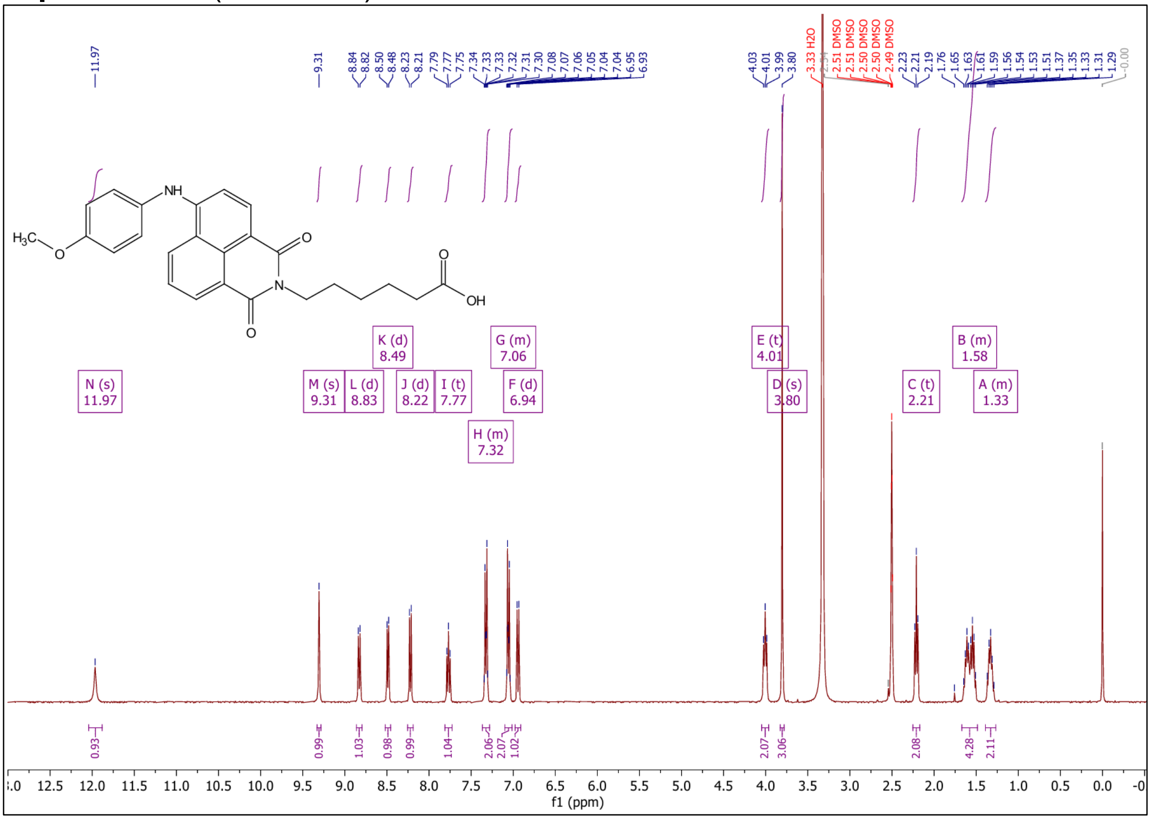

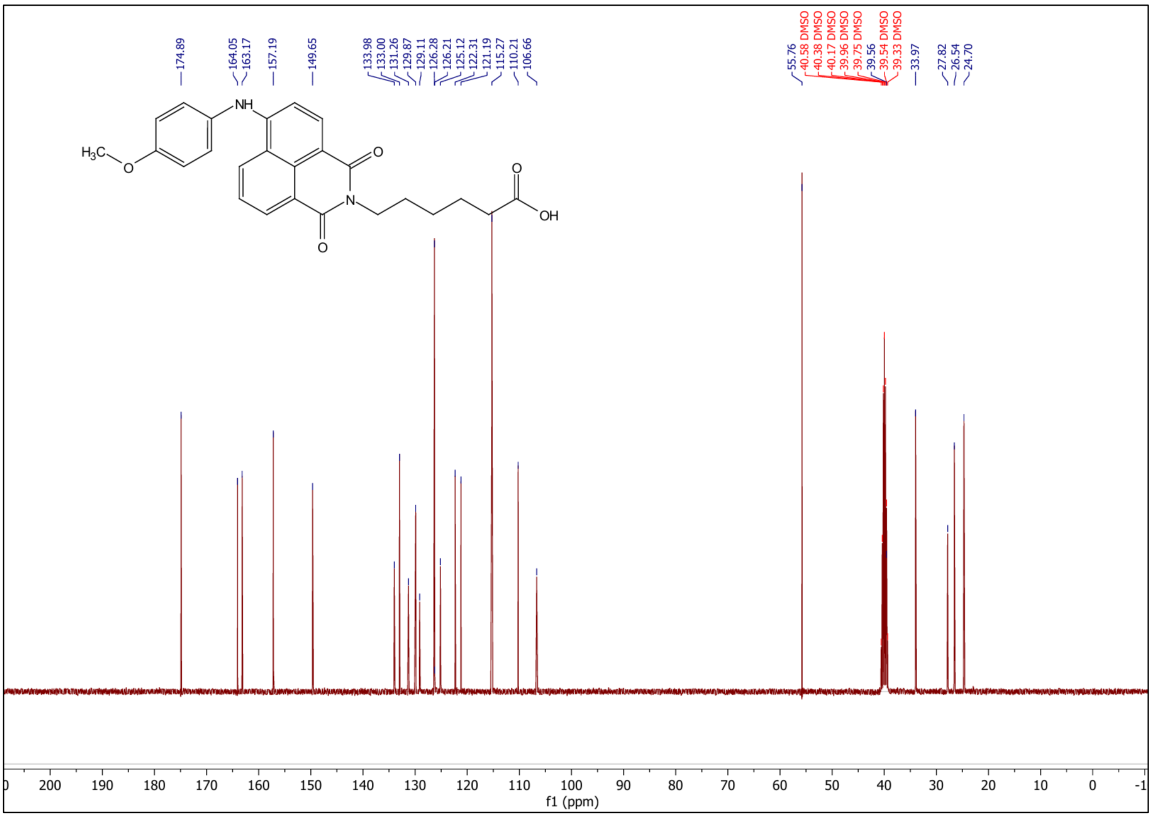

compound A13 (DMSO-d6)

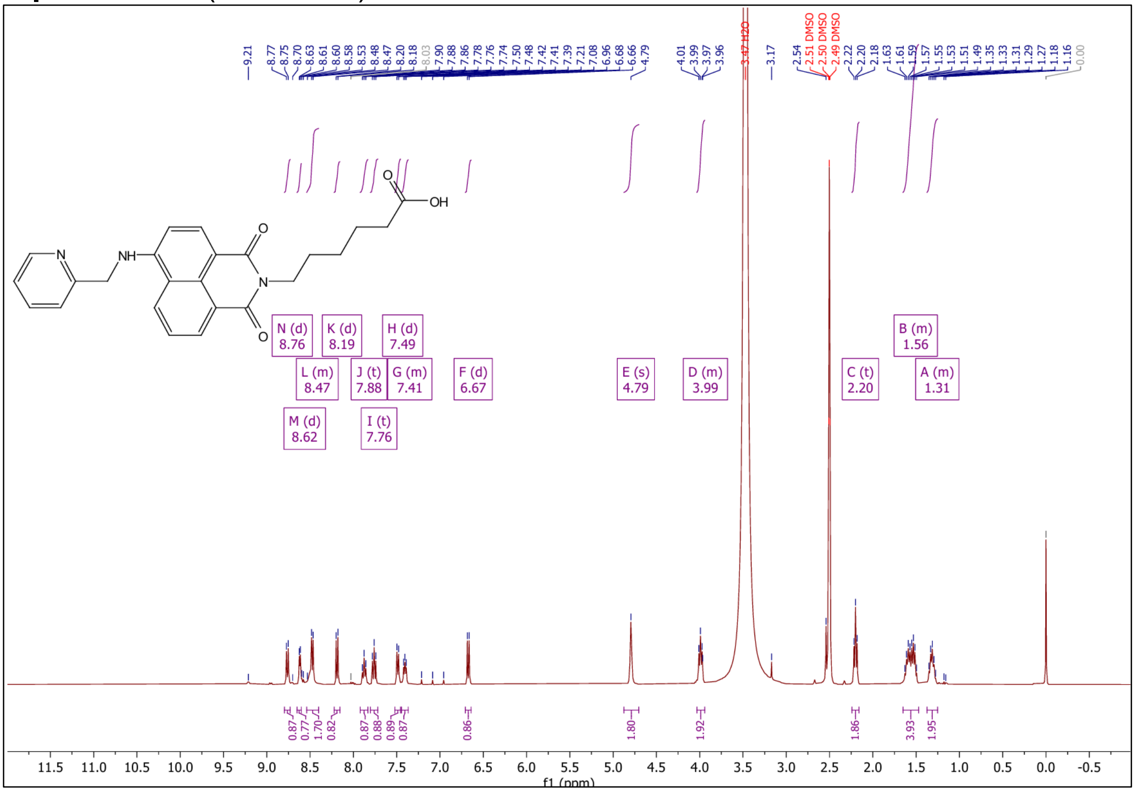

compound A14 (DMSO-d6)

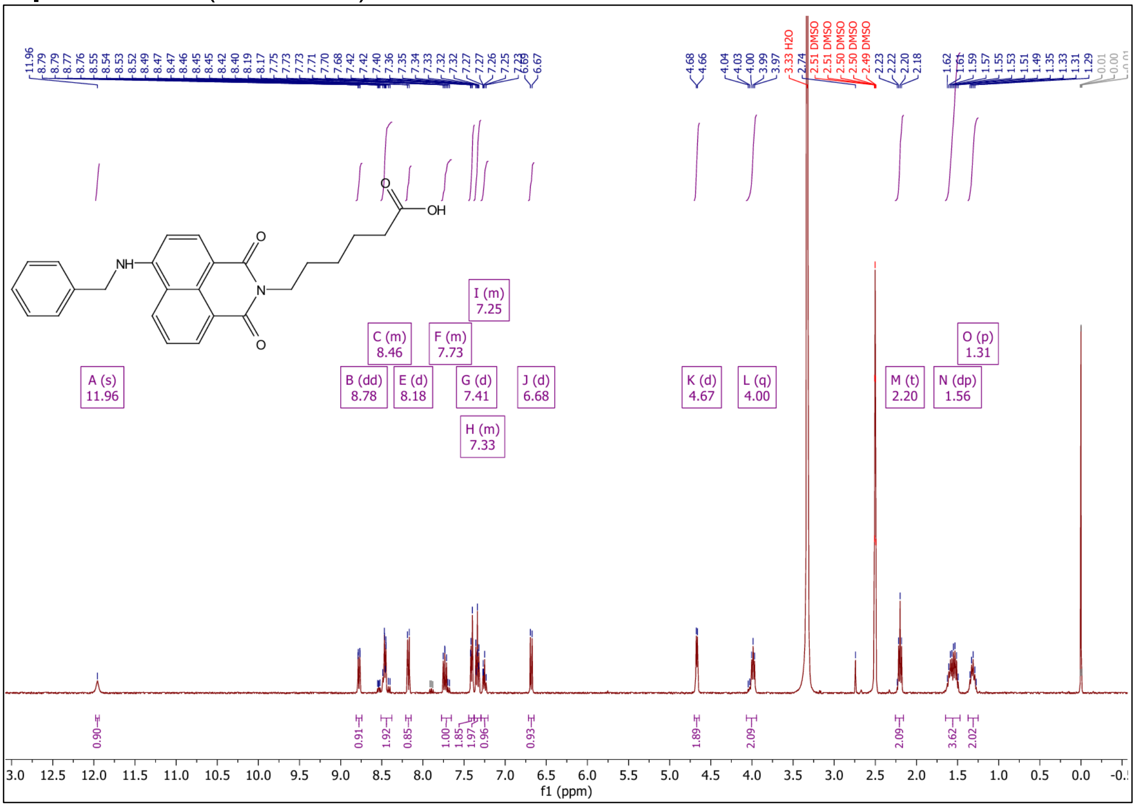

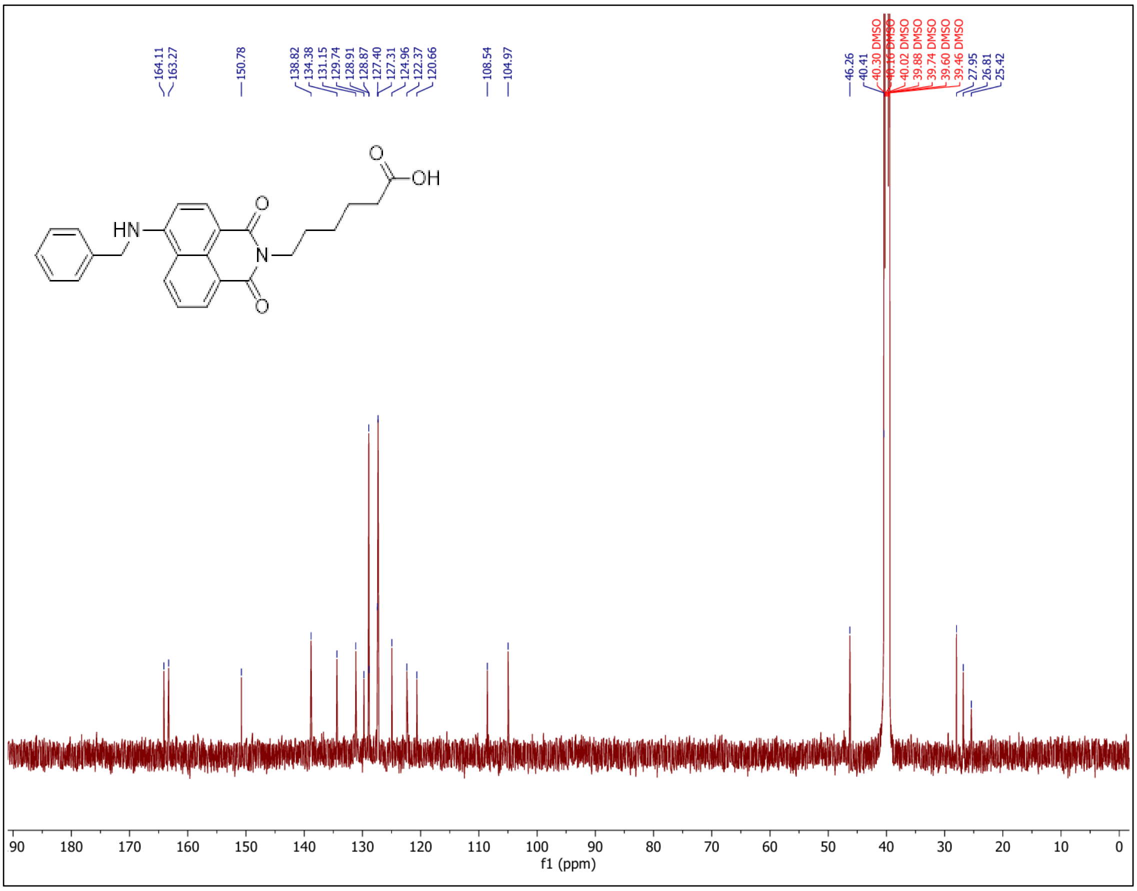

compound A15 (DMSO-d6)

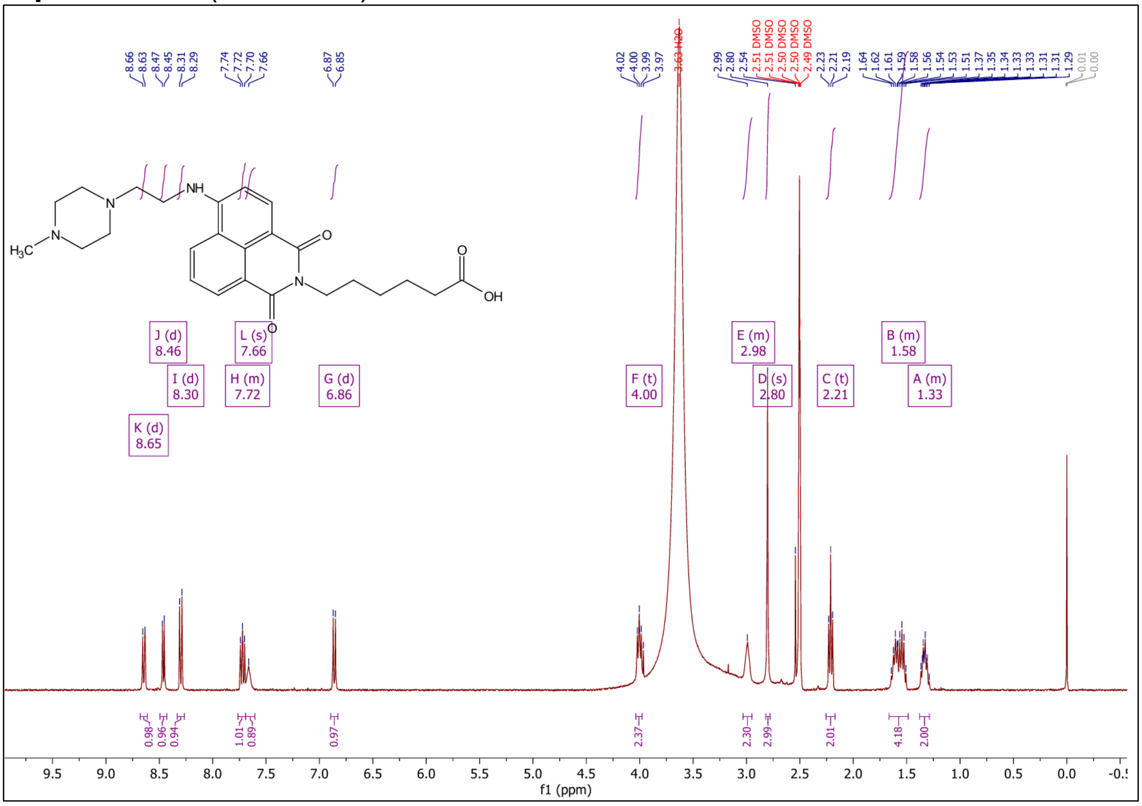

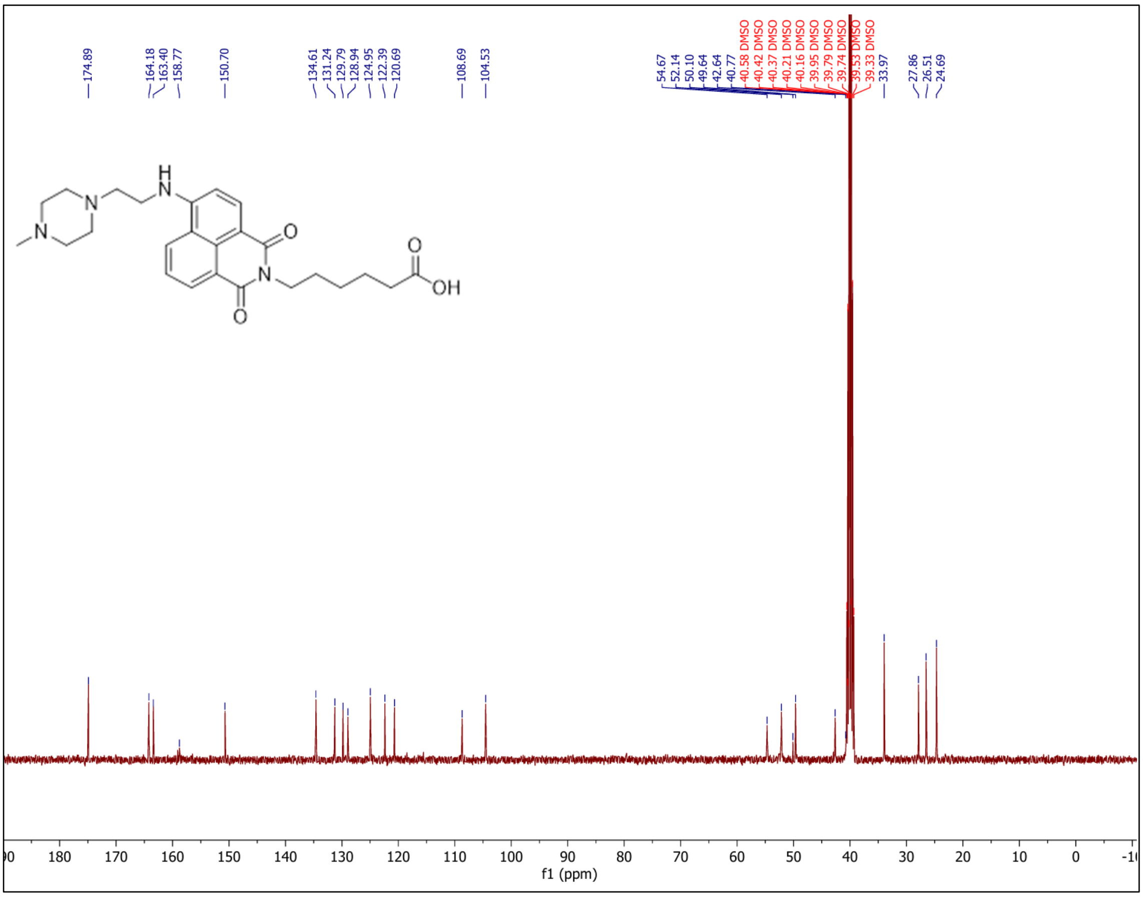

compound A16 (DMSO-d6)

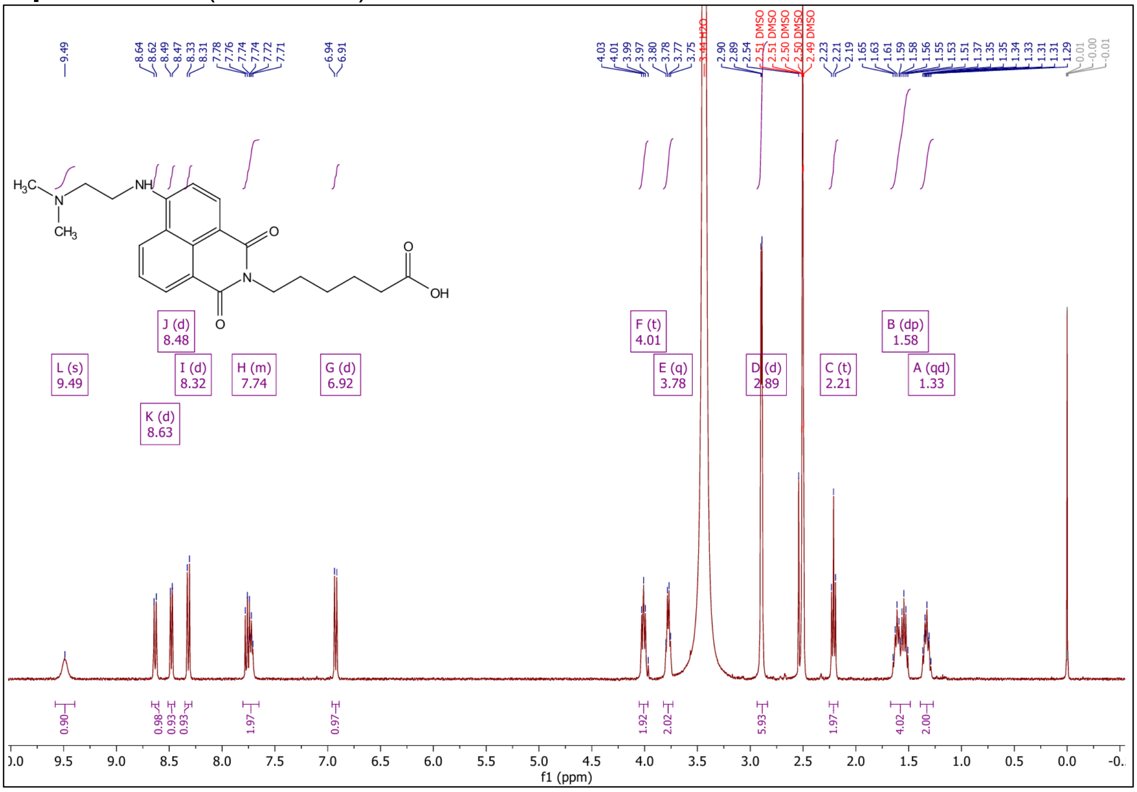

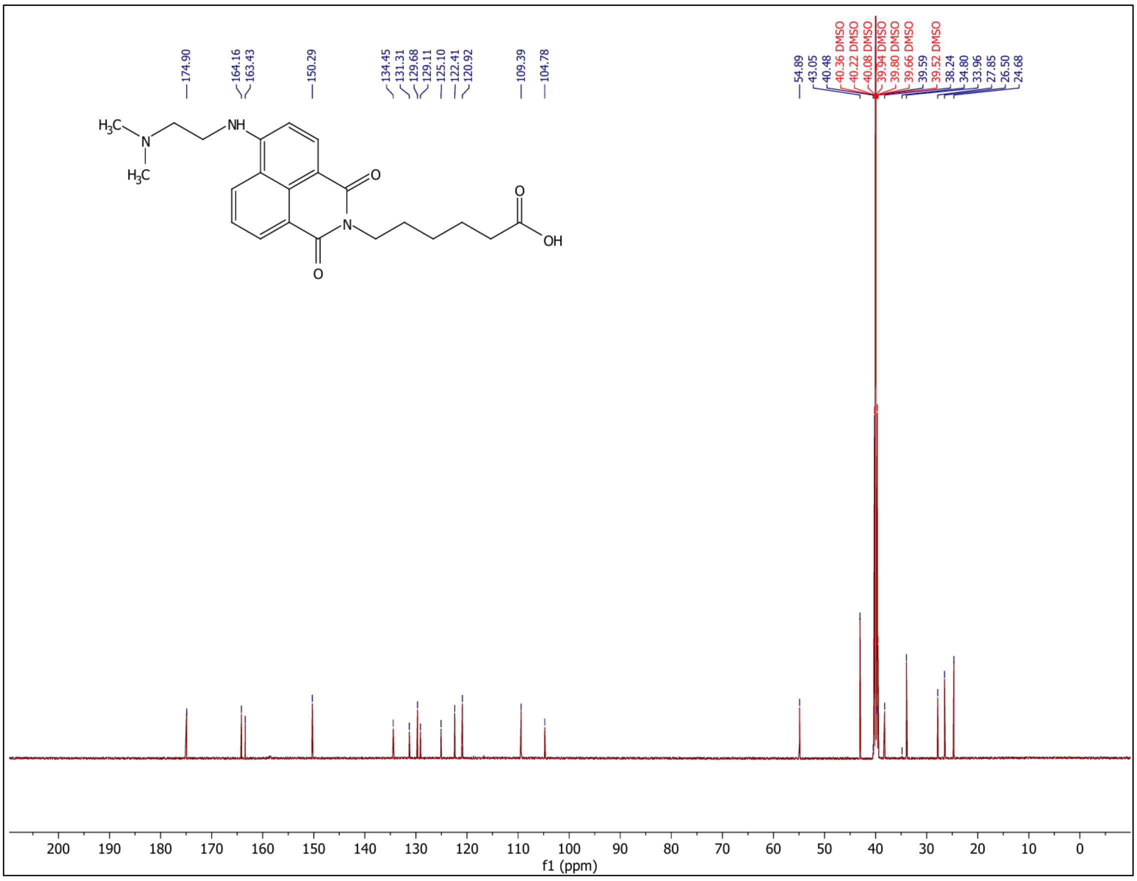

compound A17 (DMSO-d6)

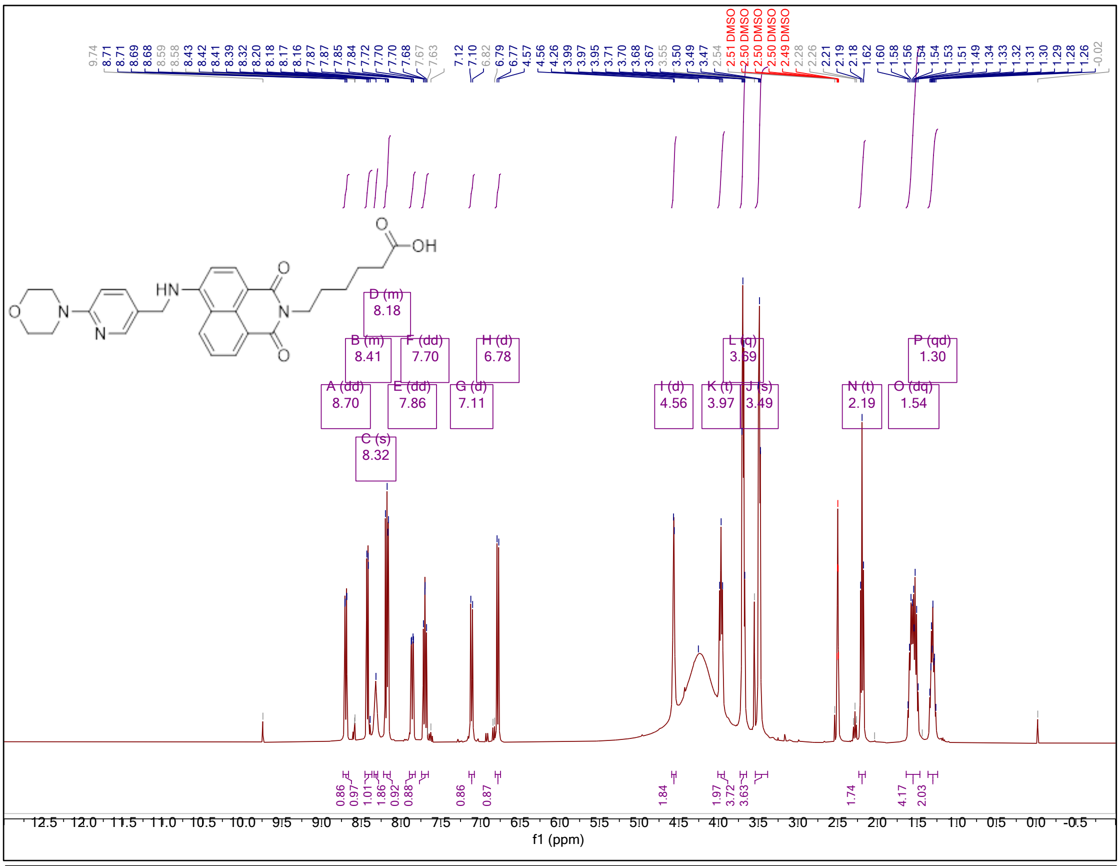

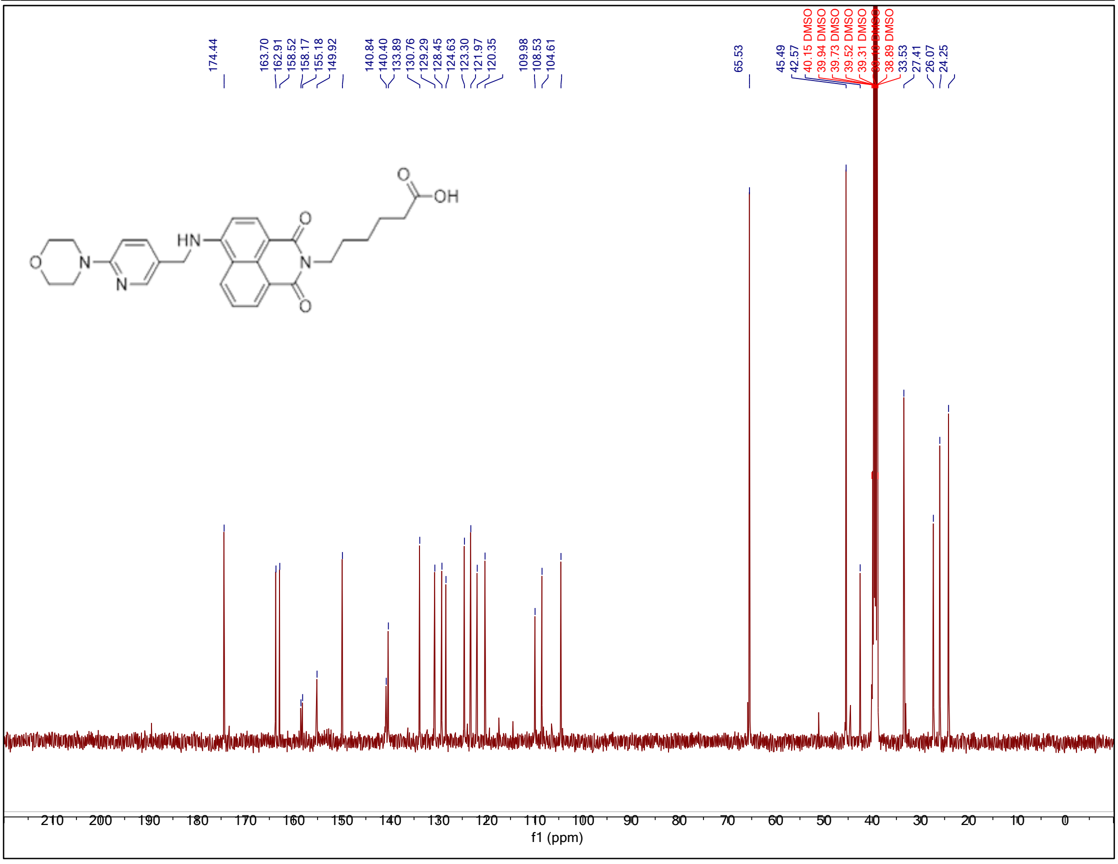

compound A18 (DMSO-d6)

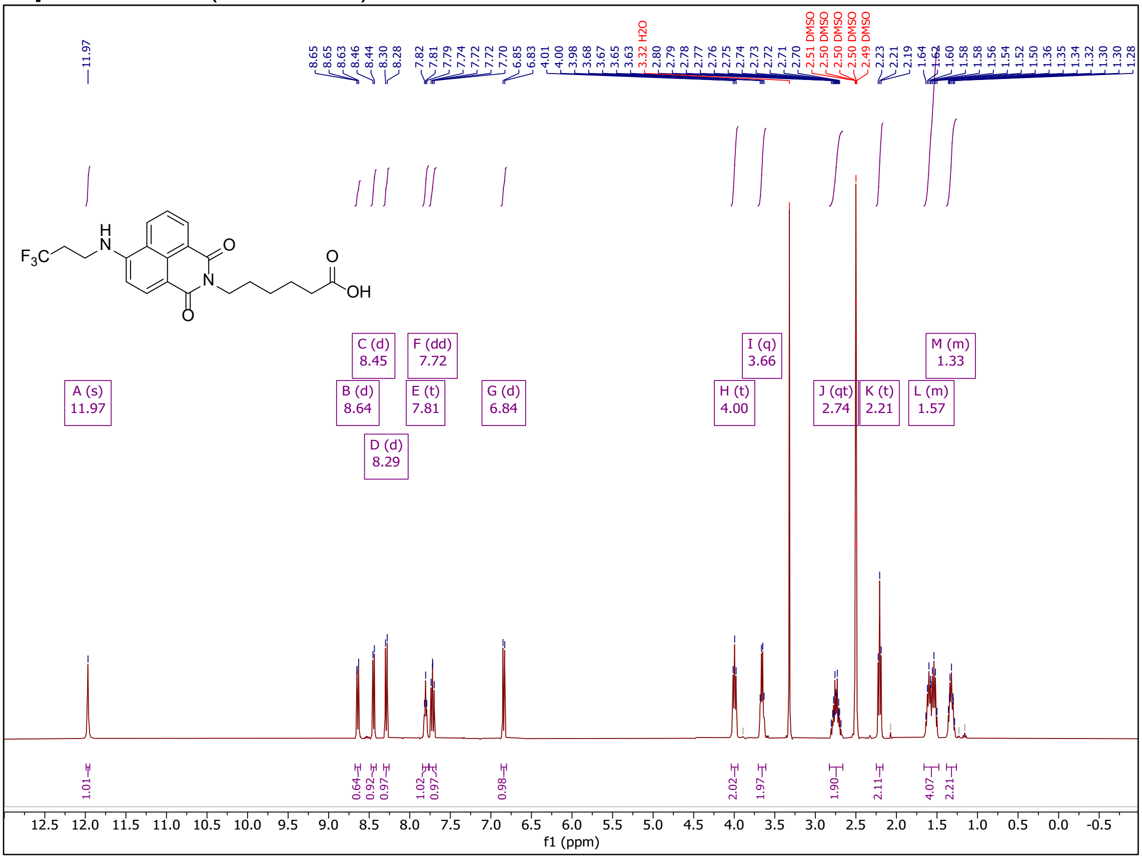

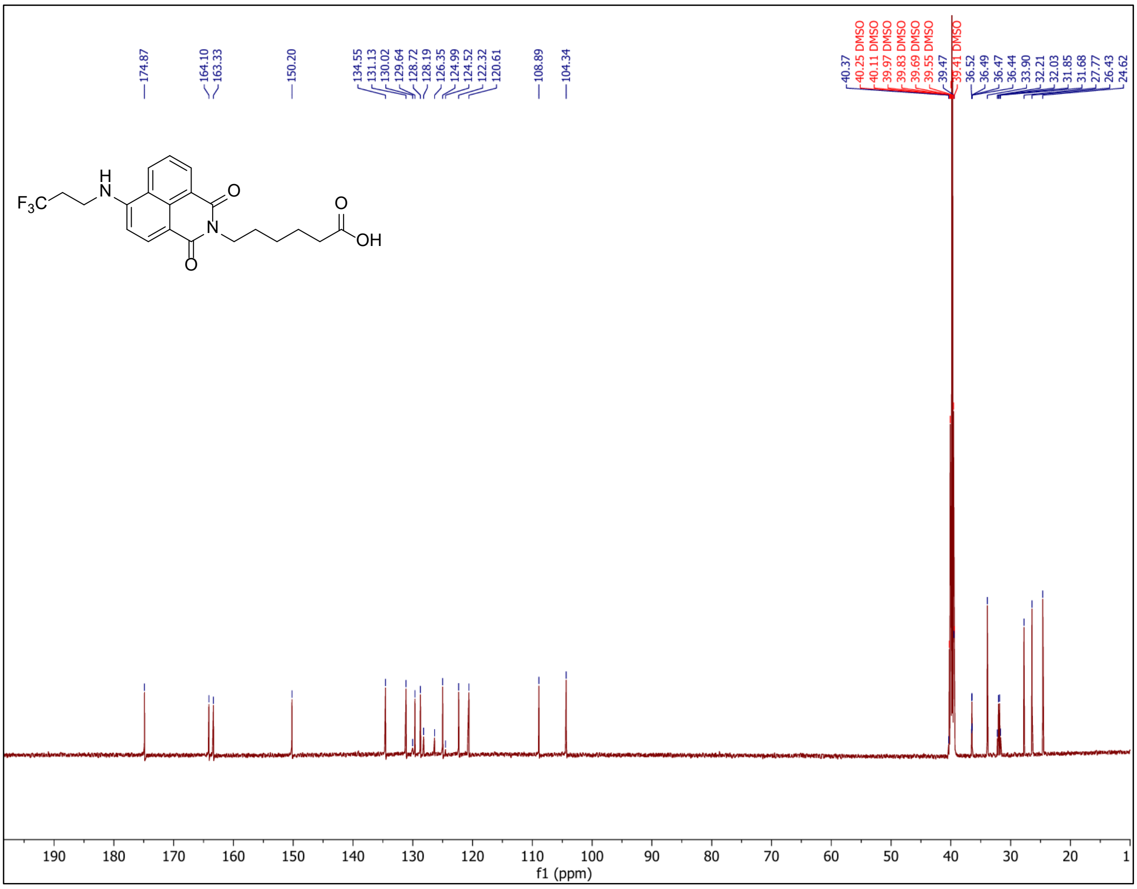

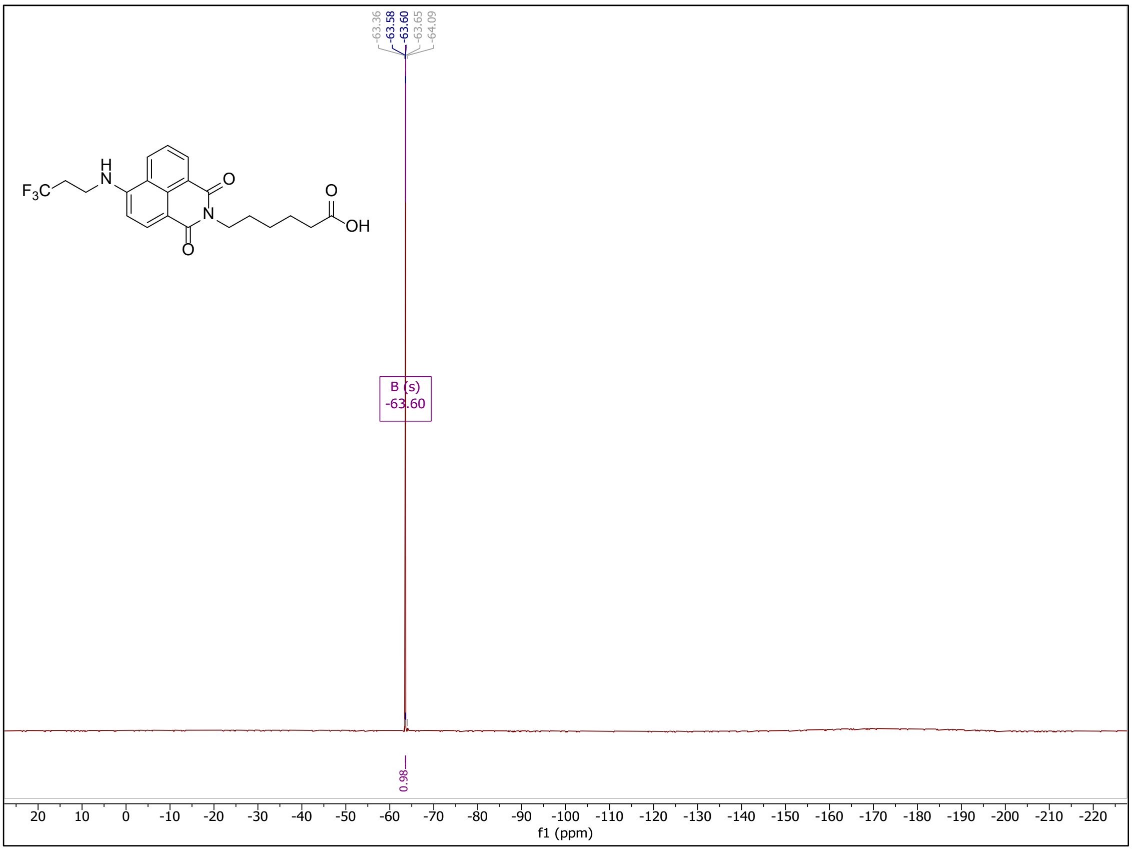
-63.36
-63.58
-63.60
-63.65
-64.09
B (s)
-63.60
0.98
20 10 0 -10 -20 -30 -40 -50 -60 -70 -80 -90 -100 -110 -120 -130 -140 -150 -160 -170 -180 -190 -200 -210 -220
f1 (ppm)

compound A19 (DMSO-d6)

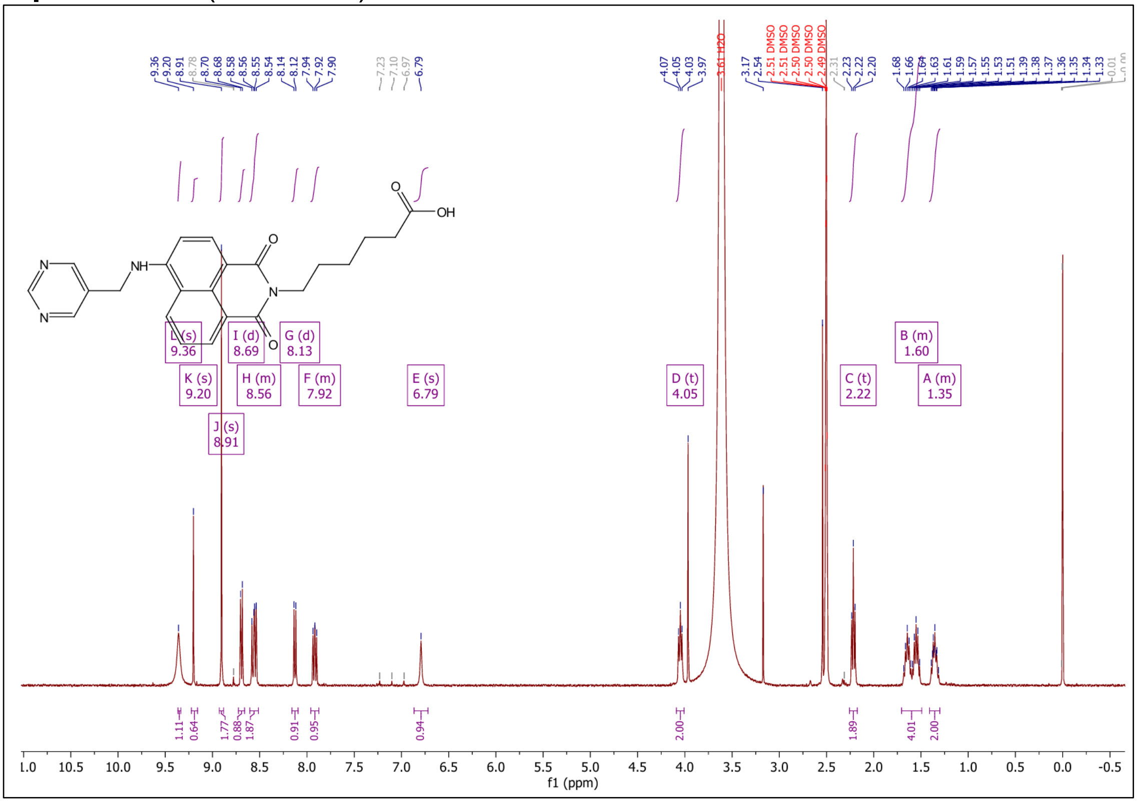

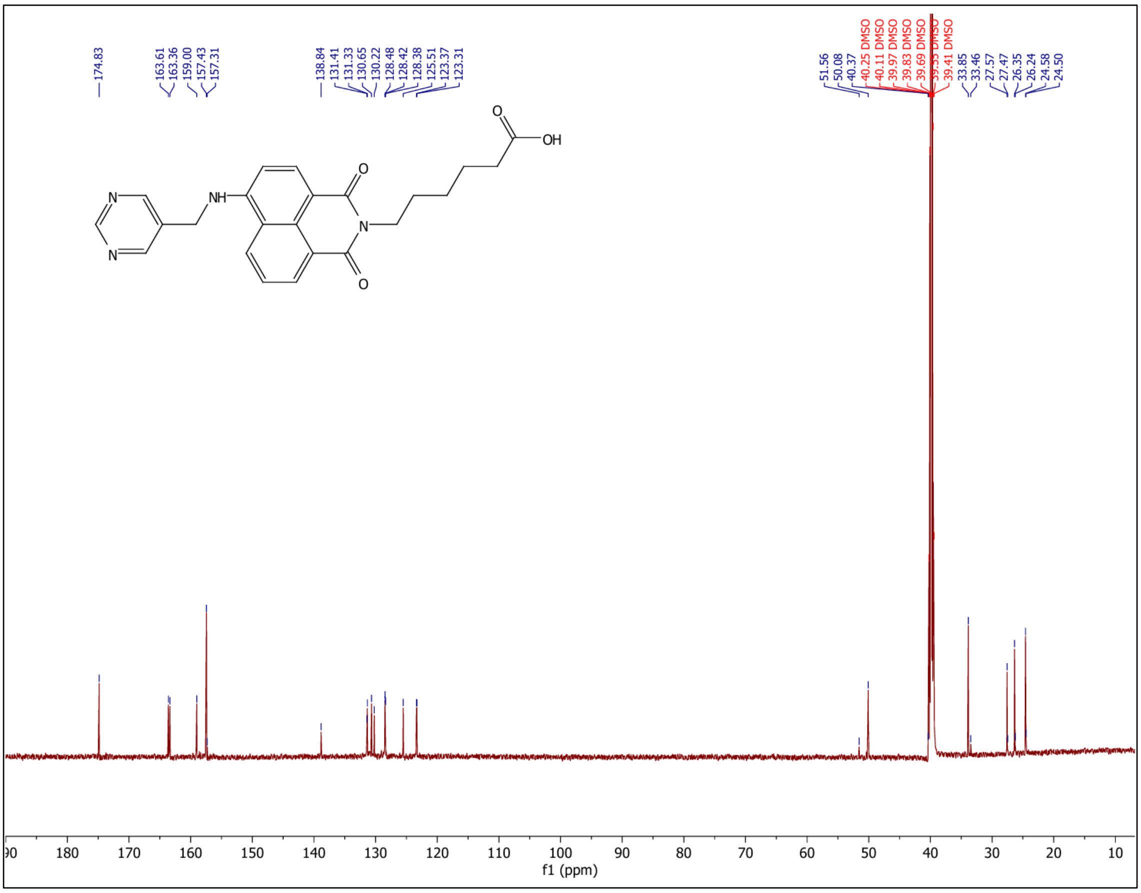

compound A20 (DMSO-d6)

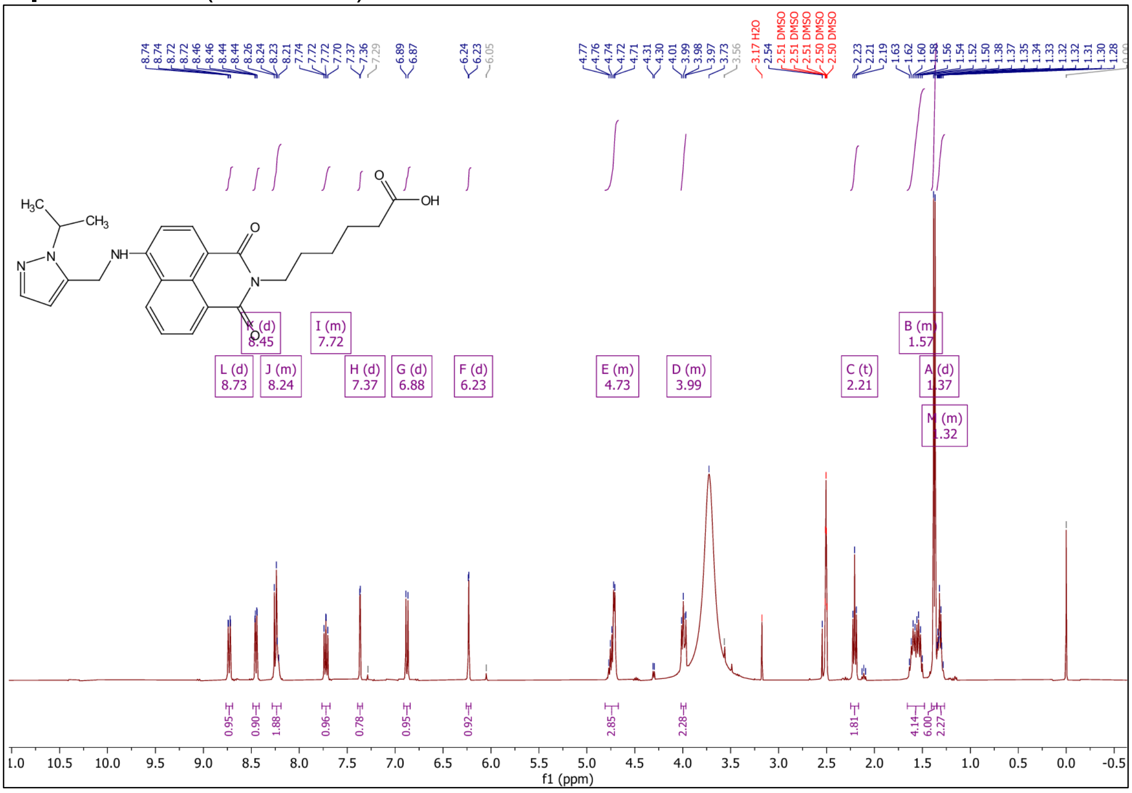

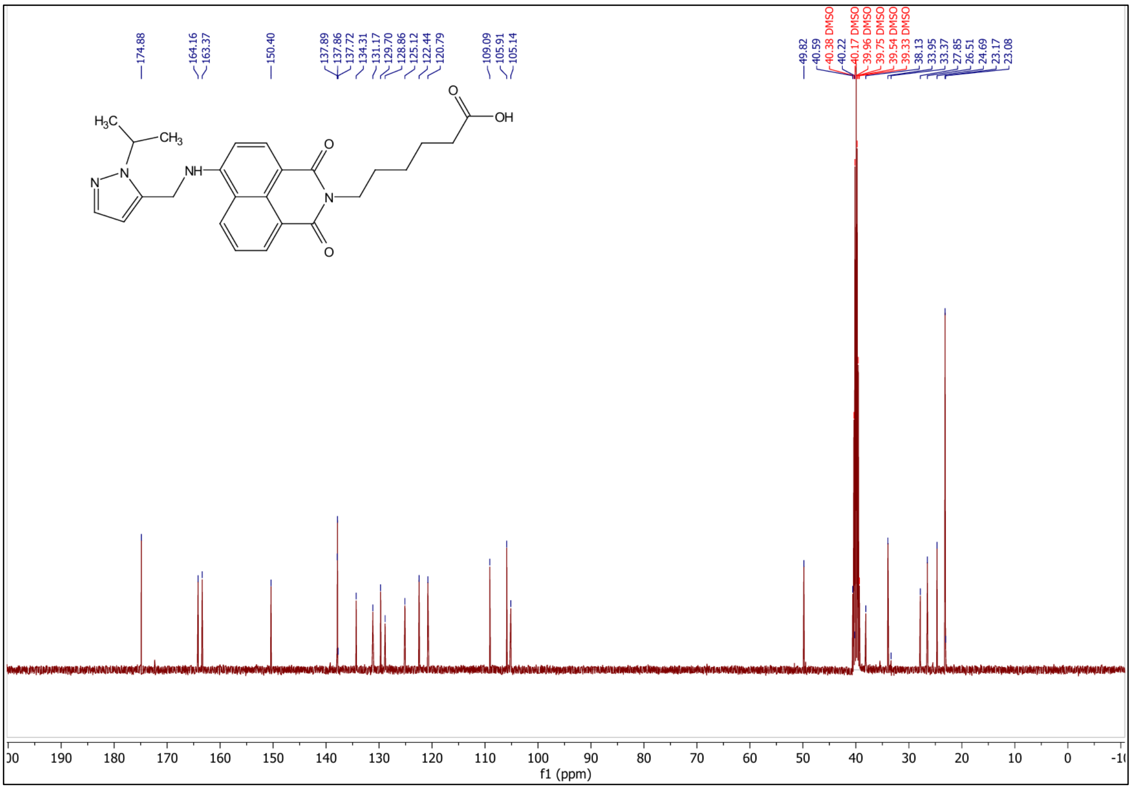

compound A21 (DMSO-d6)

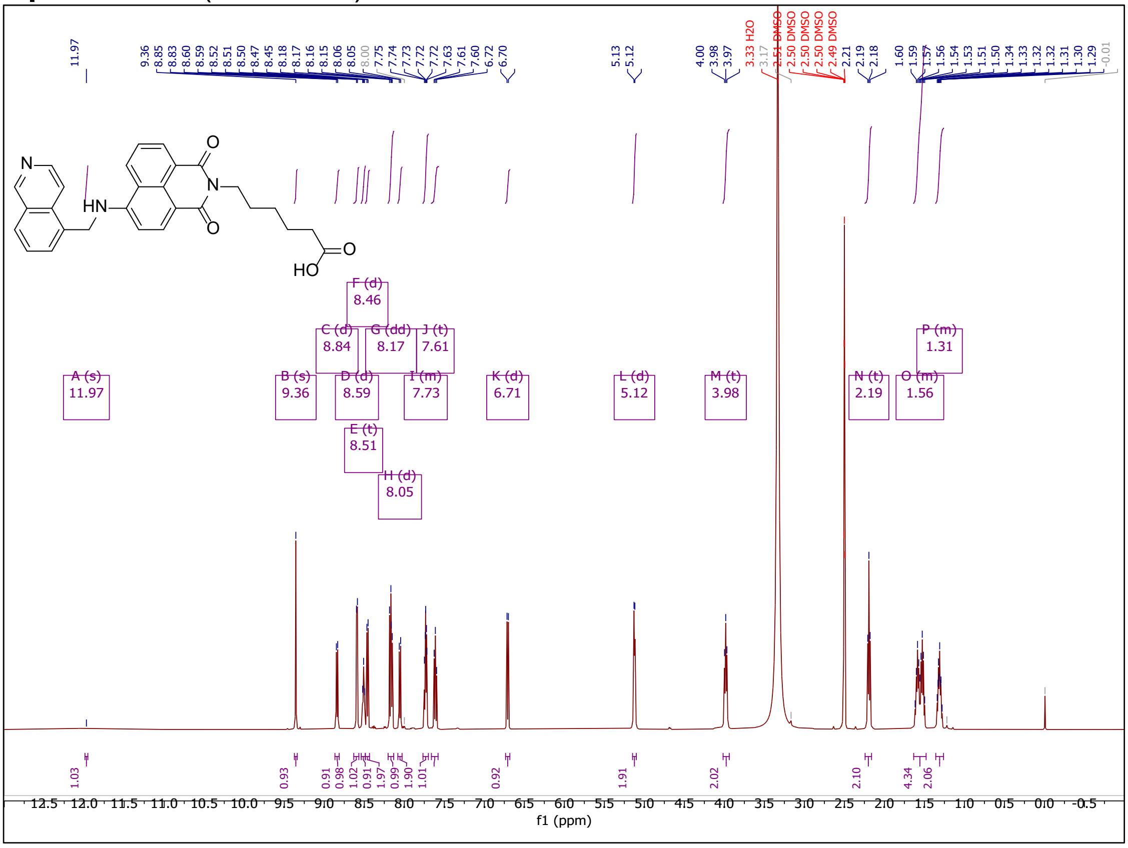

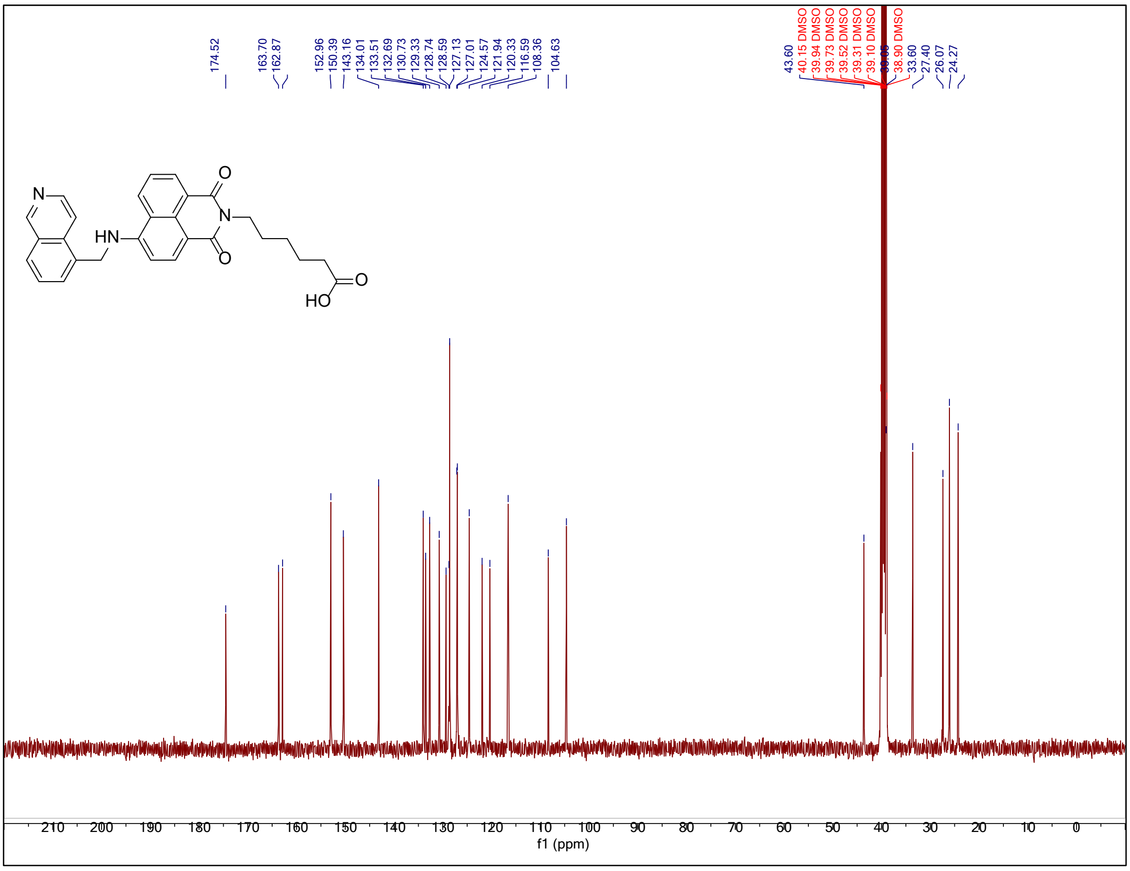

compound A22 (DMSO-d6)

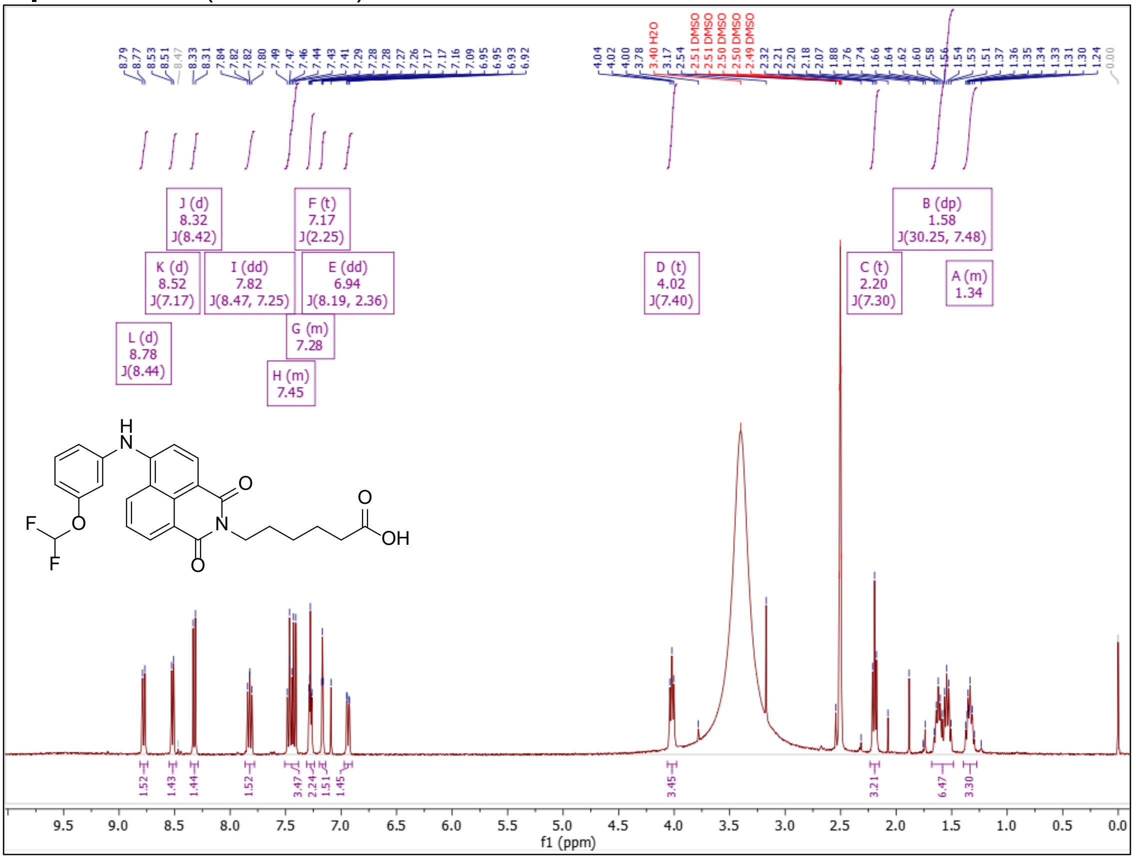

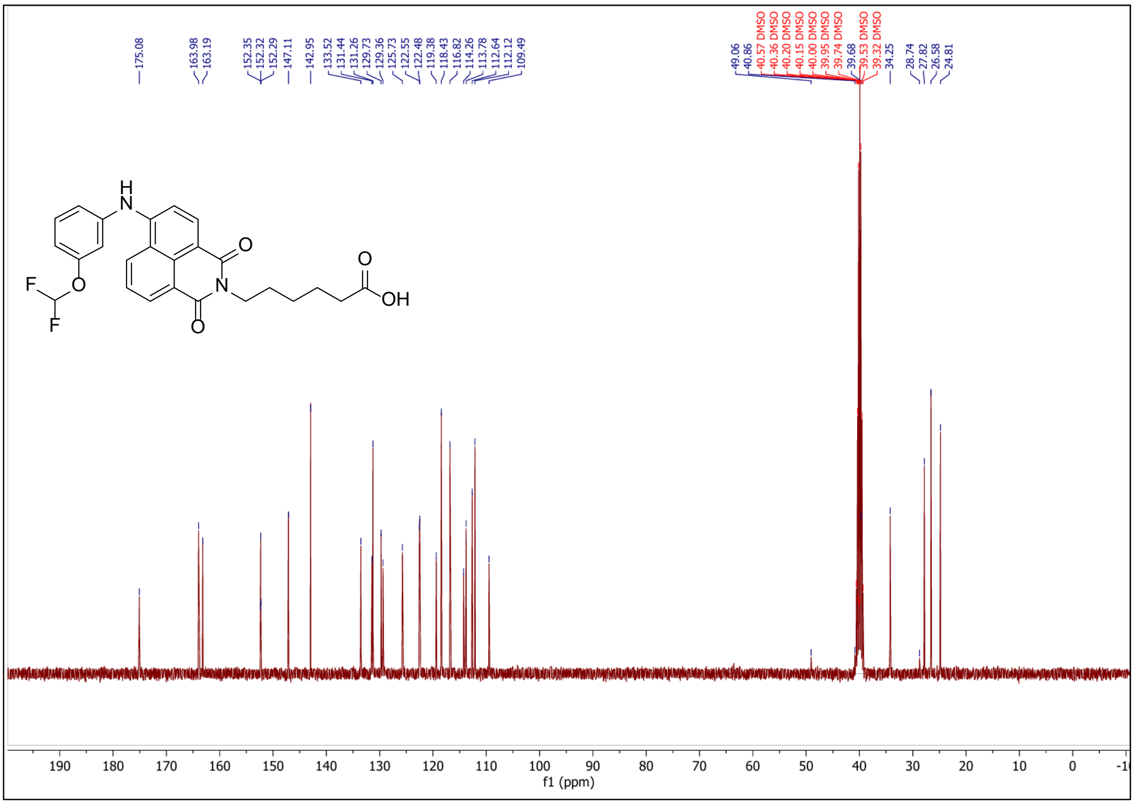

compound A23 (DMSO-d6)

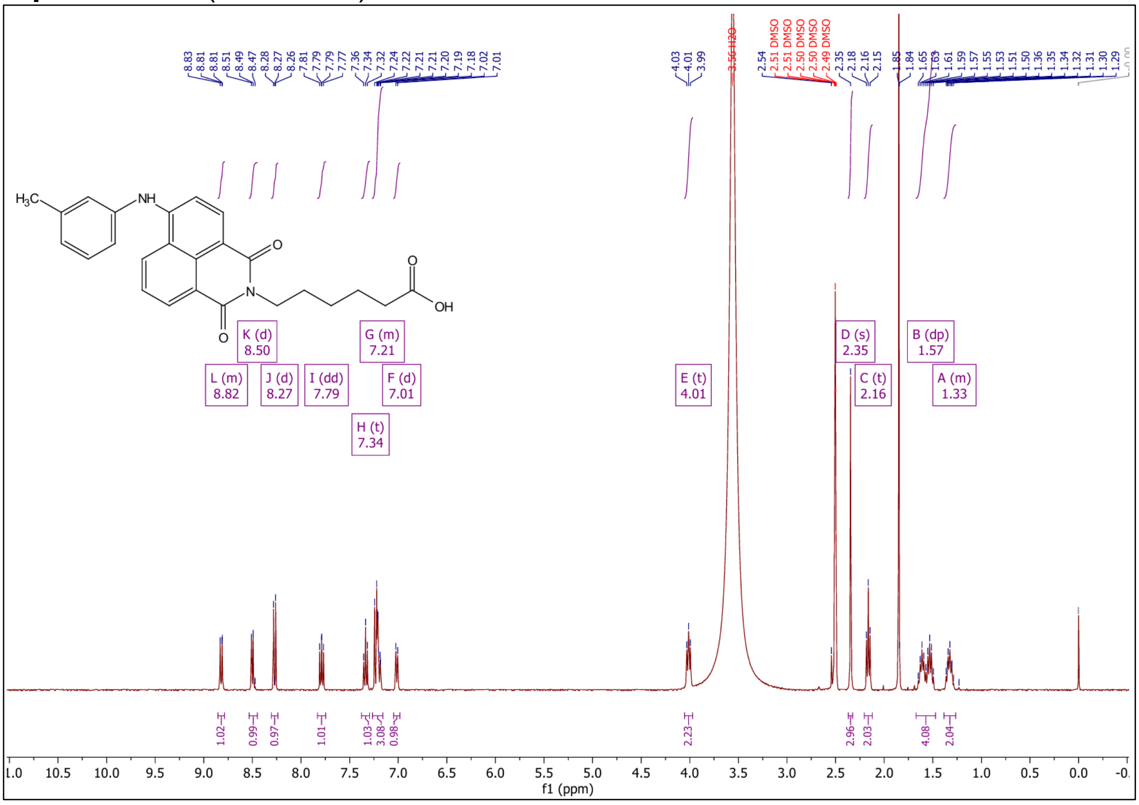

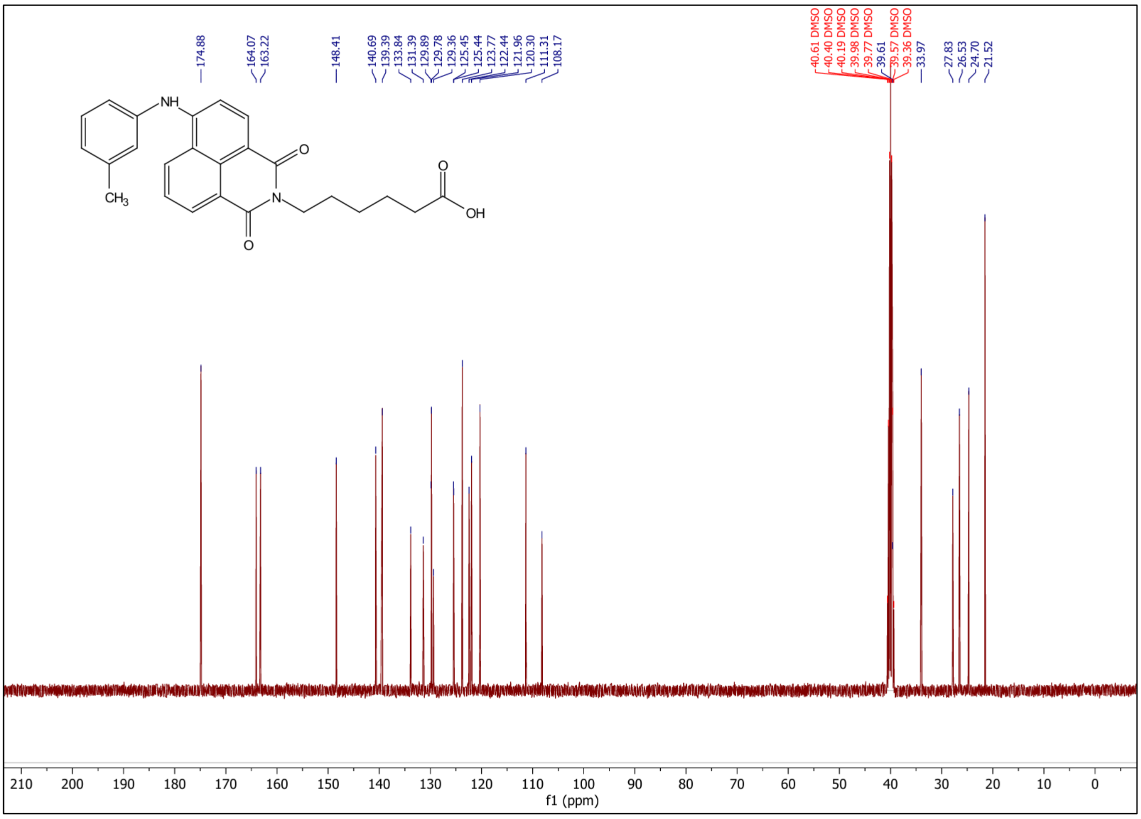

compound A24 (DMSO-d6)

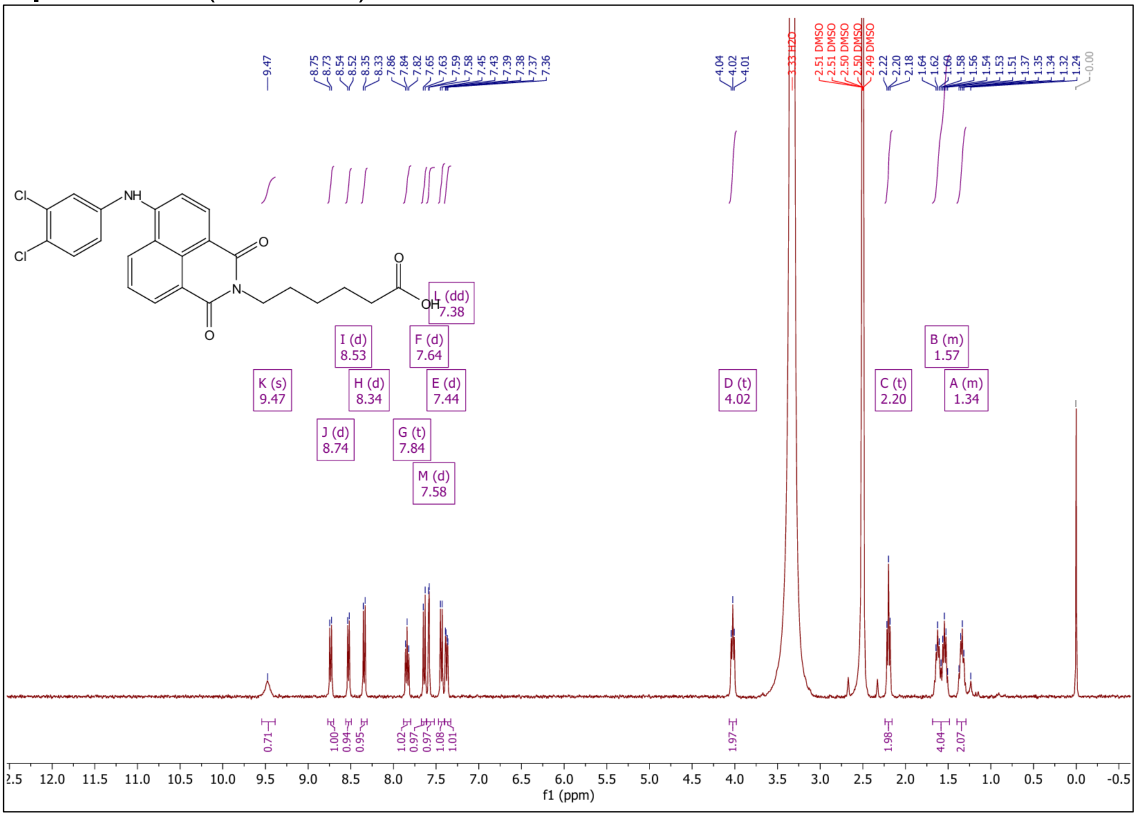

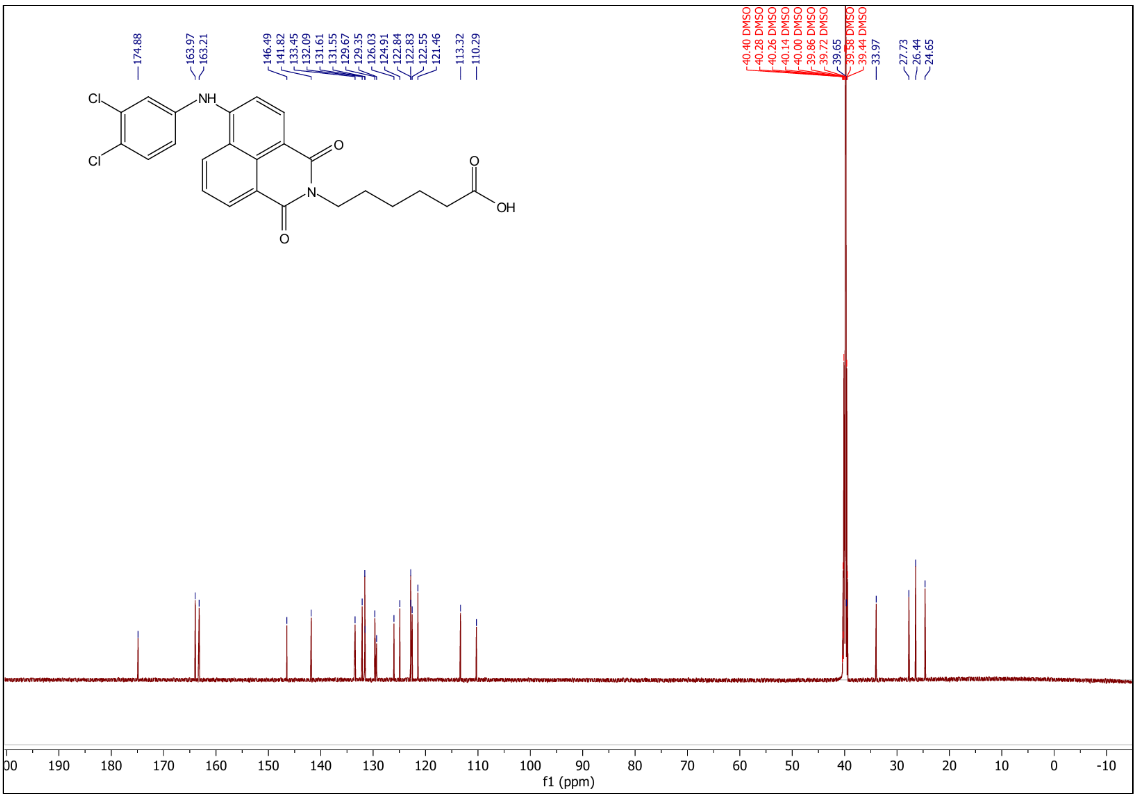

compound A25 (DMSO-d6)

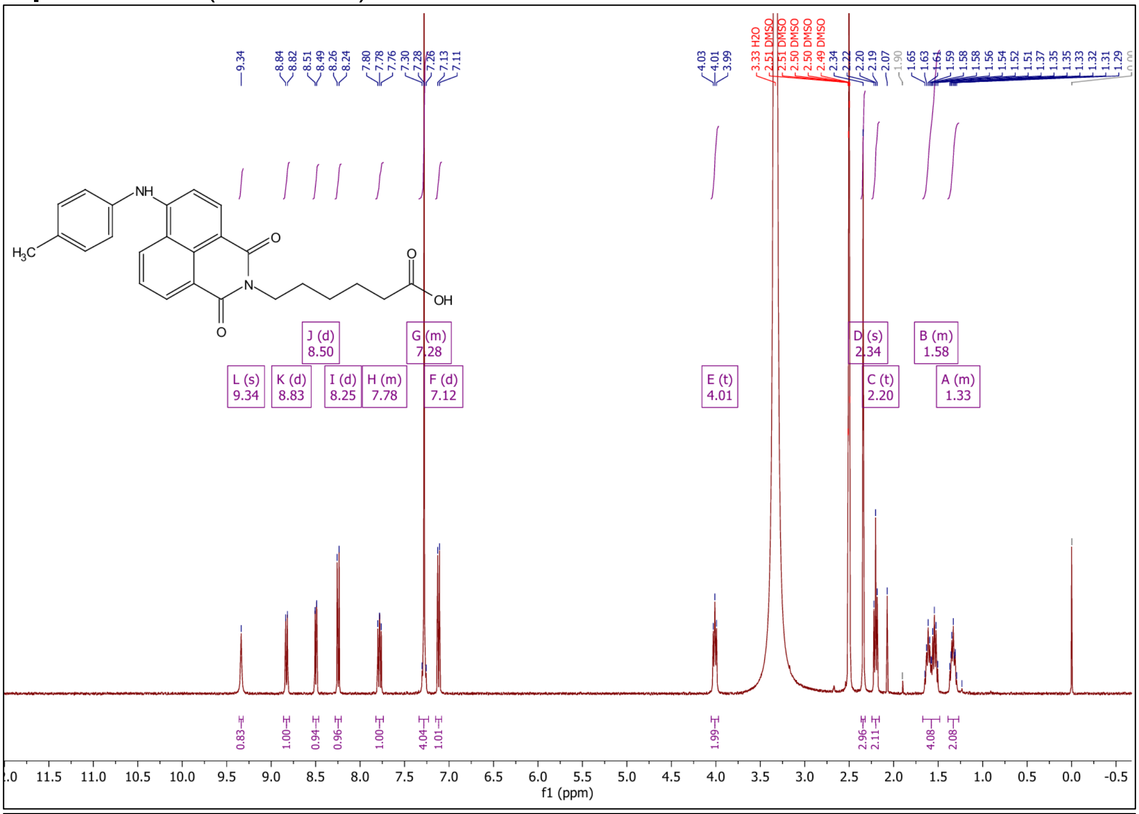

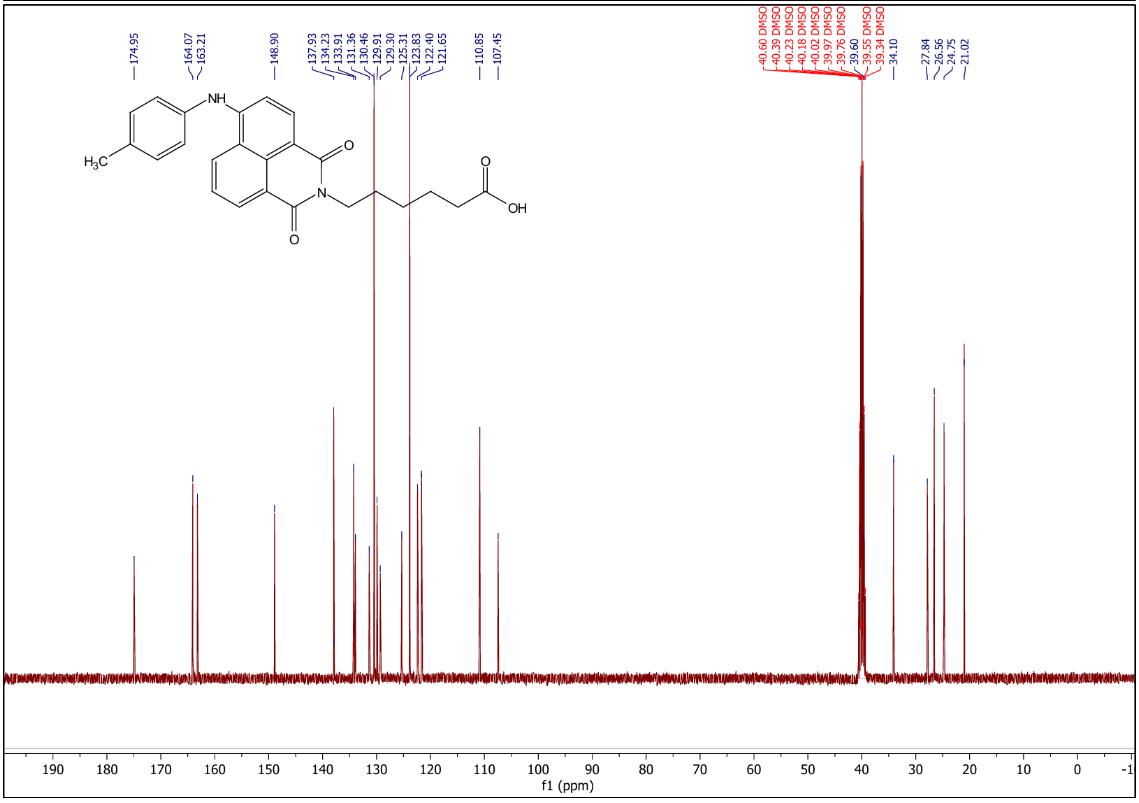

compound A26 (DMSO-d6)

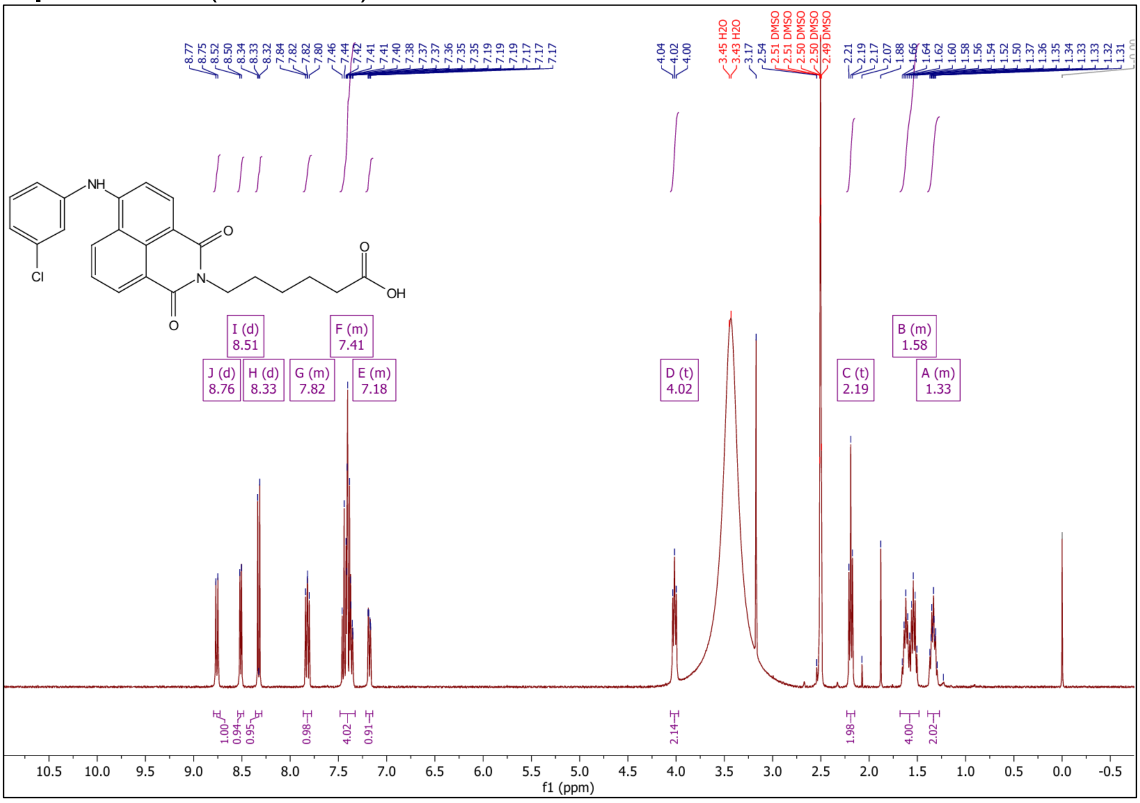

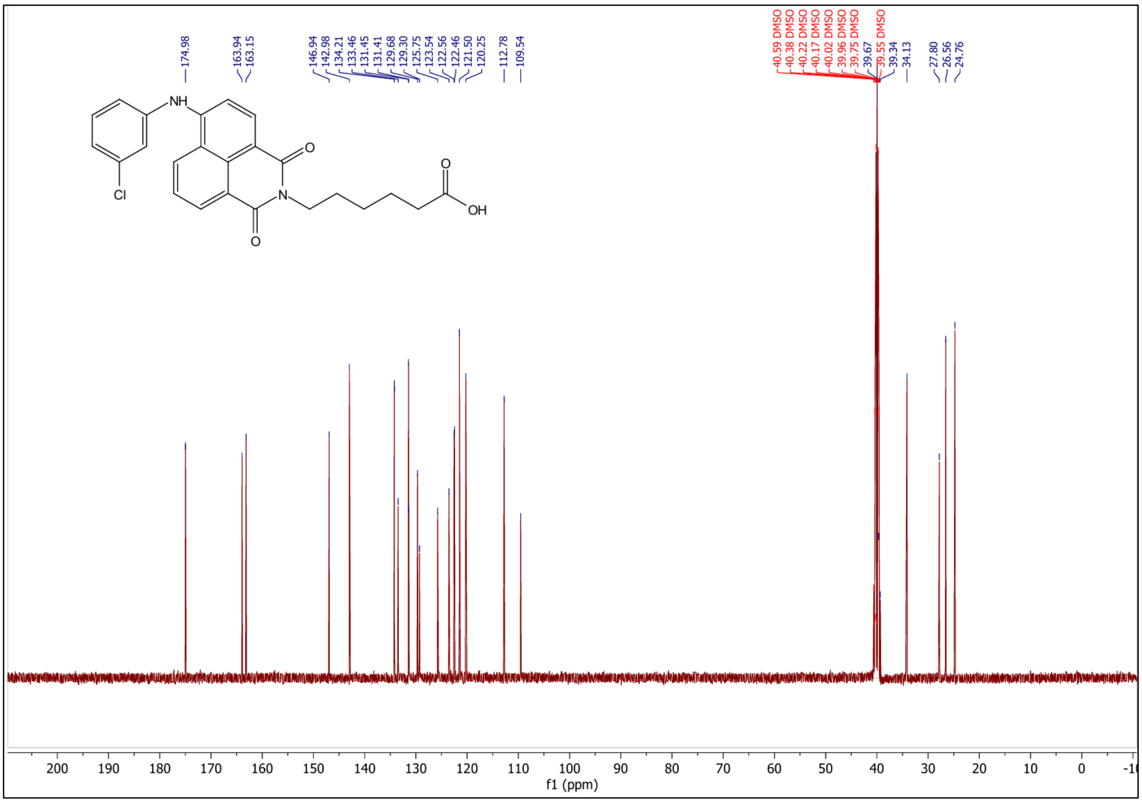

compound 4 (DMSO-d6)

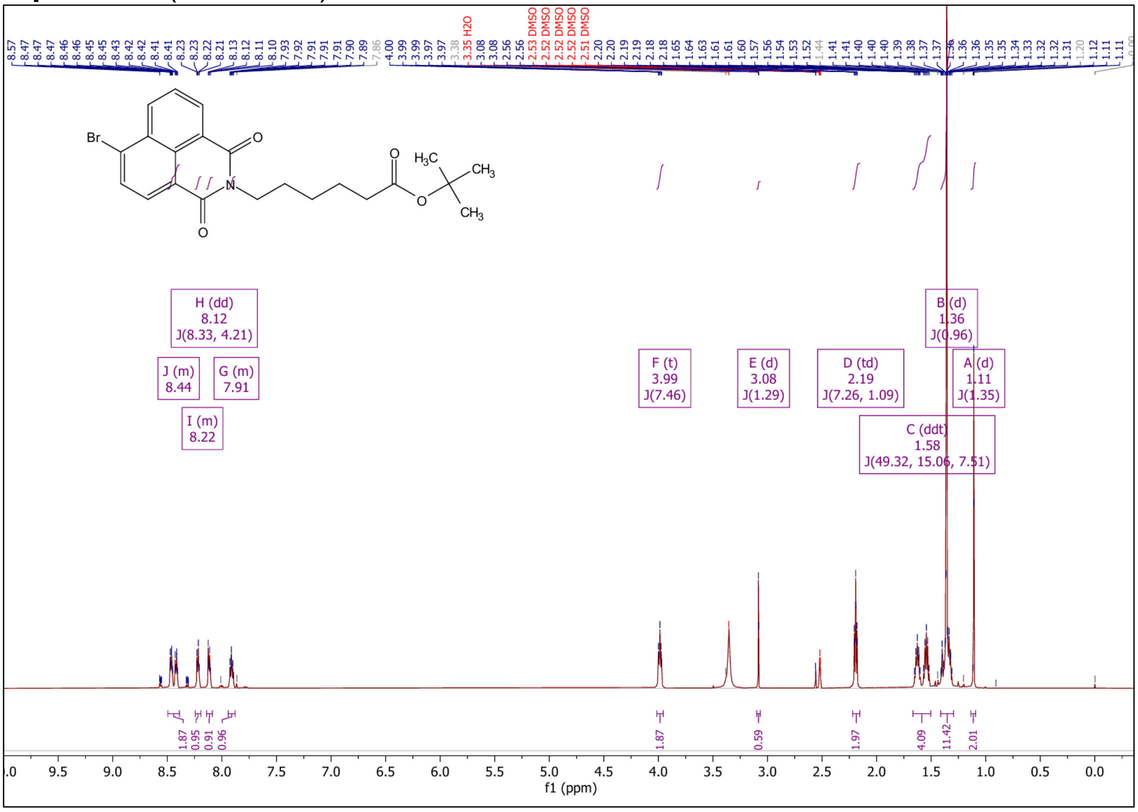

compound 5 (DMSO-d6)

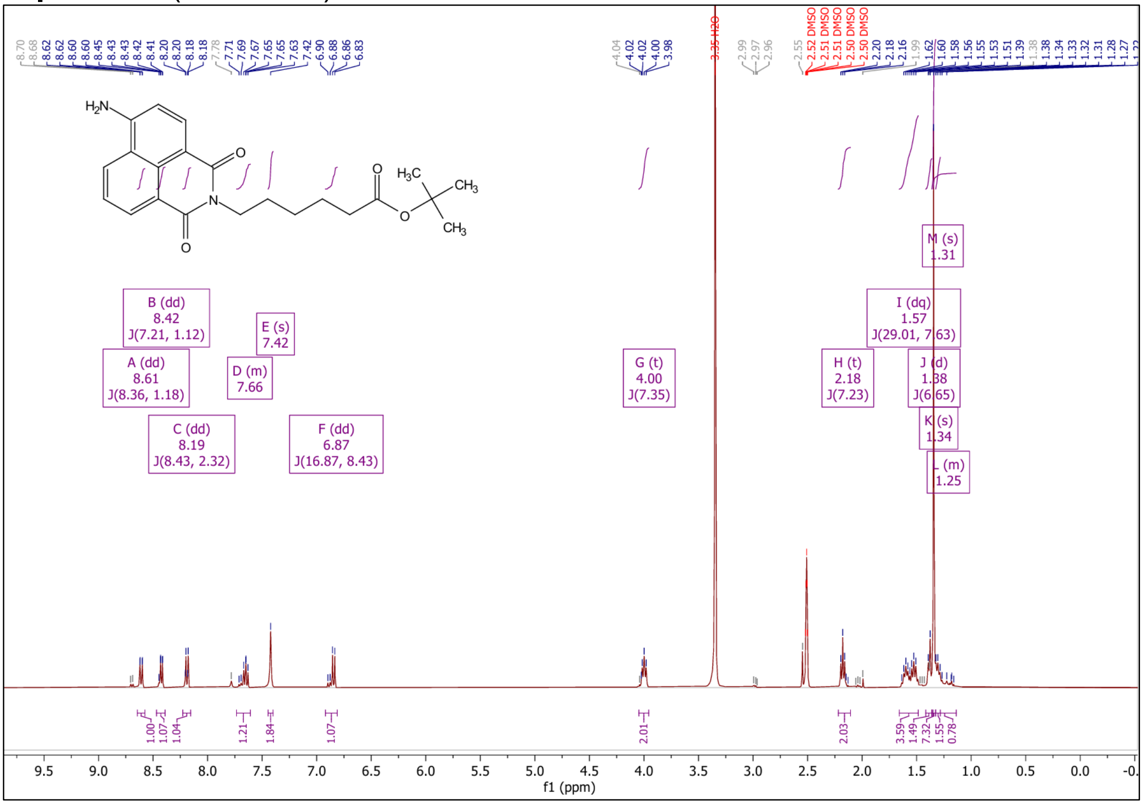

compound A17(tBu) (CDCl$_3$)

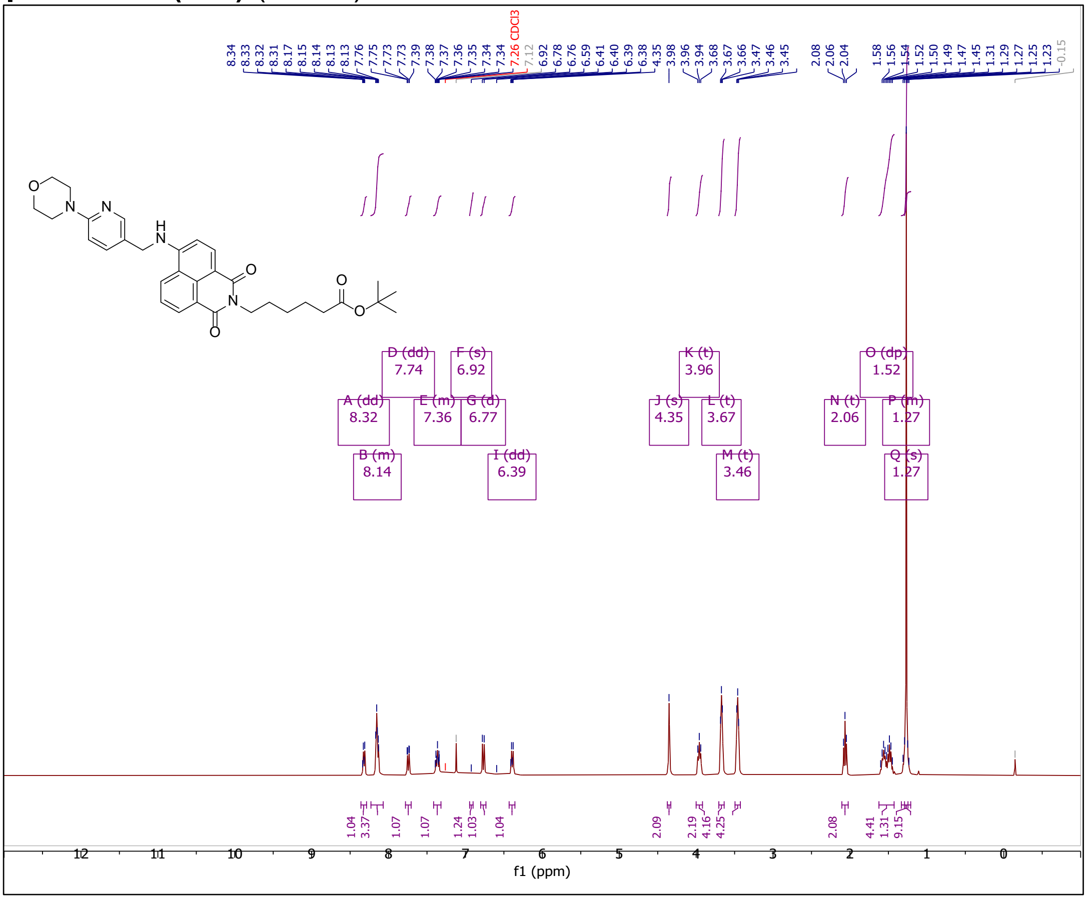

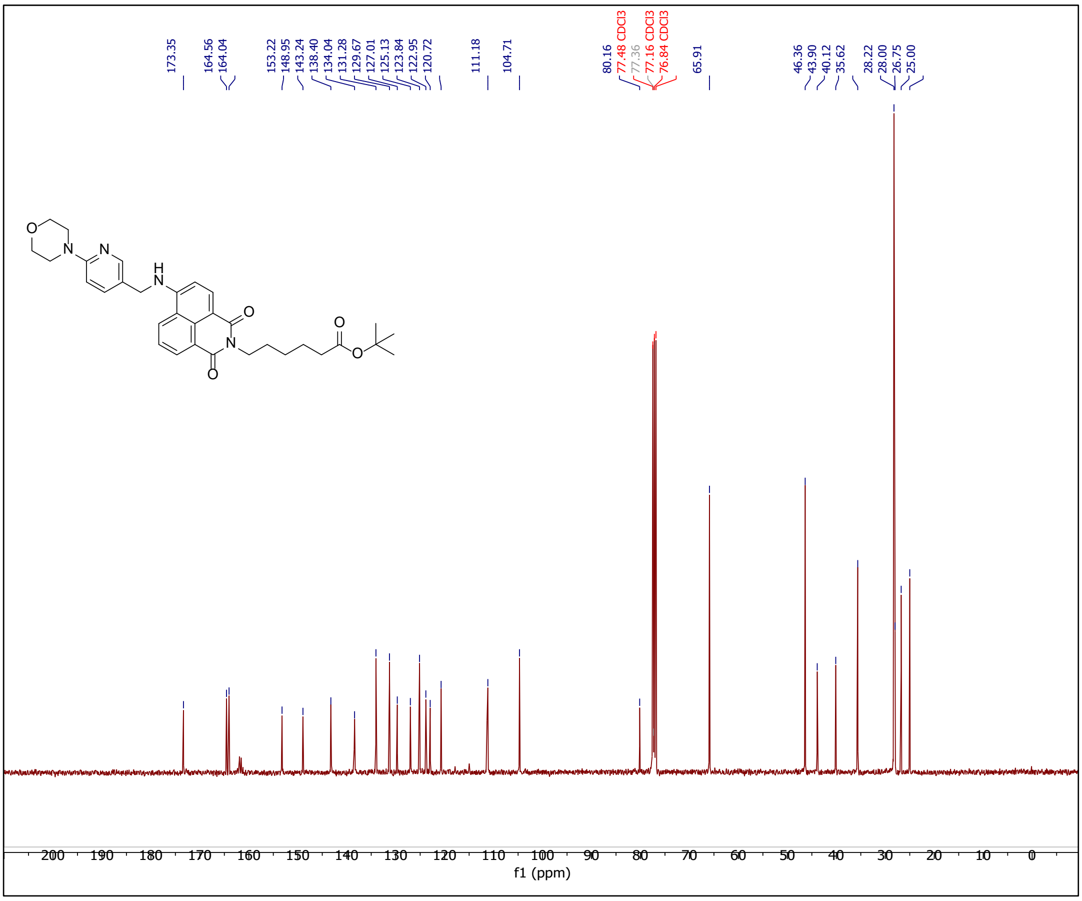

compound A17-C(tBu) (DMSO-d6)

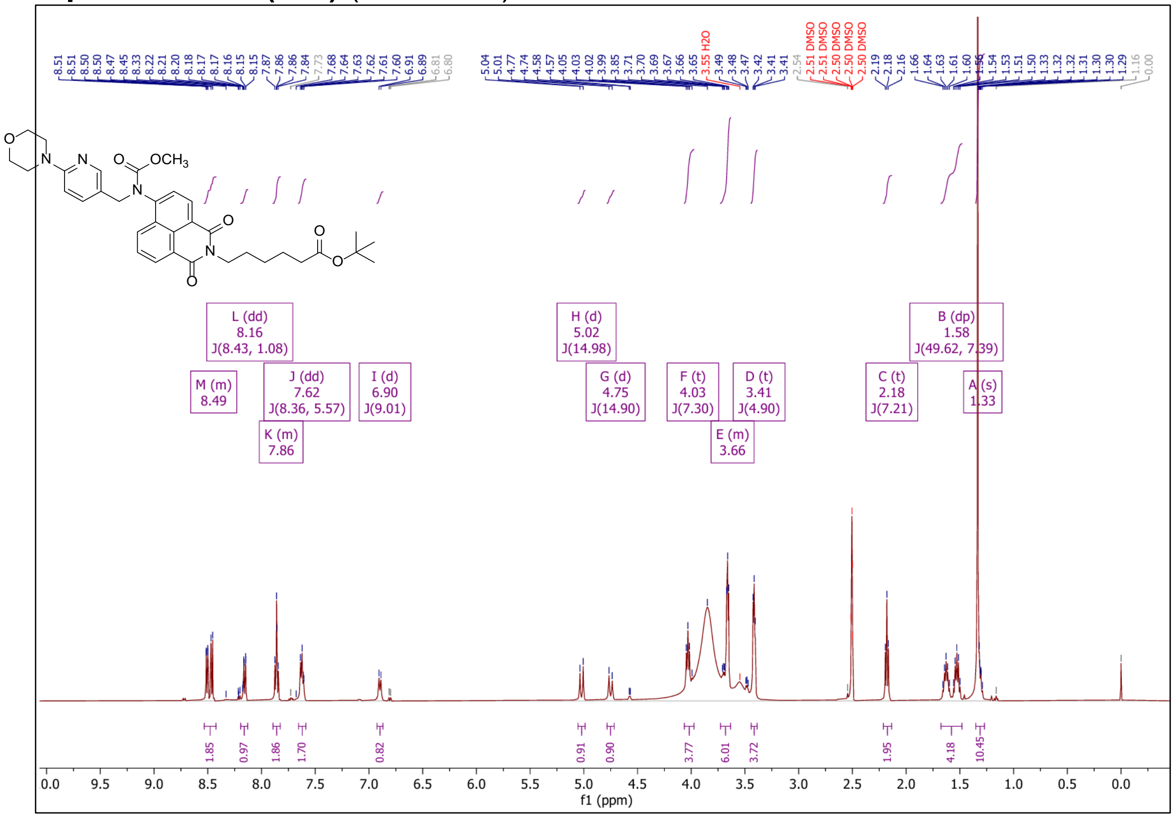

compound A17-C (DMSO-d6, 90°C)

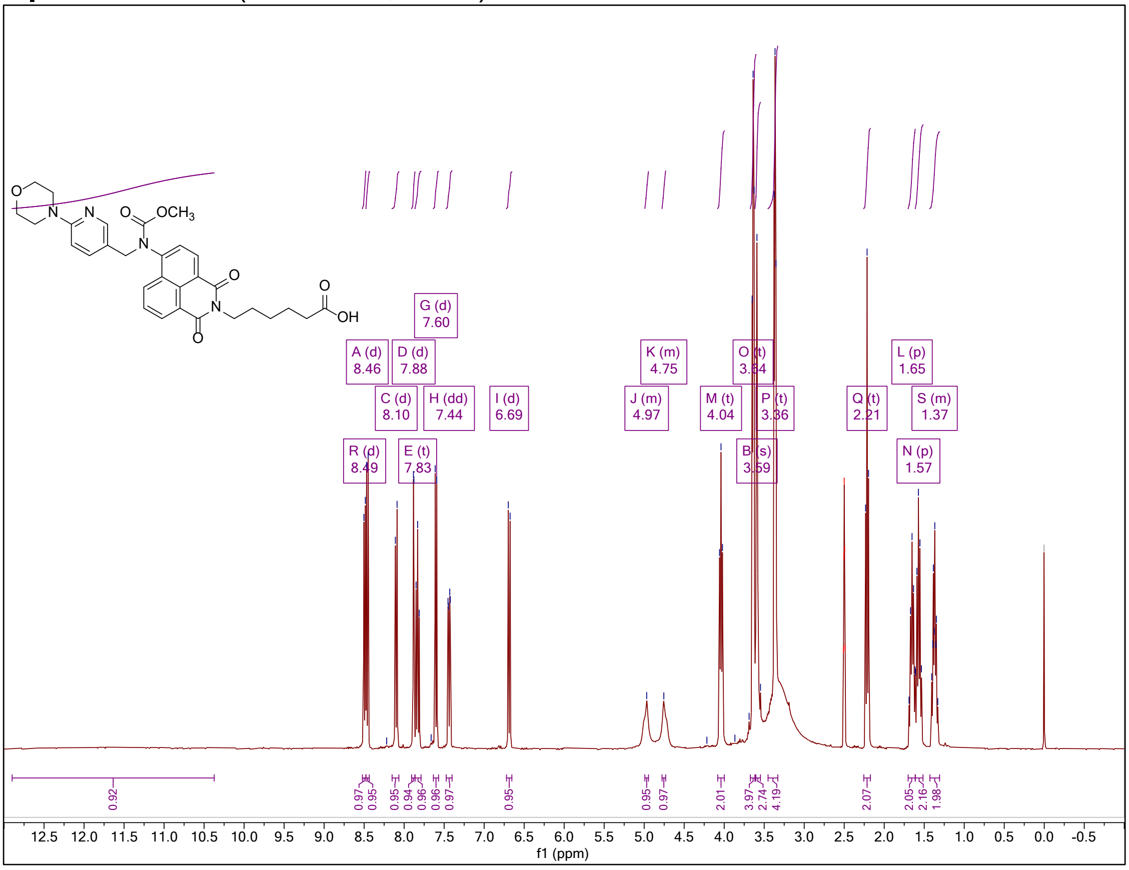

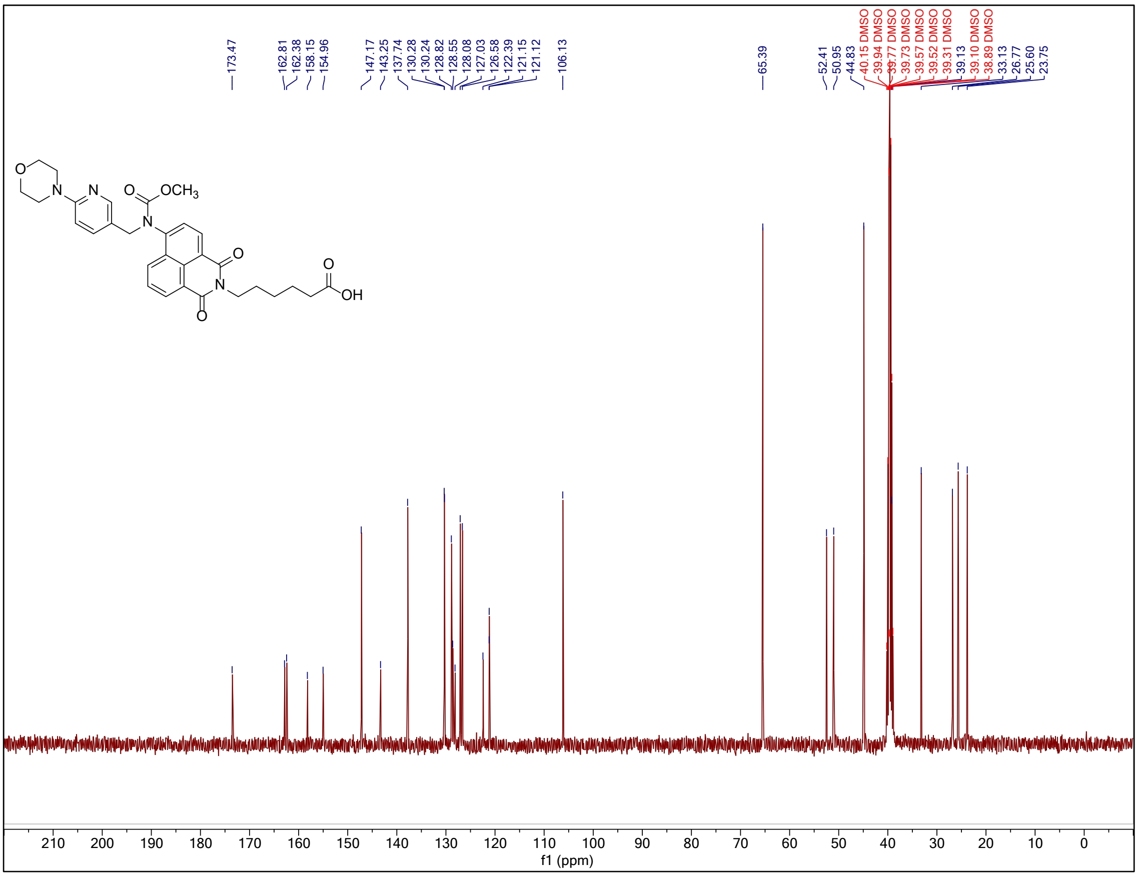

compound A17-C-AA (DMSO-d6, 90°C)

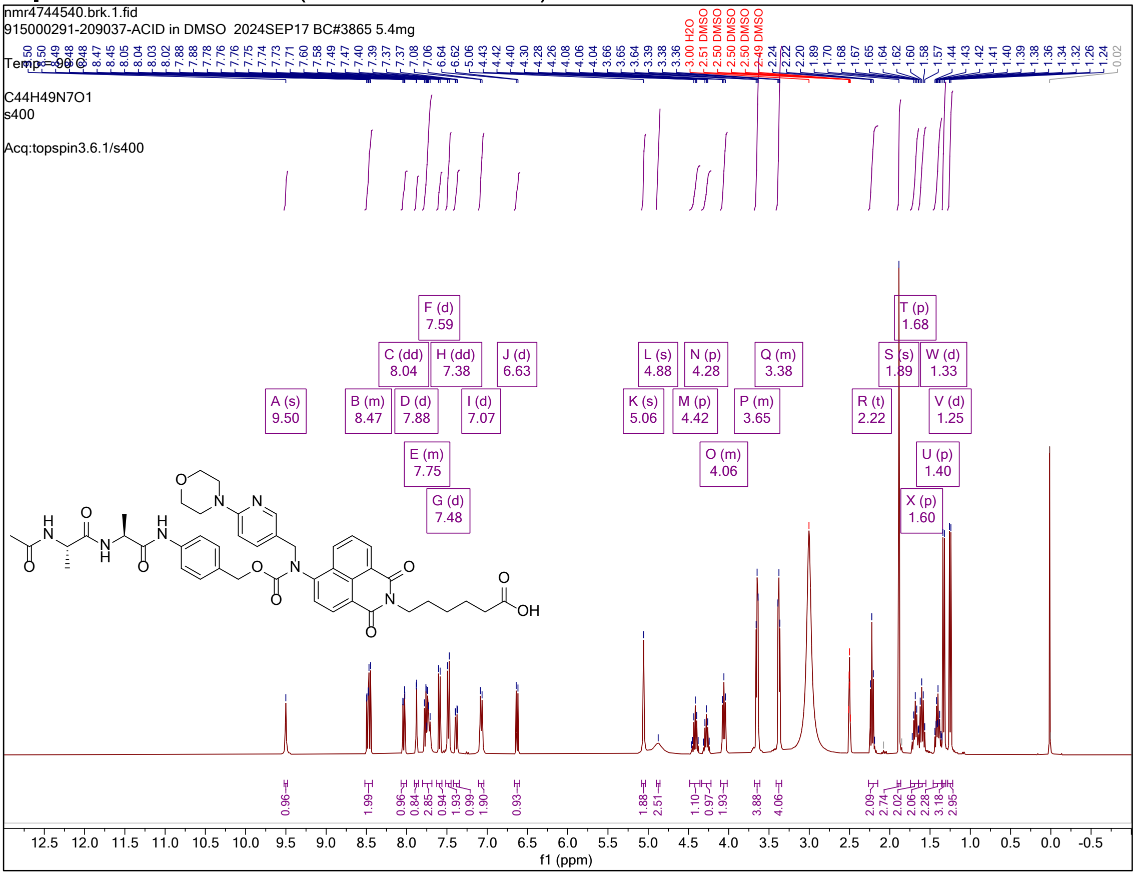

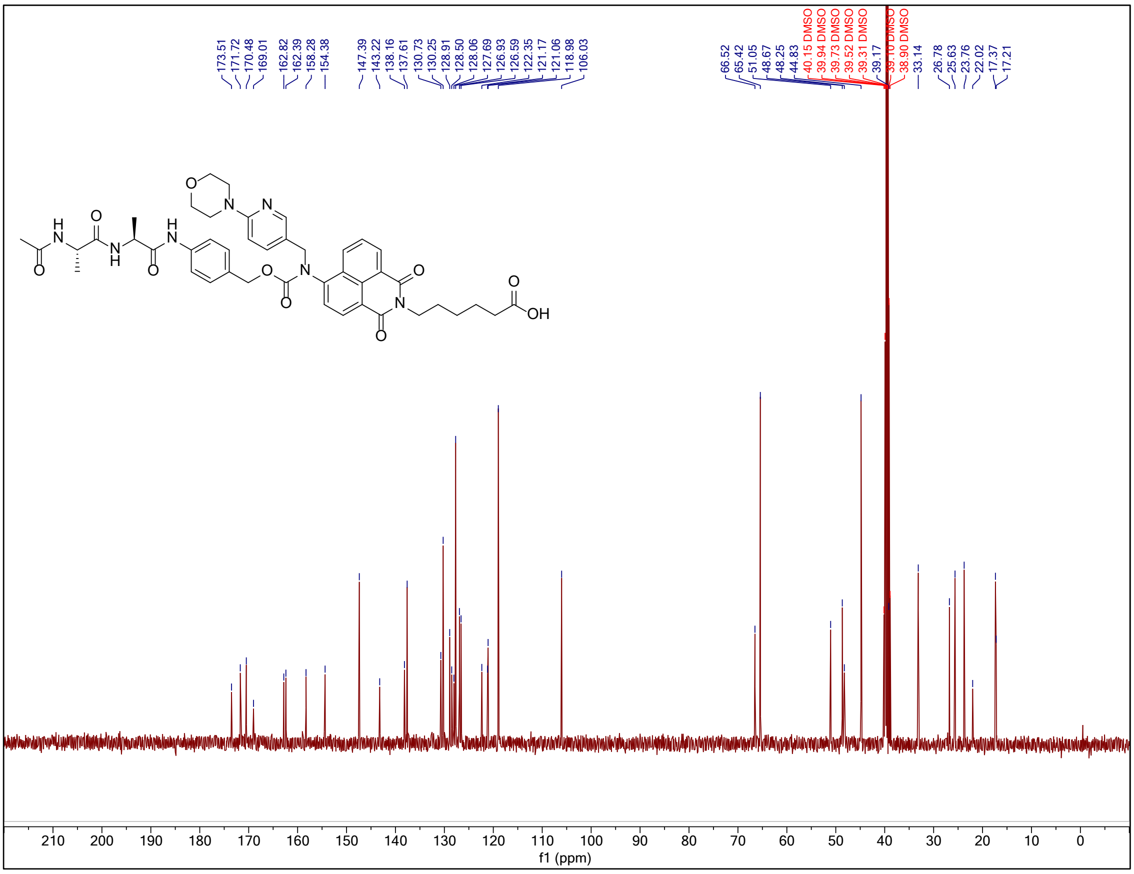

compound A21(tBu) (DMSO-d6)

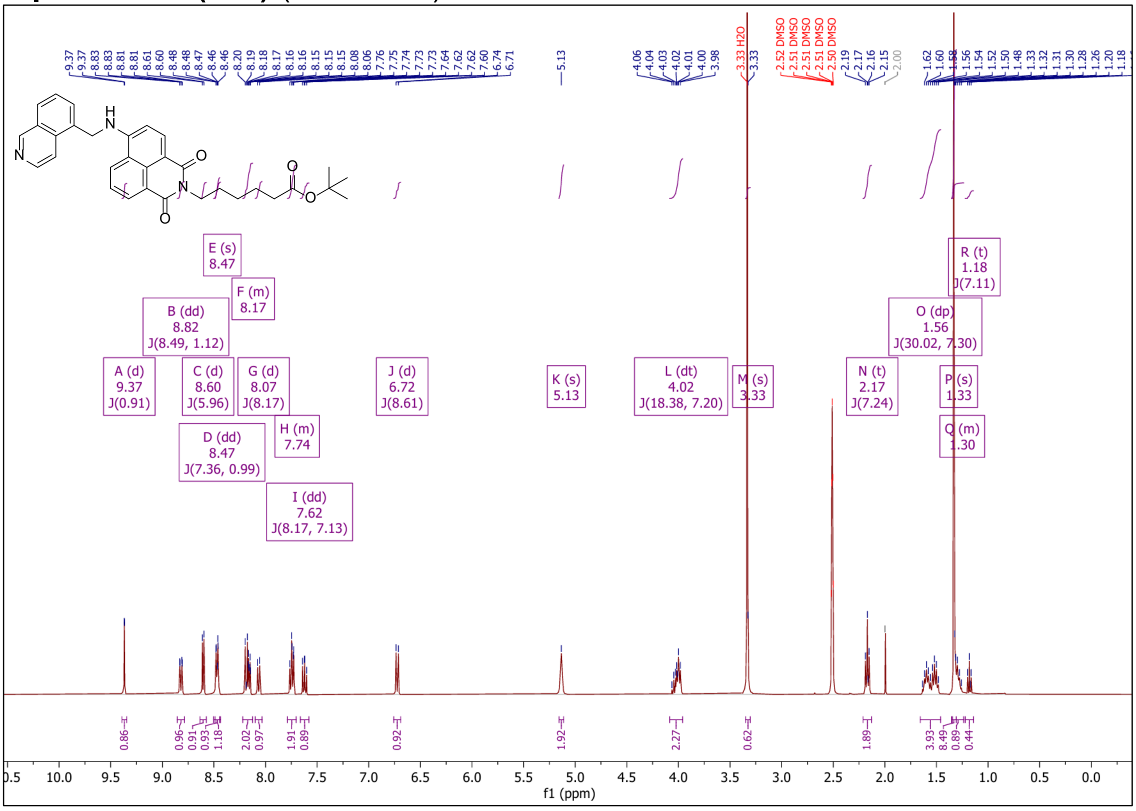

compound A21-C (DMSO-d6, 90°C)

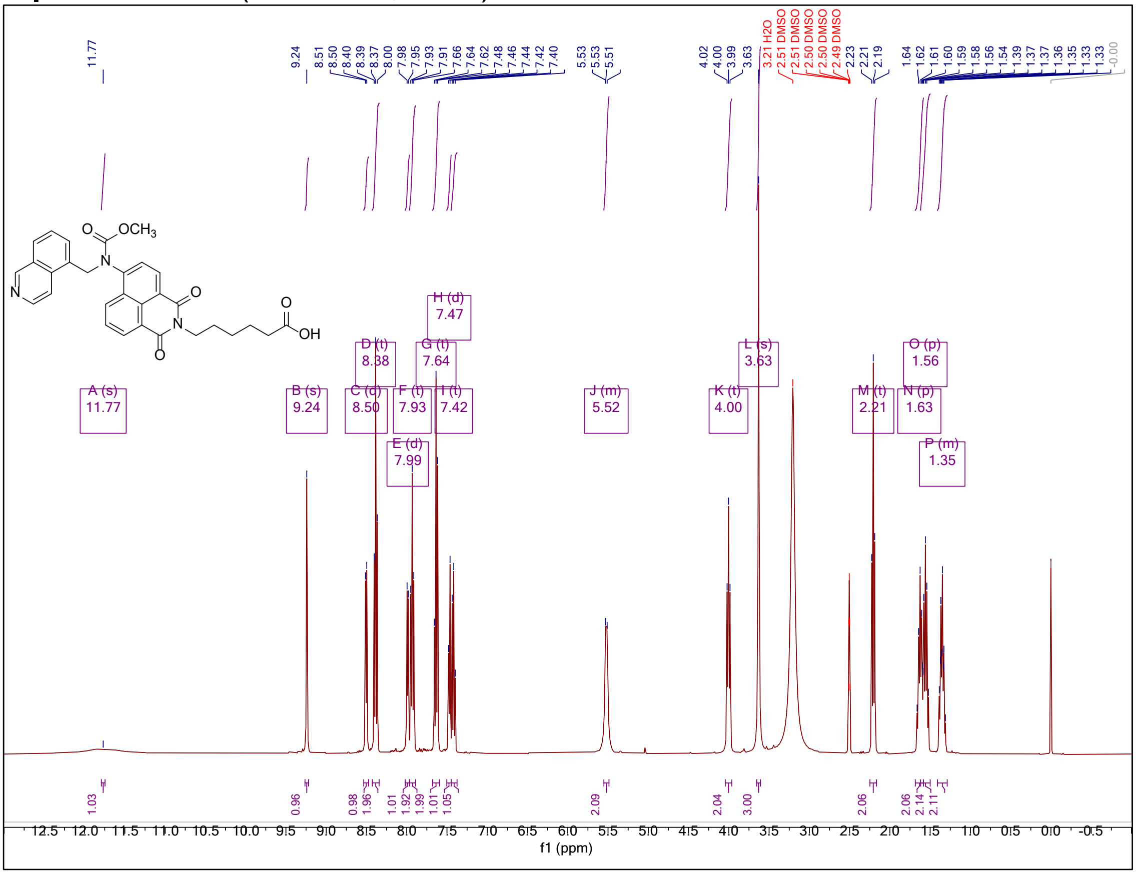

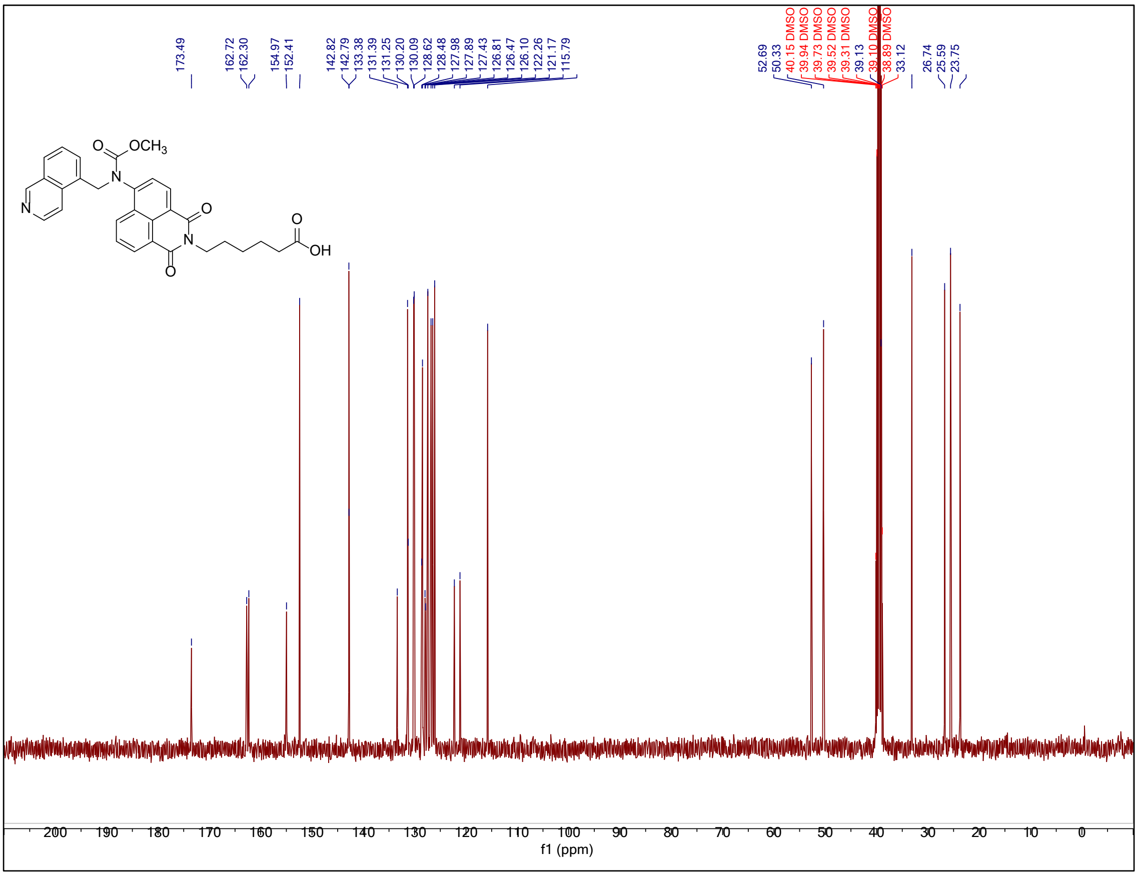

compound 7 (DMSO-d6)

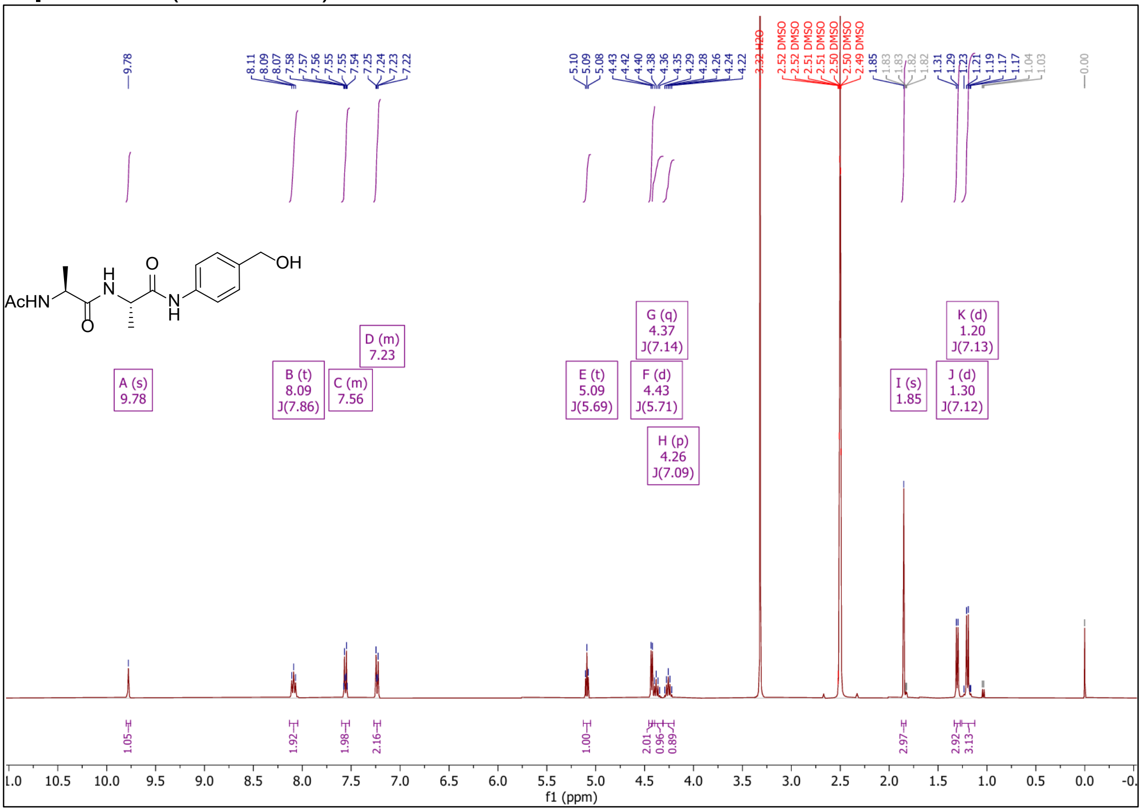

compound 8 (DMSO-d6)

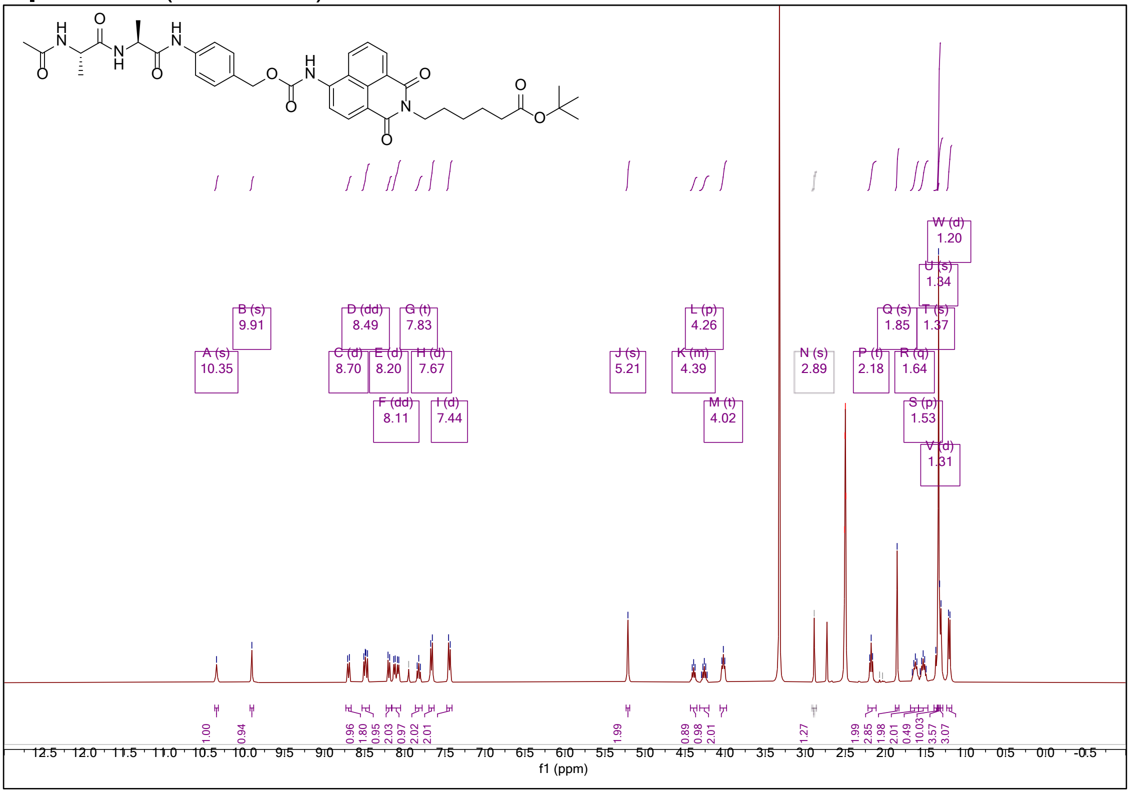

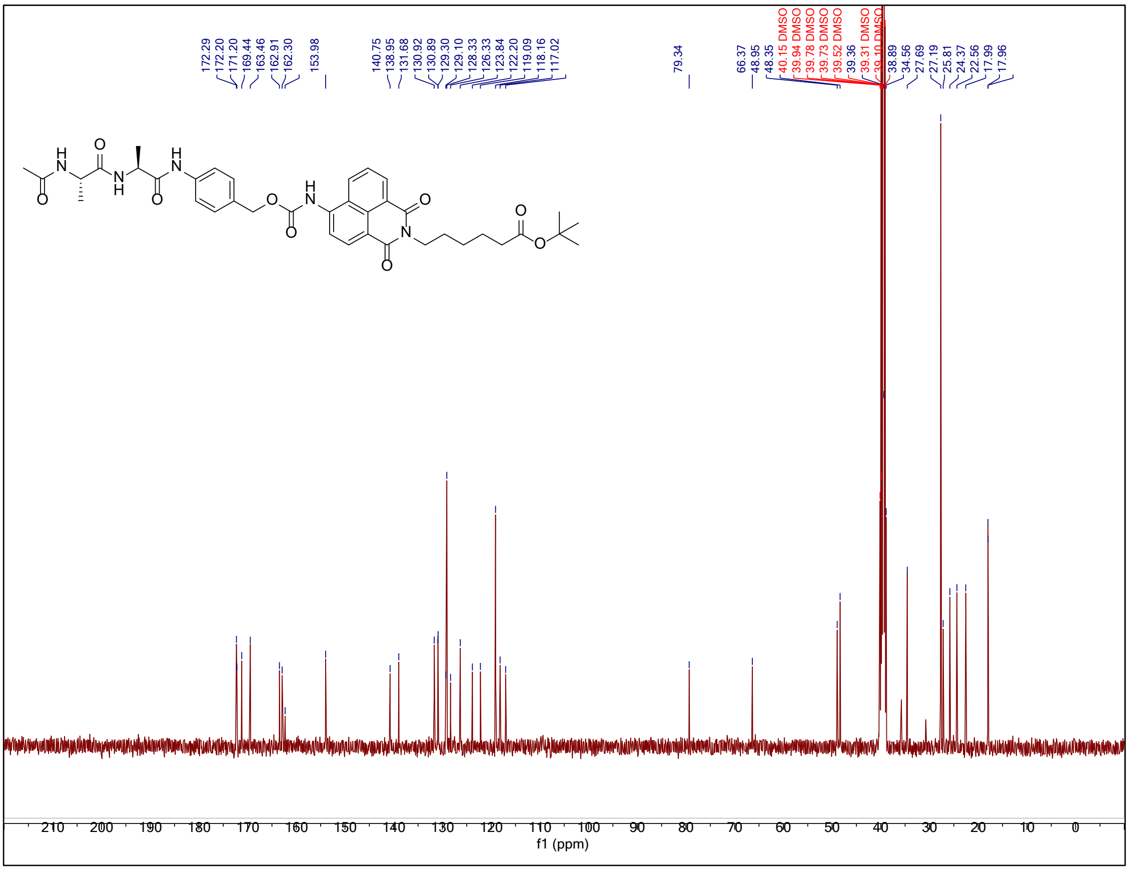

compound 10 (DMSO-d6)

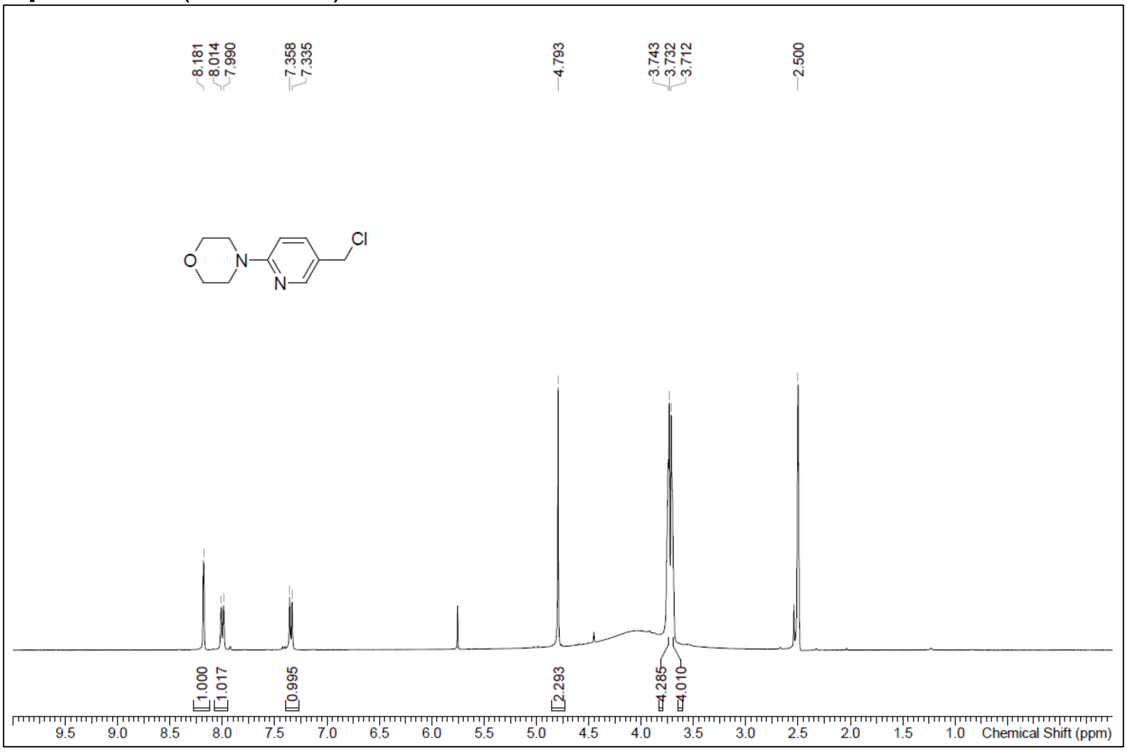

compound A17-C

1H NMR (DMSO-d6, 90°C)

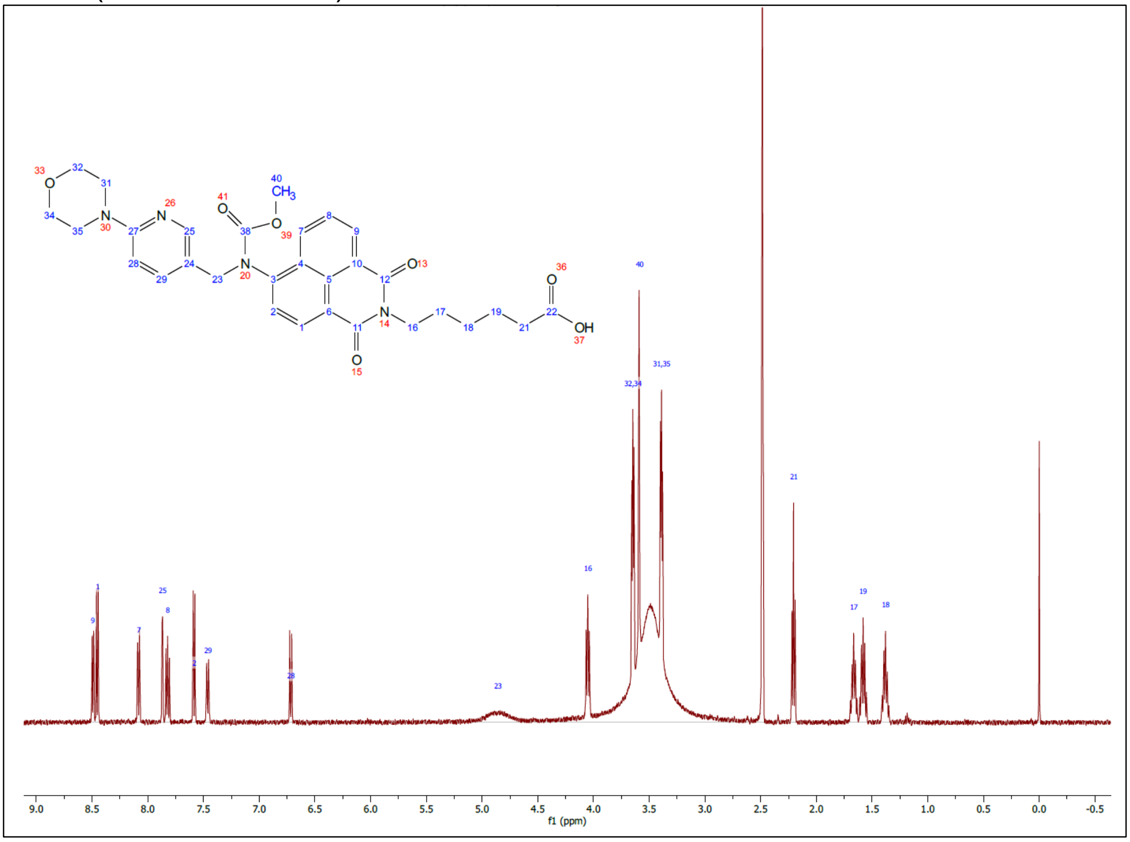

1H COSY (DMSO-d6, 90°C)

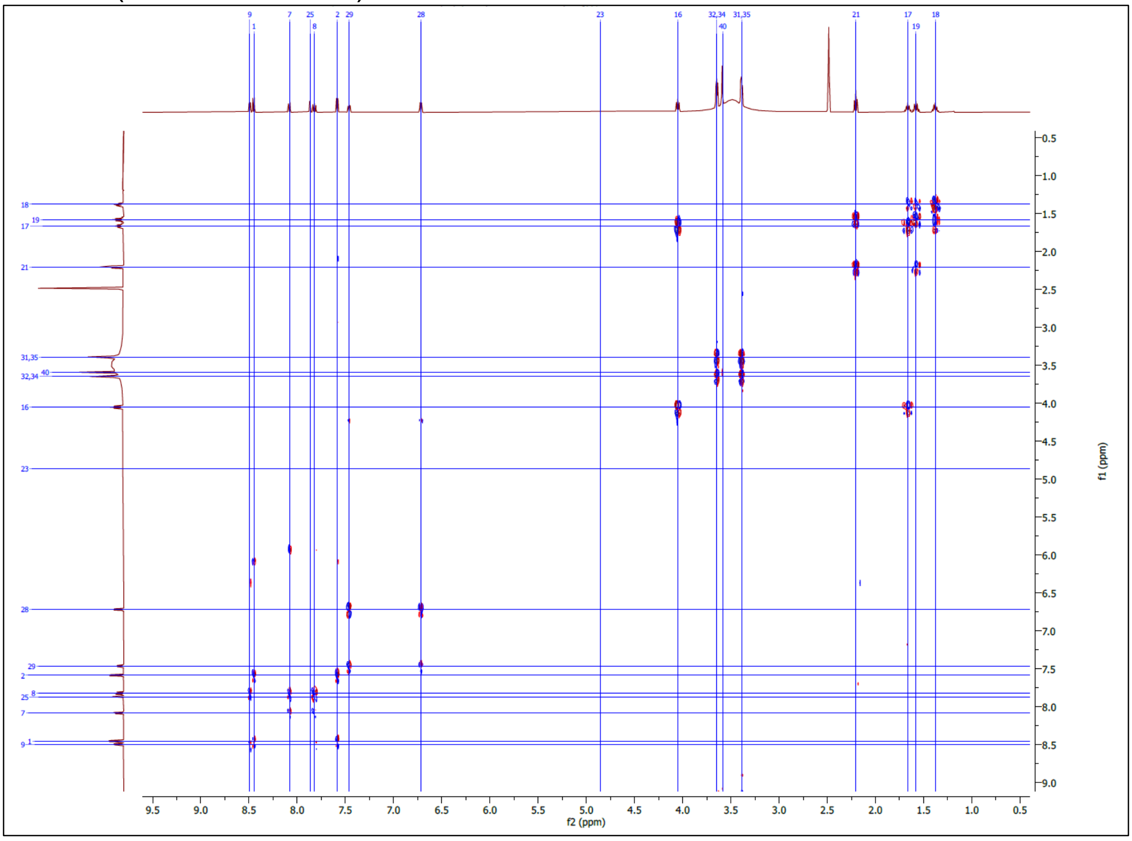

1H ROESY (DMSO-d6, 90°C)

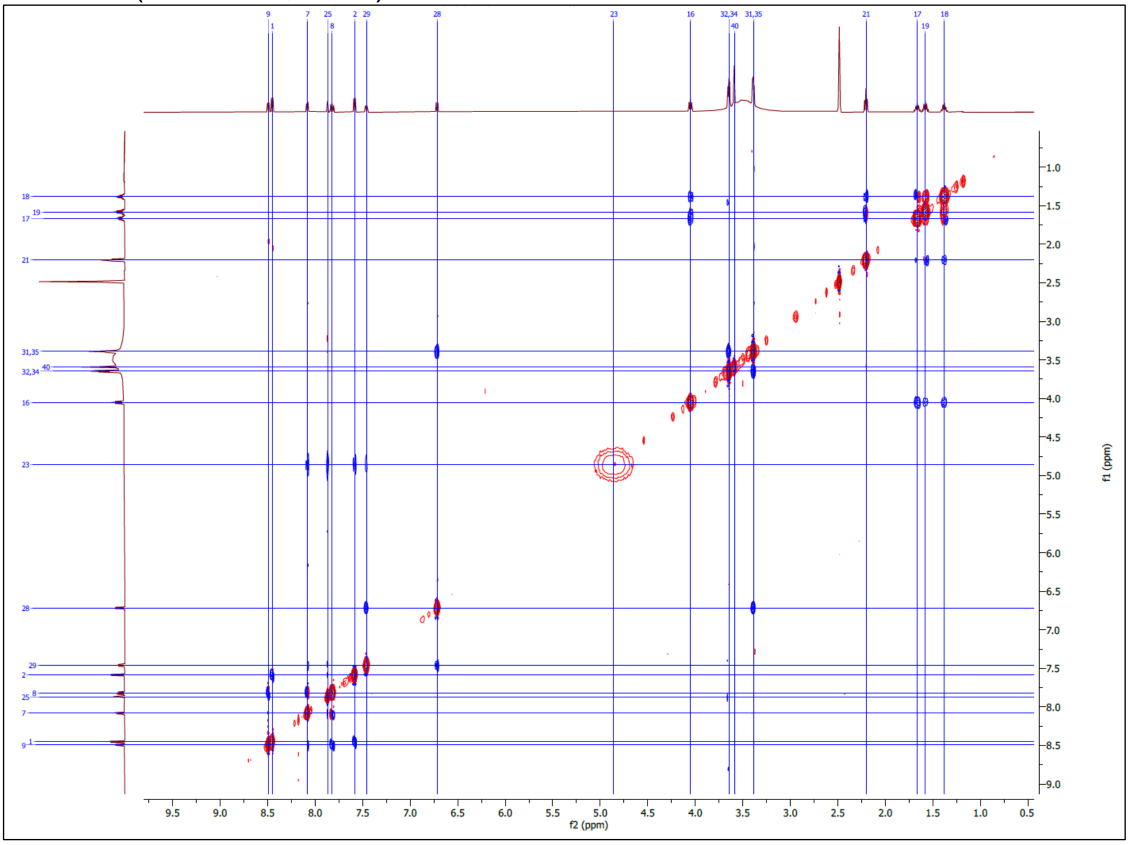

HSQC (DMSO-d6, 90°C)

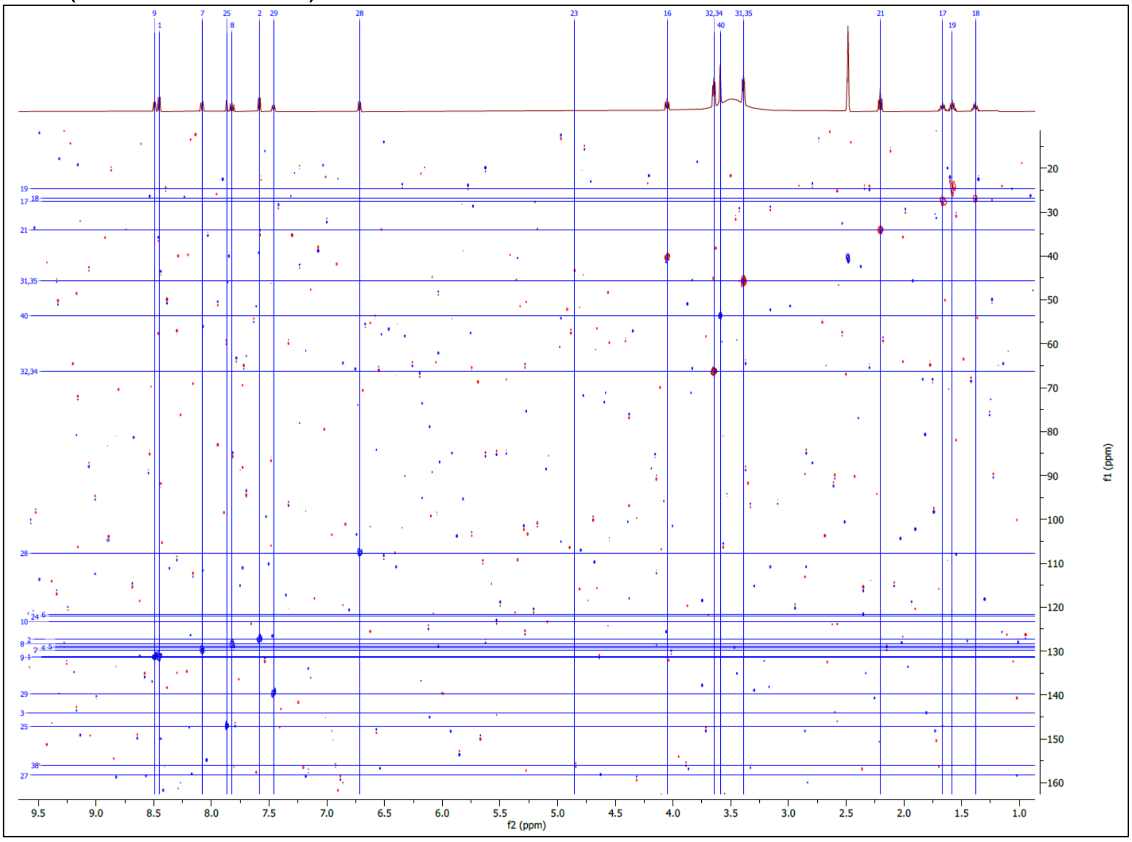

HMBC (DMSO-d6, 90°C)

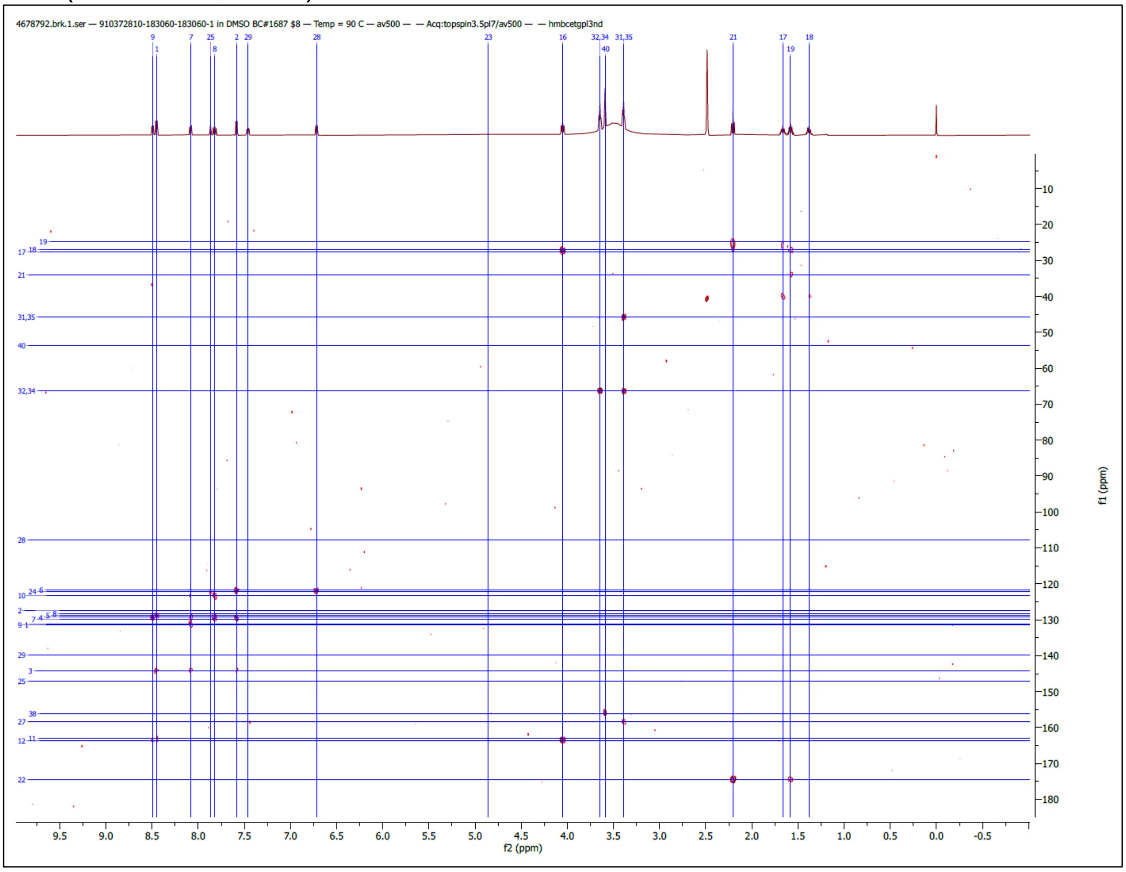

compound A21

1H NMR (DMSO-d6)

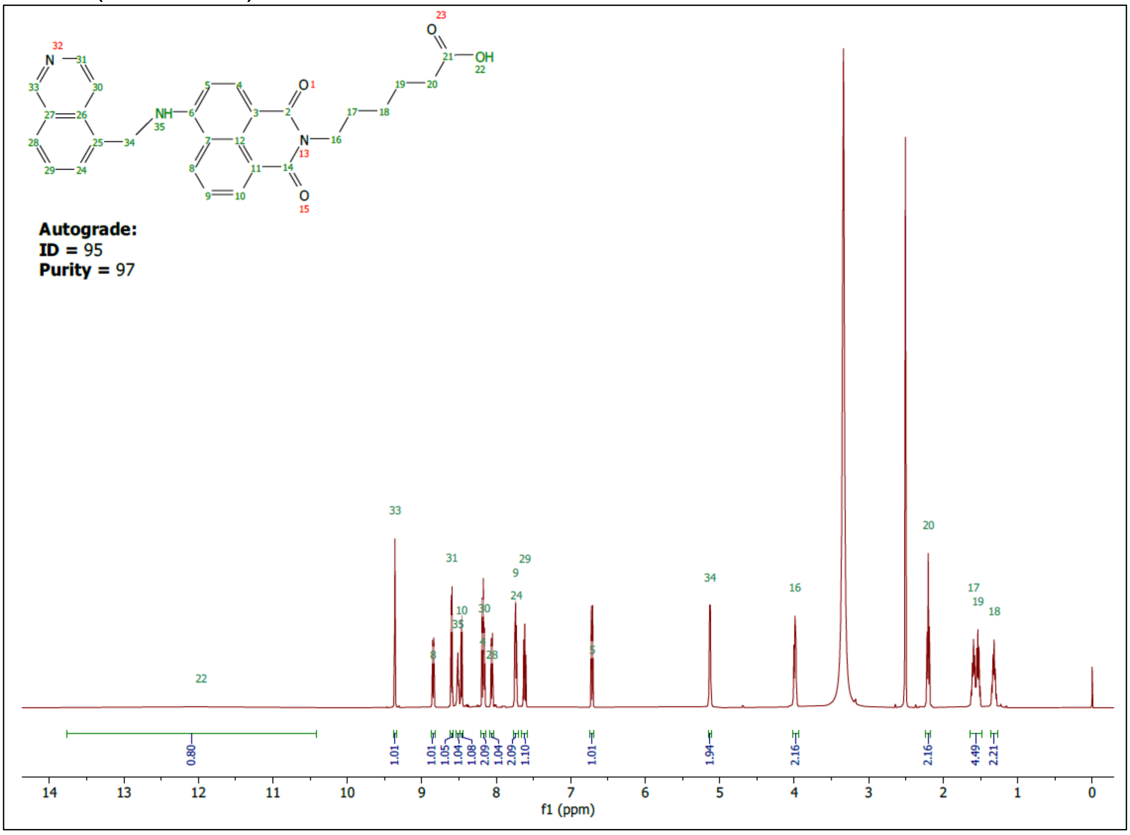

1H COSY (DMSO-d6)

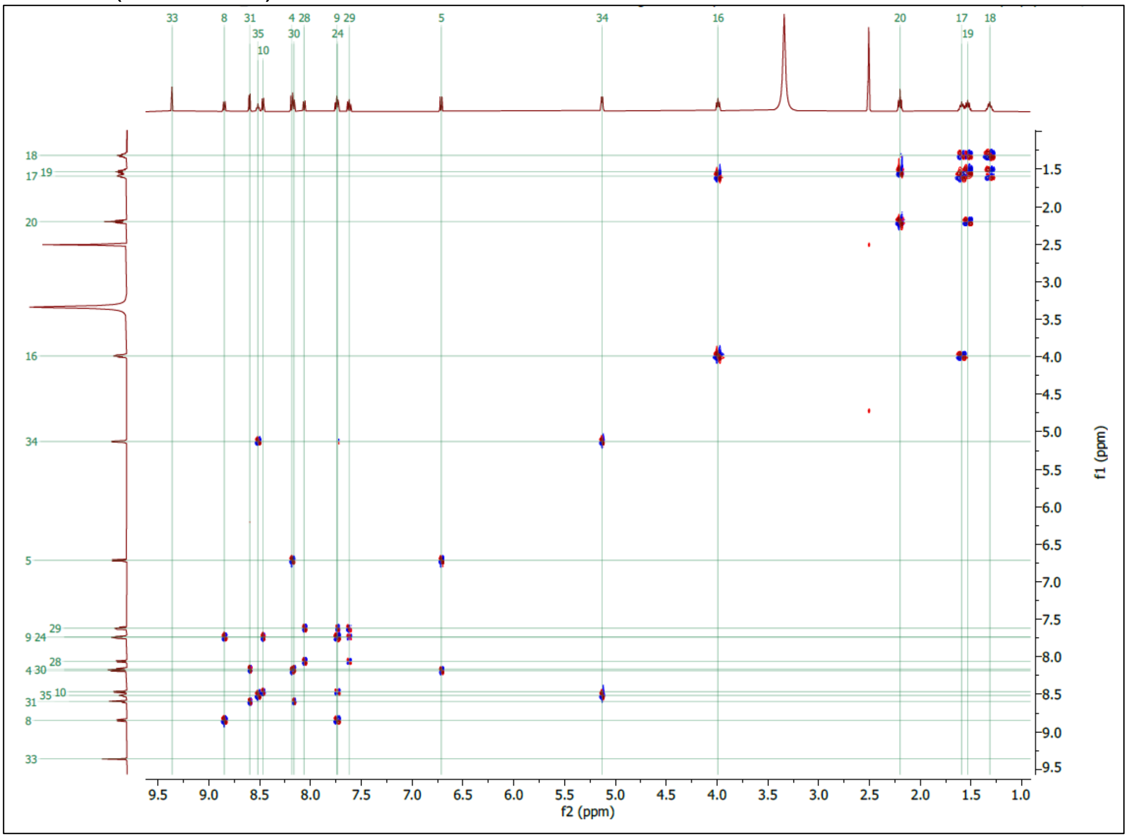

1H ROESY (DMSO-d6)

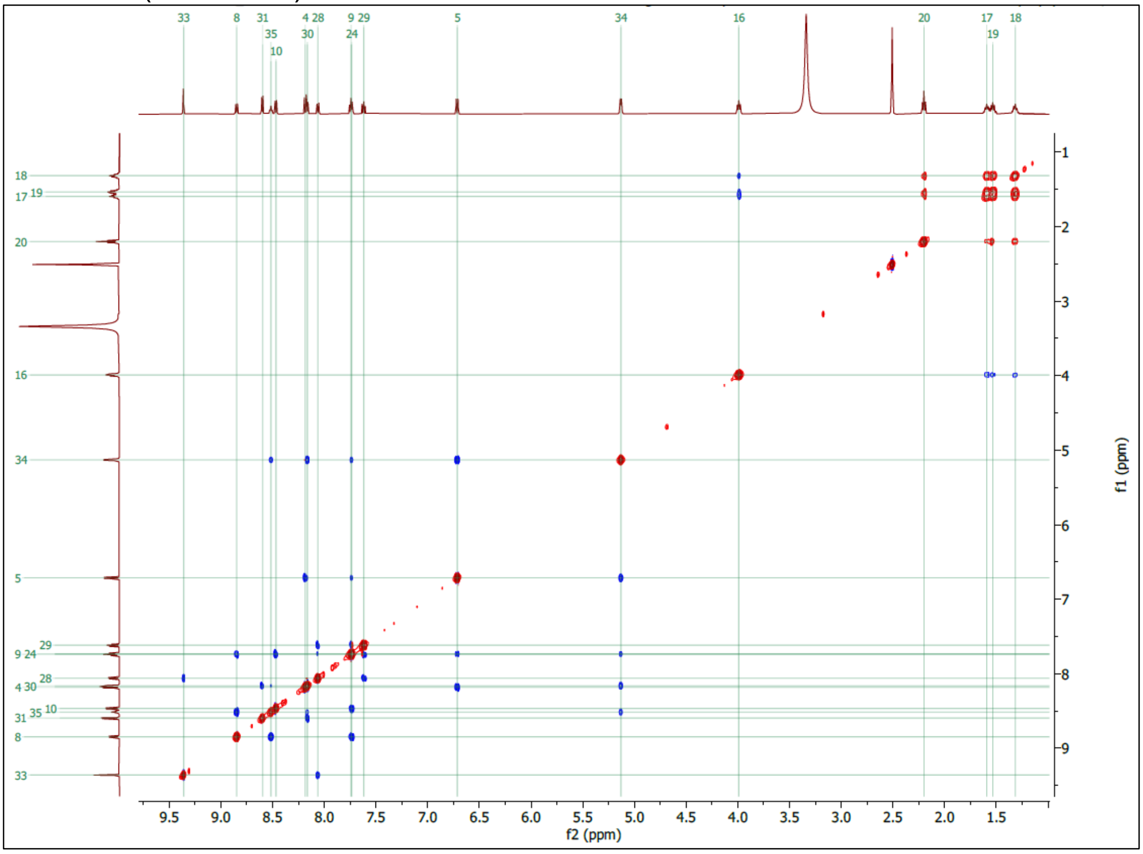

HSQC (DMSO-d6)

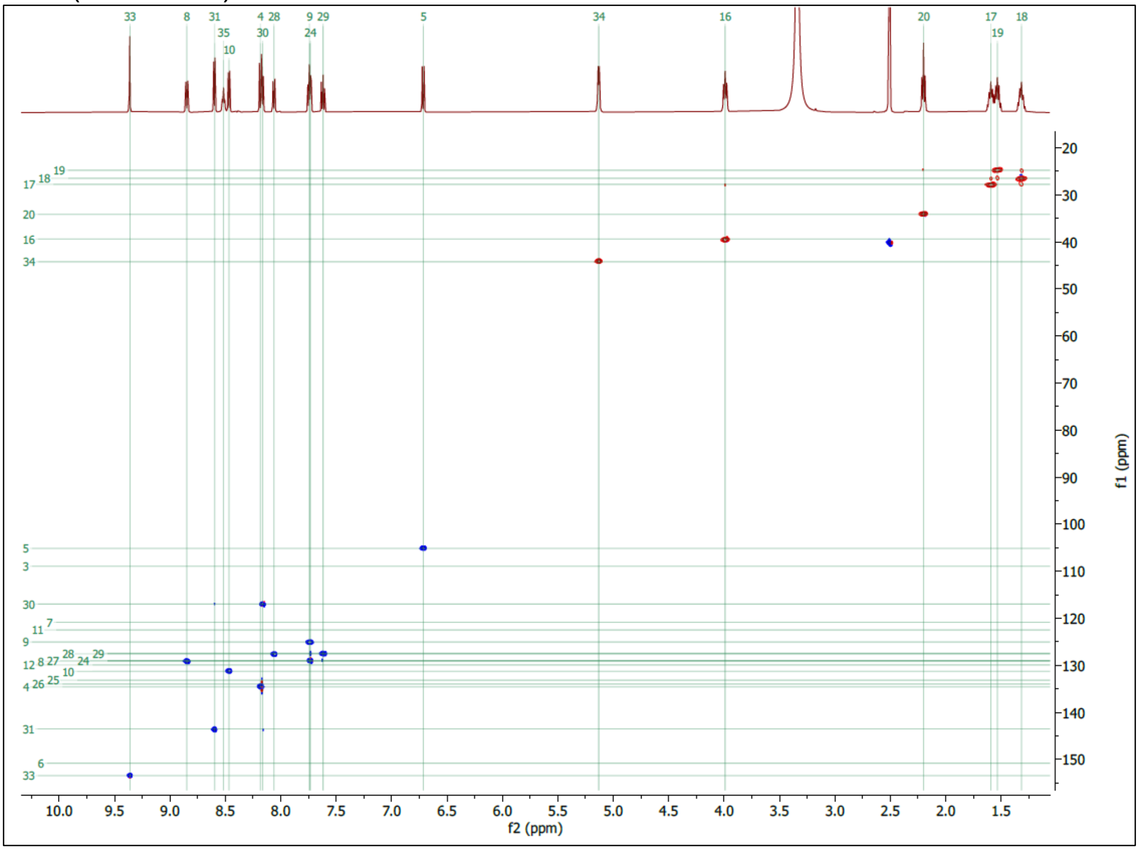

HMBC (DMSO-d6)

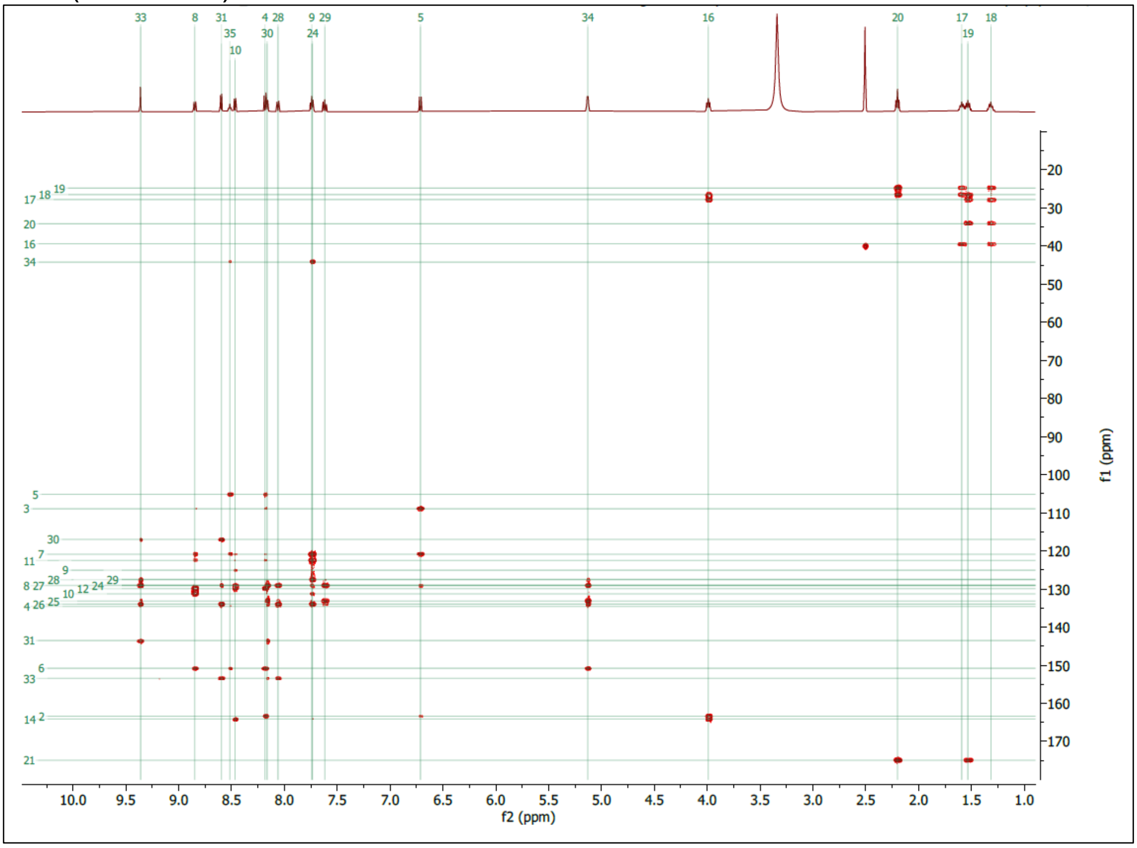

compound A17-C-AA

1H NMR (pyridine-d5, -20°C)

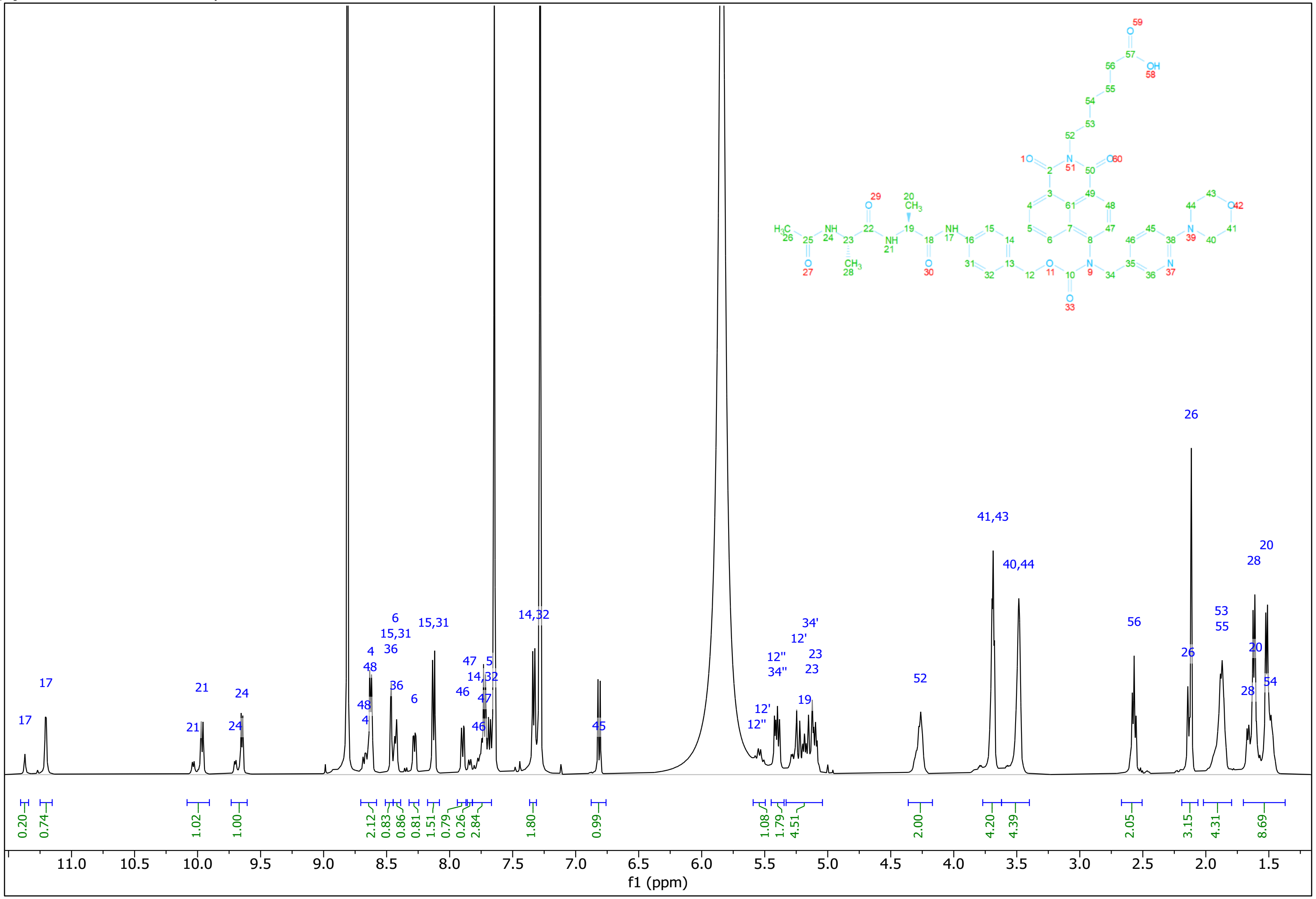

1H,1H, DQF-COSY (pyridine-d5, -20°C)

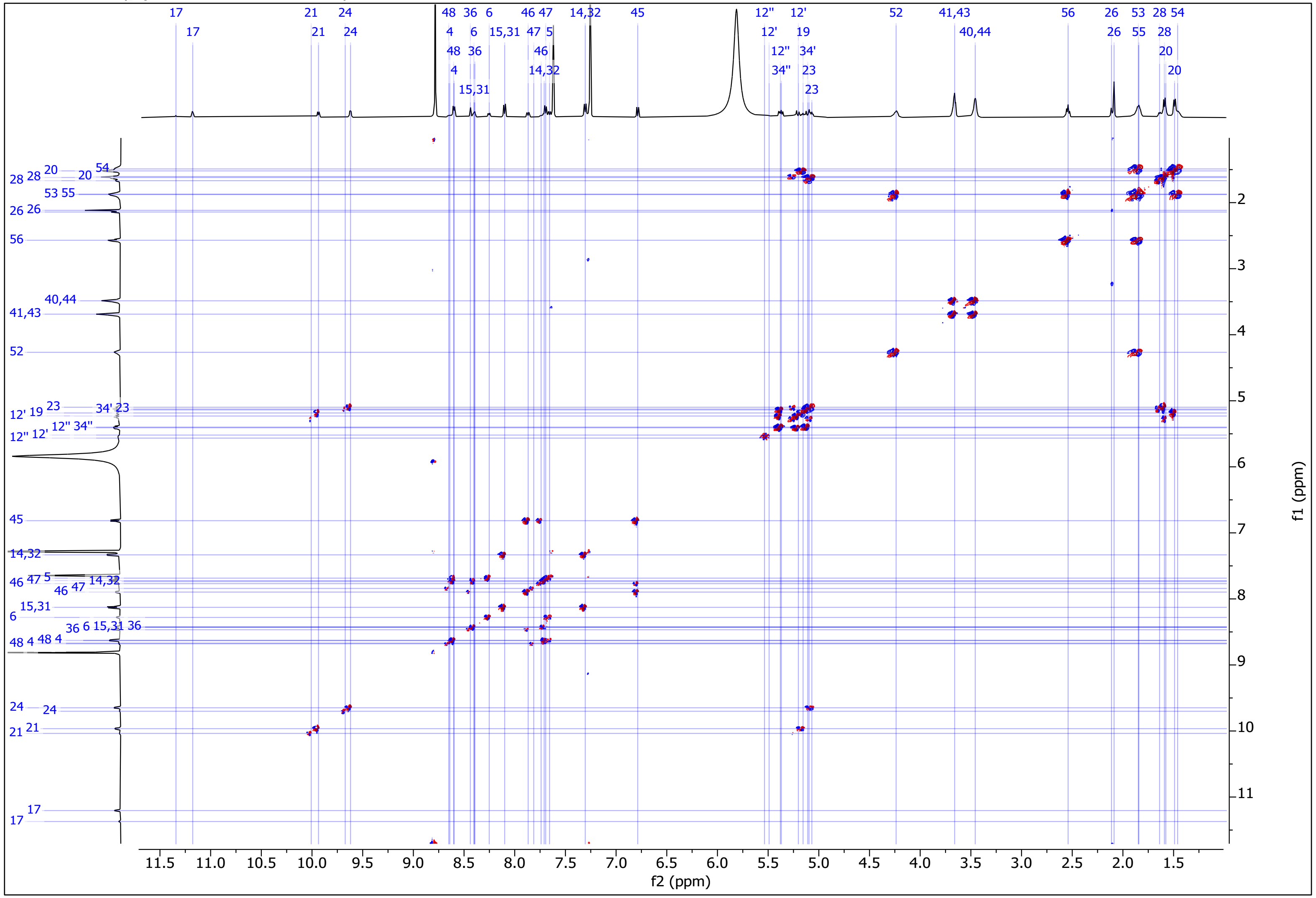

1H,1H ROESY (pyridine-d5, -20°C)

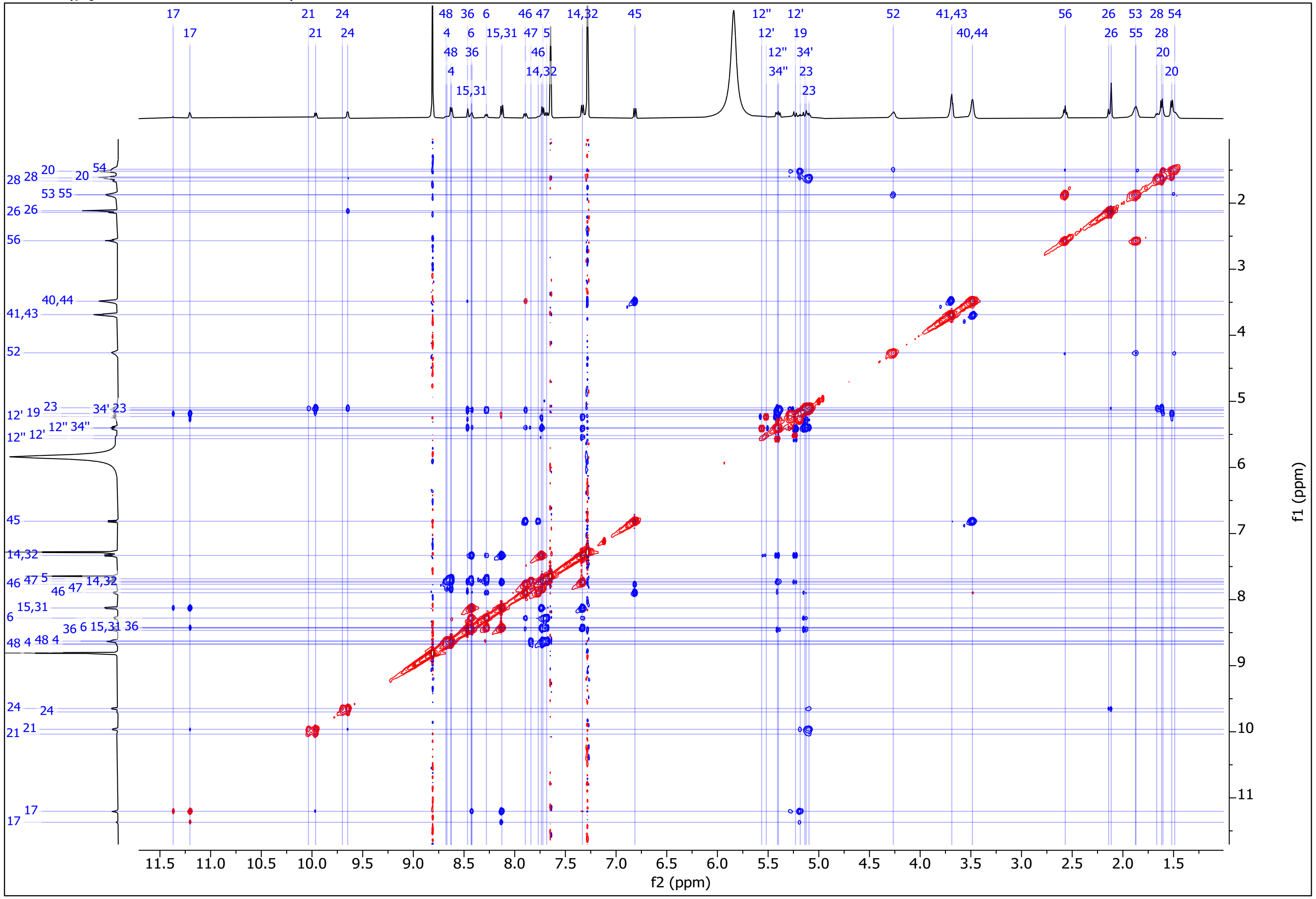

1H,13C HSQC (pyridine-d5, -20°C)

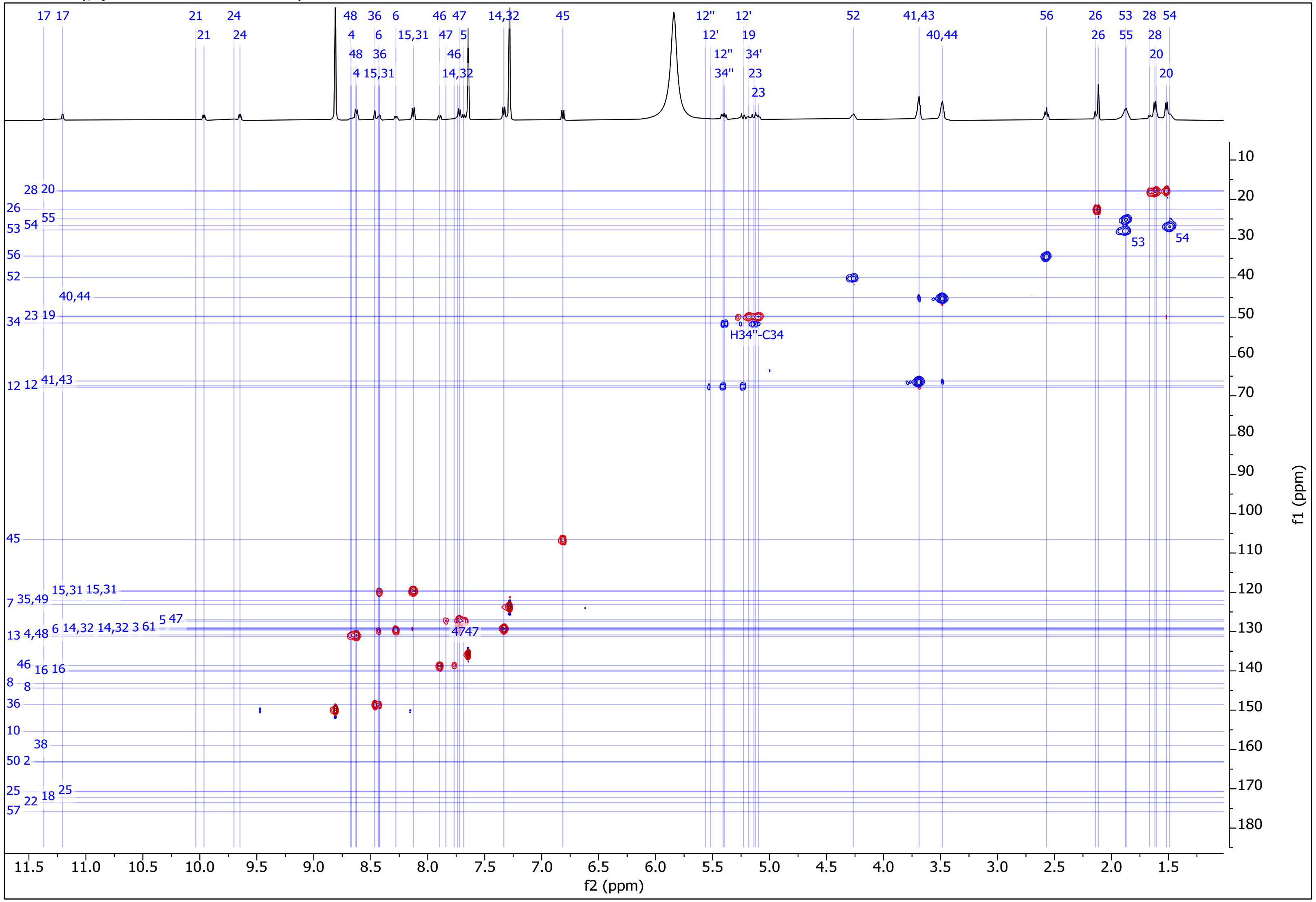

1H,13C HMBC (pyridine-d5, -20°C)

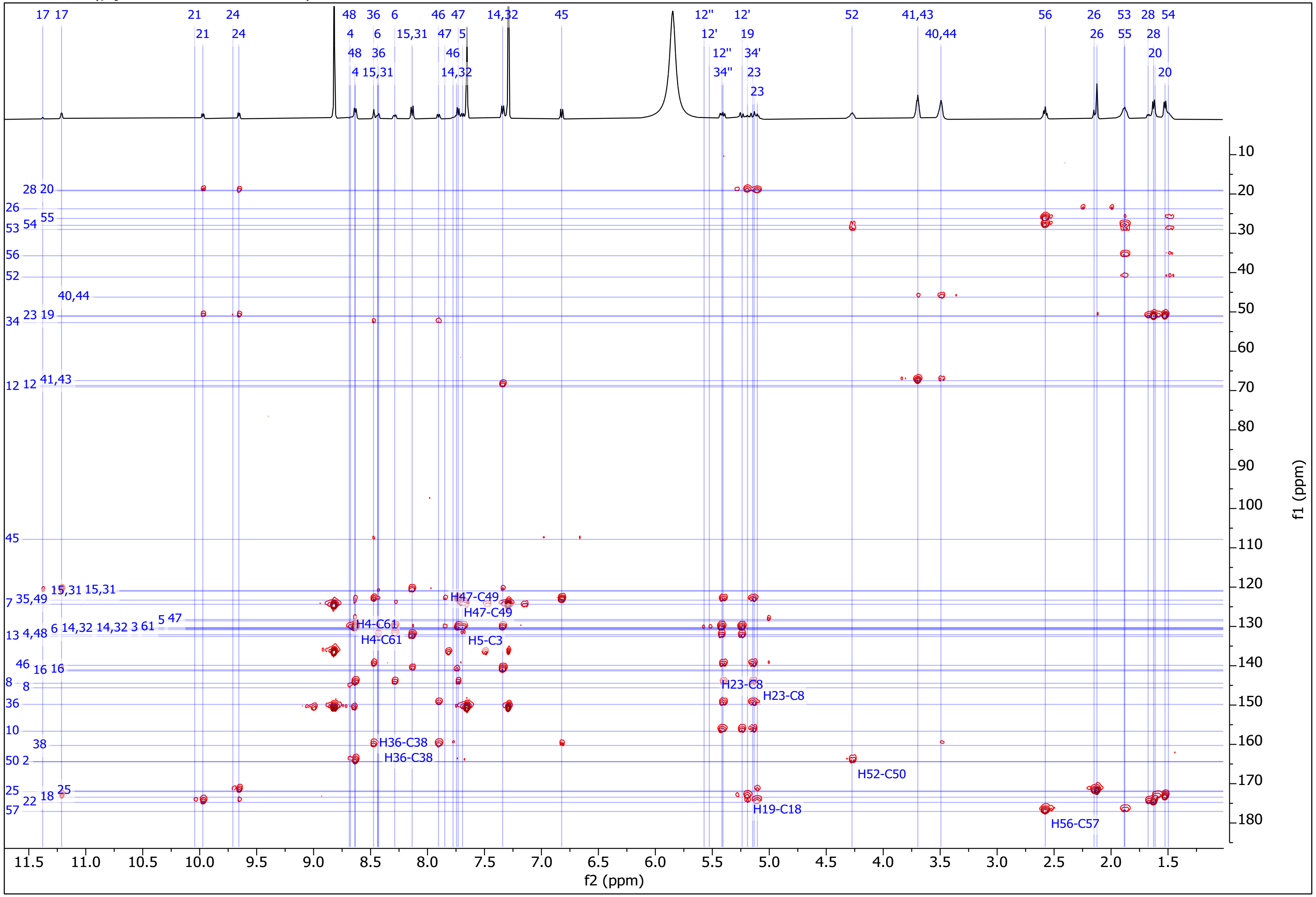

5. High-Resolution Mass Spectra

Compound A1

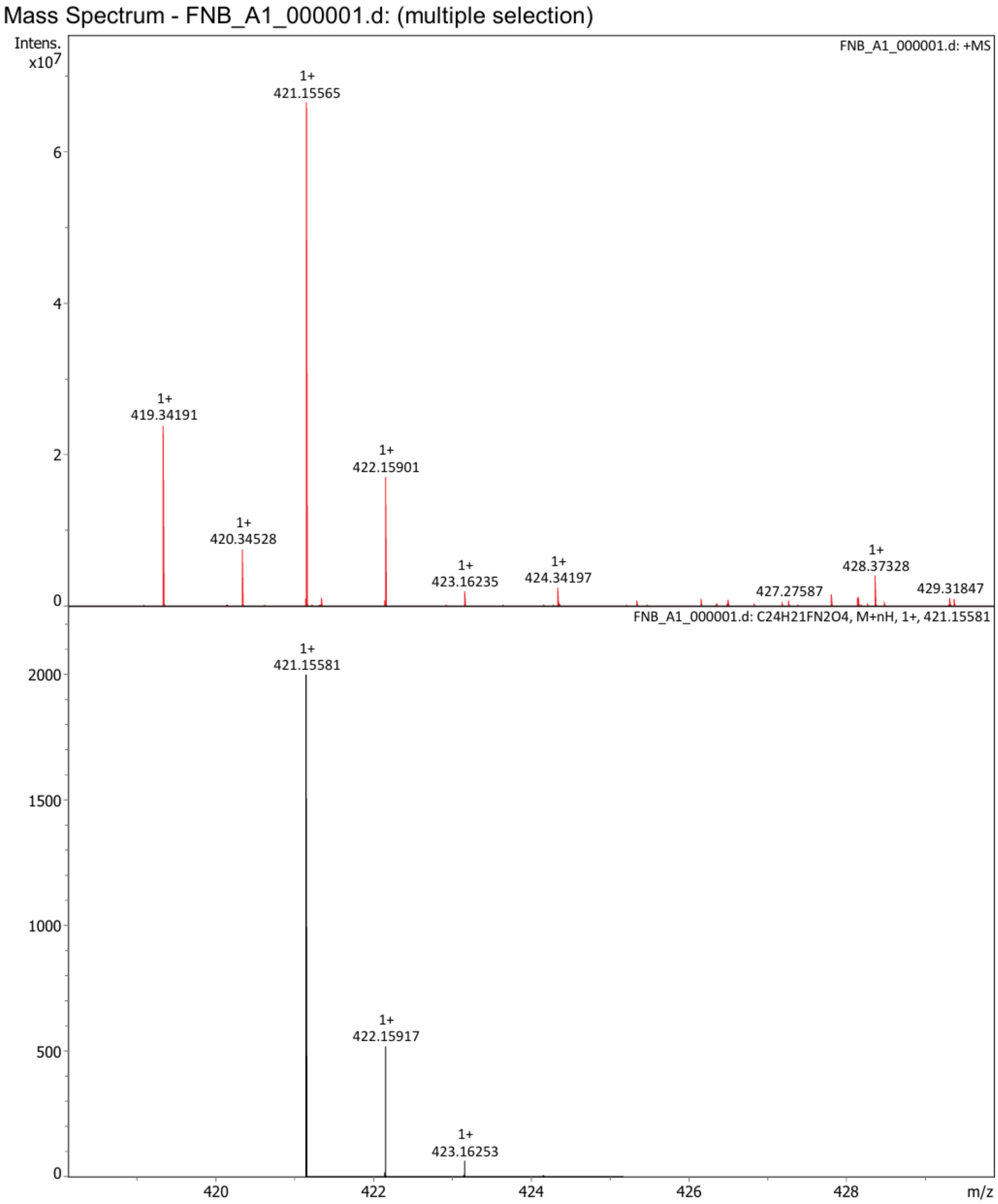

Compound A2

Mass Spectrum - FNB_A2_5ms_000001.d: C32H33N5O4, M+nH, 1+, 552.26053

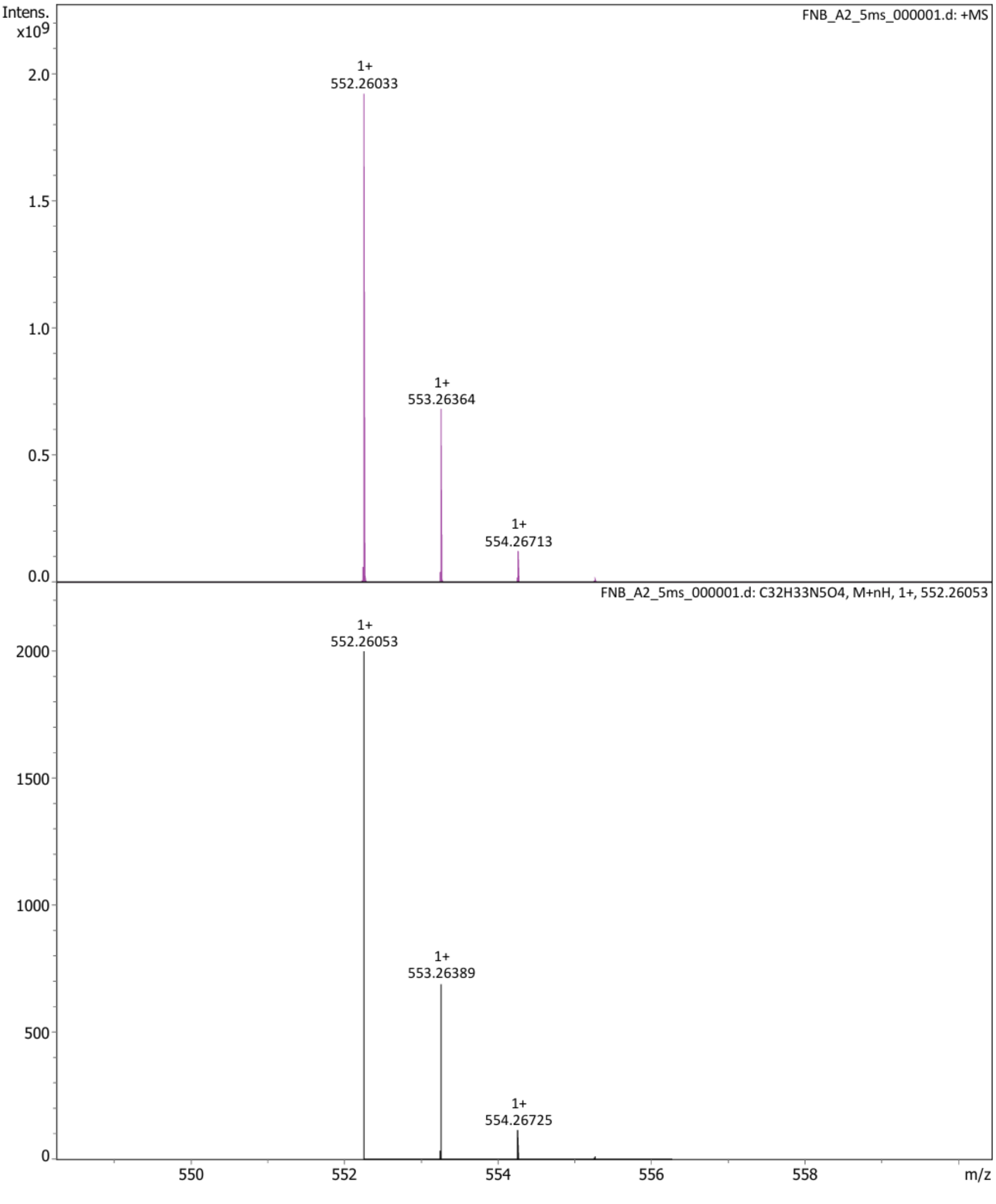

Compound A3

Mass Spectrum - FNB_A3_000001.d: (multiple selection)

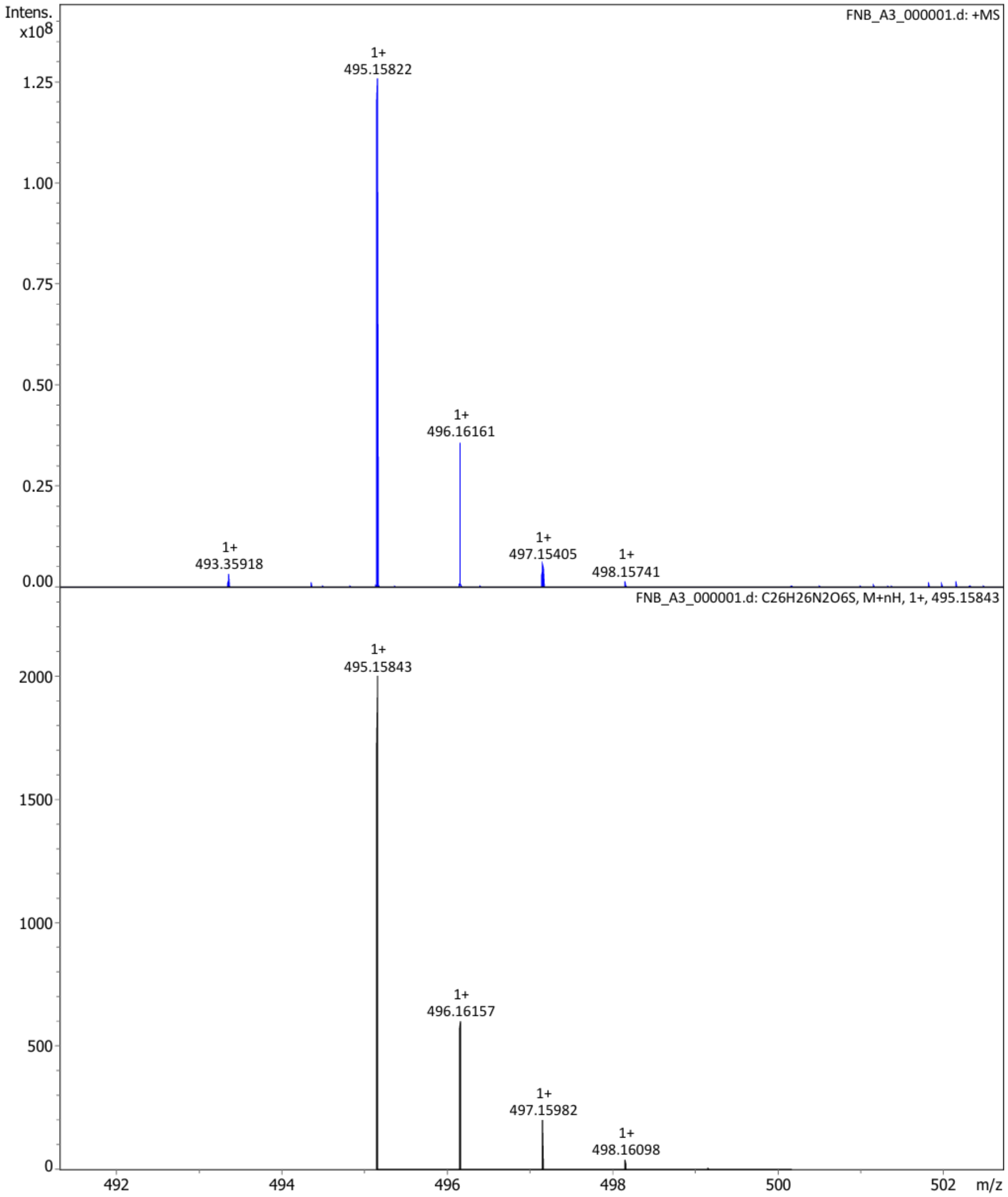

Compound A4

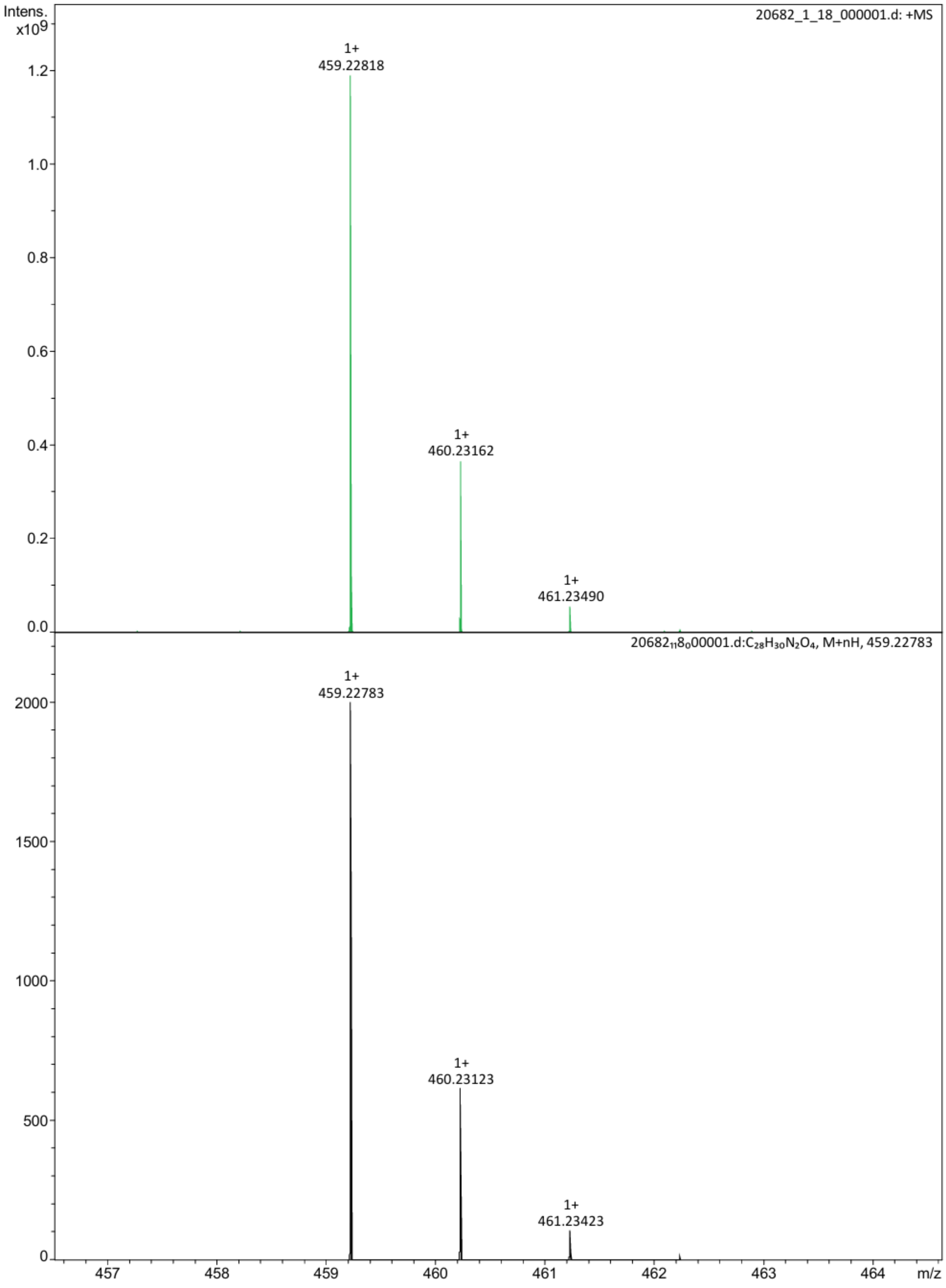

Intens.
x10^9
20682_1_18_000001.d: +MS
1+
459.22818
1+
460.23162
1+
461.23490
20682₁₁8₀00001.d:C₂₈H₃₀N₂O₄, M+nH, 459.22783
1+
459.22783
1+
460.23123
1+
461.23423
m/z

Compound A5

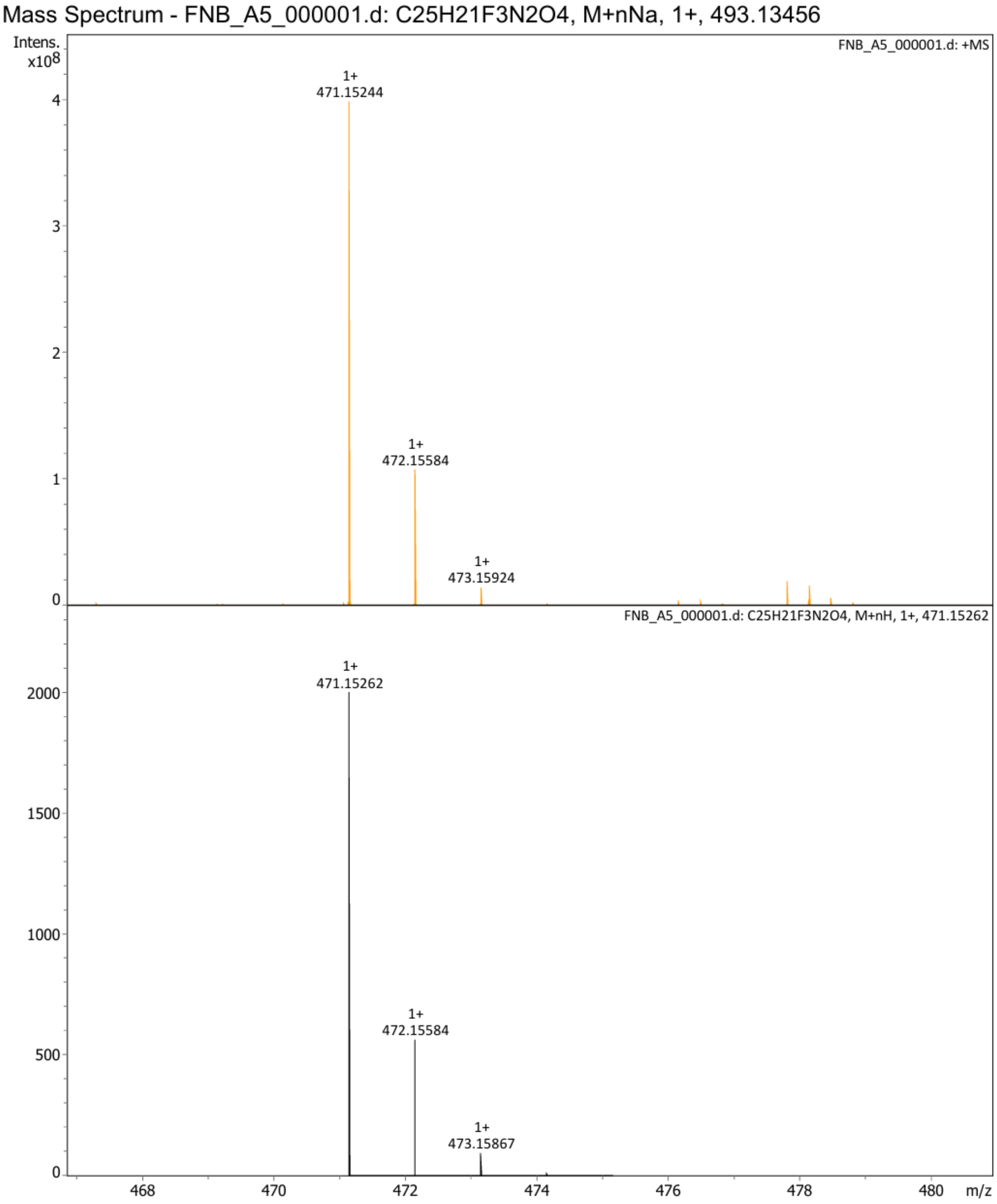
Mass Spectrum - FNB_A5_000001.d: C25H21F3N2O4, M+nNa, 1+, 493.13456
Intens.
x10^8
FNB_A5_000001.d: +MS
1+
471.15244
1+
472.15584
1+
473.15924
FNB_A5_000001.d: C25H21F3N2O4, M+nH, 1+, 471.15262
1+
471.15262
1+
472.15584
1+
473.15867
m/z

Compound A6

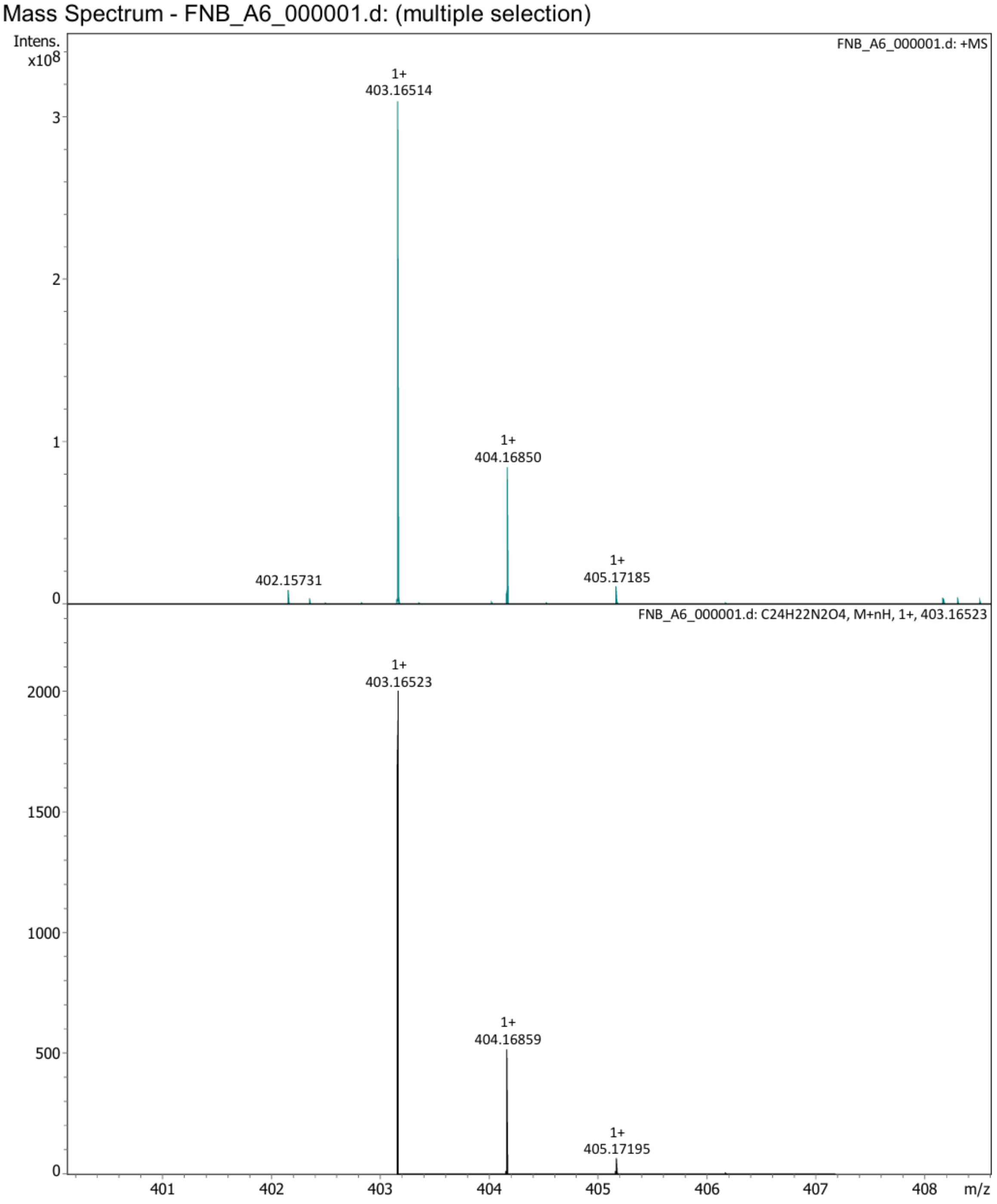

Mass Spectrum - FNB_A6_000001.d: (multiple selection)
Intens.
x10^8
FNB_A6_000001.d: +MS
1+
403.16514
1+
404.16850
1+
405.17185
402.15731
FNB_A6_000001.d: C24H22N2O4, M+nH, 1+, 403.16523
1+
403.16523
1+
404.16859
1+
405.17195
m/z

Compound A7

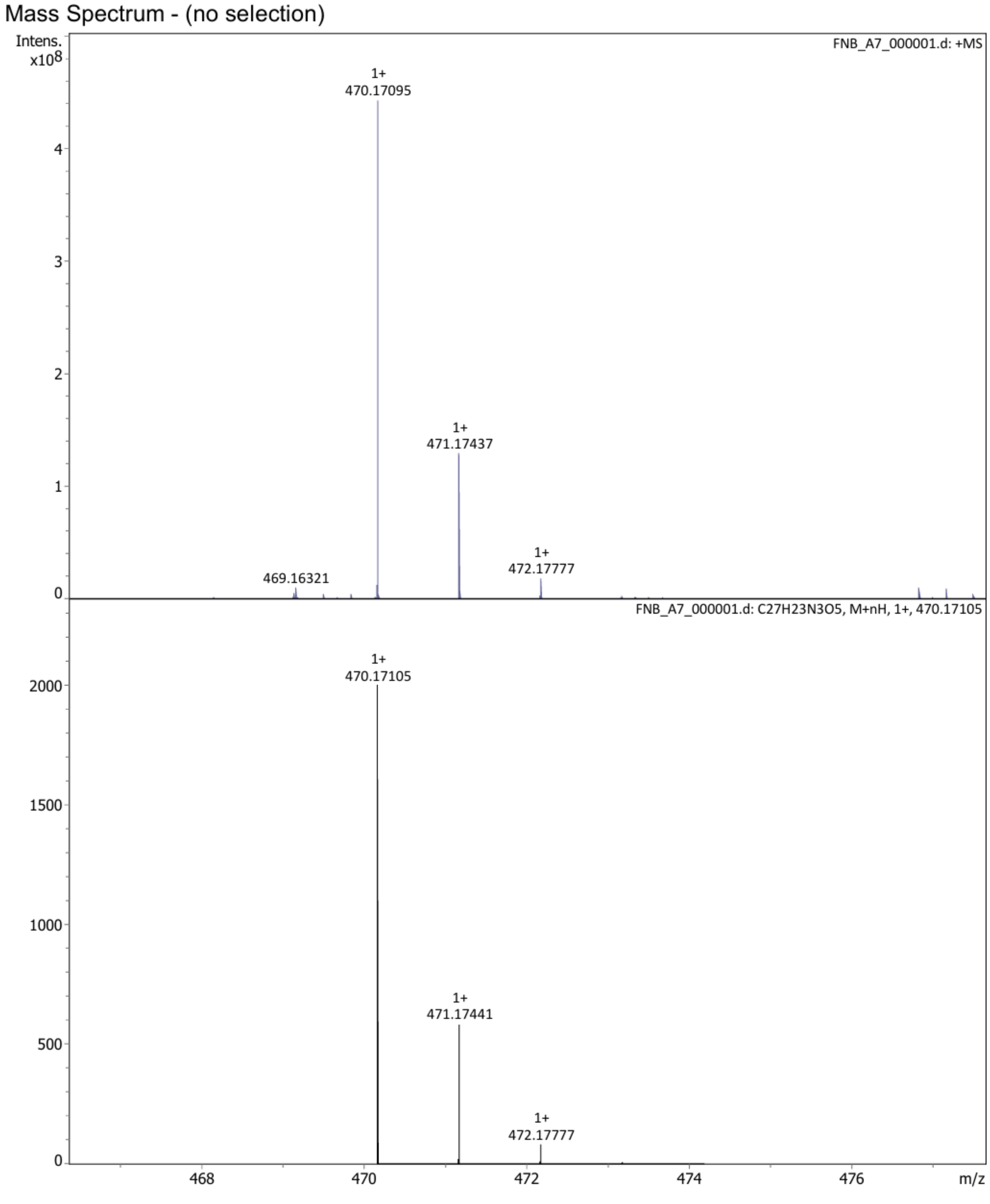

Mass Spectrum - (no selection)
Intens.
x10^8
FNB_A7_000001.d: +MS
1+
470.17095
1+
471.17437
1+
472.17777
469.16321
FNB_A7_000001.d: C27H23N3O5, M+nH, 1+, 470.17105
1+
470.17105
1+
471.17441
1+
472.17777
4
3
2
1
0
2000
1500
1000
500
0
468
470
472
474
476
m/z

Compound A8

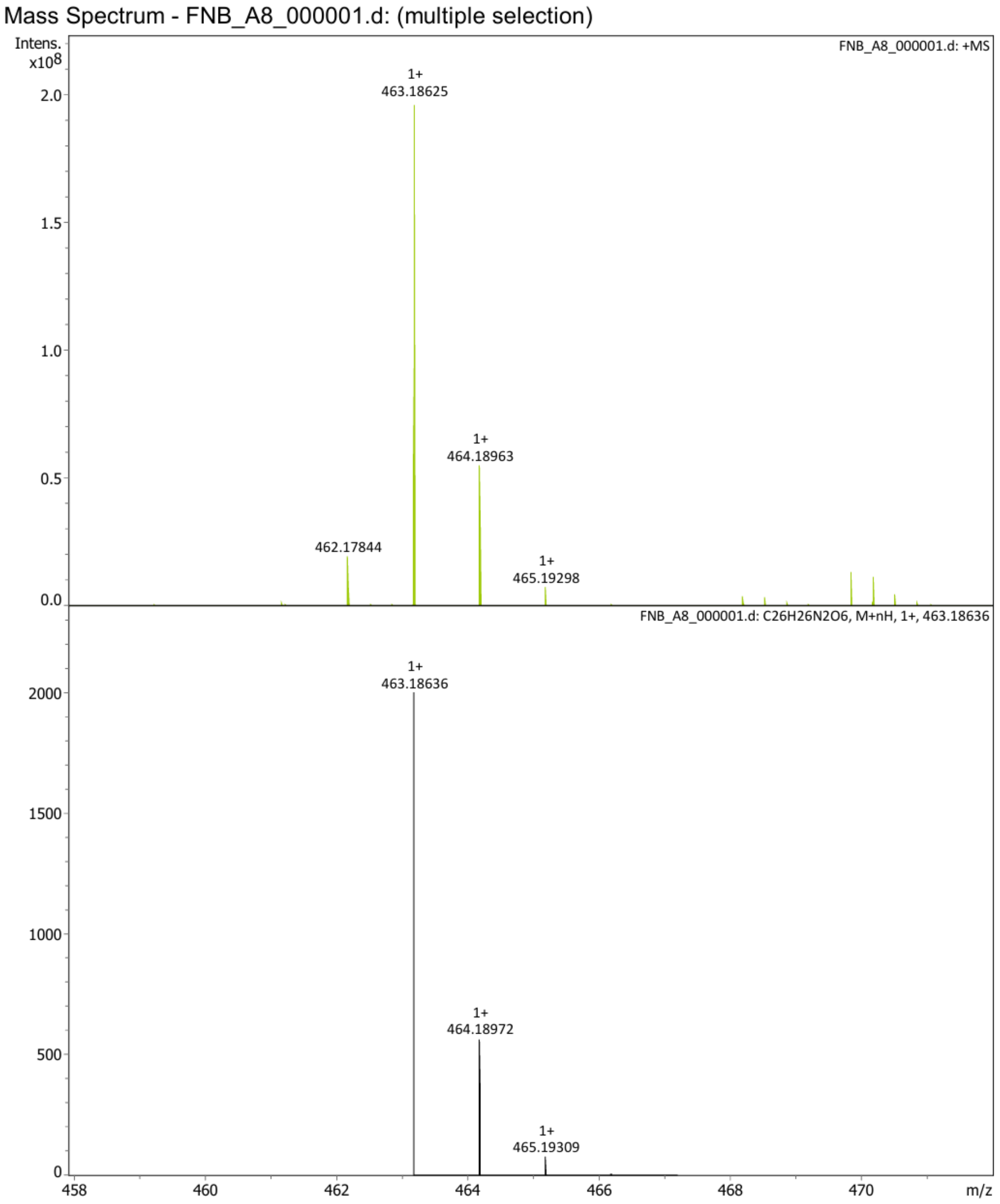

Mass Spectrum - FNB_A8_000001.d: (multiple selection)
Intens.
x10^8
FNB_A8_000001.d: +MS
1+
463.18625
1+
464.18963
462.17844
1+
465.19298
2.0
1.5
1.0
0.5
0.0
FNB_A8_000001.d: C26H26N2O6, M+nH, 1+, 463.18636
1+
463.18636
1+
464.18972
1+
465.19309
2000
1500
1000
500
0
458
460
462
464
466
468
470
m/z

Compound A9

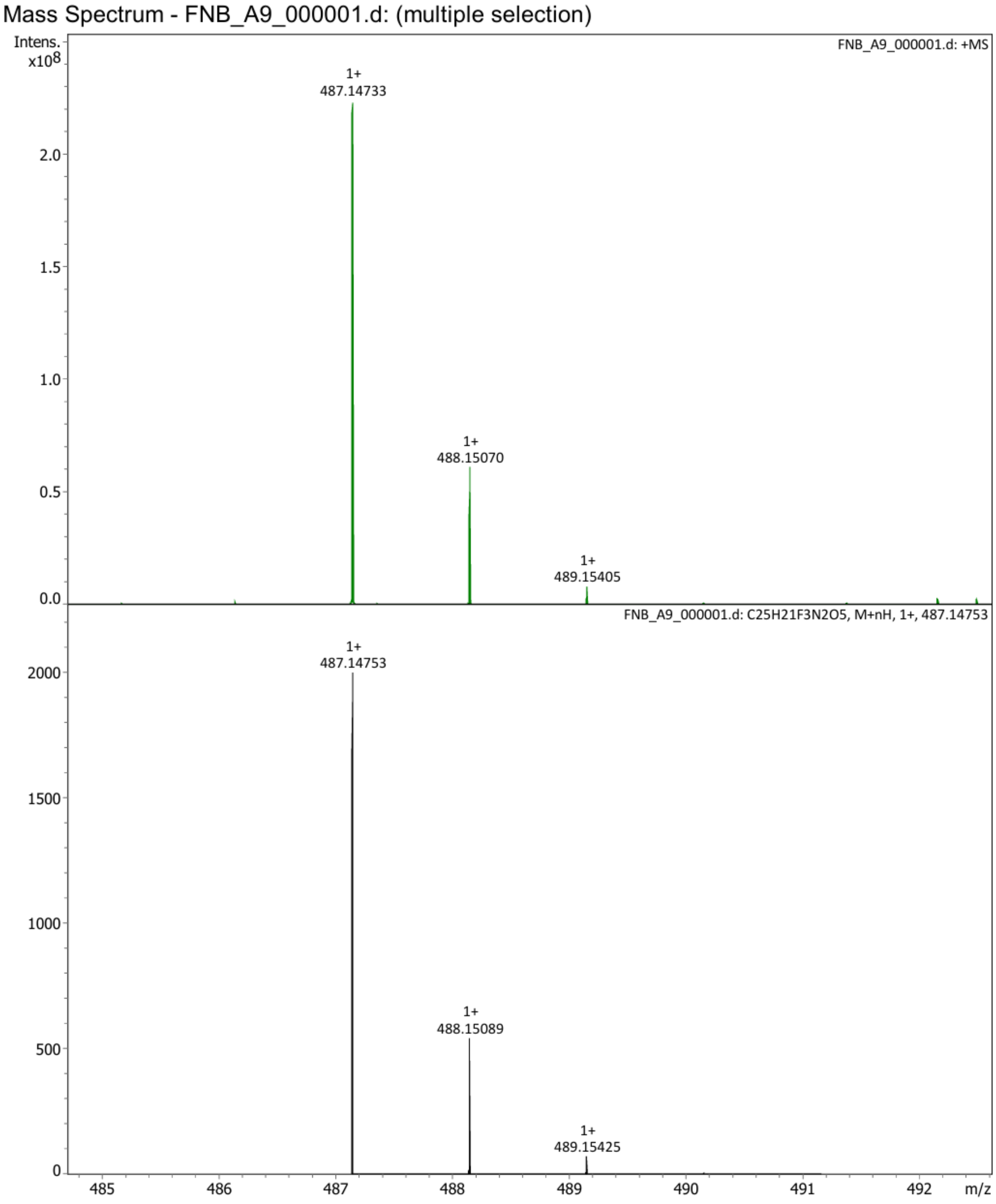

Mass Spectrum - FNB_A9_000001.d: (multiple selection)
Intens.
x10^8
FNB_A9_000001.d: +MS
1+
487.14733
1+
488.15070
1+
489.15405
FNB_A9_000001.d: C25H21F3N2O5, M+nH, 1+, 487.14753
1+
487.14753
1+
488.15089
1+
489.15425
m/z

Compound A10

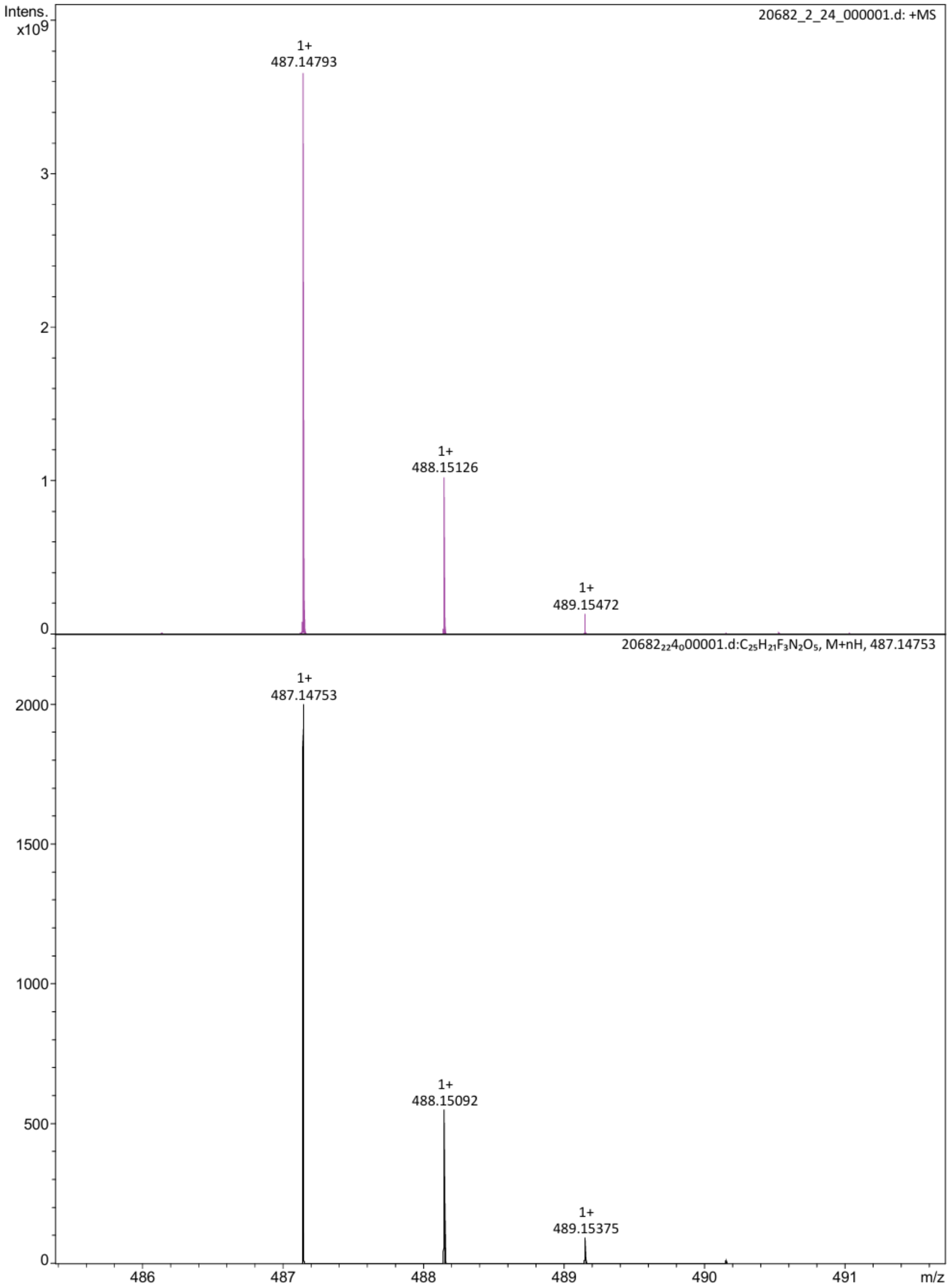

Compound A11

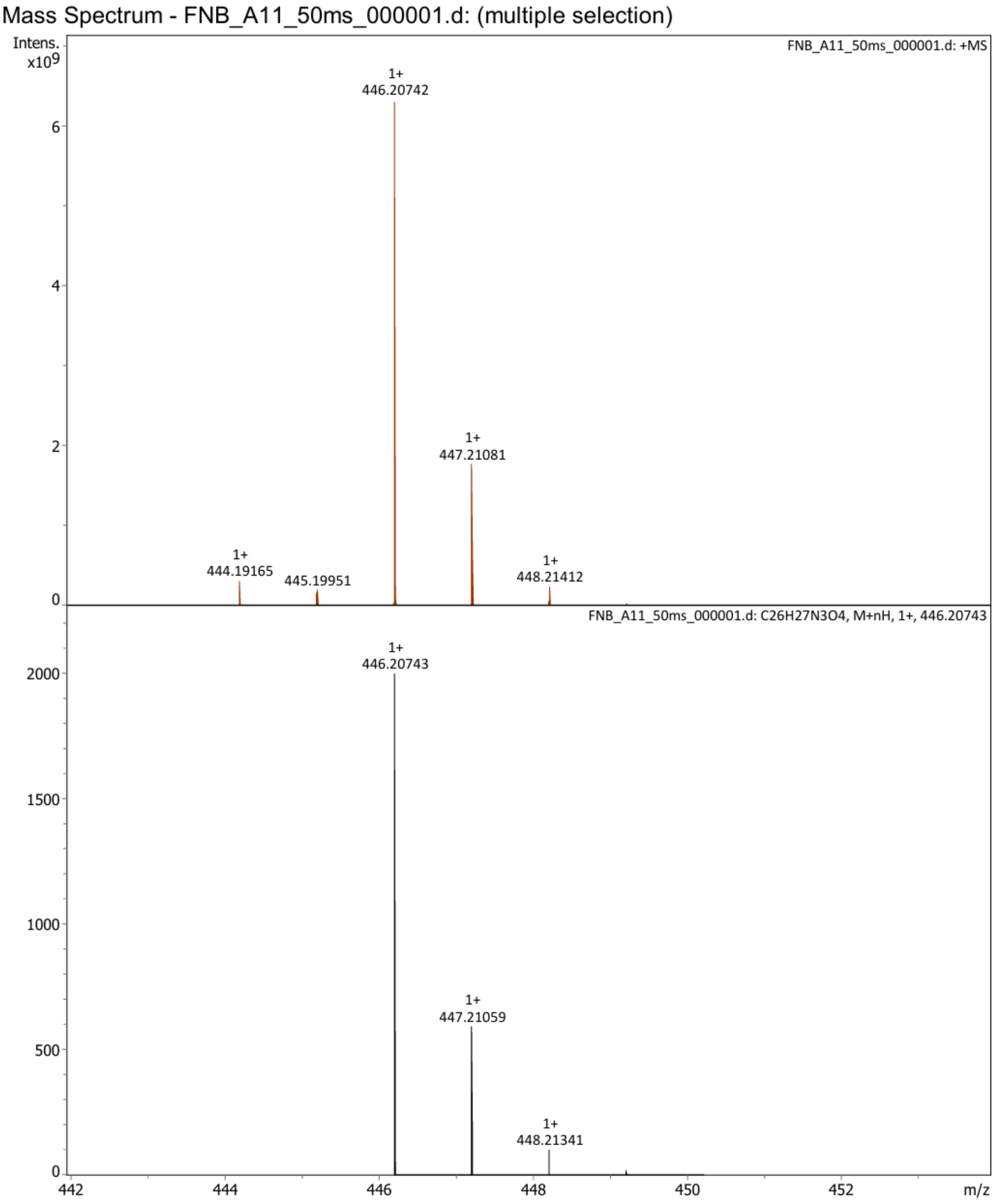

Compound A12

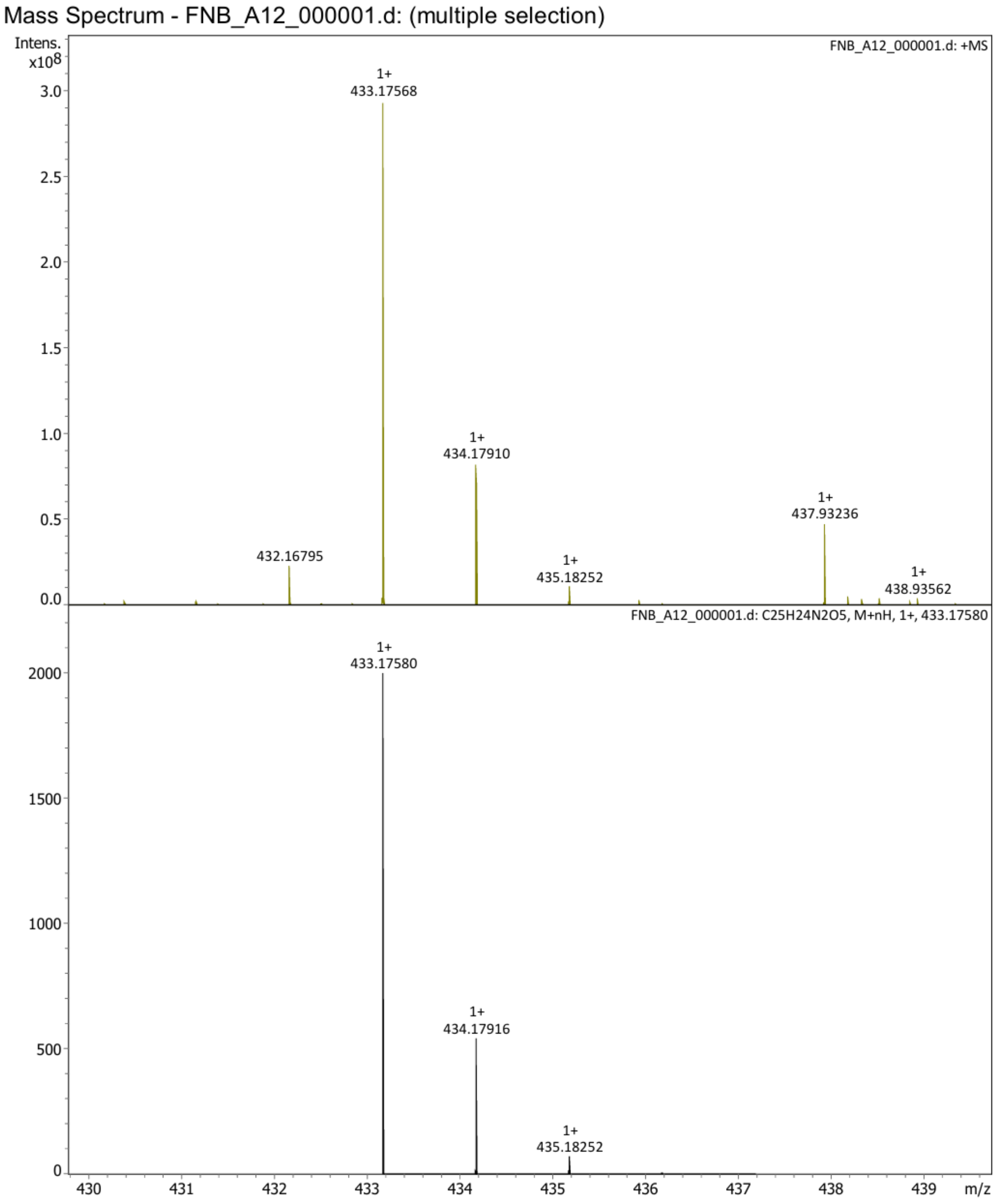

Compound A13

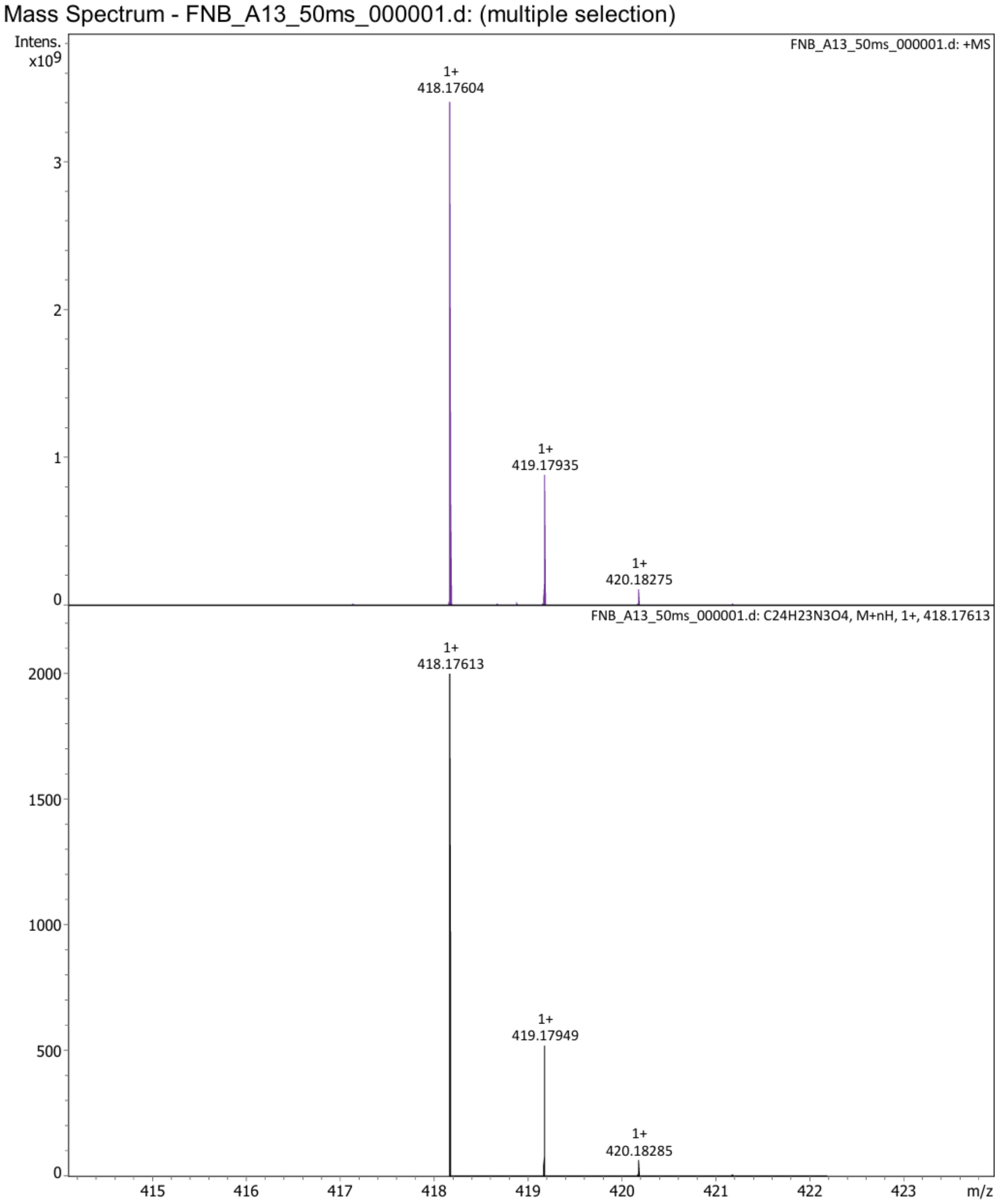

Compound A14

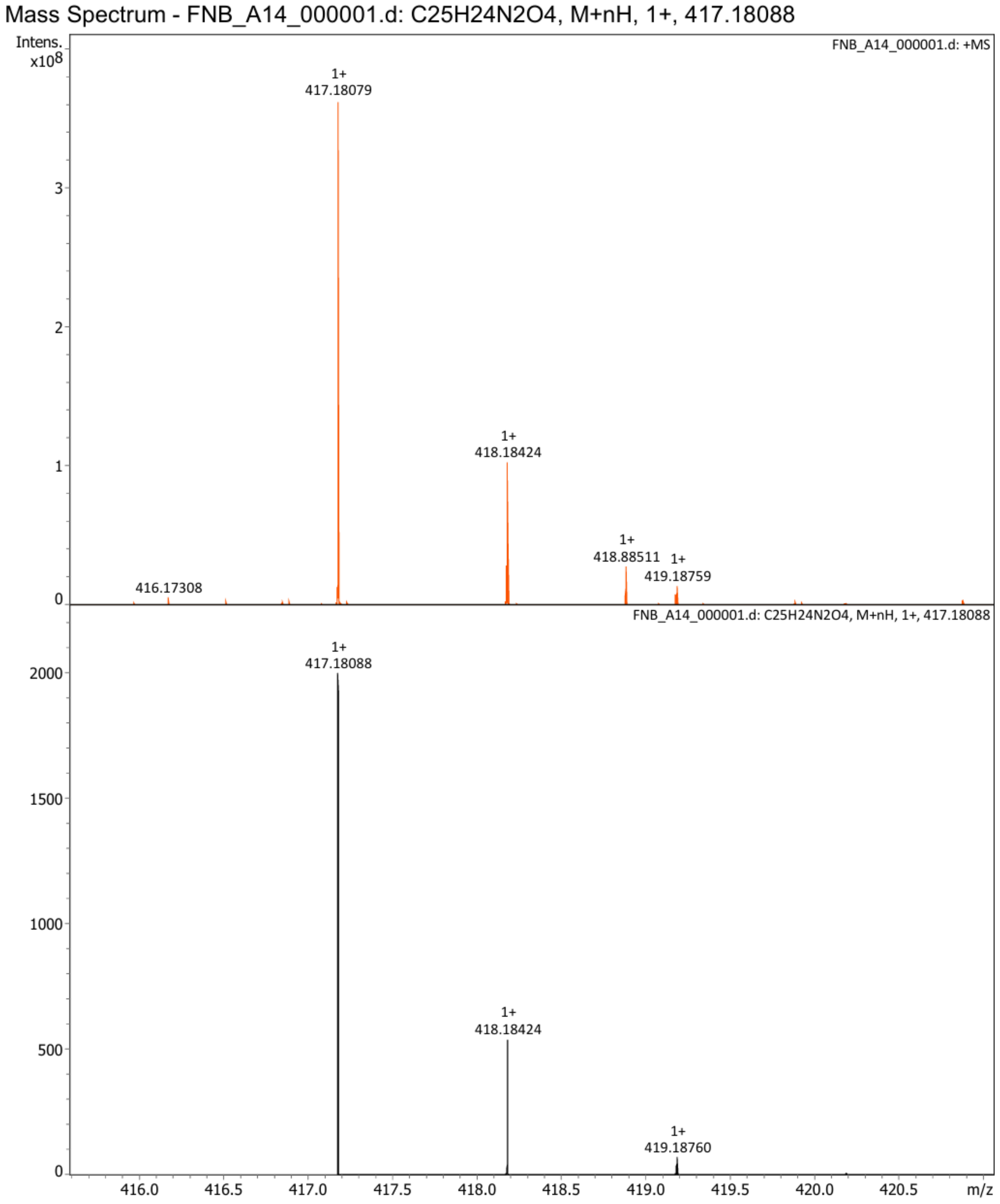

Compound A15

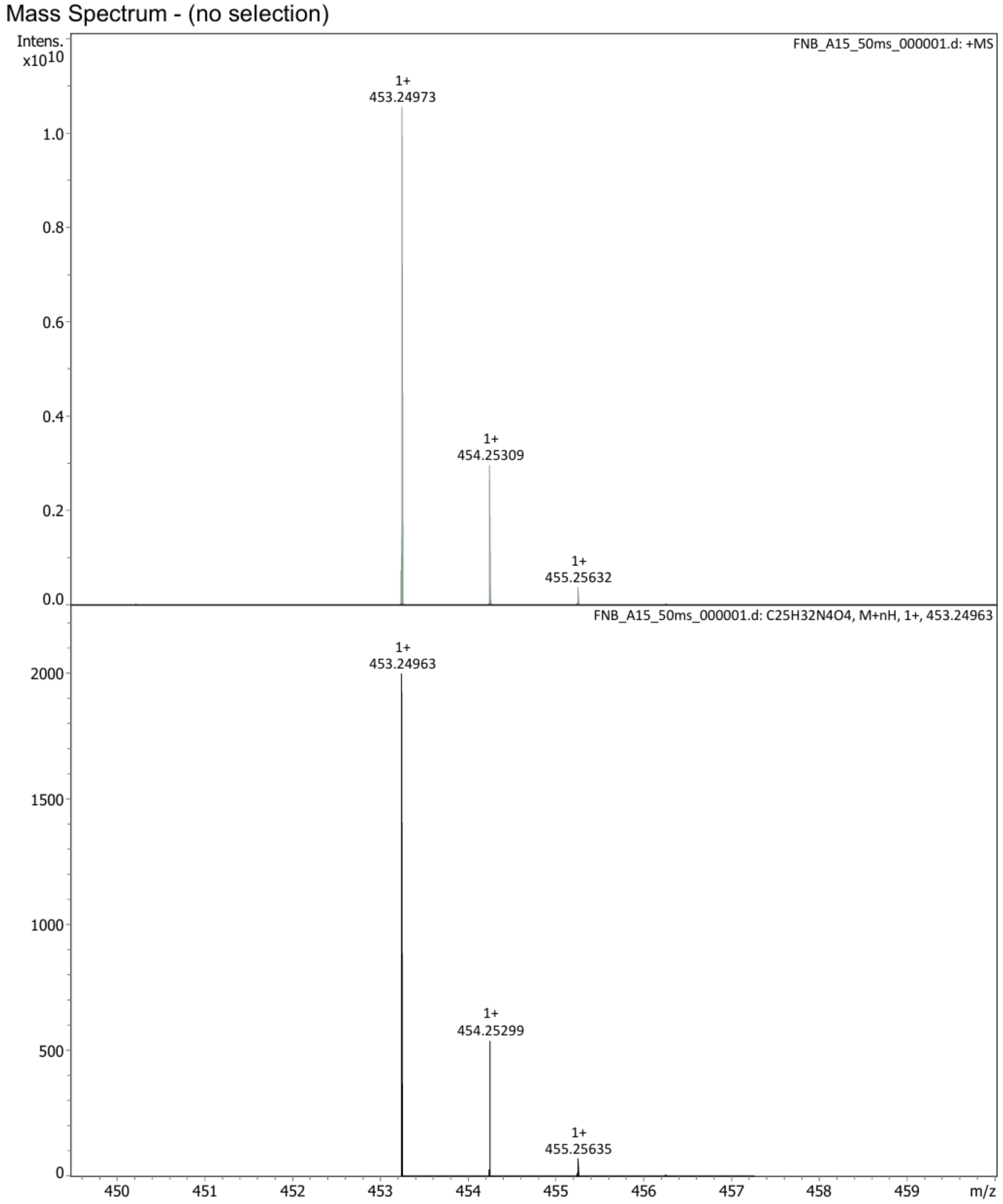

Compound A16

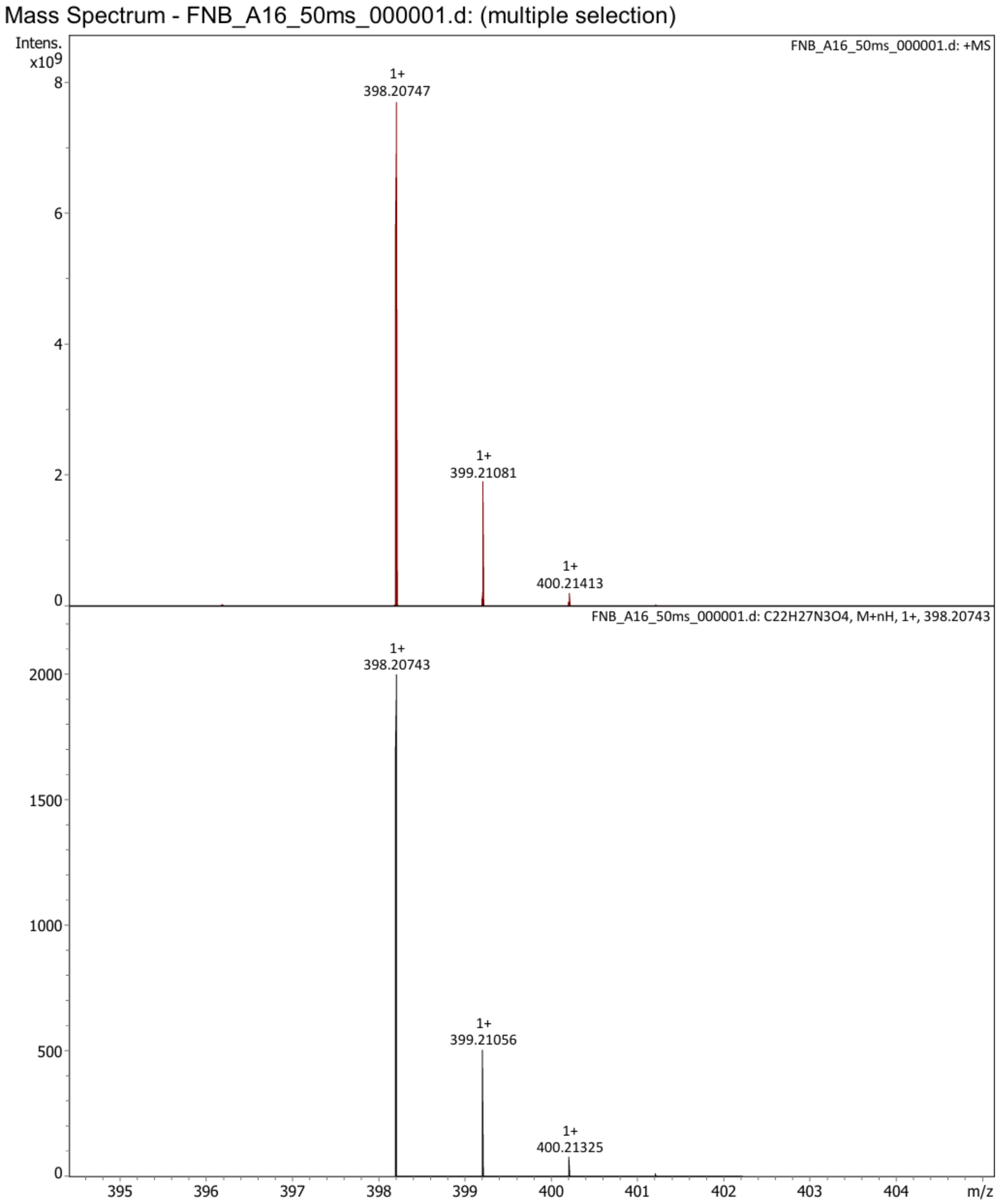

Compound A17

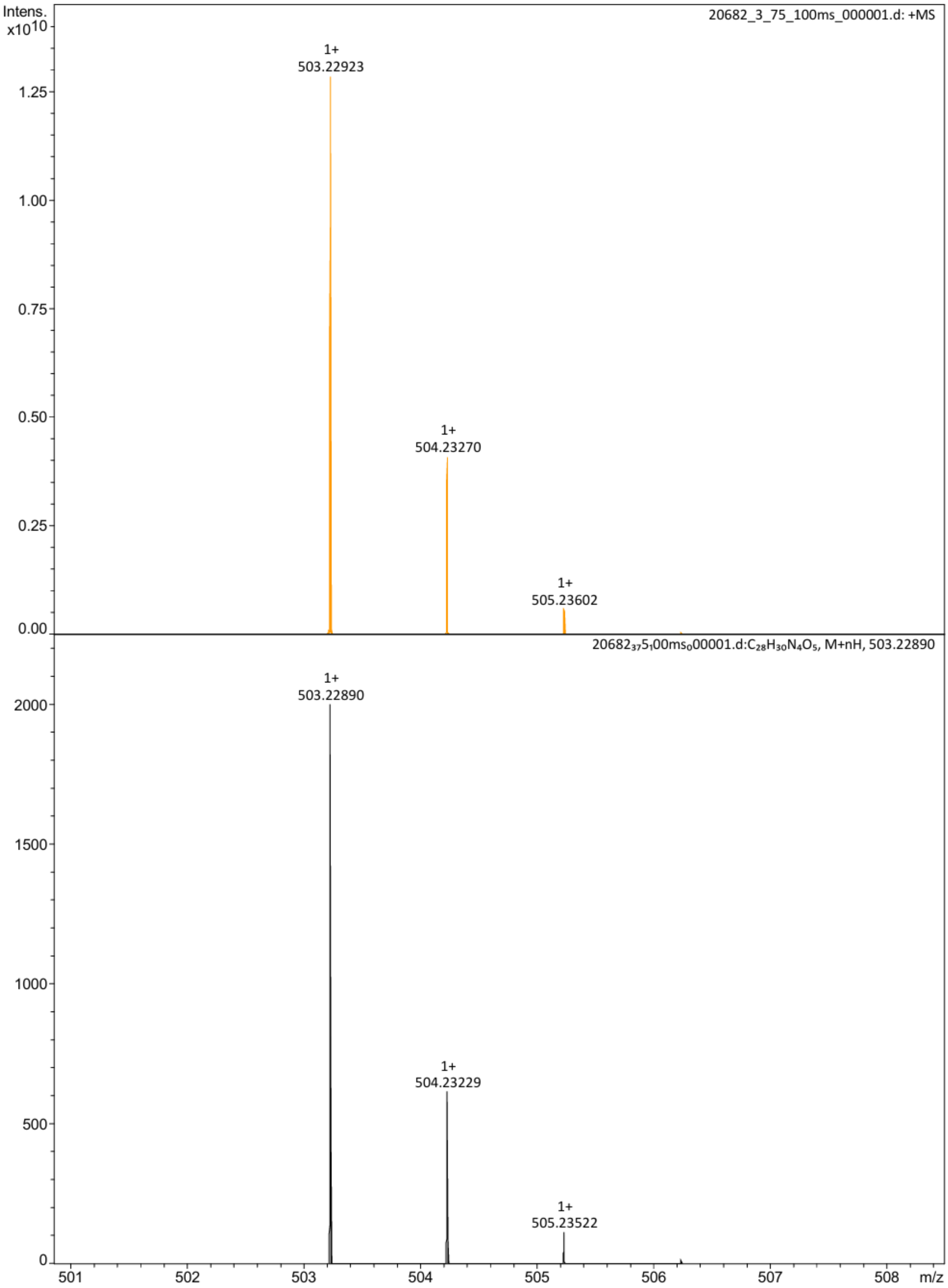

Compound A18

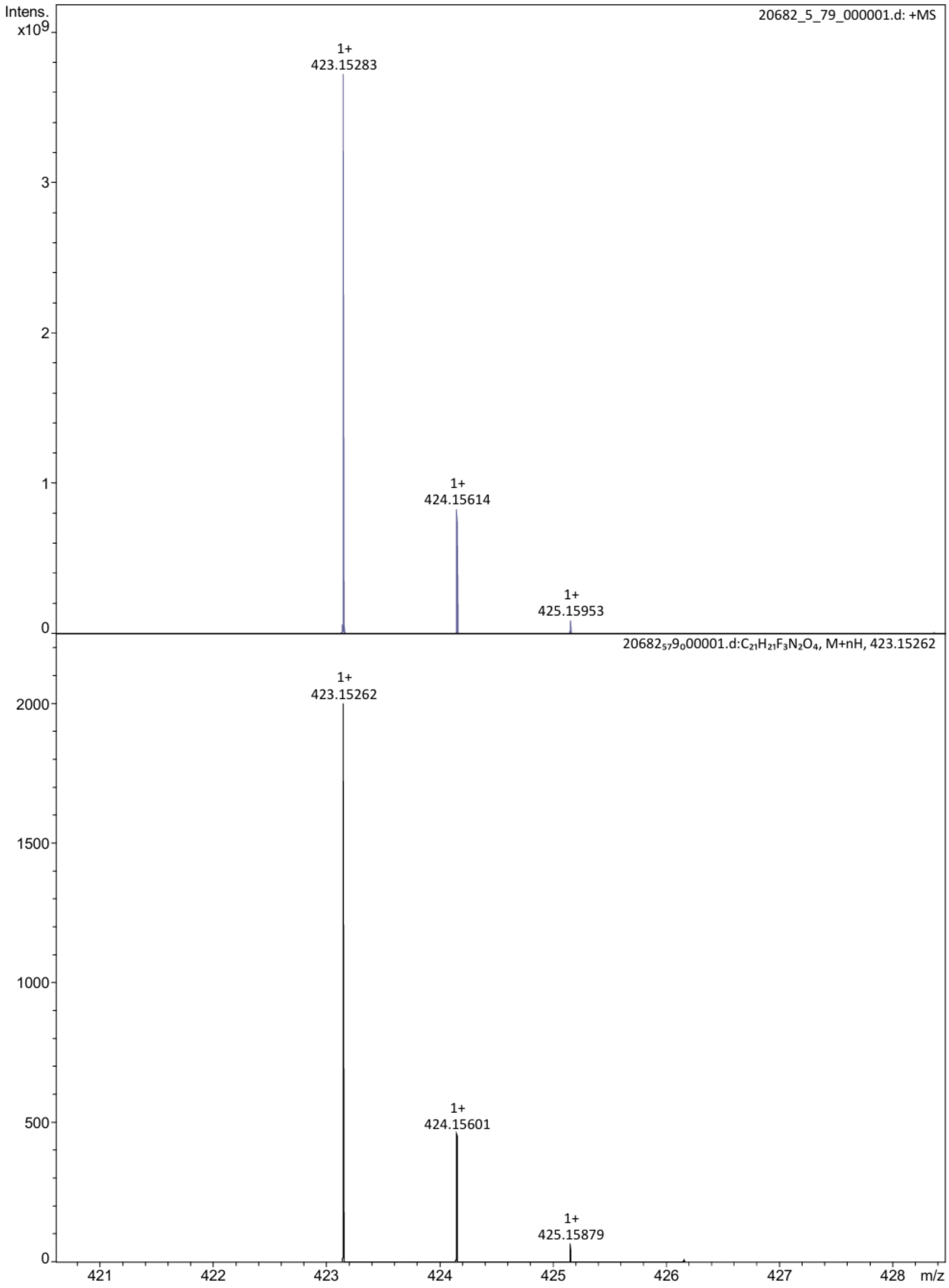

Compound A19

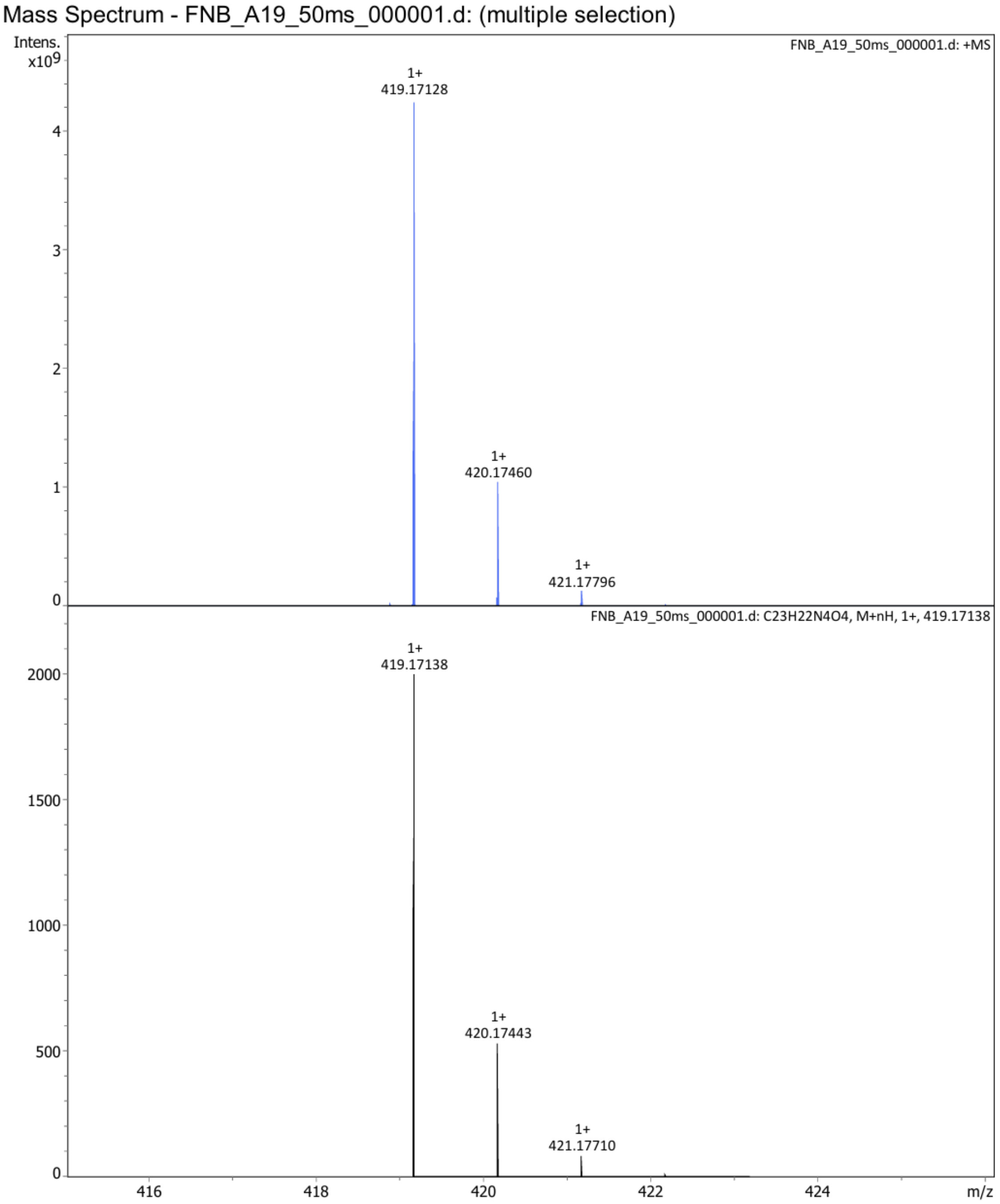

Compound A20

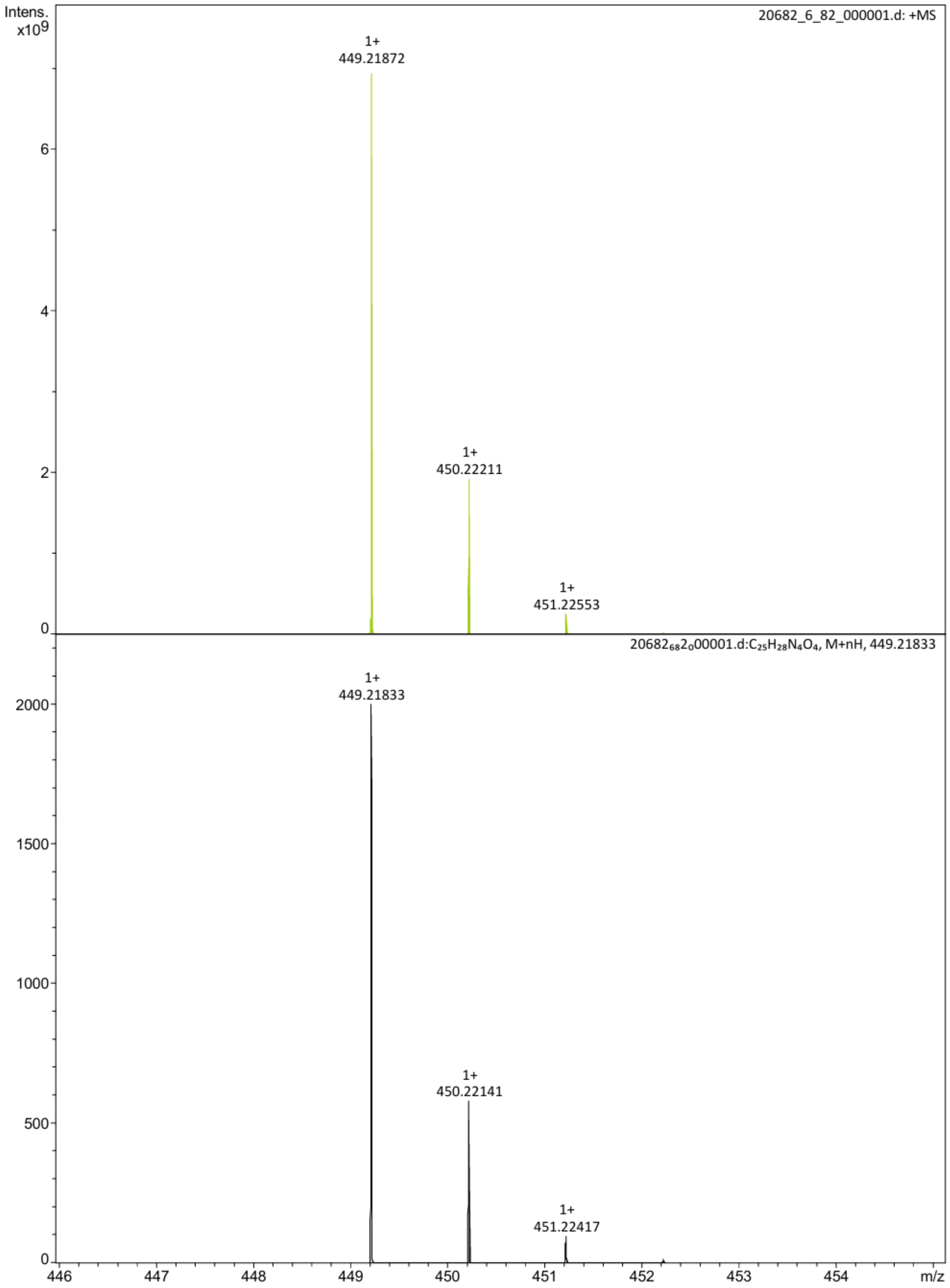

Compound A21

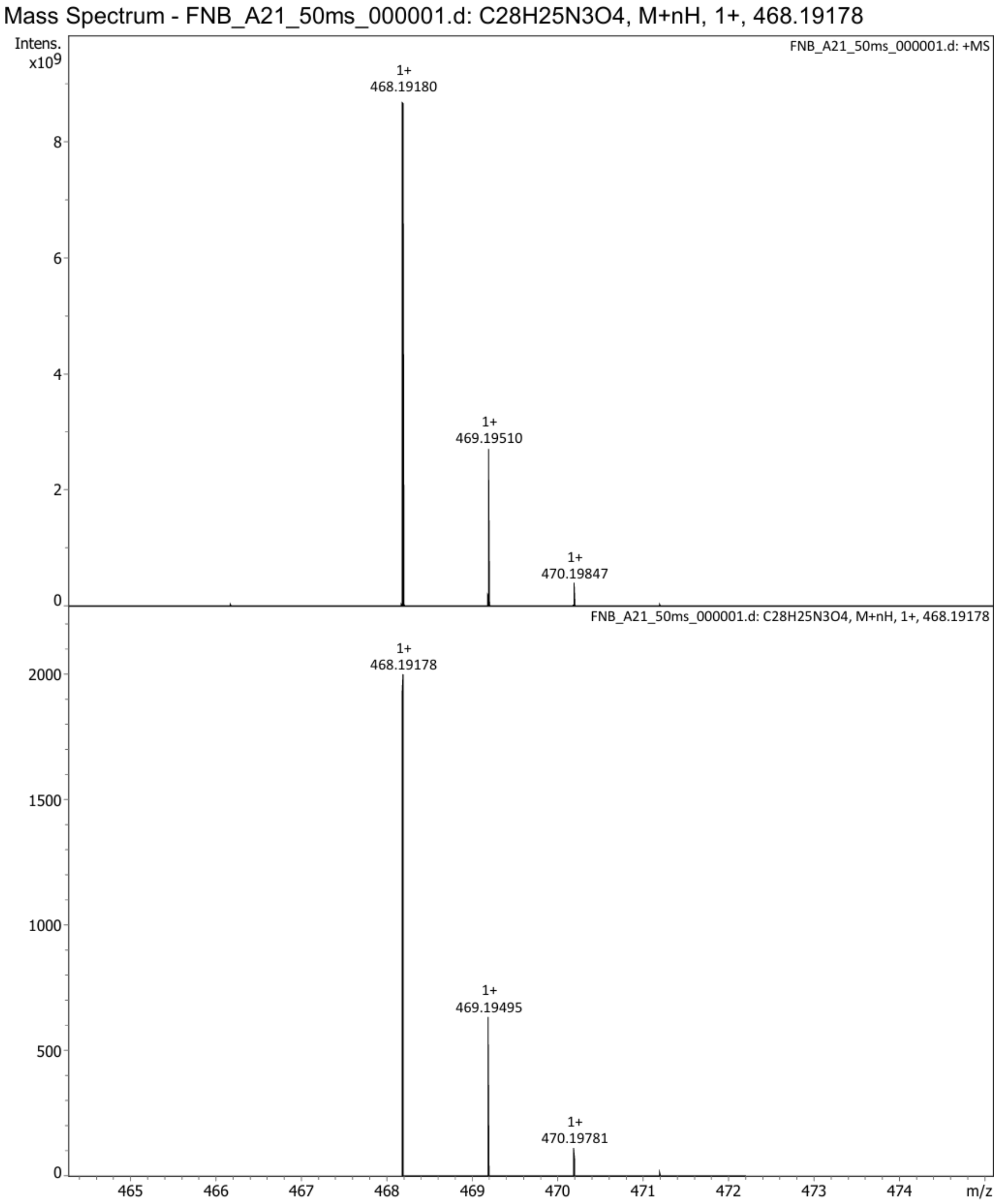

Compound A22

Mass Spectrum - FNB_A22_000001.d: (multiple selection)

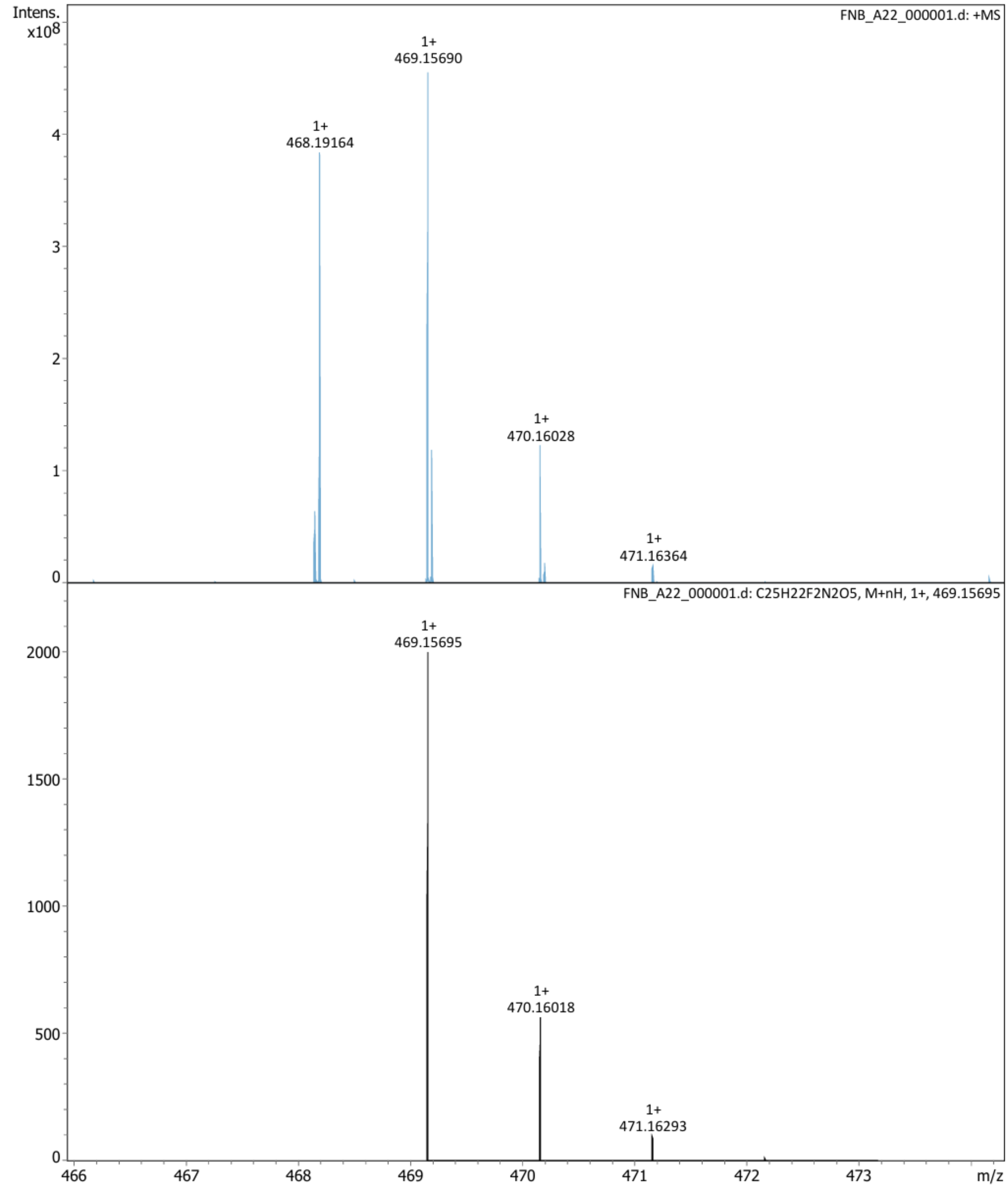

Compound A23

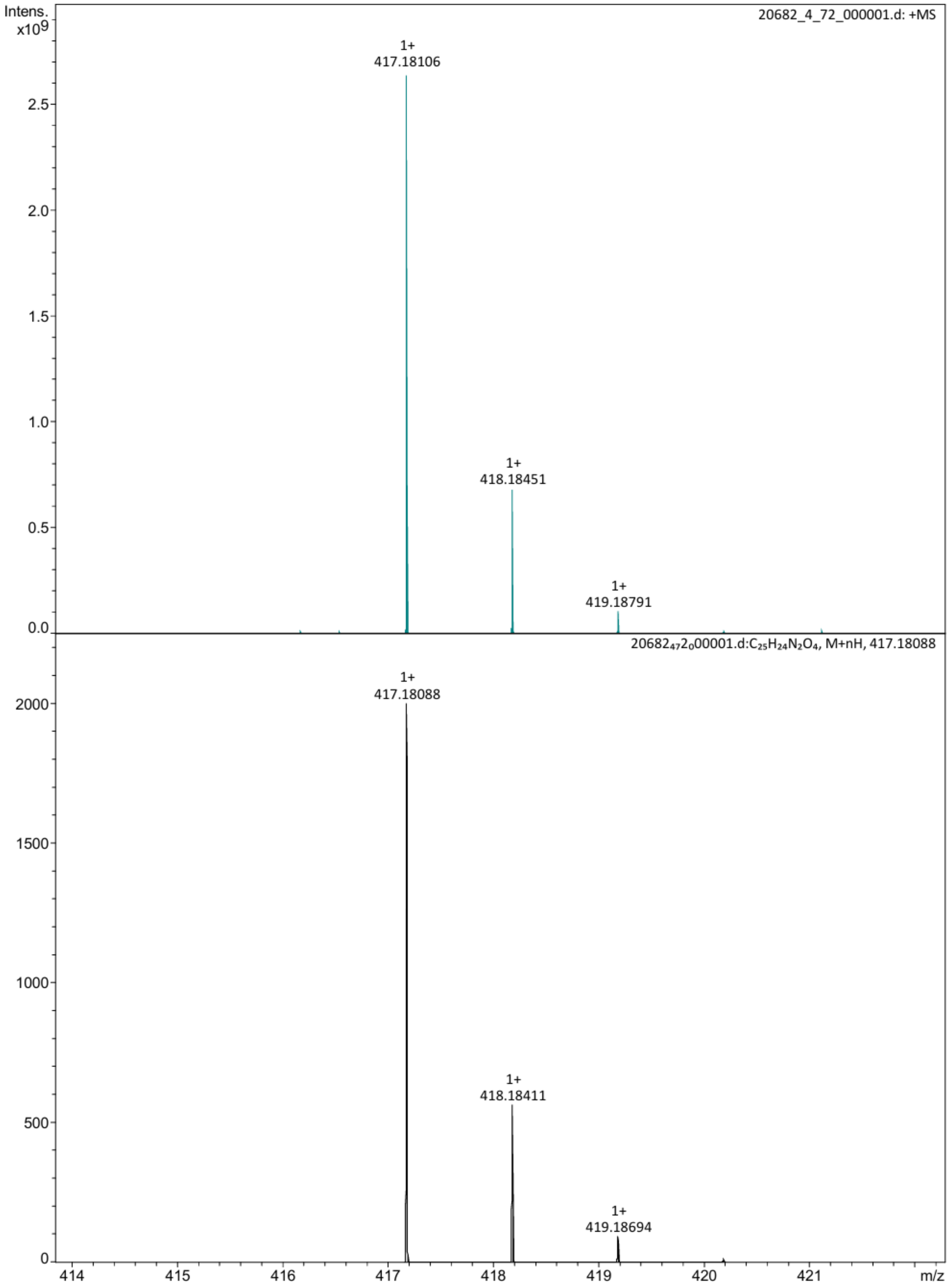

Compound A24

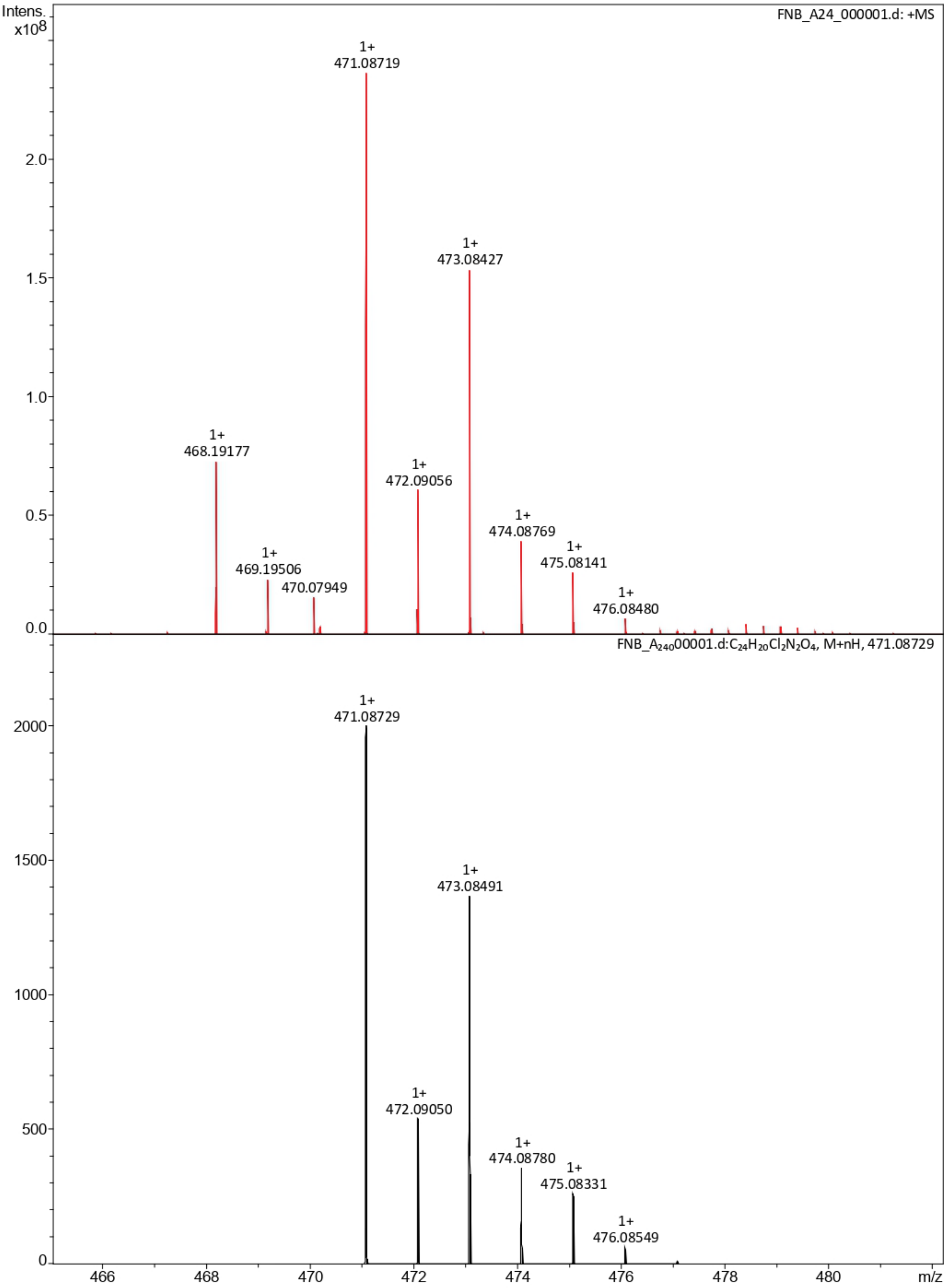

Compound A25

Mass Spectrum - FNB_A25_000001.d: (multiple selection)

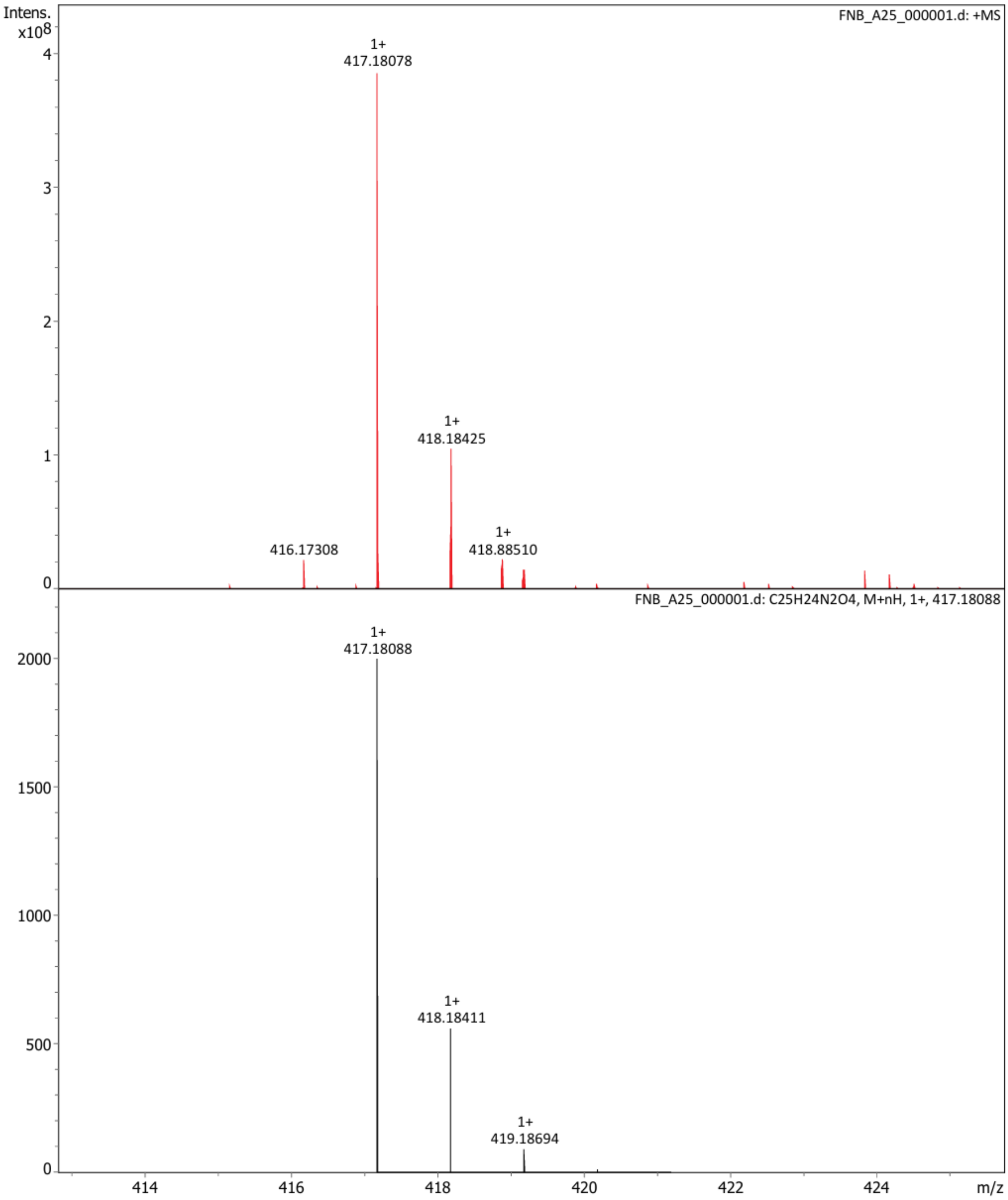

Compound A26

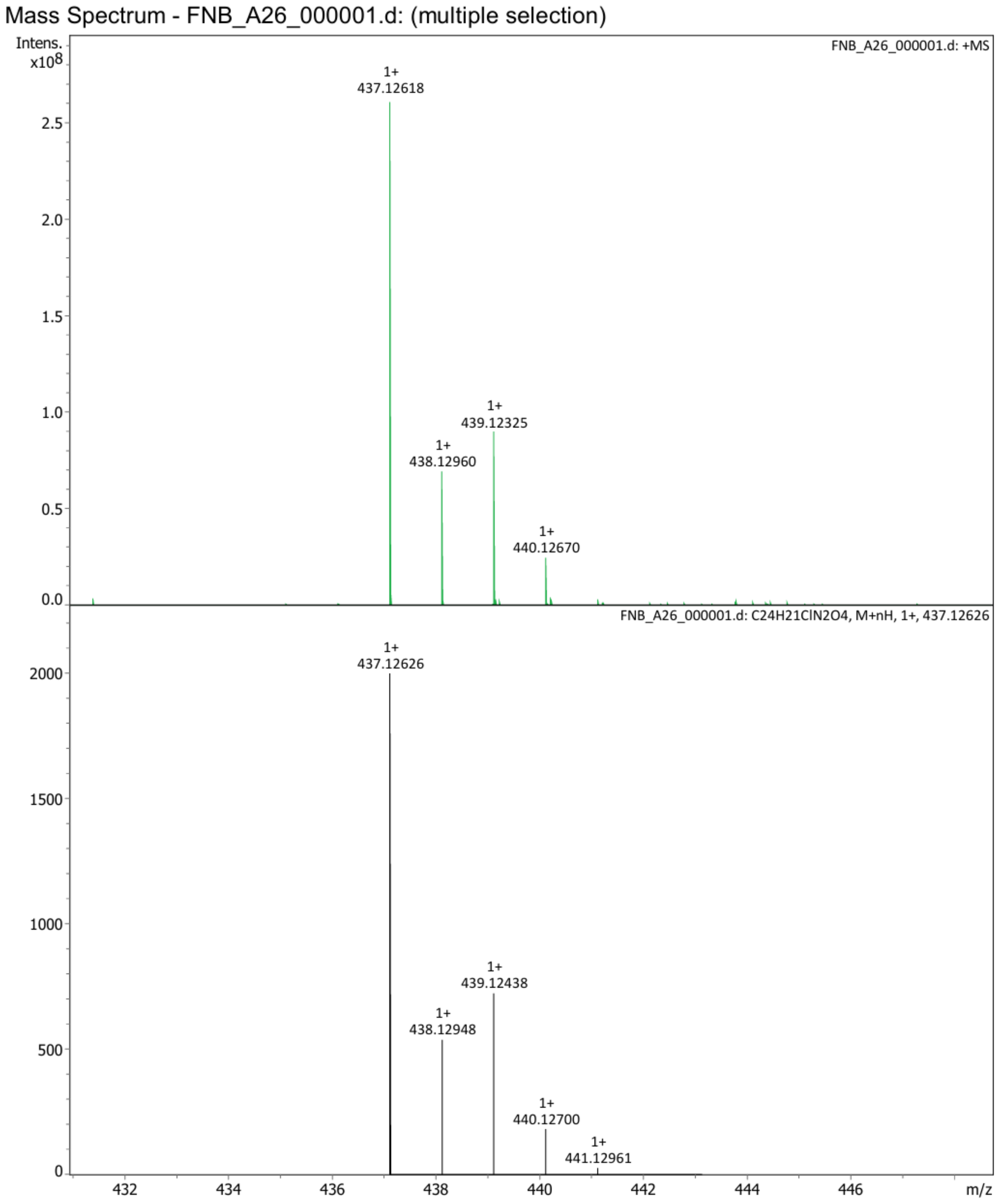

Compound A17-C

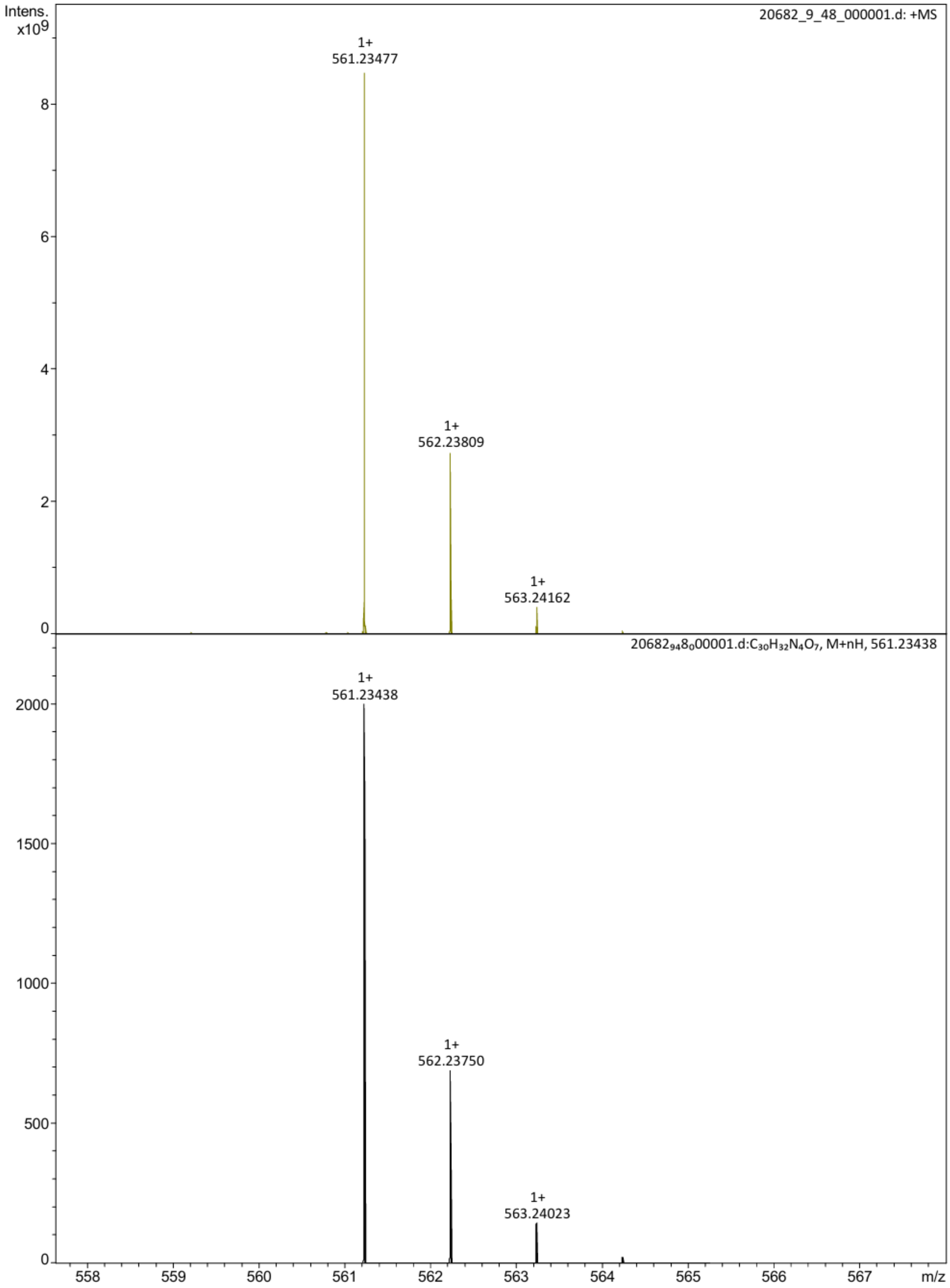

Compound A17-C-AA

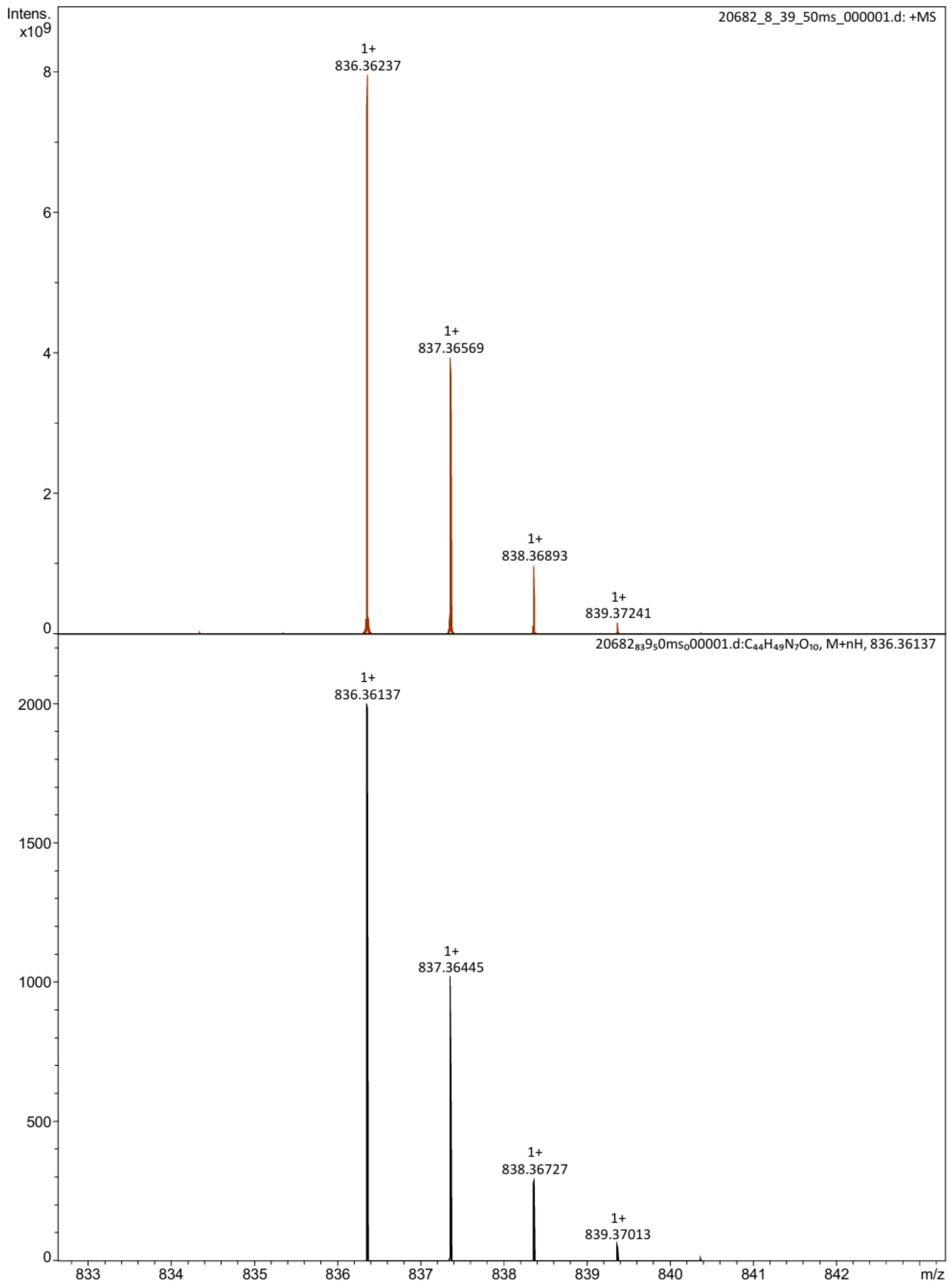

Compound A21-C

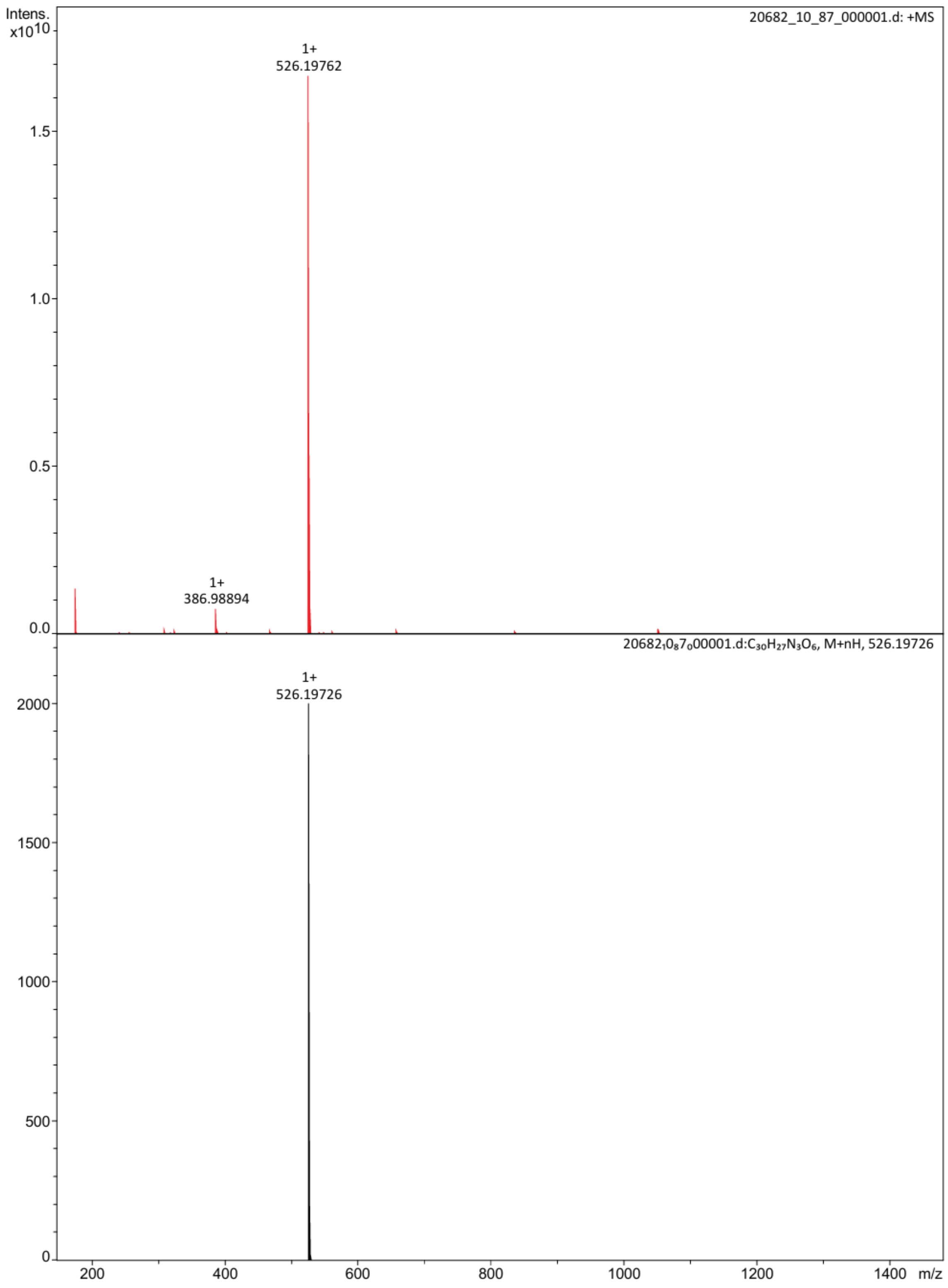

6. HPLC Traces

Compound A1

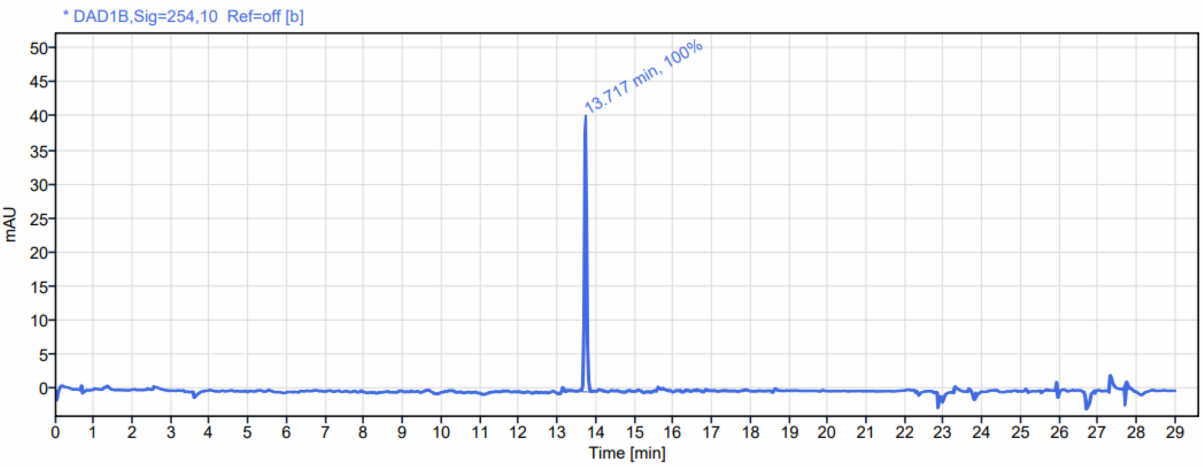

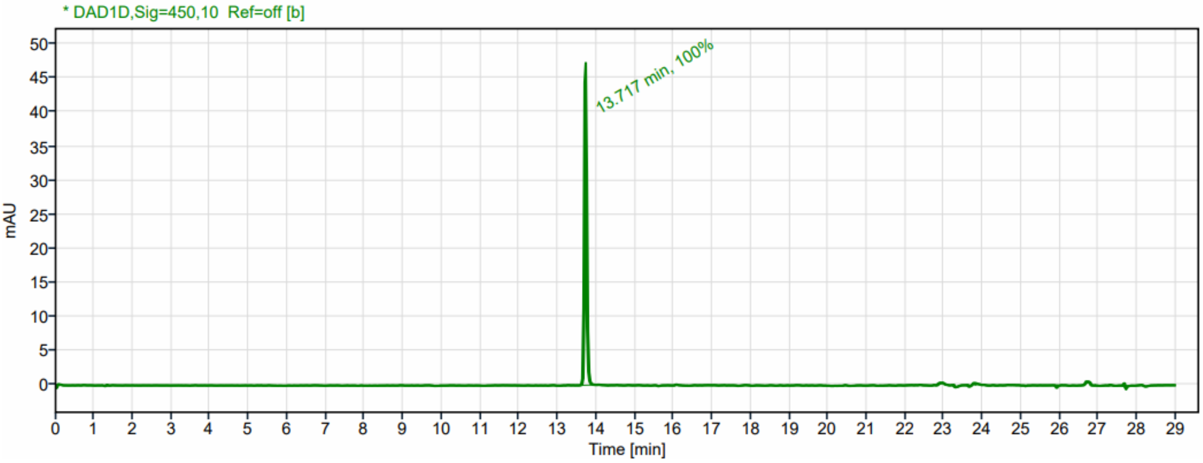

Compound A2

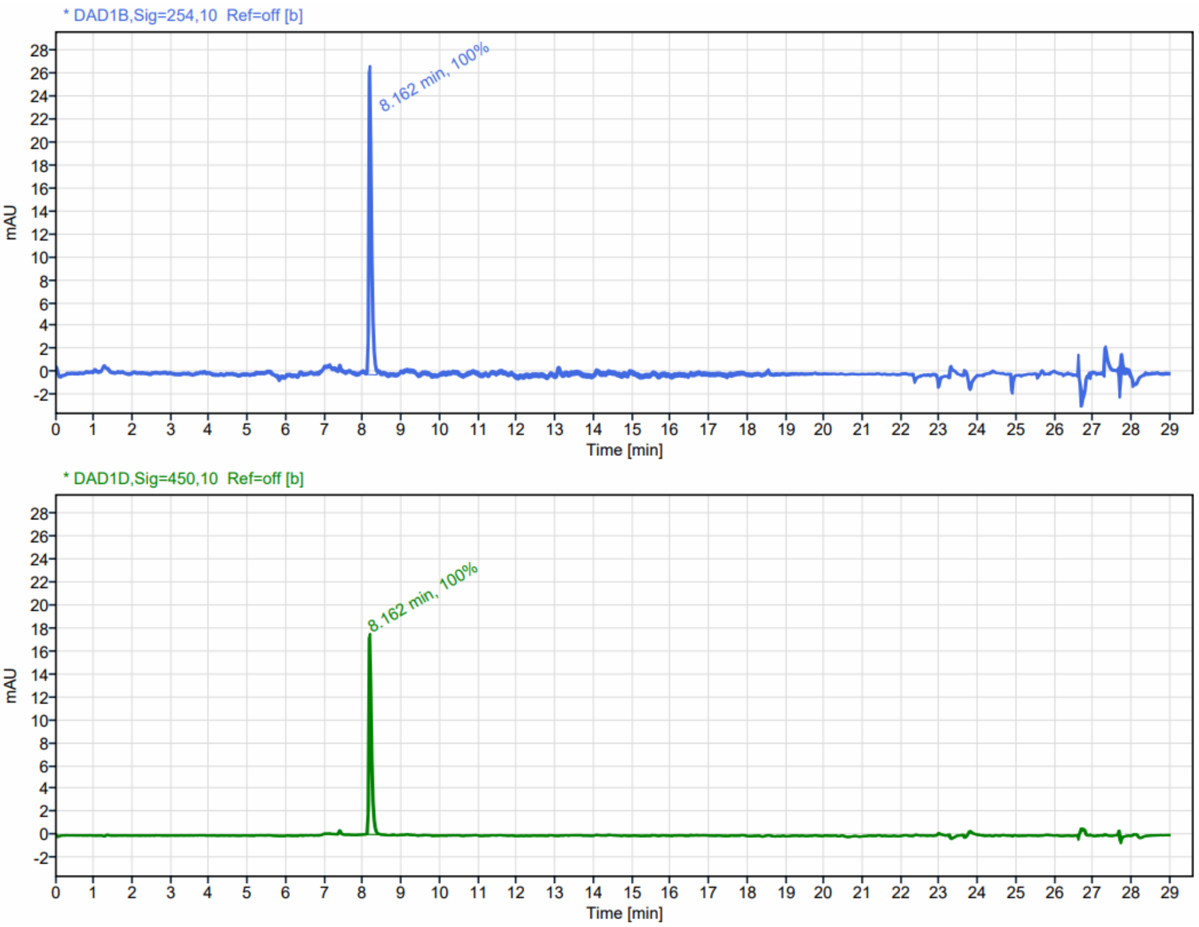

* DAD1B,Sig=254,10 Ref=off [b]
8.162 min, 100%
mAU
Time [min]
* DAD1D,Sig=450,10 Ref=off [b]
8.162 min, 100%
mAU
Time [min]

Compound A3

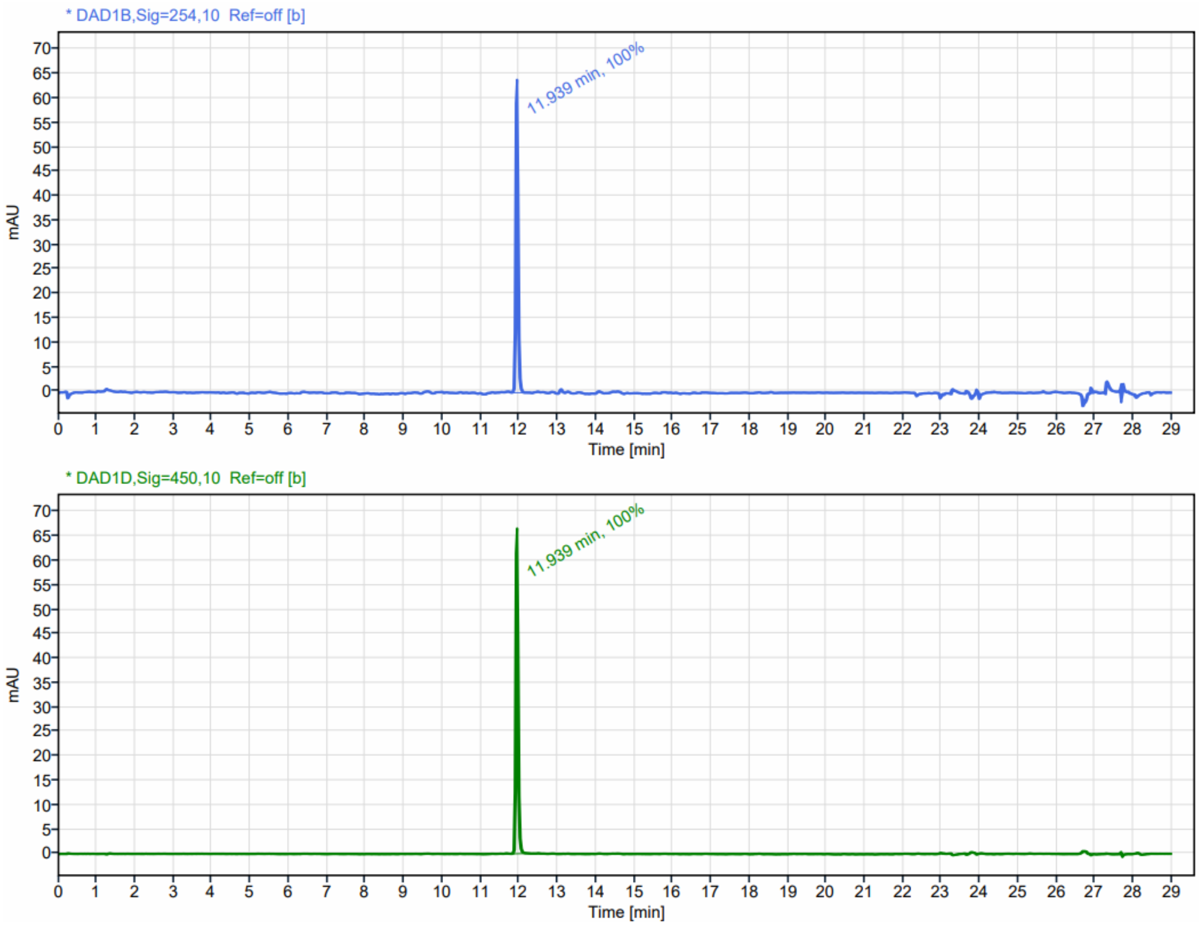

* DAD1B,Sig=254,10 Ref=off [b]
11.939 min, 100%
mAU
Time [min]
* DAD1D,Sig=450,10 Ref=off [b]
11.939 min, 100%
mAU
Time [min]

Compound A4

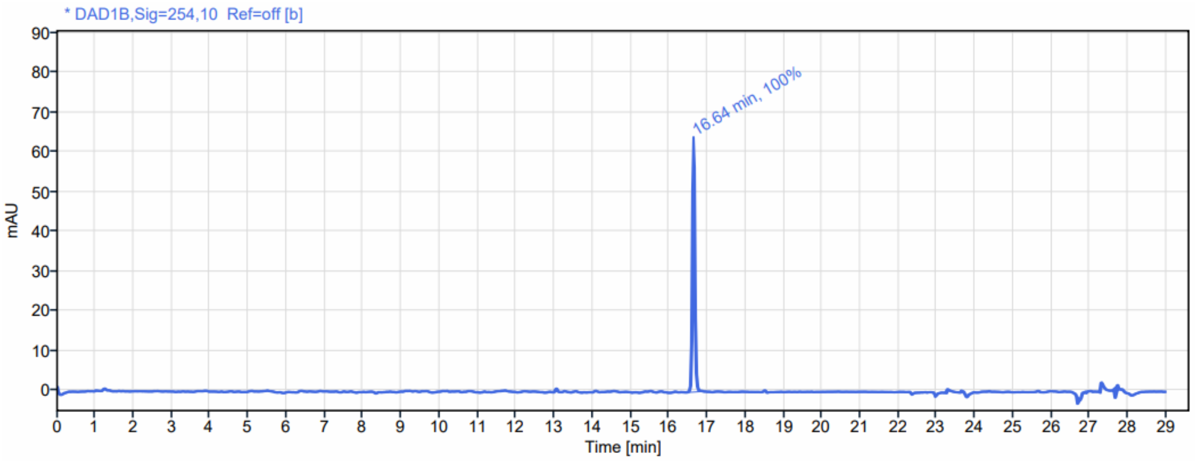

* DAD1B,Sig=254,10 Ref=off [b]
16.64 min, 100%
mAU
Time [min]

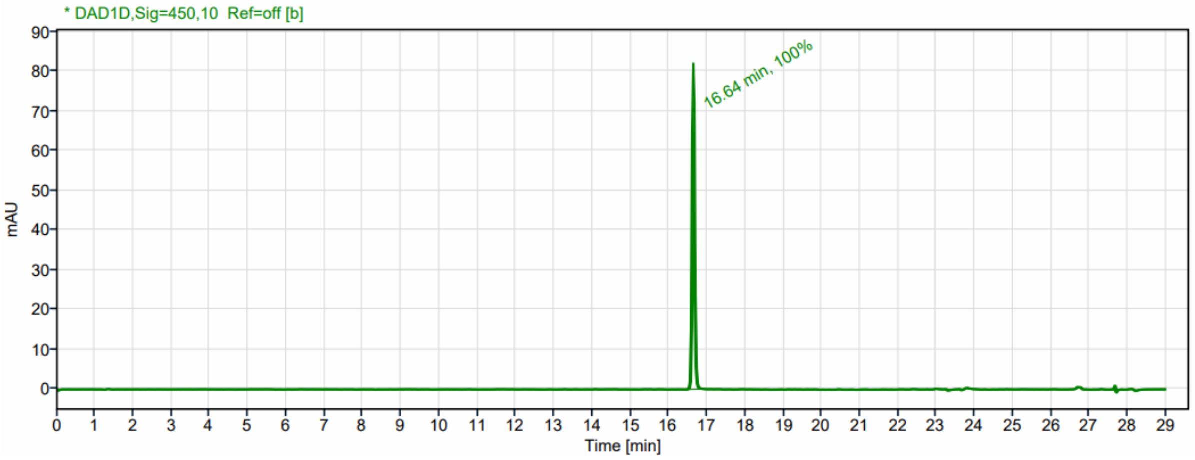

* DAD1D,Sig=450,10 Ref=off [b]
16.64 min, 100%
mAU
Time [min]

Compound A5

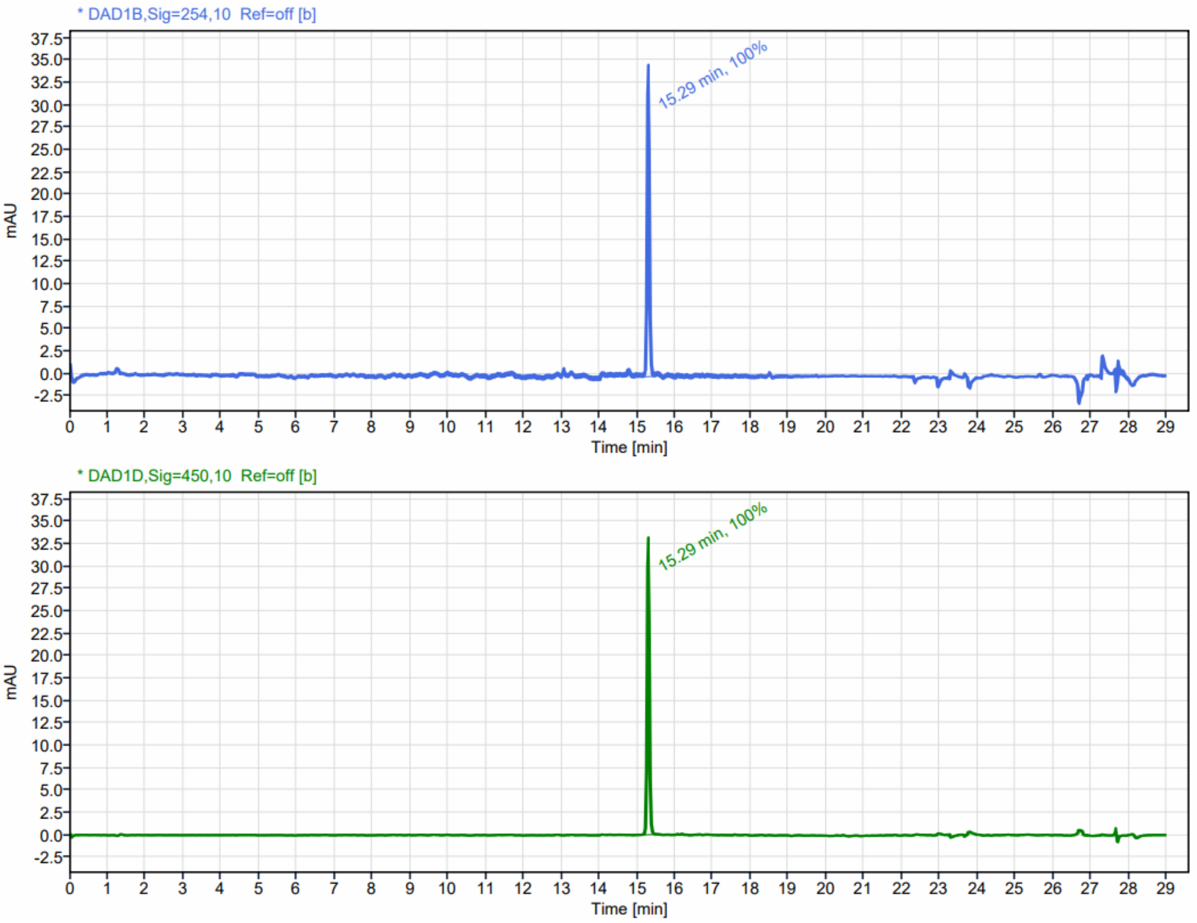

* DAD1B,Sig=254,10 Ref=off [b]
15.29 min, 100%
mAU
Time [min]
* DAD1D,Sig=450,10 Ref=off [b]
15.29 min, 100%
mAU
Time [min]

Compound A6

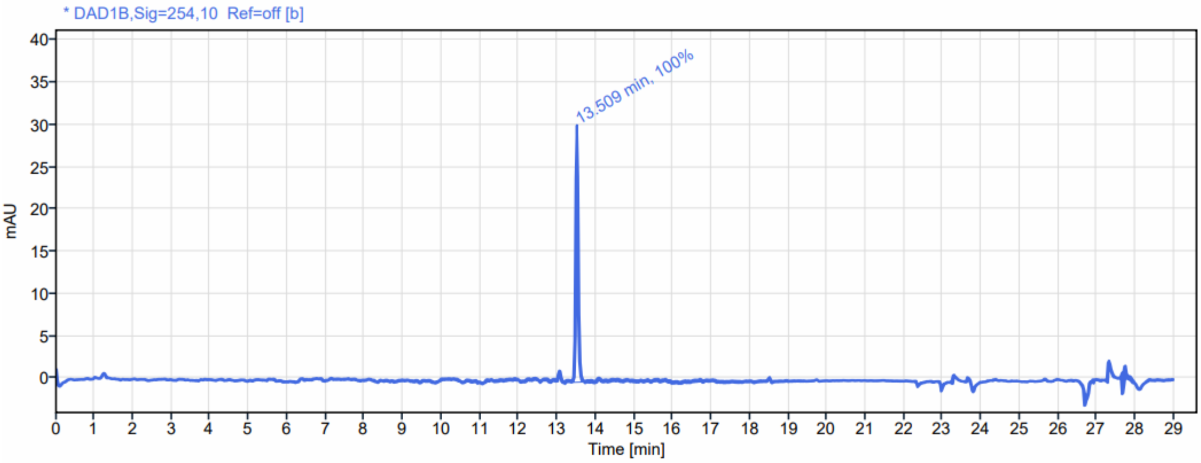
* DAD1B,Sig=254,10 Ref=off [b]
13.509 min, 100%
mAU
Time [min]

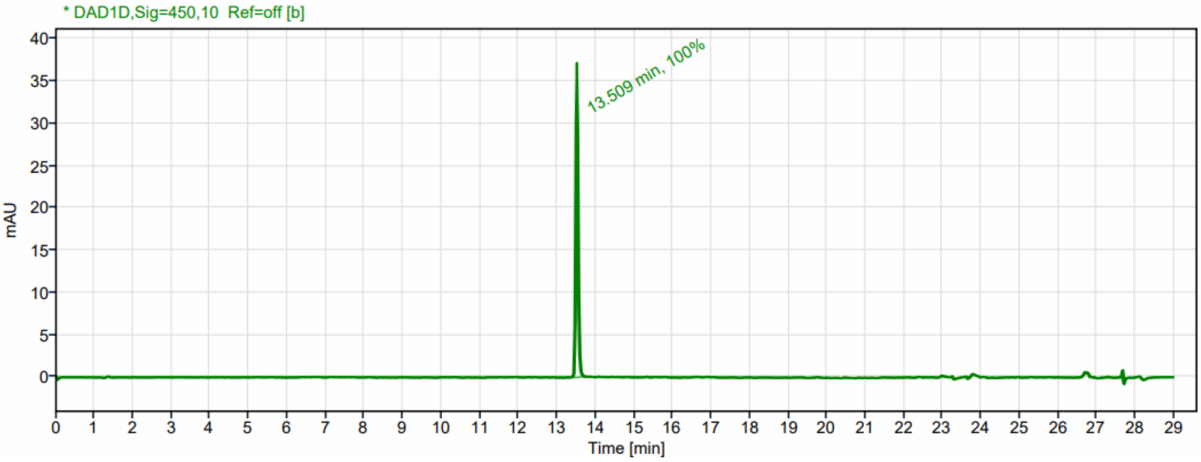
* DAD1D,Sig=450,10 Ref=off [b]
13.509 min, 100%
mAU
Time [min]

Compound A7

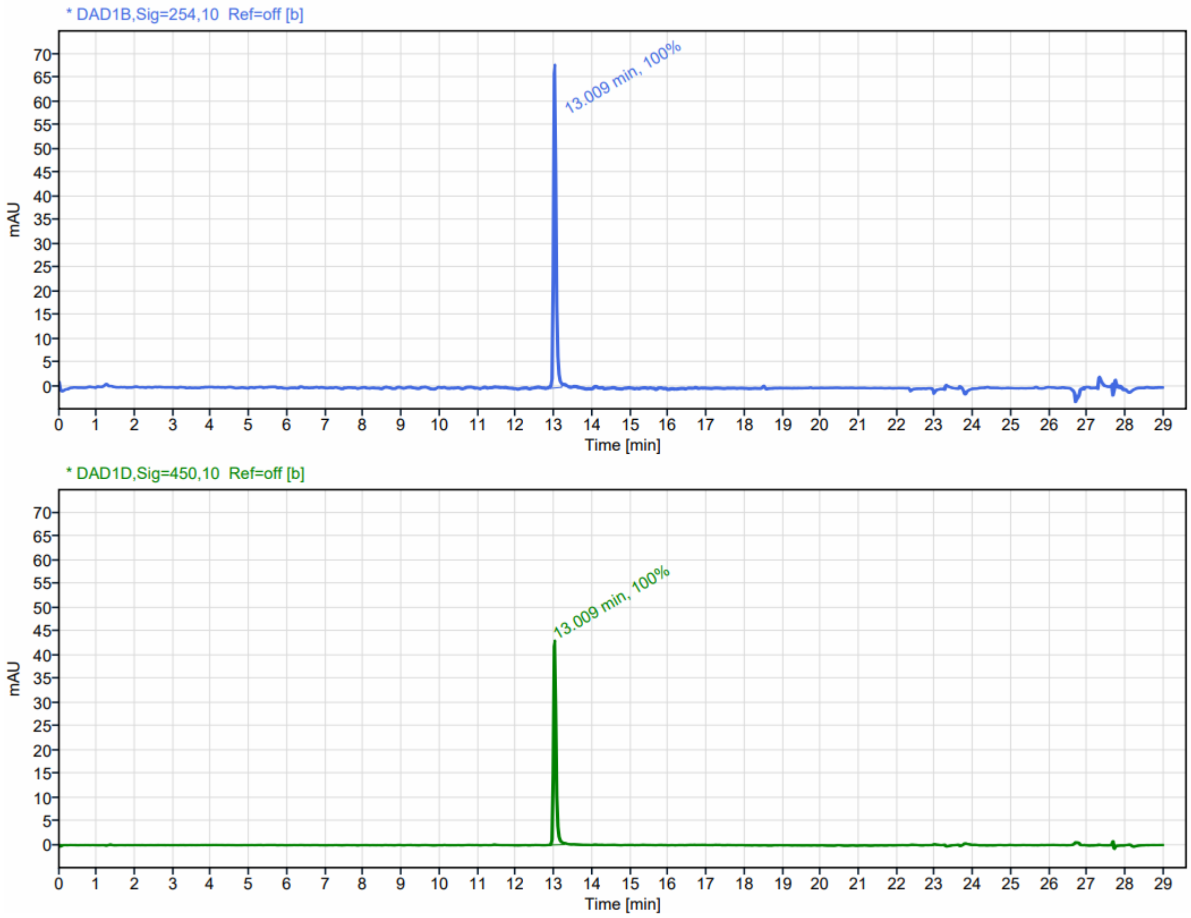

* DAD1B,Sig=254,10 Ref=off [b]
13.009 min, 100%
mAU
Time [min]
* DAD1D,Sig=450,10 Ref=off [b]
13.009 min, 100%
mAU
Time [min]

Compound A8

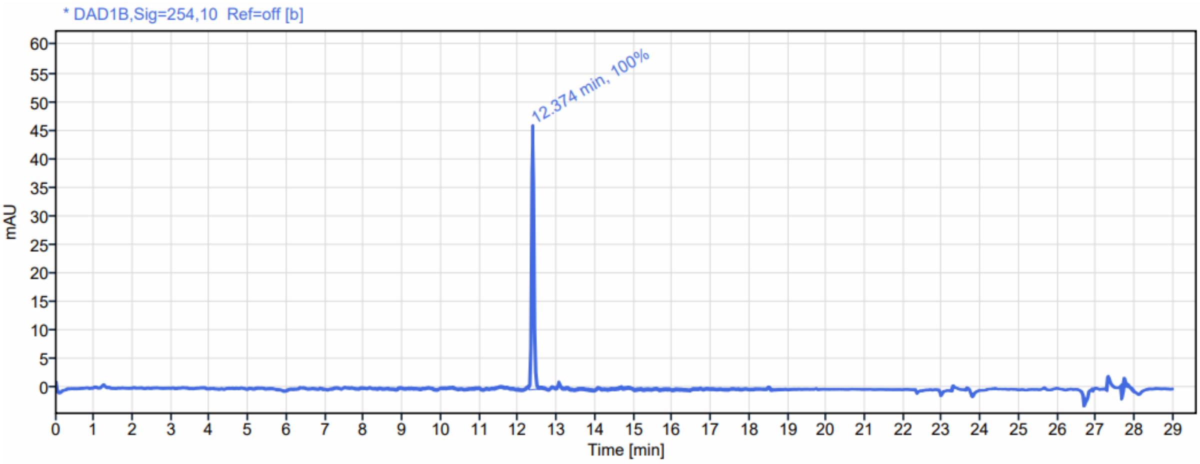

* DAD1B,Sig=254,10 Ref=off [b]
12.374 min, 100%
mAU
Time [min]

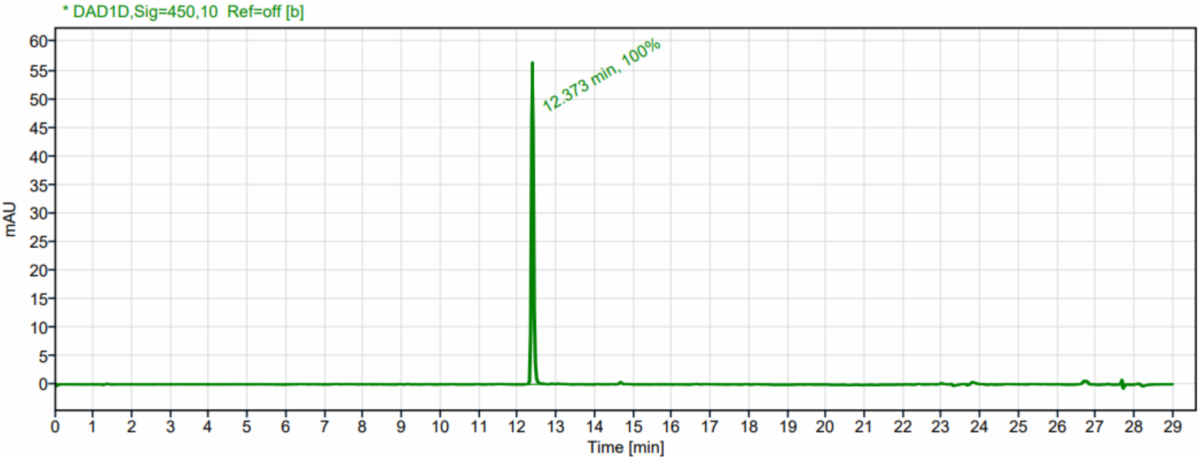

* DAD1D,Sig=450,10 Ref=off [b]
12.373 min, 100%
mAU
Time [min]

Compound A9

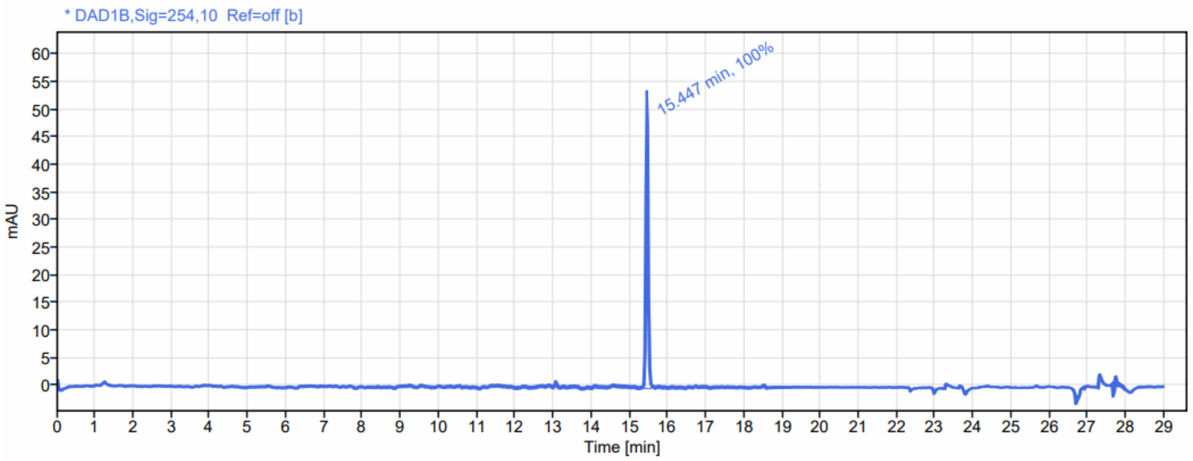
* DAD1B,Sig=254,10 Ref=off [b]
15.447 min, 100%
mAU
Time [min]

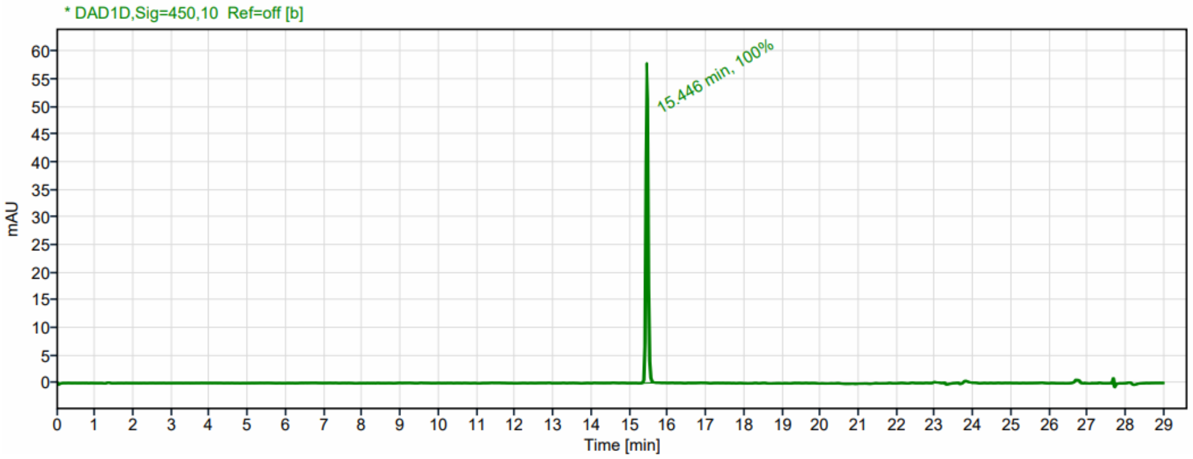
* DAD1D,Sig=450,10 Ref=off [b]
15.446 min, 100%
mAU
Time [min]

Compound A10

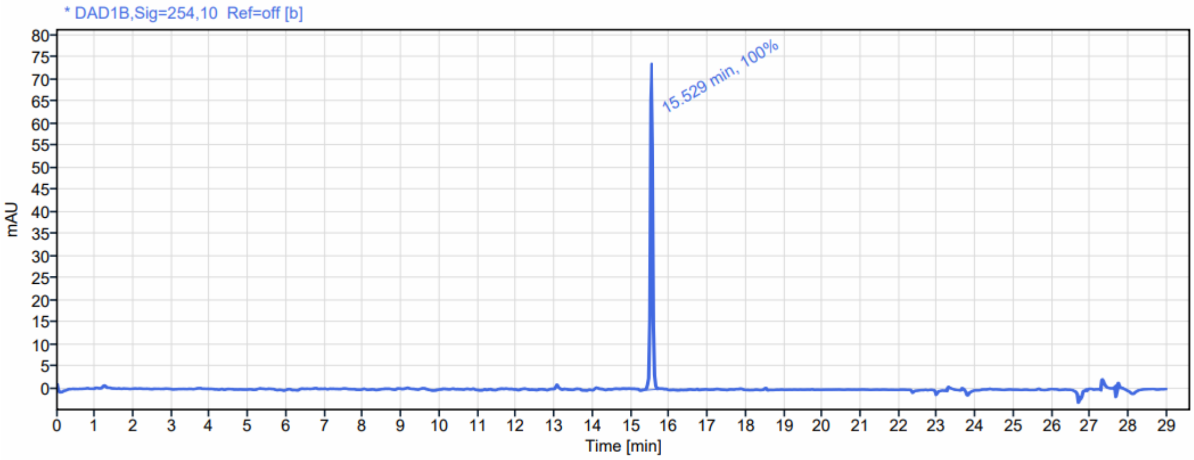

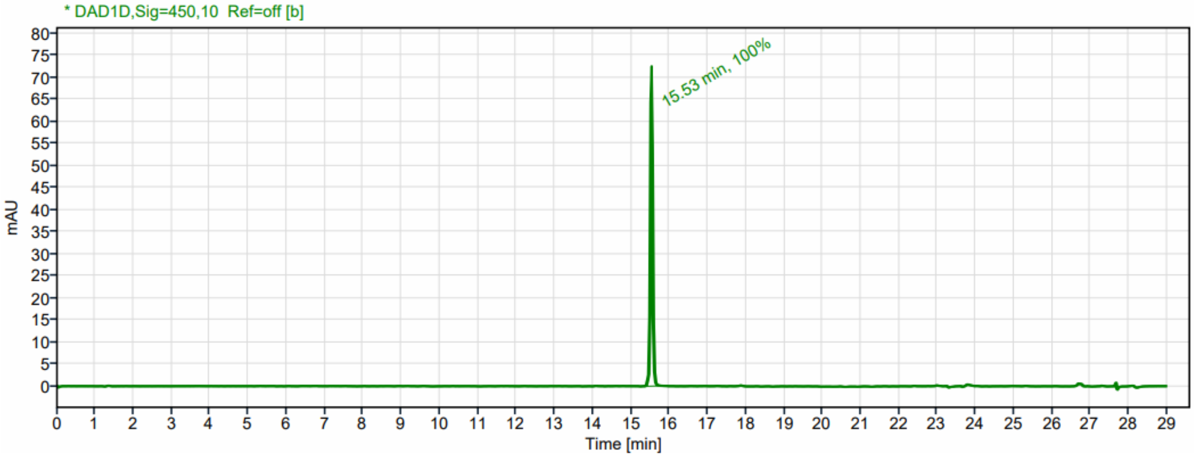

Compound A11

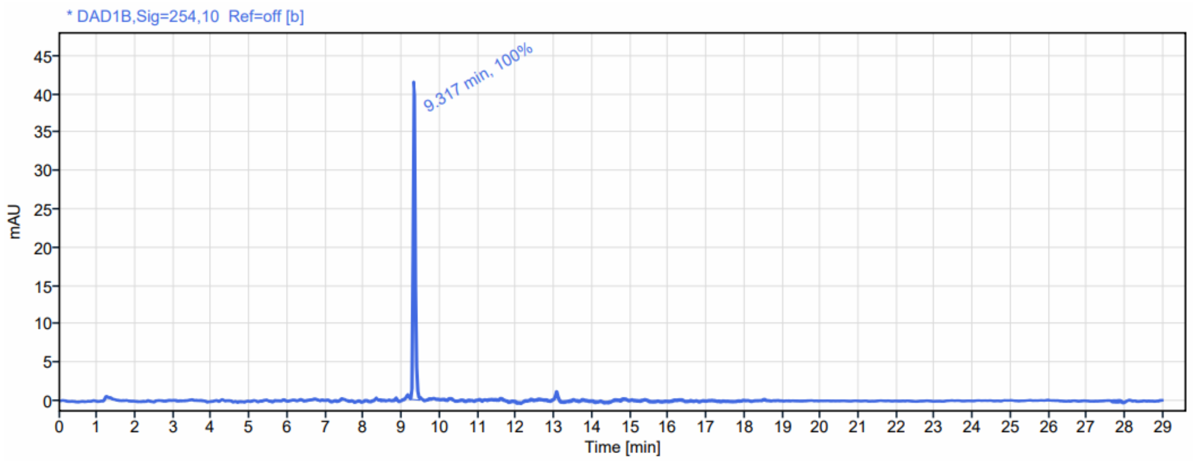

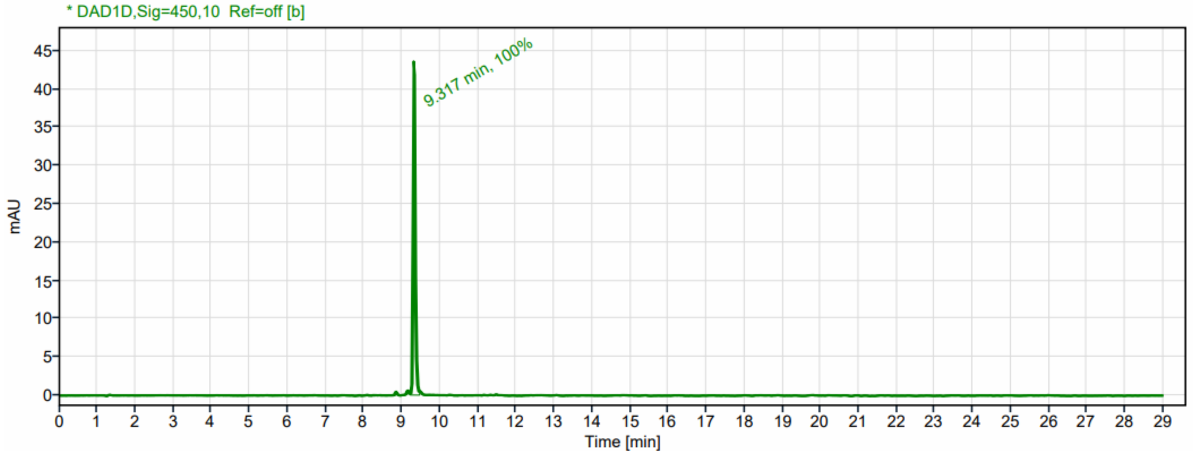

Compound A12

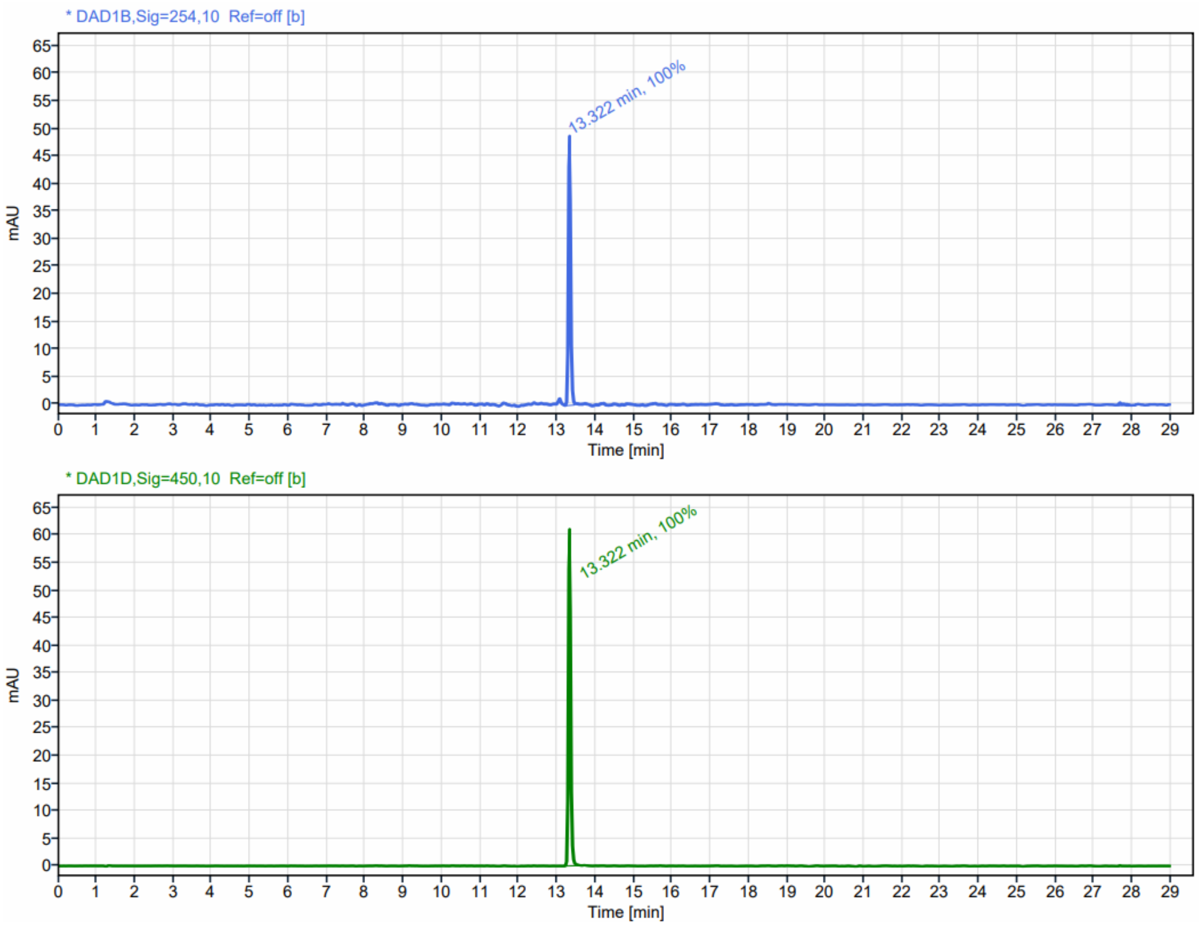

Compound A13

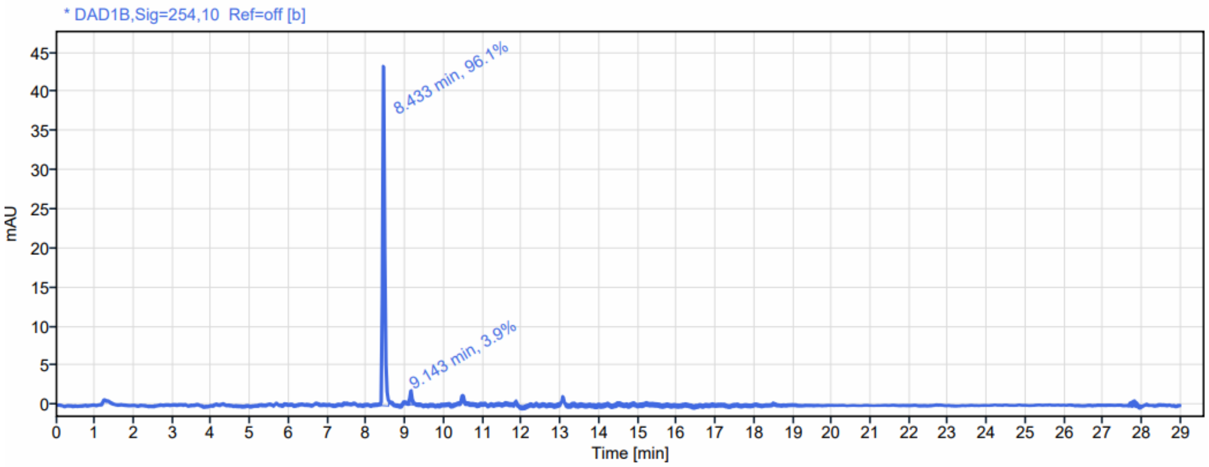

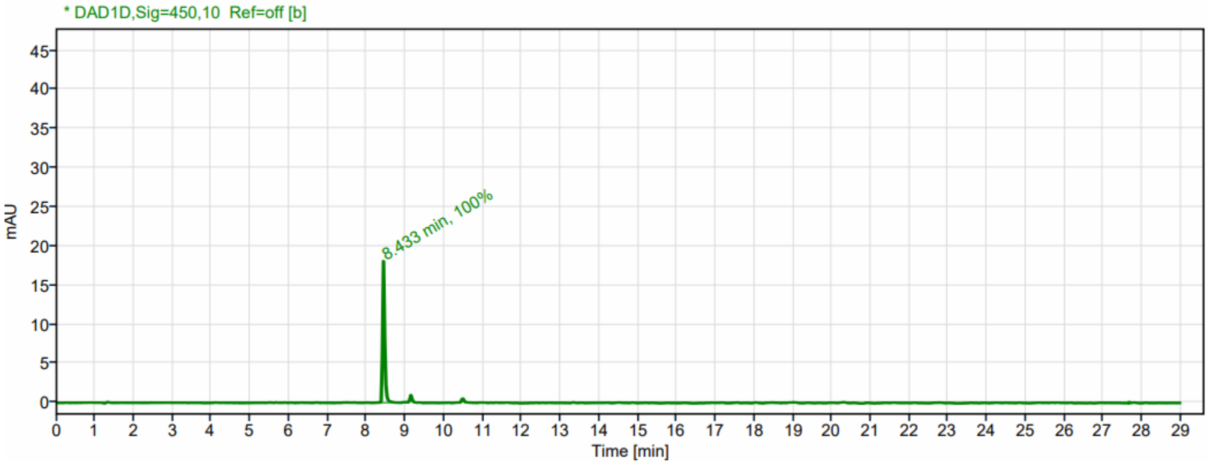

Compound A14

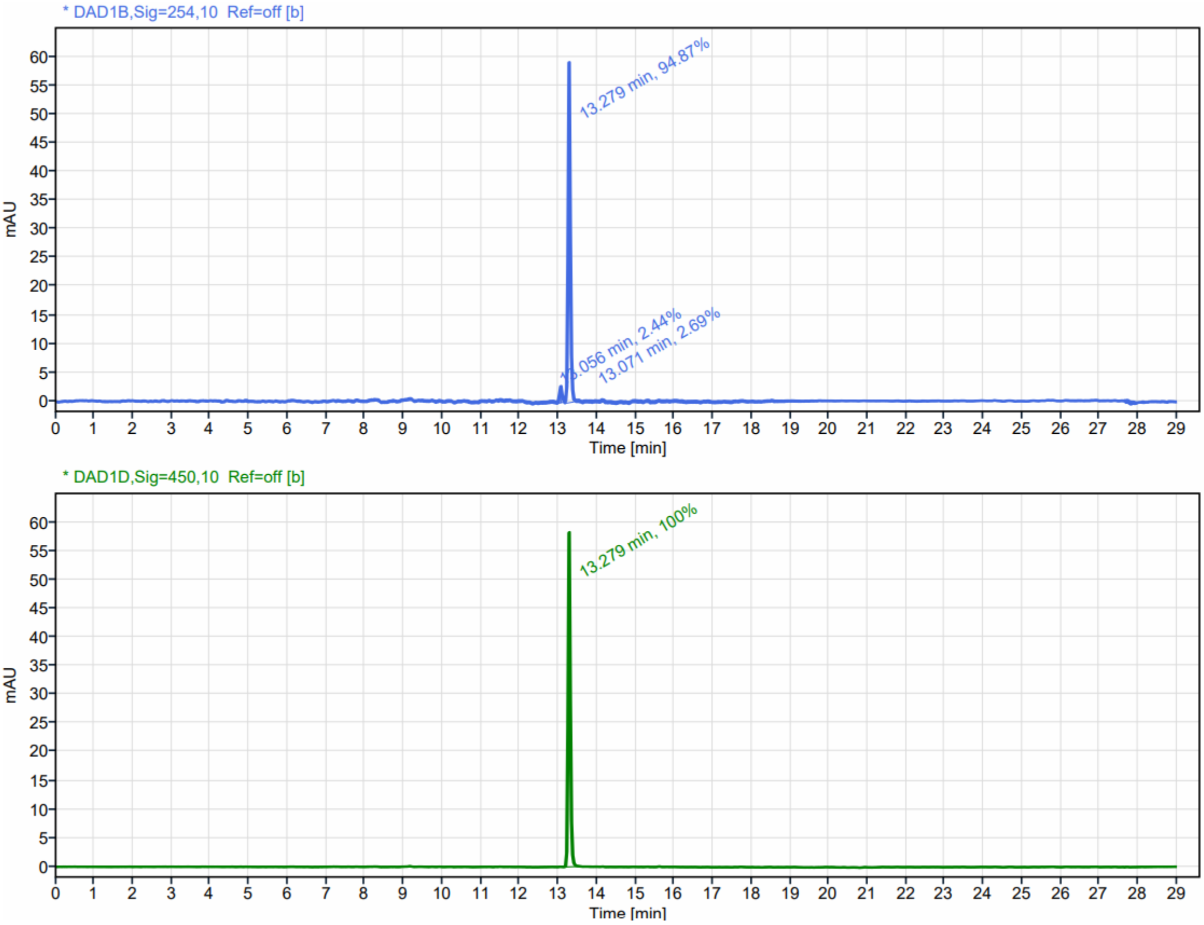

Compound A15

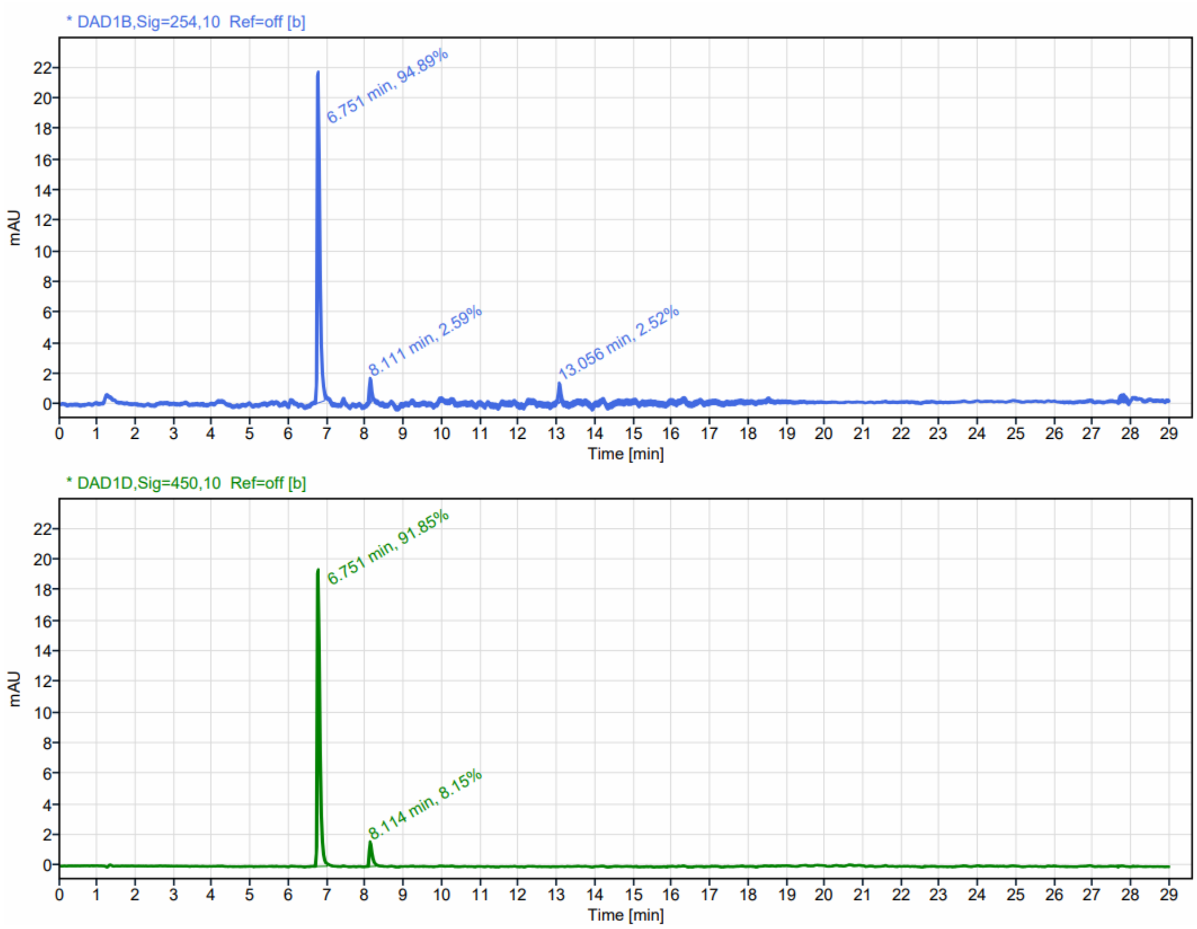

Compound A16

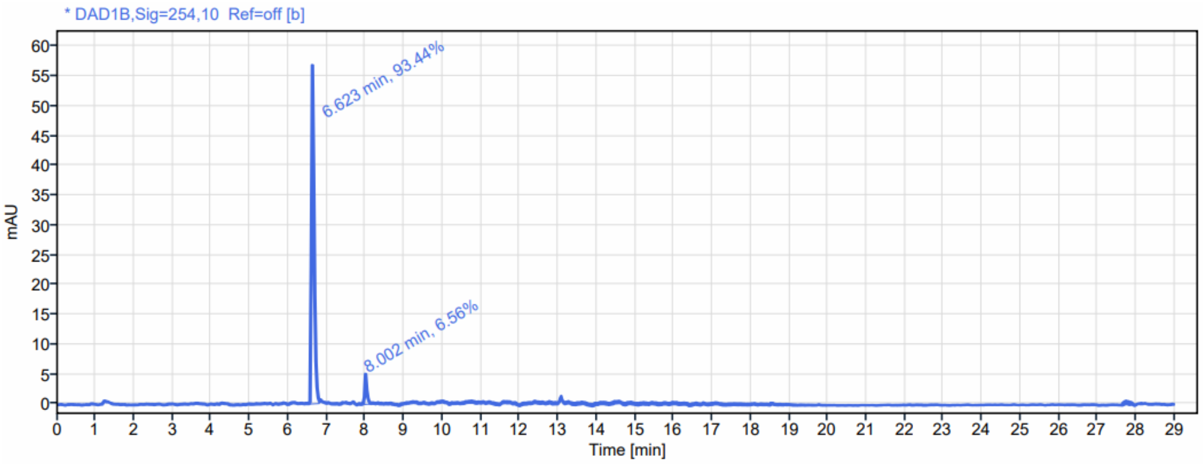

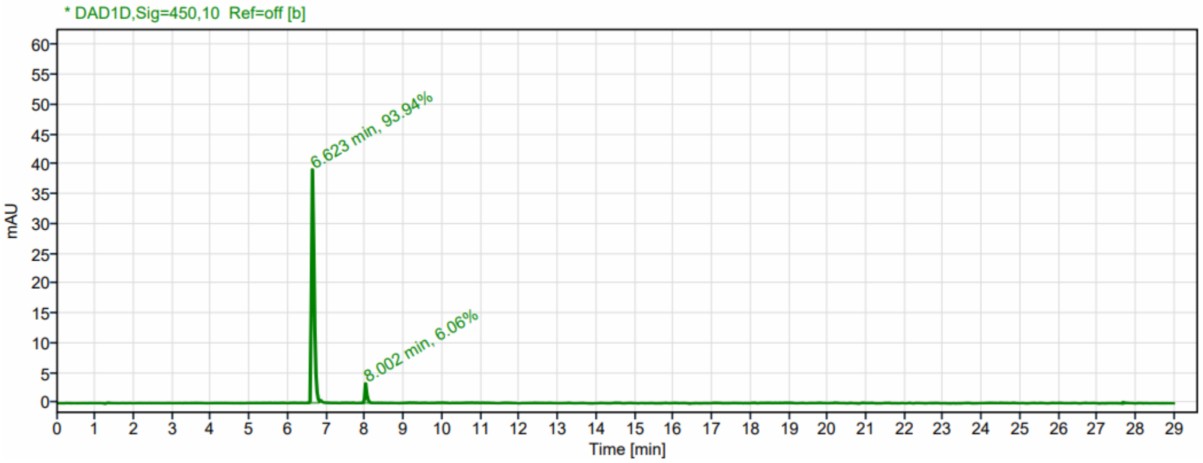

Compound A17

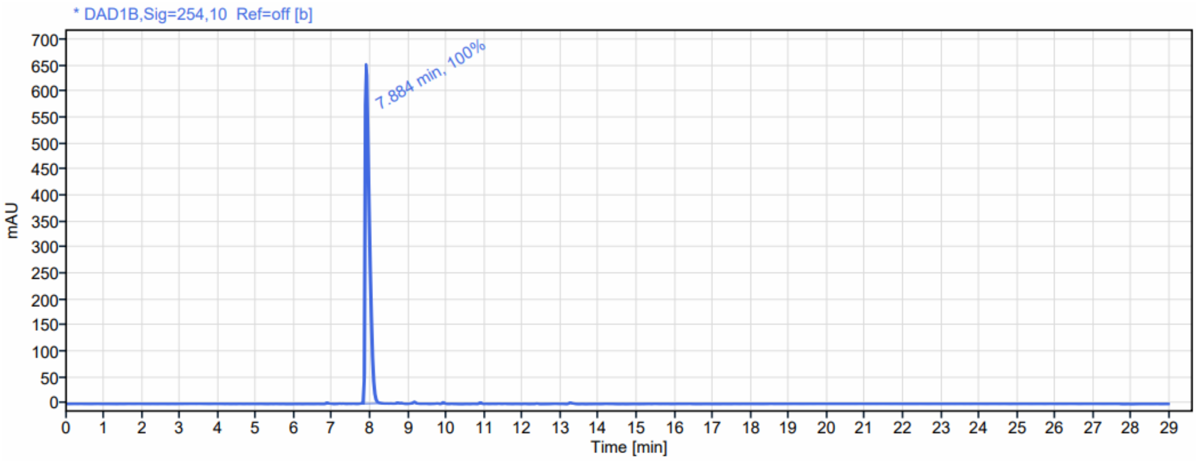

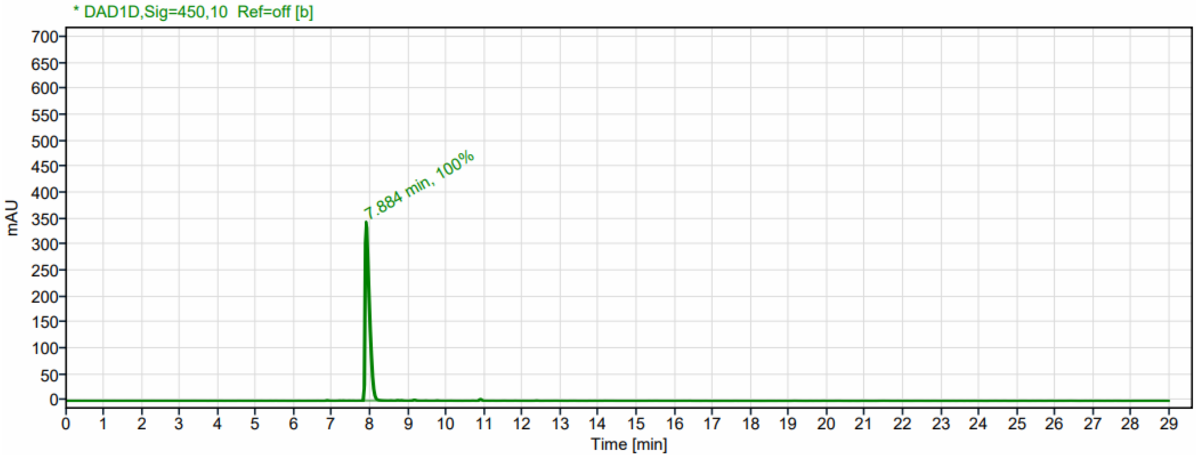

Compound A18

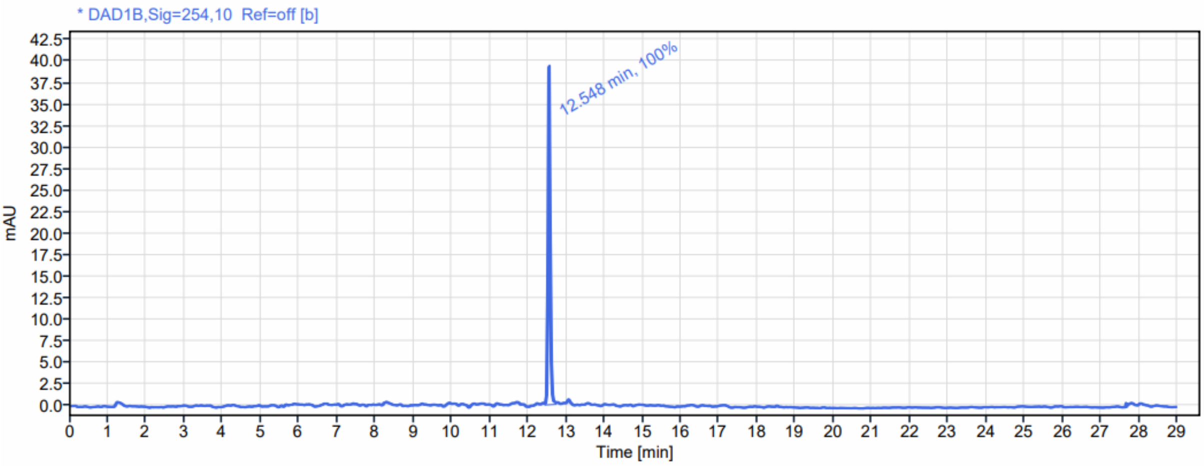

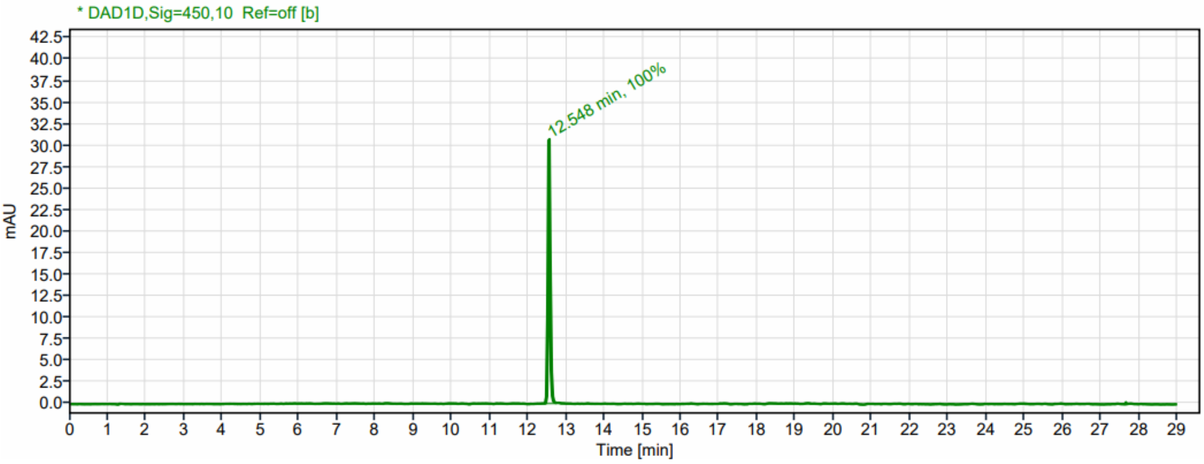

Compound A19

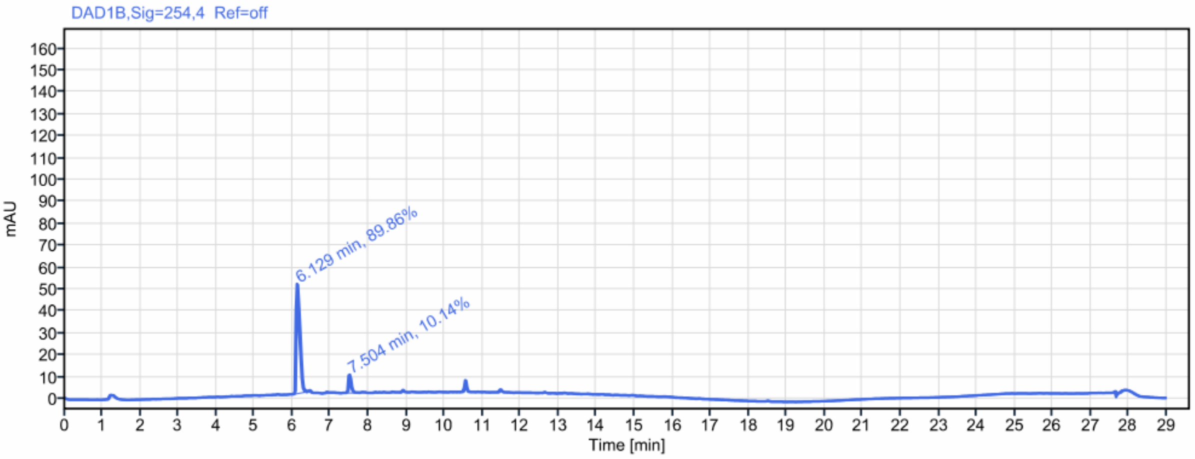

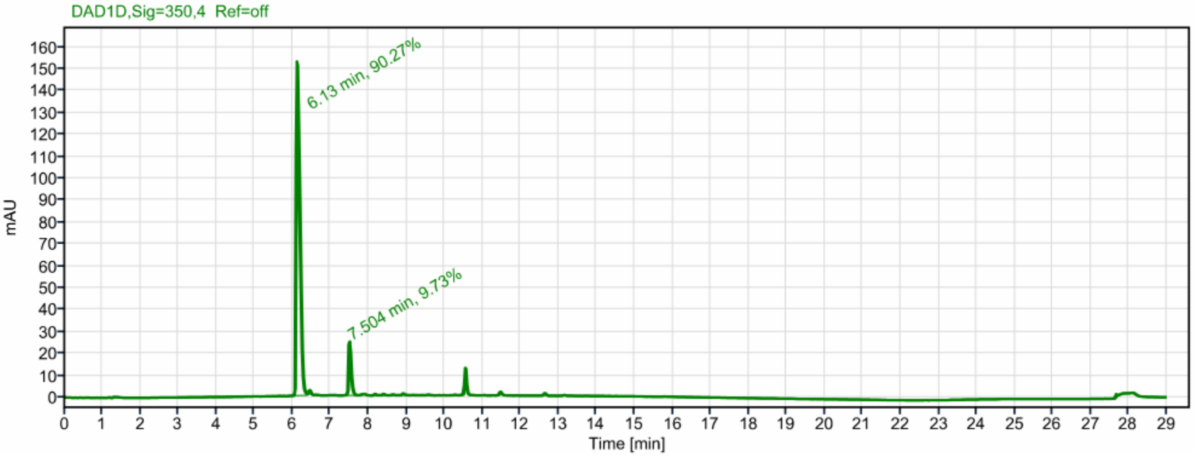

Compound A20

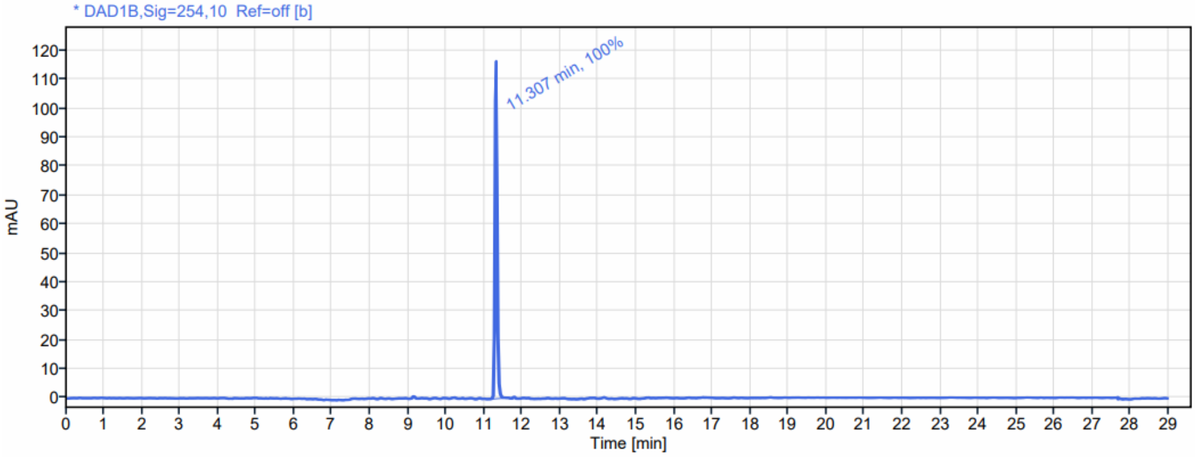

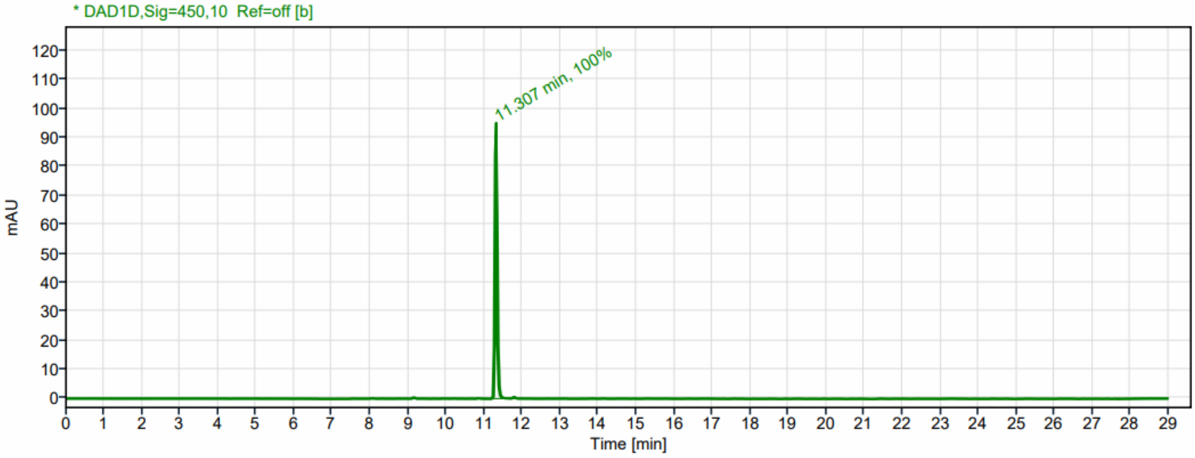

Compound A21

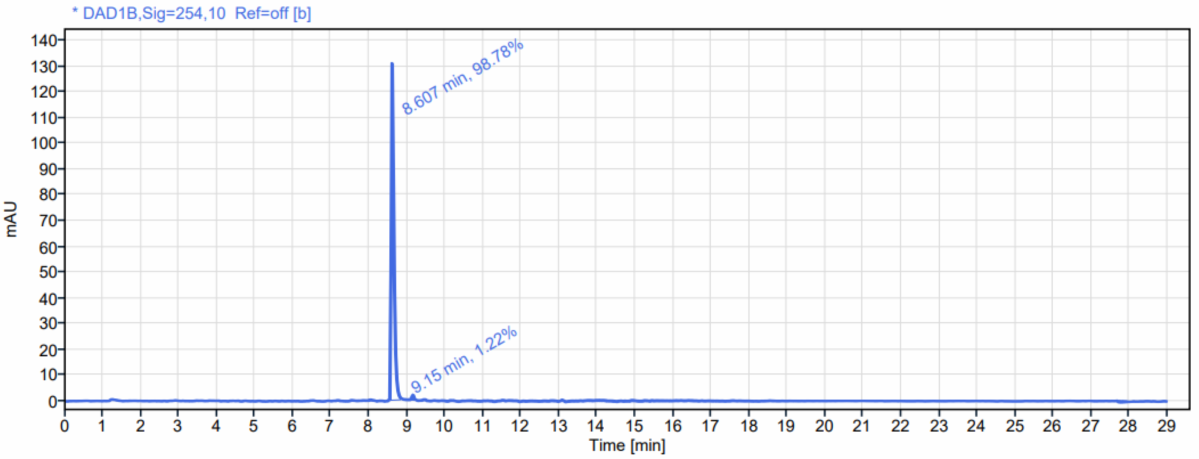

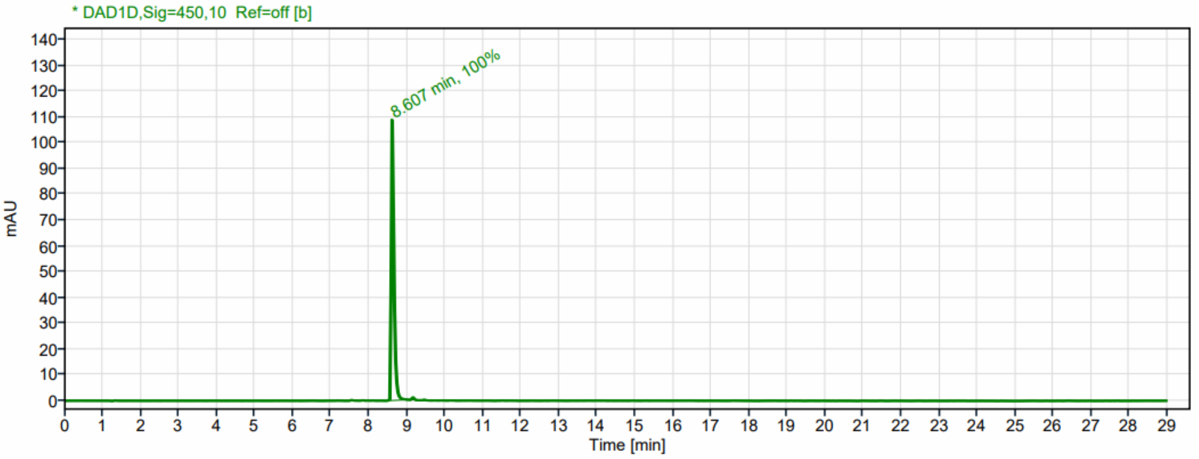

Compound A22

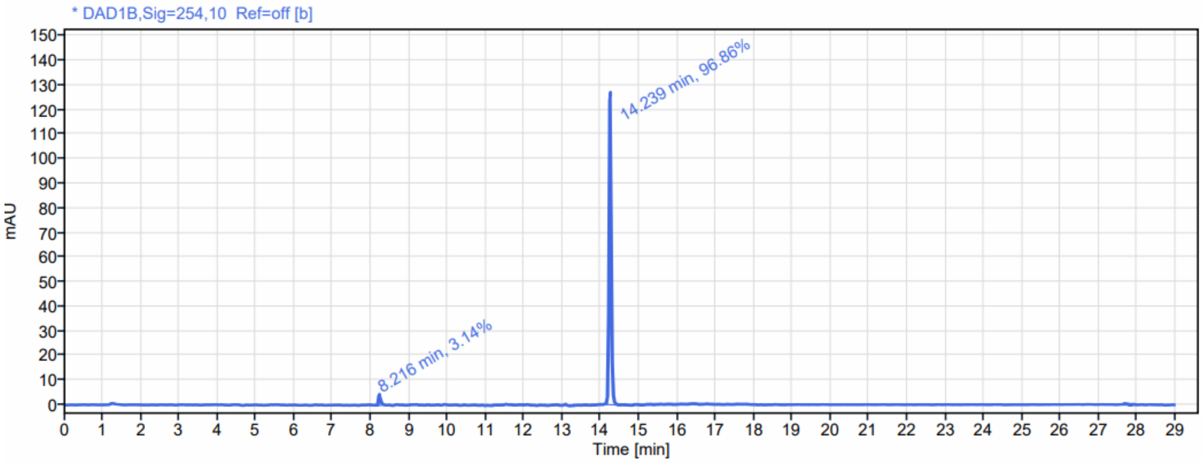

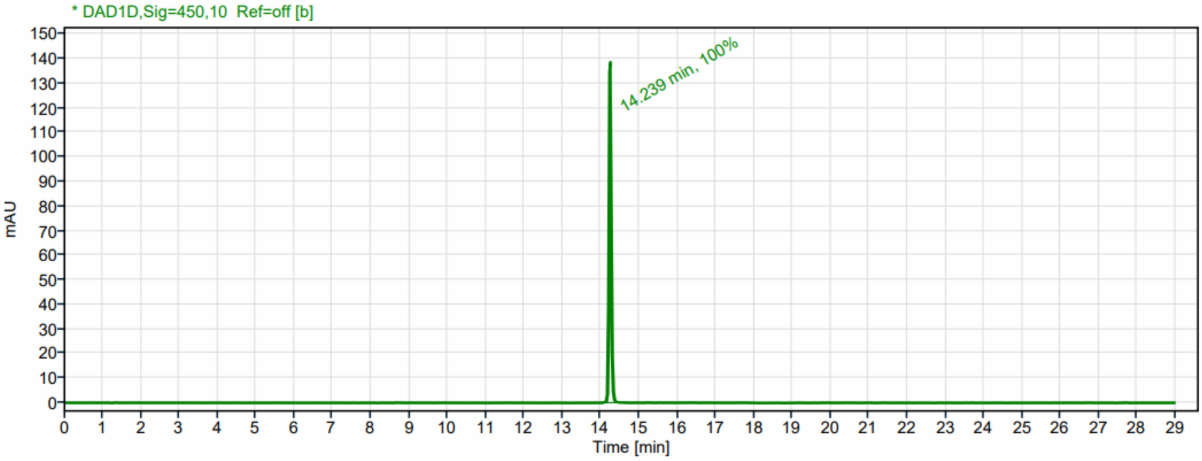

Compound A23

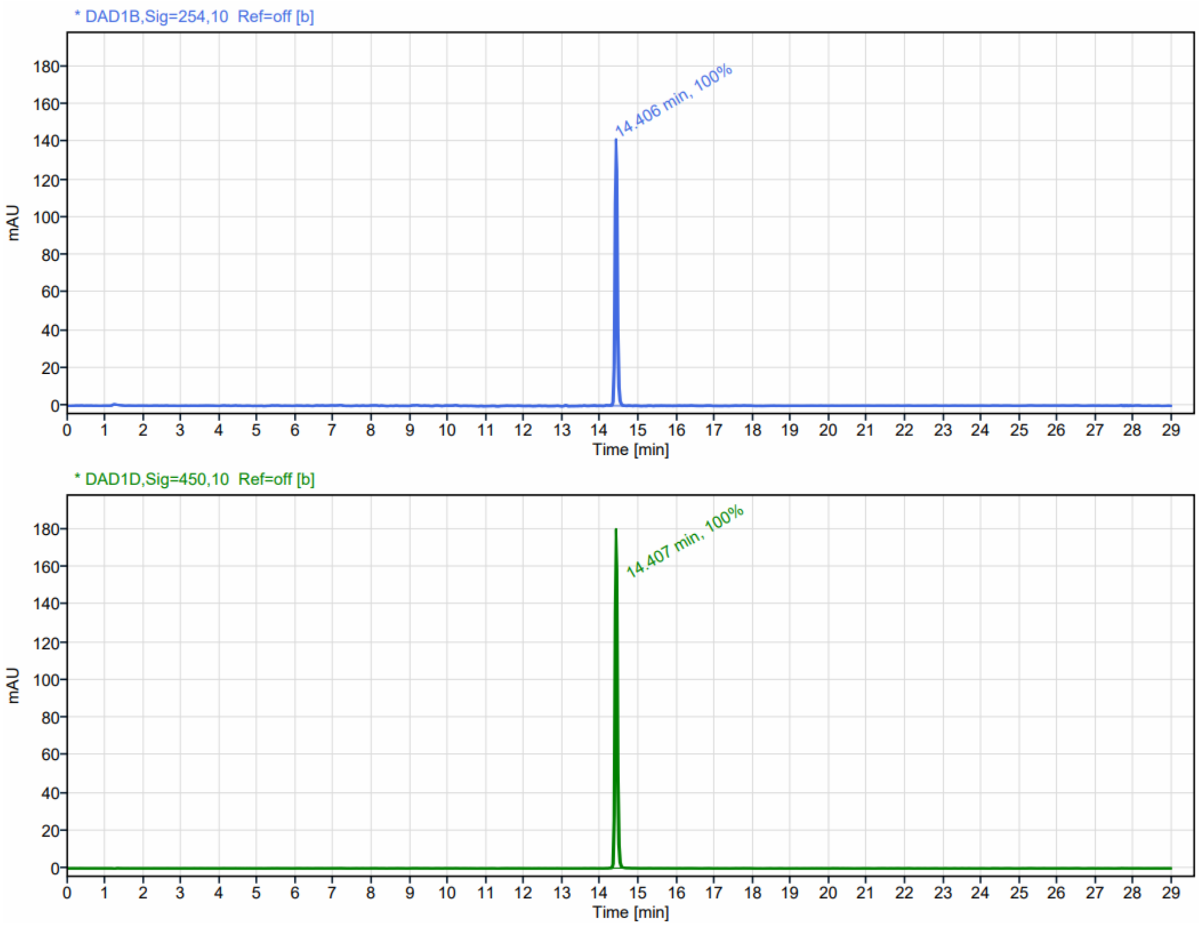

Compound A24

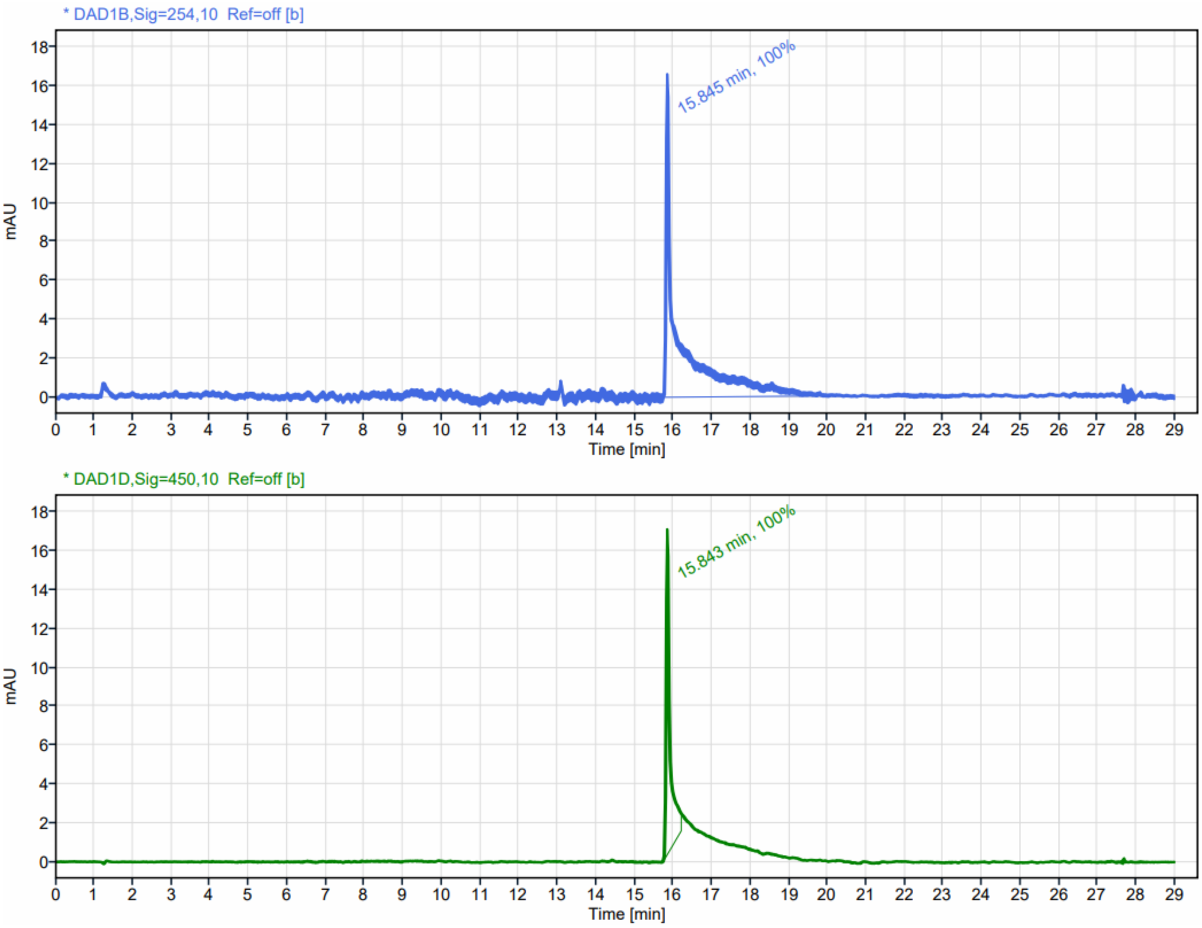

Compound A25

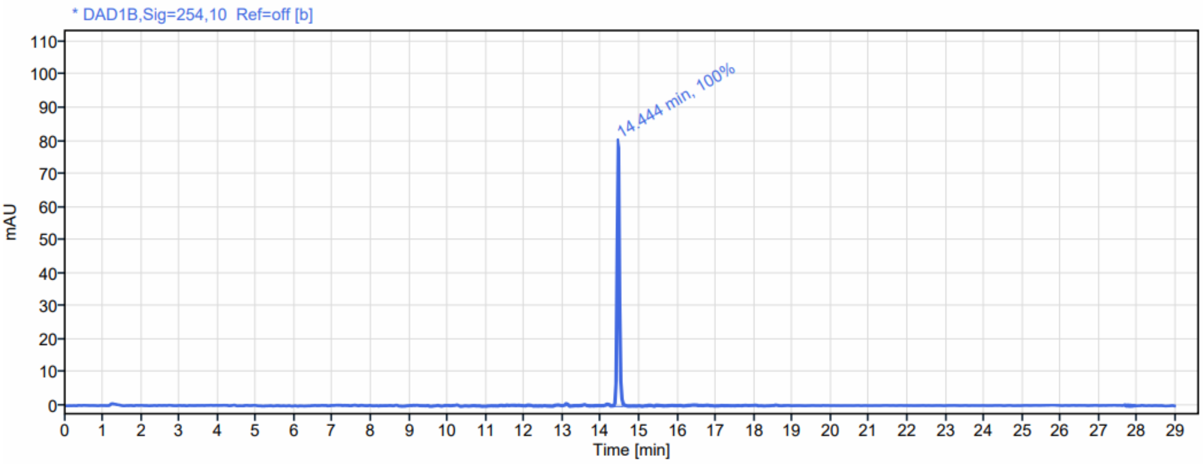

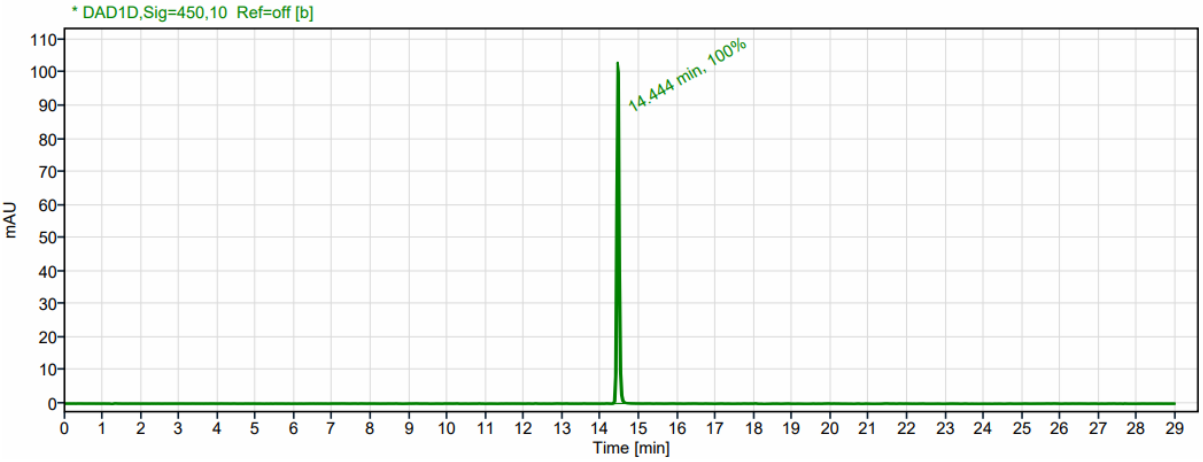

Compound A26

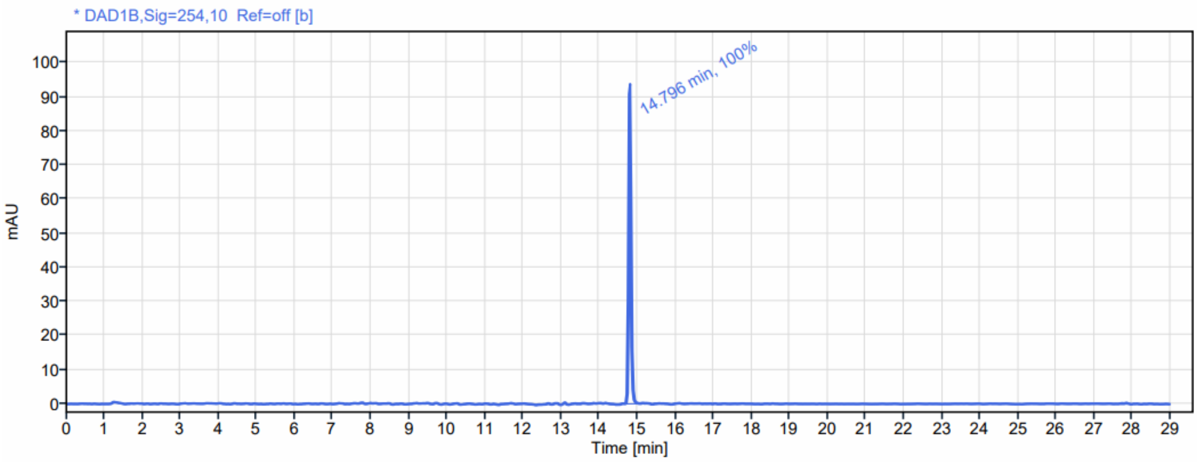

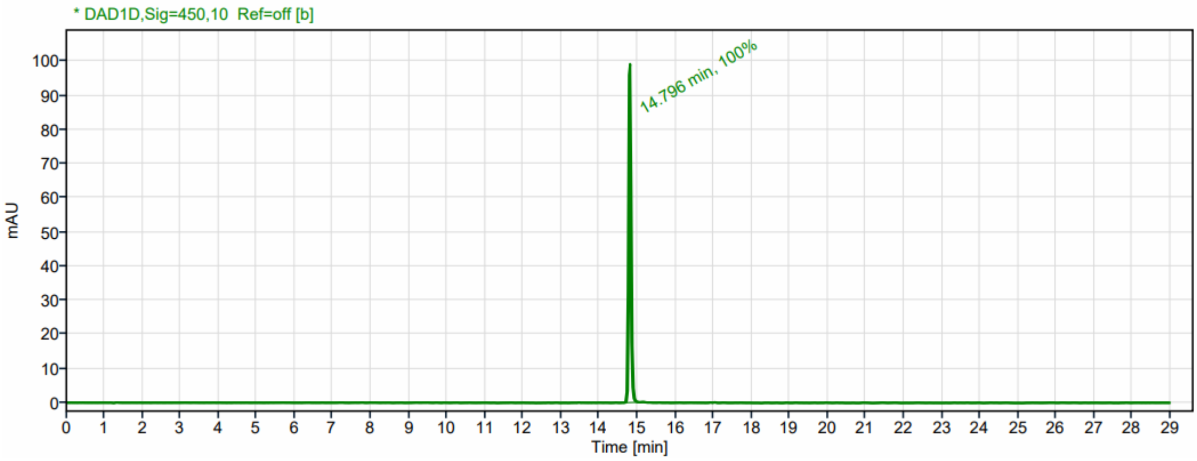

Compound A17-C

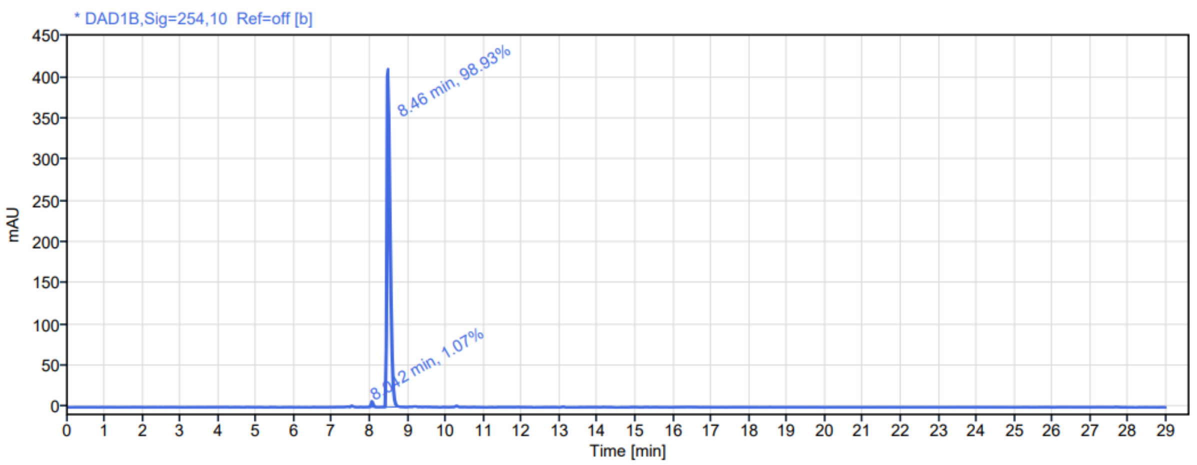

Compound A21-C

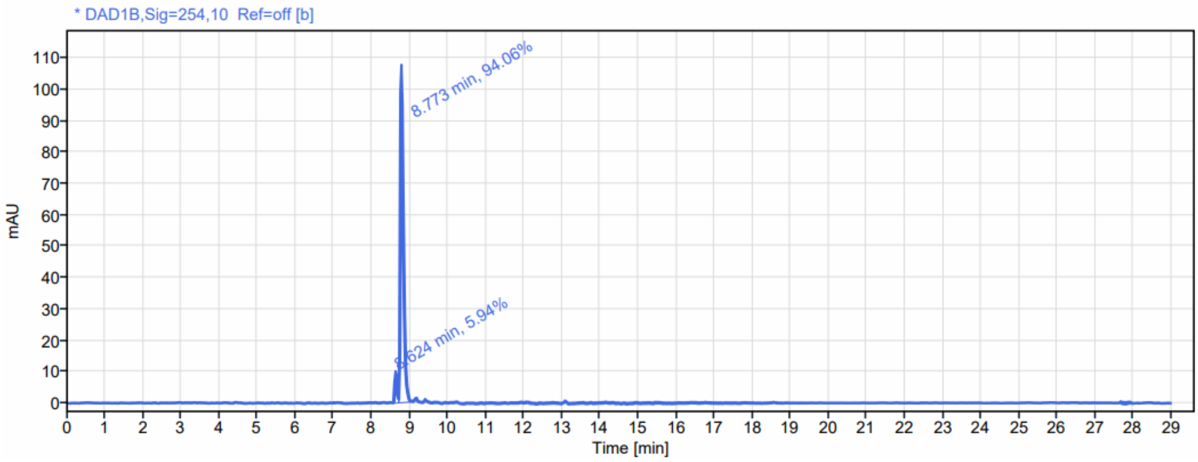

Compound A17-C-AA

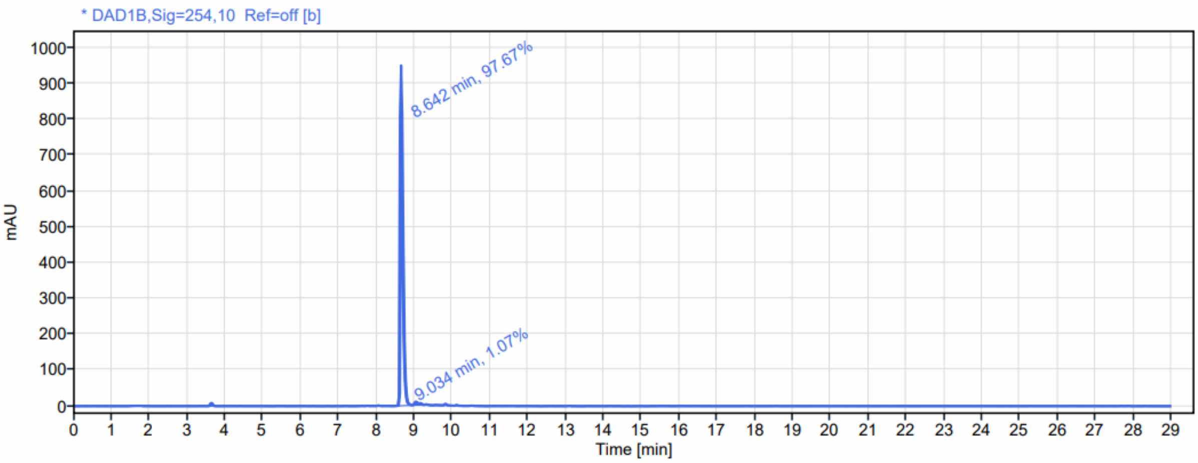

7. Supplementary References

[1] McPherson, M. J.; Hobson, A. D.; Hernandez, A., Jr.; Marvin, C. C.; Waegell, W.; Goess, C.; Oh, J. Z.; Shi, D.; Hayes, M. E.; Wang, L.; et al. An anti-TNF-glucocorticoid receptor modulator antibody-drug conjugate is efficacious against immune-mediated inflammatory diseases. *Sci. Transl. Med.* **2024**, *16*, eadd8936.

[2] a) Hobson, A. D.; McPherson, M. J.; Waegell, W.; Goess, C. A.; Stoffel, R. H.; Li, X.; Zhou, J.; Wang, Z.; Yu, Y.; Hernandez, A., Jr.; et al. Design and Development of Glucocorticoid Receptor Modulators as Immunology Antibody-Drug Conjugate Payloads. *J. Med. Chem.* **2022**, *65*, 4500-4533; b) Hobson, A. D.; McPherson, M. J.; Hayes, M. E.; Goess, C.; Li, X.; Zhou, J.; Wang, Z.; Yu, Y.; Yang, J.; Sun, L.; et al. Discovery of ABBV-3373, an Anti-TNF Glucocorticoid Receptor Modulator Immunology Antibody Drug Conjugate. *J. Med. Chem.* **2022**, *65*, 15893-15934.

[3] Salomon, P. L.; Reid, E. E.; Archer, K. E.; Harris, L.; Maloney, E. K.; Wilhelm, A. J.; Miller, M. L.; Chari, R. V. J.; Keating, T. A.; Singh, R. Optimizing Lysosomal Activation of Antibody-Drug Conjugates (ADCs) by Incorporation of Novel Cleavable Dipeptide Linkers. *Mol. Pharm.* **2019**, *16*, 4817-4825.
